# Context and culture associated with alcohol use amongst youth in major urban cities: A cross-country population based survey

**DOI:** 10.1371/journal.pone.0187812

**Published:** 2017-11-20

**Authors:** Anne W. Taylor, Bridgette M. Bewick, Alfred B. Makanjuola, Ling Qian, Valentina V. Kirzhanova, Paulo Alterwain

**Affiliations:** 1 Population Research & Outcome Studies, Discipline of Medicine, The University of Adelaide, Adelaide, South Australia, Australia; 2 School of Medicine, Leeds Institute of Health Sciences, University of Leeds, Leeds, United Kingdom; 3 Department of Behavioural Sciences, University of Ilorin Teaching Hospital, Ilorin, Nigeria; 4 Center for Health Education, PR Ministry of Health, Beijing, China; 5 Department of Epidemiology, Federal Medical Research Centre for Psychiatry and Narcology, Ministry of Health of the Russian Federation, Moscow, Russia; 6 ProHumanitas Foundation, Montevideo, Uruguay; Pennsylvania State University College of Medicine, UNITED STATES

## Abstract

**Background:**

Alcohol consumption patterns are dependent upon culture and context. The aim of this study was to interview people aged 18–34 year old living in four cities in different regions of the world to explore differences in a range of alcohol measures to assist in determining culturally appropriate alcohol initiatives for this age group.

**Method:**

Multistage random sampling was consistent across the four cities (Ilorin (Nigeria), Wuhan (China), Montevideo (Uruguay) and Moscow (Russia)). The questionnaire was forward and back translated into relevant languages and face-to-face interviewing undertaken. The data were weighted to the population of each city. Uni-variable analysis (ever consumed, first time consumed, age when drunk for first time, number of days consumed, type consumed) and logistic regression modeling were undertaken. The final model for each city was adjusted for age, sex, marital status, highest education and employment status. In total 6235 interviews were undertaken (1391 in Ilorin, 1600 in Montevideo, 1604 in Moscow and 1640 in Wuhan).

**Results:**

Alcohol was consumed by 96.4% in Montevideo, 86.1% in Moscow, 53.4% in Wuhan and 33.3% in Ilorin. There was very little difference by gender except Ilorin males were more likely to consume alcohol than females. Alcohol was consumed on more days for Ilorin males; Wuhan females consumed alcohol on the least number of days; Ilorin had the most abstainers; Montevideo and Moscow the highest proportion of light drinkers; Ilorin and Montevideo the highest proportion of heavy drinkers. Differences by type of alcohol were also apparent. The final logistic regression model provided different models including higher alcohol consumption rates for males, 25–34 years of age, divorced/separated marital status and employed part time for Ilorin respondents; males and higher educated for Montevideo; males, 25 to 29 years of age and higher educated for Moscow; and 25–29 years of age, non-married and vocationally trained for those in Wuhan.

**Conclusion:**

Alcohol consumption in these four cities does not increase with age as found in most high income countries. The alcohol consumption patterns during this stage of the life cycle are important to assess so that high level, as well as country-specific, planning and interventions can be implemented.

## Introduction

When assessing the range of alcohol prevalence rates worldwide, culture and context are important considerations [1–5]. In terms of alcohol consumption, countries differ by availability, customs, marketing practices, religious importance, government policies, cultural norms, age of legal consumption and importance of locally produced alcohol preferences [1,2,5,6]. There is a lack of data assessing alcohol consumption across countries with shortfalls in comparable methodology, sampling strategy, coverage, age ranges, risk and protective factors covered, and questions asked [1–3,5]. For younger alcohol consumers, where consumption is increasingly related to binge drinking and non-entrenched alcohol consumption patterns, negative social and health outcomes include increased road accidents, injuries, and ramifications associated with unplanned sex [3]. Alcohol consumption in younger age groups can be a precursor to more long-term alcohol problems and dependency on illicit drugs [7,8]. Accordingly, public health efforts to limit harmful excessive alcohol consumption are paramount. This study, assessing alcohol consumption patterns and behaviours in four distinctly different cities on four different continents using near identical methodology, addresses some of these shortcomings.

In terms of alcohol consumption in youth, literature has been published on the age of onset, the health effects on cognition and emotional development, the relationship between alcohol and drugs of dependence [8–10], harmful drinking in later life [11–13] and gender differences [6,14,15]. The importance of cross-country, cross-cultural comparison of young adults has been highlighted, especially when much of the research in alcohol consumption in this age group is conducted in high-income countries [3,14]. The aim of this study was to interview people aged 18–34 year old living in four cities in different regions of the world to explore differences in a range of alcohol measures to assist in providing evidence for the making of appropriate interventions and prevention initiatives.

## Methodology

Four cities were chosen pragmatically to be involved in the study based on diversity. Firstly, the city of Wuhan, the capital of Hubei Province in China, with an approximate population of over 9.8 million. The Hubei Province is one of the 34 Provincial-level Administrative Divisions of China. Secondly, the city of Moscow, the capital and the largest city of Russia, with an approximate population of 12 million was chosen. The third city was Ilorin which has an approximate population of 780,000 and is the administrative capital of Kwara State, one of the 36 States of Nigeria. Lastly, Montevideo, the capital of Uruguay which included parts of the departments of San Jose and Canelones, was included. The population of the department of Montevideo is approximately 1,319,000. Although a sample size of 210 was determined to achieve a 95% confidence limit with a confidence interval of ±5% around alcohol consumption estimates in each city (Epi-Info), a sample size of 1600 per city was determined so as to increase power and provide more precise data.

For each city, multistage random sampling was undertaken and sampling was kept consistent where possible across the four cities. The details for each specific city are contained in S1 Table. Actual number of interviews conducted is included in S2 Table. For all four cities, in each randomly selected household, the person with the most recent birthday, aged between 18 and 34 years, and who had lived in the city for at least six months, was eligible and was invited to participate in the study. In Montevideo and Moscow substitutes for non-respondents were allowed by following a protocol of systematic sampling of replacement houses. For the other two cities there was no replacement for non-respondents.

The average length of interviews was 15 minutes although for the cities that included additional city-specific questions the time was extended. The questionnaire was forward translated into the relevant languages (eg English to Chinese) and back translated (eg Chinese to English) to ensure the questions were conceptually and culturally equivalent between the cities. (See S1 Questionnaire in Chinese. S2 Questionnaire in English, S3 Questionnaire in Hausa, S4 Questionnaire in Ibo, S5 Questionnaire in Russian, S6 Questionnaire in Spanish, S7 Questionnaire in Yoruba).

In Wuhan, ethical clearance was obtained from the Hubei Provincial CDC (Hubei Provincial Society for Health Promotion & Cigarette-smoking Control, HBPHP&CCS-2014-01), in Moscow from the Ethics Committee on the NRC on Addictions, in Ilorin from the Ethics Research (independent Review Board) Committee of the University of Ilorin (UERC/ASN/2014/007) and in Montevideo from the Pro Humanities Ethics Committee. In addition, where appropriate, permission was obtained from community leaders, appropriate government officials, and significant personnel within the study communities. Respondents could terminate the interview at any time, or choose not to answer any question. In households where there was alcohol and substance abuse, referrals to recognized hospitals/caregivers for appropriate management were provided. No details on how many respondents used this service was collected.

Prior to the main survey, a pilot study of 25 to 50 interviews was conducted in each city assessing question comprehension and length of interview. Data collection was conducted face-to-face and interviewer-administered via paper-and-pencil. Interviewers read out the questions and, if necessary, prompt cards were used. Interviews were conducted in an environment where respondents felt most comfortable, and where their privacy was respected.

In Wuhan, data collection commenced on 25th October 2014 and concluded on 29th April 2015 (186 days). The interviewers were college students who received half a day’s training. Wuhan interviews were conducted in Mandarin and the Wuhan dialect. In Moscow, data collection commenced on 15th April 2015 and concluded on 23rd May 2015 (38 days). The interviewers had one day training and the interviews were conducted in Russian. In Ilorin, data collection commenced on 15th September 2014 and concluded on 18th November 2014 (64 days). The interviewers were briefed over two days and the interviews were conducted in English, Yoruba, Hausa, and Ibo. In Montevideo, data collection commenced on 11th November 2014 and concluded on 11th June 2015 (181 days). The interviewers were trained for six hours and the interviews were conducted in Spanish. At least 10% of all interviews were selected for validation in each city. This included quality control checks on each day’s interviews for inconsistency and inappropriate answers.

To reduce potential biases and to make sure that the results accurately reflect the population of interest, the data were weighted. In Wuhan the data were weighted to the stratified target population information provided by Hubei Provincial Institute for Health Education in 2014. The data were weighted by Administrative District (urban vs sub-urban area), age, gender, and probability of selection in the household to the Census data (The 6th National Census data in 2010). Weight values ranged between 0.285 and 4.300. In Moscow the weighting of data was undertaken by the research team at the Epidemiology Department, Federal Medical Research Centre for Psychiatry and Narcology, Russian Federation Ministry of Health. Weights were calculated according to gender and age of respondents. Statistical data for weighting were taken from the 2010 census. Weights for respondents were distributed within the range from 0.569 to 1.475. In Ilorin the data were weighted to the Federal Republic of Nigeria 2006 Population and Housing Census (www.population.gov.ng). The data were weighted by local government area (metro vs country), age, gender, and probability of selection in the household to the Census data (range 0.118 to 6.641). In Montevideo the data were weighted to the 2011 Census by Department (Montevideo, Canelones, and San Jose), age, gender, and probability of selection in the household to the Census data (2011 Census Instituto Nacional de Estadistica, Uruguay, http://www.ine.gub.uy/web/guest/censos-2011). The weight values ranged between 0.353 and 5.743.

Alcohol consumption questions included: Have you ever consumed alcohol (excluding sips). How old were you the first time you had a drink of an alcoholic beverage (excluding sips)? How old were you the first time you got drunk? During the past 12 months, how often did you drink beer, wine, spirits (e.g., vodka, gin, whisky, brandy), or any other alcohol beverage, even in small amounts? During the past 12 months, how many alcoholic drinks did you have on a typical day when you drank alcohol? Overall quantity-frequency (QF) (i.e. usual frequency of drinking by usual number of drinks consumed per drinking occasion) was calculated by multiplying the responses to the above two questions (how often and how many) with 25 or more drinks (coded as 25), 19 to 24 drinks (coded as 21.5), 16 to 18 drinks (coded as 17), 12 to 15 drinks (coded as 13.5), 9 to 11 drinks (coded as 10), seven to eight drinks (coded as 7.5), five to six drinks (coded as 5.5), three to four drinks (coded as 3.5), two drinks (coded as 2), one drink (coded as 1) and less than 1 full drink (coded as 0.5). The variables were recoded into four drinking status groups: 0 drinks = Abstainers, > 0 but less than 365 drinks/year = Light Drinkers, 365–729 drinks/year = Moderate Drinkers, 730 or more drinks/year = Heavier Drinkers. No distinction was made between lifetime abstainers and former drinkers with alcohol consumption assessed during the past 12 months only.

The beverage specific quantity frequency (BSQF) method employed two questions for each of the three types of alcoholic beverages: beer, wine and spirits. During the past 12 months, how often did you drink [beer/wine/ spirits/ any other alcohol beverage], even in small amounts? And on a typical day when you drank [beer/wine/ spirits], how much [beer/wine/ spirits] did you drink? BSQF for each specific alcohol beverage was calculated by multiplying the responses to the two questions. Respondents were provided with country specific visual references to beverage specific drink sizes in order to facilitate reporting of number of drinks in standard sizes.

Frequency of heavy episodic drinking (HED) was assessed by using the frequency of largest number of drinks within a 24-hour period (how often) by number of drinks consumed per largest drinking occasion (how many) with heavy drinking defined as five or more drinks for three levels of frequency of heavy drinking (at least once a week, at least twice a month, at least once a month).

Uni-variable analysis was undertaken on ever consumed alcohol, age started drinking, age first got drunk, mean number of days alcohol was consumed in the previous 12 months, HED, QF and BSQF. Logistic regression modeling was undertaken for current drinkers for each city. The model was adjusted for age, sex, marital status, highest education, region and employment status.

## Results

In total n = 6235 interviews were undertaken (1391 in Ilorin, 1600 in Montevideo, 1604 in Moscow and 1640 in Wuhan). See S2 Table for specific response rates. The mean age of respondents was 25.1 (sd 4.8) years in Ilorin, 25.8 (sd 4.9) in Montevideo, 26.2 (sd 5.0) in Moscow and 25.2 (sd 4.5) in Wuhan. Overall, 49.4% of respondents were male (47.6% in Ilorin, 49.2% in Montevideo, 49.2% in Moscow and 51.4% in Wuhan). Demographic indicators are highlighted in S3 Table.

Table 1 shows the proportion ever consuming alcohol by gender with overall higher alcohol consumption in Montevideo (96.4%–95% ci 95.1–97.3). The lowest overall prevalence rate was in Ilorin (33.3%–95% ci 30.5–35.3). There was very little difference by gender for all cities except Ilorin where the prevalence estimate for males (44.2%) was nearly double that of females (23.5%). Table 2 is the prevalence of HED. Moscow respondents indicated more episodic drinking behaviours (4.7% (95% ci 3.7–5.9) for HED at least once a week. Both Moscow (15.9% (95% ci 13.9–18.3) and Montevideo (15.9%–95% ci 14.1–18.27.8) had high levels of HED at least once a month. The lowest rate was 1.6% (95% ci 1.1–2.6) for Wuhan respondents on a weekly basis.

**Table 1:** Ever consumed alcohol (by gender) by city/country.

	Ilorin (Nigeria)		Montevideo (Uruguay)		Moscow (Russia)		Wuhan (China)	
Ever consumed alcohol	n	% (95% CI)	n	% (95% CI)	n	% (95% CI)	n	% (95% CI)
**Sex**								
Male	293	44.2 (40.5–48.0)	757	96.2 (94.7–97.4)	693	88.3 (85.8–90.3)	437	51.9 (48.5–55.2)
Female	171	23.5 (20.5–26.7)	784	96.5 (95.0–97.5)	683	84.0 (81.3–86.3)	438	55.1 (51.6–58.5)
**Overall**	**464**	**33.3 (30.5–36.3)**	**1542**	**96.4 (95.1–97.3**	**1376**	**86.1 (84.3–87.8)**	**875**	**53.4 (50.4–56.4)**

**Table 2:** Heavy episodic drinking (HED) (five or more drinks) for three levels of frequency of heavy drinking (at least once a week, at least twice a month at least once a month) and city/country.

	Ilorin (Nigeria)		Montevideo (Uruguay)		Moscow (Russia)		Moscow (Russia)	
Heavy Episodic Drinking (HED)	n	% (95% CI)	n	% (95% CI)	n	% (95% CI)	n	% (95% CI)
At least once a week	51	3.7 (2.8–4.9)	69	4.5 (3.5–5.9)	67	4.7 (3.7–5.9)	26	1.6 (1.1–2.6)
At least twice a month	65	4.7 (3.7–6.1)	144	9.4 (7.8–11.3)	124	8.6 (7.3–10.2)	61	3.9 (2.9–5.3)
At least once a month	71	5.1 (4.1–6.5)	244	15.9 (13.9–18.3)	229	15.9 (14.1–18.0)	91	5.8 (4.5–7.4)

CI: Confidence Interval.

Note: The weighting of data can result in rounding discrepancies or totals not adding. Don’t know/refused not included.

Table 3 details the mean age, by gender, of the first time alcohol was consumed and the mean age when drunk for the first time. The table also highlights the mean number of days alcohol was consumed in the last 12 months (for those who drank alcohol) with the highest rate for Ilorin males (139.2 days) and the lowest in Wuhan females (28.3 days).

**Table 3:** Descriptive statistics of age (in years) started drinking, age (in years) when first got drunk, and mean number of drinks consumed by gender and city/country.

	Male					Female				
	n	Mean	S.D	Median	Range	n	Mean	S.D	Median	Range
**Age started drinking**										
Ilorin (Nigeria)	289	19.0	3.4	19.0	10–30	159	19.8	2.5	20.0	12–27
Montevideo (Uruguay)	751	15.0	2.2	15.0	7–25	783	15.8	2.4	16.0	9–34
Moscow (Russia)	671	16.1	1.8	16.0	6–26	654	16.7	2.1	17.0	7–29
Wuhan (China)	392	18.5	2.9	18.0	1–30	394	18.8	2.6	19.0	1–29
**Age first got drunk**										
Ilorin (Nigeria)	171	20.6	4.0	20.0	10–29	67	20.7	1.8	21.0	17–27
Montevideo (Uruguay)	654	16.4	2.6	16.0	9–30	568	17.2	2.7	17.0	9–34
Moscow (Russia)	485	17.0	1.9	17.0	11–29	290	17.6	2.2	17.0	14–29
Wuhan (China)	189	20.3	3.5	20.0	3–30	155	20.7	3.8	20.0	1–33
**Mean number of days alcohol consumed in the last 12 months**										
Ilorin (Nigeria)	189	139.2	147.5	78.0	1–365	93	95.6	106.2	30.0	1–365
Montevideo (Uruguay)	710	62.0	67.8	30.0	1–365	693	41.7	56.9	30.0	1–365
Moscow (Russia)	636	71.5	72.4	30.0	1–365	597	24.7	33.2	12.0	1–259
Wuhan (China)	374	34.3	68.6	12.0	1–365	377	28.3	56.6	8.5	1–365

Don’t know/refused not included. SD: Standard deviation. Only includes participants that reported drinking alcohol.

The proportion by drinker type (QF) in each of the cities is detailed in Table 4 with more abstainers in Ilorin (79.8%–95% ci 77.3–82.1), higher proportion of light drinkers in Montevideo (71.6%–95% ci 69.0–74.0) and Moscow (68.9%–95% ci 66.4–71.2) with a higher proportion of the heavy drinkers in Ilorin (5.0%–95% ci 3.9–6.4) and Montevideo (5.6%–95% ci 4.5–7.0). Differences by gender are also reported.

**Table 4:** QF- Four drinking status group (abstainers, light drinker, moderate drinker, and heavier drinker) by city/country.

	Ilorin (Nigeria)		Montevideo (Uruguay)		Moscow (Russia)		Wuhan (China)	
	n	% (95% CI)	n	% (95% CI)	n	% (95% CI)	n	% (95% CI)
Abstainer	1110	79.8 (77.6–81.8) *	197	12.3 (10.8–14.0) *	367	23.1 (21.1–25.2) *	885	54.2 (51.7–56.6) *
Lighter	183	13.2 (11.5–15.1) *	1145	71.6 (69.3–73.7) *	1096	68.9 (66.6–71.1) *	702	42.9 (40.6–45.4) *
Moderate	28	2.0 (1.4–2.9) *	169	10.5 (9.1–12.1) *	72	4.5 (3.6–5.7)	28	1.7 (1.2–2.5) *
Heavier	70	5.0 (4.0–6.3) *	90	5.6 (4.6–6.8) *	56	3.5 (2.7–4.5)	19	1.2 (0.7–1.8) *
**Overall**	**1391**	**100.0**	**1600**	**100.0**	**1592**	**100.0**	**1634**	**100.0**
**Male**								
Abstainer	473	71.5 (67.9–74.8) *	77	9.7 (7.9–12.0) *	152	19.4 (16.8–22.3) *	467	55.6 (52.2–58.9) *
Lighter	113	17.1 (14.4–20.2) *	514	65.3 (62.0–68.6) *	516	65.8 (62.4–69.1) *	348	41.4 (38.1–44.8) *
Moderate	22	3.3 (2.2–5.0) *	120	15.3 (12.9–18.0) *	63	8.1 (6.4–10.2)	16	1.9 (1.1–3.0) *
Heavier	54	8.1 (6.3–10.4) *	76	9.6 (7.8–11.9) *	53	6.7 (5.2–8.7)	10	1.2 (0.6–2.2) *
**Overall**	**662**	**100.0**	**787**	**100.0**	**784**	**100.0**	**841**	**100.0**
**Female**								
Abstainer	637	87.3 (84.7–89.5) *	120	14.8 (12.5–17.4) *	216	26.7 (23.8–29.9) *	418	52.7 (49.2–56.1) *
Lighter	70	9.6 (7.7–12.0) *	631	77.6 (74.6–80.3) *	580	71.8 (68.6–74.8) *	353	44.6 (41.1–48.0) *
Moderate	6	0.8 (0.4–1.8) *	48	5.9 (4.5–7.8) *	9	1.1 (0.5–2.0) *	13	1.6 (0.9–2.7)
Heavier	17	2.3 (1.4–3.6) *	14	1.7 (1.0–2.8)	3	0.4 #	9	1.2 (0.6–2.2)
**Overall**	**729**	**100.0**	**813**	**100.0**	**808**	**100.0**	**793**	**100.0**

CI: Confidence Interval.

# Insufficient numbers.

Note: The weighting of data can result in rounding discrepancies or totals not adding. Don’t know/refused not included.

* Significantly different to all other categories combined (p<0.05).

**Fig 1:**
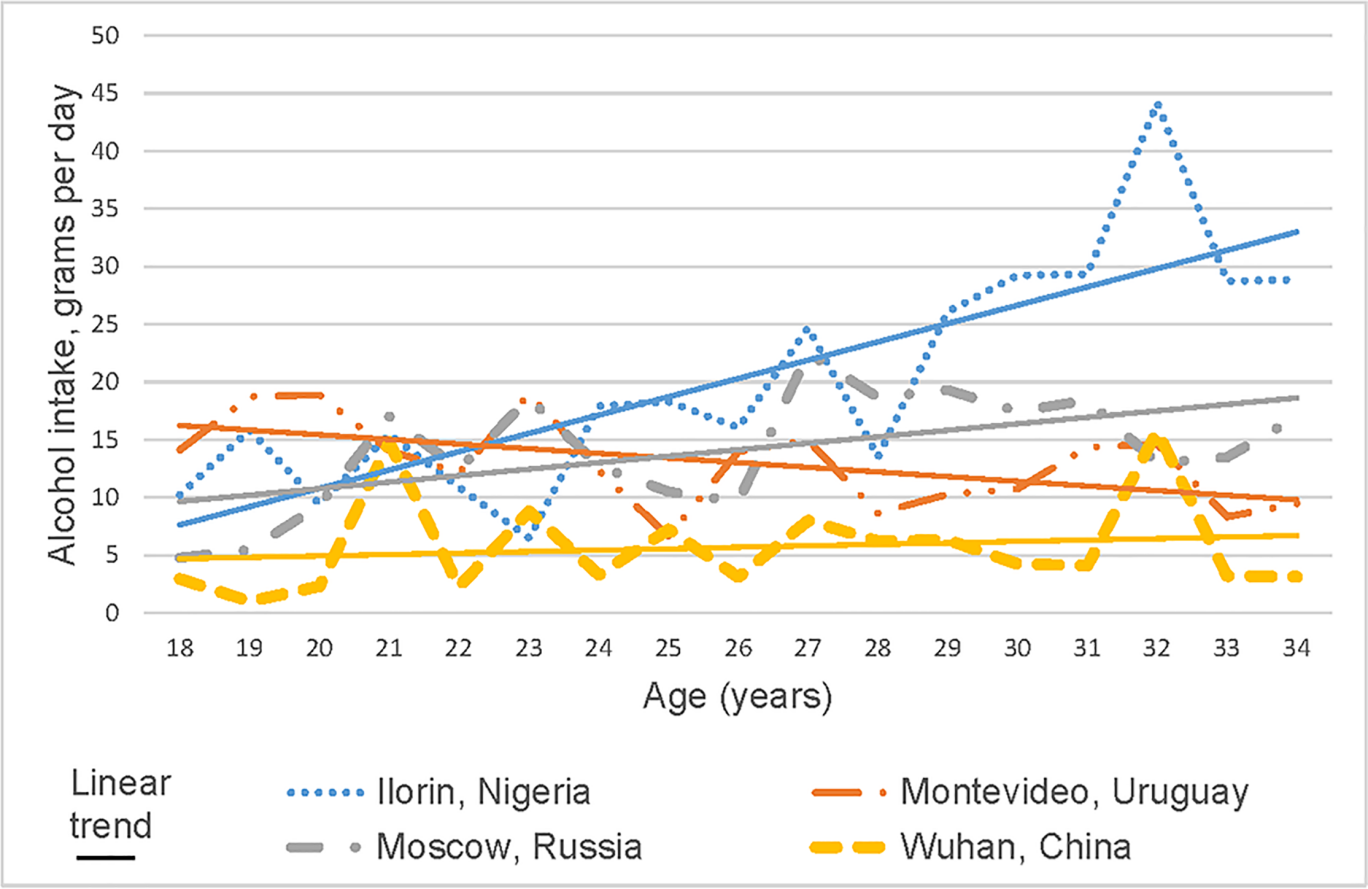
Average alcohol intake, in grams per day, by city.

Table 5 details the BSQF (mean number of drinks consumed in the previous 12 months by type of alcohol (beer, wine, spirits and other)) for three different age groups. ‘Other’ was not asked in Montevideo. Beer drinking increased by age group for respondents in Ilorin and Moscow, while wine and spirits increased by age in Moscow. Overall, beer was consumed more in Ilorin, wine and beer in Montevideo and spirits in Ilorin with age group differences apparent. ‘Other’ alcohol (alcopops) was the highest consumption for Moscow. Fig 1 highlights the average alcohol intake in grams per day for each city with increases apparent by age for Moscow and Ilorin. For current drinkers, average grams per day were 20.8 grams (SD 31.8 median 11.1 grams) for Ilorin, 14.6 grams (SD 33.0 median 4.3 grams) for Moscow, 13 grams (SD 36.1 median 3.9 grams) for Montevideo and 5.9 grams (SD 18.9 median 0.9 grams) for Wuhan.

**Table 5:** Descriptive statistics of Beverage Specific Quantity Frequency (BSQF) (beers, wines and spirits) consumed in the last 12 months by age group and city/country^.

	Ilorin (Nigeria)				Montevideo (Uruguay)				Moscow (Russia)				Wuhan (China)			
	n	Mean number of drinks	SD	Median	n	Mean number of drinks	SD	Median	n	Mean number of drinks	SD	Median	n	Mean number of drinks	SD	Median
**Overall**																
Beer	223	504.4	710.6	312.0	1234	199.3	519.6	60.0	905	212.5	456.4	60.0	553	82.7	244.0	17.0
Wine	146	113.4	201.6	48.0	750	188.9	779.1	25.5	769	68.5	215.2	24.0	359	68.2	307.7	3.5
Spirits	99	200.4	420.9	78.0	781	89.8	204.2	24.0	735	103.8	279.9	30.0	328	151.9	471.6	24.0
Other	132	105.2	439.7	14.0					208	125.3	523.0	12.3	57	73.8	273.8	7.0
**18–24 years**																
Beer	66	288.0	367.9	156.0	521	225.2	585.0	78.0	299	167.4	341.4	60.0	294	64.1	141.1	12.3
Wine	57	96.3	113.8	51.0	312	227.8	919.7	36.0	267	50.5	119.2	17.0	205	84.6	274.6	3.5
Spirits	29	167.5	366.6	78.0	371	113.7	246.3	36.0	209	78.3	189.9	19.3	158	159.9	372.4	12.0
Other	42	117.1	601.1	12.0					90	149.9	536.6	12.0	33	31.9	56.5	10.5
**25–29 years**																
Beer	88	419.9	441.9	312.0	371	173.0	363.2	60.0	326	229.8	462.5	66.0	141	104.1	252.9	24.0
Wine	49	151.6	272.7	60.0	214	160.2	627.0	24.0	260	74.6	249.9	24.0	77	22.1	65.7	3.5
Spirits	33	301.3	537.8	156.0	203	65.8	145.3	14.0	269	106.4	252.7	42.0	86	149.3	412.9	30.0
**Other**	49	126.6	344.2	24.0					77	142.7	543.1	24.0	11	247.0	713.7	7.0
**30–34 years**																
Beer	69	816.4	1120.7	546.0	341	188.3	549.4	48.0	280	240.5	542.7	458.0	119	103.5	459.6	21.0
Wine	40	90.5	168.3	24.0	224	162.0	705.1	24.0	242	81.8	253.8	205.7	77	70.5	402.0	7.0
Spirits	37	137.0	261.6	36.0	208	70.5	165.0	12.0	258	121.7	375.4	320.9	84	139.7	625.3	30.0
Other	41	68.0	270.5	14.0					41	38.9	106.2	92.3	12	27.6	73.2	7.0

SD: Standard deviation. Note: The weighting of data can result in rounding discrepancies or totals not adding. Don’t know/refused not included.

^Excluding those that do not drink.

Table 6 highlights the results of the final fully adjusted models for each city. Current drinkers in Ilorin were more likely to be males, older, divorced or separated and less likely to be Muslim. In Montevideo current drinkers were more likely to be male, with higher levels of education and were less likely to be employed part-time and to cite their employment status as ‘other’. In Moscow current drinkers were more likely to be males, aged 25 to 29 years, higher educated and less likely to be Muslim and cite ‘other’ as their employment status. In Wuhan, current drinkers were more likely to be aged 25 to 29 years, divorced/separated or never married, with a middle level of education.

**Table 6:** Adjusted odds ratios (OR) for current drinkers by city/country^a^.

	Ilorin (Nigeria)		Montevideo (Uruguay)		Moscow (Russia)		Wuhan (China)	
	OR (95% CI)	p value	OR (95% CI)	p value	OR (95% CI)	p value	OR (95% CI)	p value
**Sex**								
Females	1.00		1.00		1.00		1.00	
Males	3.58 (2.42–5.31)	**<0.001**	1.44 (1.02–2.04)	**0.039**	1.42 (1.07–1.88)	**0.016**	0.86 (0.66–1.11)	0.248
**Age group**								
18–24 years	1.00		1.00		1.00		1.00	
25–29 years	2.53 (1.65–3.87)	**<0.001**	1.32 (0.88–1.98)	0.184	1.86 (1.20–2.86)	**0.005**	1.55 (1.05–2.29)	**0.028**
30–34 years	2.62 (1.49–4.63)	**0.001**	0.97 (0.63–1.49)	0.895	1.48 (0.94–2.35)	0.093	1.73 (1.11–2.71)	0.016
**Marital status**								
Married	1.00		1.00		1.00		1.00	
Divorced/ separated / widowed	3.35 (1.26–8.90)	**0.015**	0.78 (0.31–1.97)	0.602	1.44 (0.71–2.90)	0.313	4.07 (1.34–12.33)	**0.013**
Never married	1.24 (0.75–2.05)	0.392	1.33 (0.92–1.92)	0.126	1.25 (0.85–1.83)	0.251	1.48 (1.00–2.19)	**0.049**
**Highest education**								
Secondary school or less	1.00		1.00		1.00		1.00	
Vocational/professional/Non-university tertiary education	0.90 (0.61–1.33)	0.591	1.52 (1.03–2.24)	**0.034**	2.20 (1.52–3.18)	**<0.001**	1.42 (1.08–1.85)	**0.011**
University degree or higher	1.34 (0.85–2.10)	0.206	1.96 (1.05–3.67)	**0.035**	1.72 (1.14–2.61)	**0.01**	1.22 (0.74–2.02)	0.441
**Religion**								
Christian	1.00		1.00		1.00		1.00	
Muslim	0.22 (0.16–0.31)	**<0.001**	-		0.20 (0.11–0.34)	**<0.001**	-	
Agnostic/atheist	^		1.12 (0.81–1.54)	0.505	0.95 (0.64–1.42)	0.809	0.96 (0.35–2.64)	0.942
Other	^		1.08 (0.47–2.48)	0.861	0.59 (0.33–1.04)	0.070	1.22 (0.43–3.47)	0.710
**Employment status**								
Employed full-time	1.00		1.00		1.00		1.00	
Employed part-time	1.87 (1.06–3.29)	**0.031**	0.63 (0.40–0.98)	**0.040**	0.71 (0.44–1.17)	0.179	0.89 (0.42–1.85)	0.746
Unemployed	0.88 (0.53–1.47)	0.636	0.64 (0.40–1.02)	0.061	1.01 (0.32–3.14)	0.992	0.98 (0.48–2.01)	0.951
Student	1.27 (0.78–2.06)	0.331	0.96 (0.55–1.70)	0.900	0.60 (0.07–4.97)	0.636	1.49 (0.97–2.29)	0.066
Other	0.89 (0.36–2.21)	0.794	0.39 (0.16–0.97)	**0.042**	0.30 (0.21–0.42)	**<0.001**	0.82 (0.52–1.29)	0.382

^a^ Adjusted by sex, age, marital status, education, religion and employment status.

^ Insufficient numbers.

OR: Odds Ratio. CI: Confidence Interval

## Discussion

The results of this study show that differences exist between these four cities in terms of alcohol consumption of 18 to 34 year olds. There were major differences in overall alcohol consumption prevalence ranging from a third of Ilorin respondents consuming alcohol to over 95% of those living in Montevideo. Although the proportion of males who consumed alcohol in Ilorin was lower overall, those who did consume alcohol drank on more days (139 days) than the male alcohol consumers in Wuhan (34 days), Uruguay (62 days) and Moscow (71 days). This pattern was repeated for females with overall prevalence of consumption lower in Ilorin but a with a higher mean number of days when alcohol was consumed (96 days) when compared to Moscow (25 days), Wuhan (28 days) and Montevideo (42 days). In terms of differences by gender, overall prevalence rates were similar for males and females except in Ilorin. Of note was the higher overall prevalence of consumption for females in Wuhan. When analysis was undertaken by gender on more specific aspects of alcohol consumption, including mean number of day’s alcohol was consumed and QF, females consistently reported lower consumption patterns for all four cities. Multivariable modelling showed that in three of the four cities higher odds for current drinkers was found for males (Ilorin, Montevideo and Moscow) but not in Wuhan. The modelling highlighted city-specific results with little commonality across the four cities, indicating cultural specific important indicators.

Gender differences are reported in many areas of alcohol research [2,6,11,14,15]. While some studies report male excesses are no longer apparent with an apparent ‘narrowing of the gender gap’ [6,14] other studies highlight that males are still more likely than females to consume alcohol, consume more alcohol, and have increased behavioral problems related to their alcohol consumption [15]. Cultural differences are reported to be major influences in any gender gap [6]. Confirming these assumptions, in our uni-variable analysis, gender gaps in overall alcohol consumption were found only in Ilorin confirming other Nigerian studies [15,16]. In all other cities, the proportion of females who consumed alcohol in the last 12 months was the same as males. Wuhan was the only city where females were more likely than males to have ever consumed alcohol, although they drank on fewer days than males (28 days compared to 34 days). Females were also less likely to be abstainers, moderate and heavier drinkers than males and more likely to be light drinkers (Table 4). Typically in Chinese studies males have higher prevalence rates than females [17]. For the variables that assessed age of first drink and age the respondent first got drunk, very few differences by city or by gender were apparent for all four cities.

Although our overall prevalence estimates show that the gender divide is not obvious (other than in Ilorin) other more detailed results indicate that there are still major gender differences. For the mean number of days alcohol was consumed in the previous 12 months, females were more likely to have consumed alcohol on less days for all four cities. This was also apparent when analysis was undertaken for QF with females notably more likely to be abstainers. This has been reported previously [15] and WHO [1] report that females in all regions of the world are more likely to be abstainers. Females were also less likely to be moderate or heavy drinkers for three of the four cities with Wuhan being the exception. Our final multi-variable model also indicated somewhat different results in terms of gender differences. Once estimates were controlled for by age, sex, marital status, education, religion and employment status, males were over 3.5 times more likely to be current alcohol consumers in Ilorin but also 1.4 times more likely in Montevideo and Moscow. No significant difference was found for Wuhan although of note again is the higher odds ratios for females rather than males.

Research indicates that alcohol consumption generally declines as age increases but this varies by country and culture [4,18]. Although studies, mainly from the USA show that alcohol consumption peaks in early adulthood, our analysis by age showed none or little decrease in alcohol consumption as age increased. For Moscow, Ilorin and Wuhan average alcohol intake, measured by grams per day, actually increased with age (Fig 1). When we assessed the specific wine, beer and spirit consumption by the three age groups, the Moscow older respondents were more likely to report higher consumption of all three types of alcohol compared to younger respondents except for the ‘other’ types of alcohol (alcopops). In Montevideo wine, beer and spirit consumption was higher in the youngest age group although total grams per day consumption only showed a moderate decrease as age increased.

Early age of initiation of alcohol has been highlighted in previous research as a risk factor for later alcohol abuse and dependence [10,19]. Our results indicate differences in age of first drink between the four cities with Montevideo males (mean 15.0 years) and females (mean 15.8 years) starting earlier, and Ilorin males (19.0 years) and females (19.8 years) starting to drink alcohol later. While our study is not longitudinal and no outcome variables were assessed, it is of note that more Montevideo male respondents were heavier drinkers based on the number of drinks per day (9.6%), have less abstainers and more report HED.

In terms of city specific alcohol prevalence rates, alcohol consumption in Moscow has previously been acknowledged as a major public health issue with high prevalence rates across the population [20,21]. In our study Moscow was the only city that showed a steady increase of beer, wine and spirits as age increased although a decrease by age was apparent for alcopops. In terms of Ilorin, Nigeria is a country where, as found in our study, male alcohol consumption excess is still apparent. Ibanga et al [22] also report the importance of gender differences although a study by Adelekan et al [23] reported that there was an increase in consumption of alcohol by high school children with the latest study showing no gender difference among school children. Research reported by Dumbili [24] indicates that females in Nigeria are challenging the cultural norm that alcohol consumption is a male’s domain only. Of interest is the comparison with a previous Nigerian 2001/3 survey [25] where similar prevalence estimates of ever consuming alcohol were reported for males (47% for the Nigerian study compared with 44% for our study). Strikingly the female prevalence rates indicated a large increase in prevalence (12% compared to 24%). Whether this increase is because our study was limited to Ilorin specifically or whether the increase in female prevalence rates reflects studies that suggest Nigerian women are ‘challenging the cultural norm’ cannot be determined. Nevertheless, increase in alcohol consumption in low and middle income countries has been reported [3]. In terms of previous studies undertaken in Wuhan, Zhang et al [26], reporting on a survey of a sample of adults living in Wuhan, also reported higher consumption levels of alcohol and highlighted the cultural importance as alcohol consumption associated with ‘happy and successful events’. Calls for appropriate interventions as Chinese prevalence estimates of alcohol consumption increased has been made [26]. The high rate of alcohol consumption in Montevideo has previously been reported [1] and campaigns aiming to decrease problem alcohol consumption in younger people are now challenging the cultural norm [27].

The limitations associated with this study include the self-reported nature and mode of the data collection with socially desirable responses possible because of the somewhat sensitive nature of the topic, and the fact that the questionnaire was interviewer administered with face-to-face interviews within the home, during which other family members or friends may have been close which may influence responses. Other limitations include the possibility of an underestimate compared with point of sale data. Studies have consistently shown that self-report data collected by surveys, whatever the mode, underestimate consumption when compared against point-of-sale data [28] although our study may provide a more accurate estimate because we included unrecorded alcohol. The cross-sectional nature of the data collection indicates that no cause and effect can be implied. No analysis assessing country-specific policies/guidelines/legislation was undertaken in this analysis. In addition, drinking status was assessed by number of drinks per year but average grams per day could have been used to assess drinking level. Different conclusions might be apparent if this measures was classified differently. Furthermore the data collection was limited to urban populations and the results are not nationally representative.

Notwithstanding, the strengths of the study include the use of a representative sample, and the use of probability-based sampling method used (stratified, clustered, systematic) with selection to ensure random selection of the population thus allowing inferences to be made about the general population. The weighting of the data also allows estimates to be representative of the general population. Further strengths include the relatively large sample size in each city, the range of alcohol-related variables able to be considered, the language specific interviewing, the involvement of local communities and the use of standardized questions (back-translations) to allow cross-cultural comparisons with all four countries. In addition, the specific age groups considered, the high response rates and the data collected on type of alcohol should be considered strengths of the study. The pre-designed methodology employed, rather than post-data manipulation of previously collected data bases, is an important strength of this study.

This analysis has confirmed high alcohol consumption patterns in Ilorin and Montevideo, a higher female overall prevalence rate in Wuhan, and high number of days when alcohol was consumed by those drinking alcohol in Ilorin. The lack of decrease of alcohol consumption in the older age groups we examined could be a result of the delay in transitions to adulthood often cited [8,9]. Further analyses of additional variables included in the data collection on aspects associated with transition to adulthood, but not analyzed as part of this initial research, are planned. This also includes additional analysis assessing aspects associated with negative consequences of inappropriate alcohol consumption. This study has shown that there are some major differences in alcohol consumption and these differences could be are most probably based on different cultural norms. Increased emphasis on decreasing the harmful use of alcohol is warranted. Continual monitoring of this important age group is also a priority so that adequate planning, interventions and appropriate resource allocation can be implemented.

## Supporting information

S1 Questionnaire Questionnaire in Chinese. (DOC)

S2 Questionnaire Questionnaire in English. (DOCX)

S3 Questionnaire Questionnaire in Hausa. (DOC)

S4 Questionnaire Questionnaire in Ibo. (DOC)

S5 Questionnaire Questionnaire in Russian. (DOCX)

S6 Questionnaire Questionnaire in Spanish. (DOCX)

S7 Questionnaire Questionnaire in Yoruba. (DOCX)

S1 Table Additional details on sampling. (DOCX)

S2 Table Response rates by city/country. (DOCX)

S3 Table Demographic characteristics by city/country. (DOCX)
