# Supplementary material for: Context and culture associated with alcohol use amongst youth in major urban cities: A cross-country population based survey
Source: PLoS One. 2017 Nov 20;12(11):e0187812. doi: 10.1371/journal.pone.0187812 (PMC5695777; doi:10.1371/journal.pone.0187812)
Supplement: S1 Questionnaire — (DOC) [file pone.0187812.s001.doc]

**中国城市年轻人饮酒行为与文化背景调查**

**目录**

[黄色标记部分：填写说明； 1](#__RefHeading___Toc397777581)

[红色标记部分：跳转说明； 1](#__RefHeading___Toc397777582)

[绿色标记部分：参考标准杯卡片 1](#__RefHeading___Toc397777583)

[模块A: 家庭确认、知情同意和调查管理问题 （ADMN） 2](#__RefHeading___Toc397777584)

[模块 1: 人口学特征 6](#__RefHeading___Toc397777585)

[模块2: 健康行为，包括饮酒状况 11](#__RefHeading___Toc397777586)

[模块3：过去12个月的饮酒情况 12](#__RefHeading___Toc397777587)

[3.1一般饮酒情况 12](#__RefHeading___Toc397777588)

[3.2 不同酒类的饮酒量和频率 13](#__RefHeading___Toc397777589)

[3.3 醉酒或酒精中毒的经历 15](#__RefHeading___Toc397777590)

[3.4 饮酒情景 16](#__RefHeading___Toc397777591)

[模块4: 饮酒（或反对饮酒）的动机、饮酒影响的认知 – 饮酒者回答 （MTCD） 18](#__RefHeading___Toc397777592)

[模块5: 饮酒（或反对饮酒）的动机、饮酒产生结局的认知 – 曾饮酒者或非饮酒者回答 （MTND） 21](#__RefHeading___Toc397777593)

[模块6: 对酒及饮酒行为的认知（PCPN） 24](#__RefHeading___Toc397777594)

[模块7:青少年和年轻成人的成年期倾向（ADYA） 25](#__RefHeading___Toc397777595)

[模块8: 调查对象参与度、招募和筛选（RCRT） 26](#__RefHeading___Toc397777596)

# 黄色标记部分：填写说明；

# 红色标记部分：跳转说明；

# 绿色标记部分：参考标准杯卡片

#
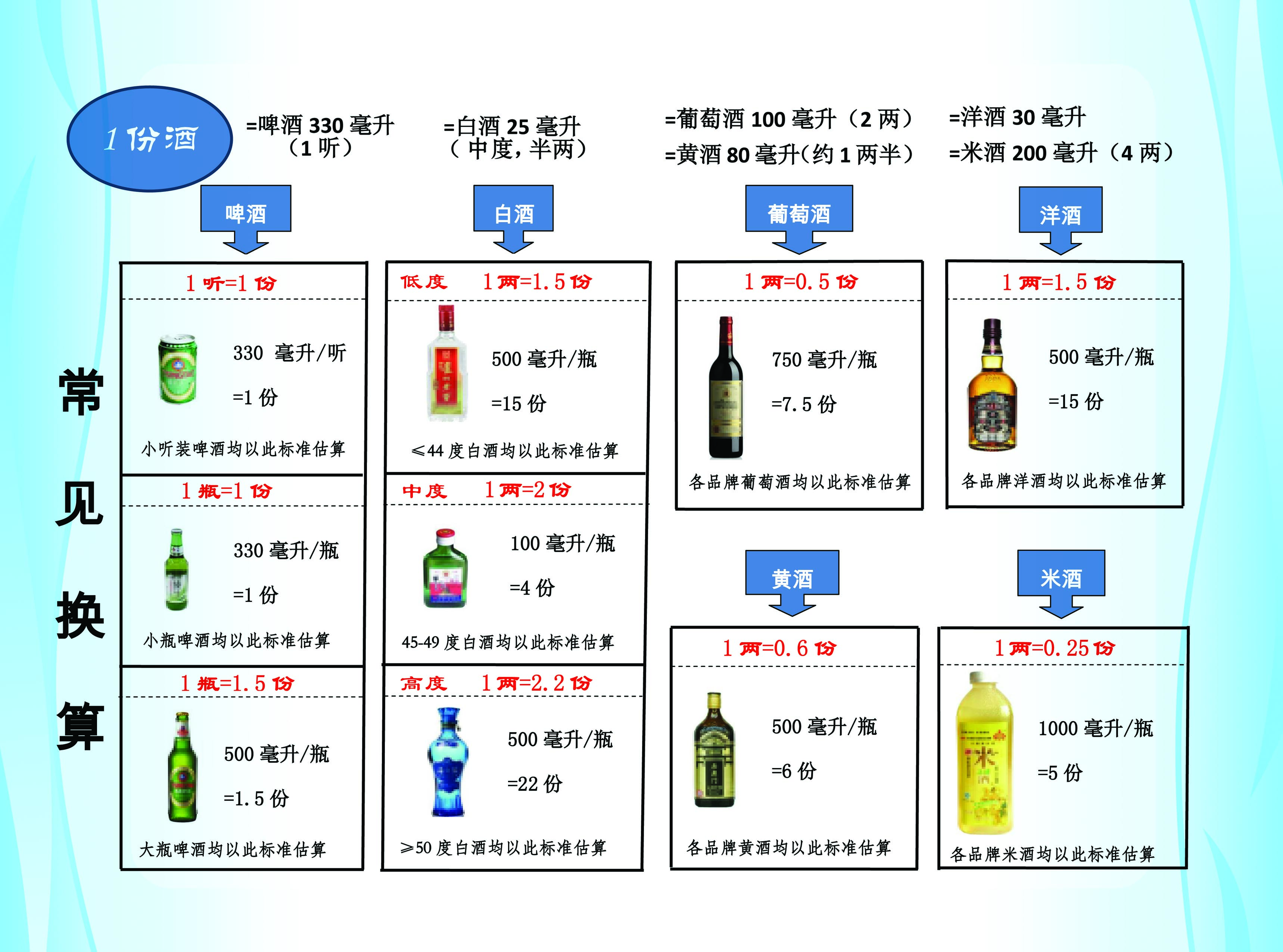


# 模块A: 家庭确认、知情同意和调查管理问题 （ADMN）

***A.0 抽取的调查户确认，调查日期 （调查户抽样完成后，可以在入户调查前完成）***

PARTCIP 调查对象编码（从0001开始）：__ __ __ __（事后编码）

INTID 调查员编码： __ __ __

ADMNLEV… __ 城市： 4 武汉市

ADMNLE0… __ __ 区：1江岸区 2江汉区 3硚口区 4汉阳区 5武昌区 6青山区 7洪山区

8汉南区 9东西湖区 10蔡甸区 11江夏区 12黄陂区 13新洲区

ADMNLE1… __街道：__ __ __ ____ ____ ____ ____ __

（事后编码，根据各区抽取街道数，编1，2，…等.）

ADMNLE2… __ 居民社区（居委会）：__ __ __ ____ ____ ____ ____ __

（事后编码，根据各街道抽取社区数，编1，2，…等.）

ADMNLE3… 调查户地址：____________________________（文字录入）

ADMNDAT 调查日期：__ __ （日）__ __ （月） 2014（年）

***A.1 调查户和调查对象的应答情况 （入户调查后完成）***

ADMNHDI __ __ 调查户应答情况：

调查户合格（包括调查对象应答或不应答）

11 有合格调查对象：调查户中有合格的调查对象（年龄在18-34 岁之间，且在此地居住6个月及以上）

12 调查户拒绝参与：调查户中有合乎要求的调查对象，但调查户户主不同意其成员参与，或者选中的调查对象拒绝参与。（如果调查户户主就是选中的调查对象，则标记HDIS=11，RDIS=22）

13 与调查户有语言交流障碍：调查户中有合乎要求的调查对象，但由于语言沟通问题，调查员不能与调查户户主进行有效的沟通交流。（如果是因为与调查对象的语言沟通障碍，则标记HDIS=11，RDIS=24）

调查户是否合格未知（全部计入无应答）

21 访问调查户三次均未取得联系。

22 与调查户取得联系，但不知是否有住户。

23 与调查户取得联系，且确认是住户，但由于拒绝调查、语言交流障碍或其他问题，不能进一步了解家庭成员状况，完成调查对象选择。

24 由于找不到或不能到达具体地址，或出去调查员安全考虑，没有联系调查户。

25 由于其他方面的原因，没有联系调查户。

调查户不合格（全部计入无应答）

31 不是家庭户单位（如企业、政府、或其他办公/机构）

32 家庭户无人居住

33 季节性、度假或临时居住地

34 调查户成员不符合调查对象要求（年龄在18-34 岁之间，且在此城市居住6个月及以上）

ADMNRDI: __ __ 调查对象应答情况：

应答 11 完成调查

无应答

21 调查期间，调查对象外出；或者访问调查对象三次均未遇见

22 调查对象拒绝参与

23 由于身体或精神方面的原因，调查对象不能参与调查

24 由于语言不通，调查对象不能参与调查

25 调查已经开始，但调查对象提前终止调查

26 调查已经开始，但调查员提前终止调查

ADMNRD0 提前结束调查的原因（如果RDIS = 25或 26）

________________________________________________________________（文字录入）

***A.2调查员入户介绍，收集并选择调查对象***

...INTR 你好，我叫 ，是湖北省疾病预防控制中心的专业调查员。这项调查由国际酒精政策中心支持，在全球4个国家（中国，俄罗斯，尼日利亚和乌拉圭）同时进行，主要是为了了解处于不同文化背景中的城市年轻人对饮酒的认知和饮酒行为状况，以使我们能够更好地理解你们的所思所想所做，为制定文化适宜的酒精政策，减少有害饮酒模式提供依据。

您的地址是被随机抽取的，在正式开始前，我是否能问几个有关家庭成员的问题，以确认合适的对象参加这项调查？

如果**不同意，则结束调查，并回到***回到A.1，标记*调查户应答情况HDIS=23。

... **CENS** 在获得同意后：谢谢您。请您告诉我目前居住在这里的每个家庭成员的性别和年龄？（将年龄填入下表中，并圈出性别。对于年龄在18-34岁的成员，询问其出生日期，并填入表中；同时询问其是否在此城市连续居住了6个月及以上，并在相应栏目中圈出）

家庭成员情况

|  | **年龄** | **出生日期** | **性别** | **调查之前，是否在此城市连续居住6个月及以上** |
| --- | --- | --- | --- | --- |
| **成员 1** | ____ | ________ （年）____（月） ____（日） | __ 男=1/女=2 | __ 是=1 / 否=2 |
| **成员2** | ____ | ________ （年）____（月） ____（日） | __ 男=1 /女=2 | __ 是=1 / 否=2 |
| **成员3** | ____ | ________ （年）____（月） ____（日） | __ 男=1/女=2 | __ 是=1 / 否=2 |
| **成员4** | ____ | ________ （年）____（月） ____（日） | __ 男=1 /女=2 | __ 是=1 / 否=2 |
| **成员5** | ____ | ________ （年）____（月） ____（日） | __ 男=1/女=2 | __ 是=1 / 否=2 |
| **成员6** | ____ | ________ （年）____（月） ____（日） | __ 男=1 /女=2 | __ 是=1 / 否=2 |
| **成员7** | ____ | ________ （年）____（月） ____（日） | __ 男=1/女=2 | __ 是=1 / 否=2 |
| **成员8** | ____ | ________ （年）____（月） ____（日） | __ 男=1 /女=2 | __ 是=1 / 否=2 |
| **成员9** | ____ | ________ （年）____（月） ____（日） | __ 男=1/女=2 | __ 是=1 / 否=2 |
| **成员10** | ____ | ________ （年）____（月） ____（日） | __ 男=1 /女=2 | __ 是=1 / 否=2 |
| **成员11** | ____ | ________ （年）____（月） ____（日） | __ 男=1/女=2 | __ 是=1 / 否=2 |
| **成员12** | ____ | ________ （年）____（月） ____（日） | __ 男=1 /女=2 | __ 是=1 / 否=2 |

**如果该调查户没有符合要求的调查对象（年龄在18-34 岁之间，且在此地居住6个月及以上），则标记调查户应答情况HDIS=34，结束调查。进入下一抽样调查户中。**

**如果该调查户有多名符合要求的调查对象，则询问其出生日期，选择出生日期与调查日期最接近者；如果两个人都接近，选择年轻者。【如果被选中者当时不在家中，三次访问均未见到，则视为失访。请回到A.1，标记HDIS=11，RDIS=21】。**

***A.3对调查对象的介绍语和知情同意***

ADMNINS __ **调查员自我介绍，介绍调查目的并取得调查对象的口头同意。**

你好，我叫 ，是湖北省疾病预防控制中心的专业调查员。这项调查由国际酒精政策中心支持，在全球4个国家（中国，俄罗斯，尼日利亚和乌拉圭）同时进行，主要是为了了解处于不同文化背景中的城市年轻人对饮酒的认知和饮酒行为状况，以使我们能够更好地理解你们的所思所想所做，为制定文化适宜的酒精政策，减少有害饮酒模式提供依据。

您的地址是被随机抽取的，您的参与对我们而言非常重要。完成调查将需要**15-20分钟**。是否参加这项调查，我们完全遵从您的意愿。同时，我们将对您的所有回答进行保密。您也可以直接告诉我们不愿意回答哪个问题。您也可以随时终止调查。

您是否同意参加本次调查呢？

1 – 同意 调查员编码 （表示调查对象口头同意参加调查）

2 – 不同意（如果调查对象拒绝参加调查，则调查终止。请回到A.1，标记HDIS=11，RDIS=22）

***A.4 筛选以及调查管理问题***

ADMN...INST2 谢谢您，在开始调查前，我将问您几个问题，以确认您符合我们调查的年龄和居住时间 要求。.

ADMNBYR 您是哪一年出生的？ _______年

98 – 不知道

99 – 拒绝回答

**如果调查对象的年龄与***A.2部分***家庭成员情况不符，则***回到A.2，进行***调整。**

**如果调查对象不符合年龄要求（18-34岁，即1980年-1996年期间出生）, 则回到A2，选择另外一位符合要求的调查对象（居住时间在6个月及以上，出生日期与调查日期最接近者）进行调查。**

**如果调查对象拒绝回答或不知道年龄，则以A2部分提供的信息为准，继续调查。**

**如果该调查户中没有符合要求的调查对象，请停止调查，并***回到A.1，*标记HDIS=34。

ADMNRES 此前6个月，您是否在此地居住？

1 – 是

2 – 否

98 – 不知道

99 – 拒绝回答

**如果调查对象之前的6个月没有居住此地，则回到A2，选择另外一位符合要求的调查对象（居住时间在6个月及以上，出生日期与调查日期最接近者）进行调查。**

**如果调查对象拒绝回答或不知道居住时间，则以A2部分提供的信息为准，继续调查。**

**如果该调查户中没有符合要求的调查对象，请停止调查，并***回到A.1，*标记HDIS=34。

ADMNLA1 您通常交流的语言是?

1 汉语普通话

2 汉语武汉方言

3 其他（请注明: ）

98 – 不知道

99 – 拒绝回答

ADMNLAN 本次调查所使用的语言是?

1 汉语普通话

2 汉语武汉方言

3 其他（请注明: ）

**【将采用调查对象使用的第一语言进行调查】**

**【如果不能做到或者不知道调查对象使用的第一语言，则回答问题LANG3:标注出调查对象对调查者所使用的语言的理解和接受程度】**

ADMNLA0 **调查对象对调查者所使用的语言的理解和接受程度** *（在1-5之间，其中1 = 调查对象不能听懂，不能完成调查; 5 = 调查对象很容易就理解了问题，流畅地回答）***：***____（本题在调查结束后填写。是否终止调查，根据调查员的判断；如果中止调查，请回到A.1，*标记HDIS=11，RDIS=24*]*

***A.5 调查注意事项***

ADMN…INST3 如果您准备好了，我们现在开始。请记住：对您的任何回答，我们将保守秘密，诚恳希望您能**诚实地回答每个问题**。任何问题，如果您不愿意回答，或者不知道答案，请告诉我。我们可以移到下一个问题。

ADMNSTRT 记录调查开始时间（24小时制）: __ __（ 时）: __ __（分）

# 模块 1: 人口学特征

DEM…SEX 1.1 性别（根据观察记录。必要的时候可询问）

1 – 男性

2 – 女性

98 – 不知道

99 – 拒绝回答

DEM...MAR 1.2 婚姻状况?

1 – 已婚

2 – 离婚或分居

3 – 寡居或鳏居

4 – 从未结婚 （跳至第1.3题）

98 – 不知道（跳至第1.3题）

99 – 拒绝回答（跳至第1.3题）

DEM…MAR_FU 1.2.1 您是哪一年开始处于这种婚姻状况（已婚/离婚或分居/寡居或鳏居）？（如果有多种婚姻状况，以最近的一次为准）?

__ __ __ __ 年

98 – 不知道

99 – 拒绝回答

DEM...HH.AD 1.3 不包括您自己，有几个18岁及以上成人居住在这里（家或者宿舍）？

__ ____ __个18岁及以上成人 *（如果为0,跳至*第1.3.2题*）*

98 – 不知道

99 – 拒绝回答

DEM…HH.AD_FU1 1.3.1 他们是? （多选题）

1 – 配偶或伴侣

2 – 父母或监护人（跳至第1.4题*）*

3 – 其他家庭成员（兄弟姐妹等）

4 – 朋友，室友或其他非亲缘关系的人

98 – 不知道

99 – 拒绝回答

DEM…HH.AD_FU2 1.3.2 您是哪一年不再和父母住在一起？（在题1.3.1中，没有选择“2”的调查对象回答此问题）

__ __ __ __ 年

98 – 不知道

99 – 拒绝回答

DEM...PNT1 1.4 您有孩子吗？（包括自己生育或领养的孩子）

1 – 有

2 – 没有 （跳至 第1.5题）

98 – 不知道 （跳至第1.5题）

99 – 拒绝回答 （跳至第1.5题）

DEM…PNT1_FU 1.4.1 您有几个孩子? ______个孩子

98 – 不知道 （跳至第1.5题）

99 – 拒绝回答 （跳至第1.5题）

*DEM…PNT2 …PNT2_FU 1.4.2 请告诉我，您孩子的具体情况：年龄，是否和您一起居住。*

|  | 孩子的年龄（从最小的孩子开始） | *是否和您住在一起?* |
| --- | --- | --- |
| 第1个孩子 | PNT2_1  岁  98 – 不知道  99 – 拒绝回答 | PNT2_1_FU  1 – 是 2 – 否  98 – 不知道  99 – 拒绝回答 |
| 第2个孩子 | PNT2_2  岁  98 – 不知道  99 – 拒绝回答 | PNT2_2_FU  1 – 是 2 – 否  98 – 不知道  99 – 拒绝回答 |
| 第3个孩子 | PNT2_3  岁  98 – 不知道  99 – 拒绝回答 | PNT2_3_FU  1 – 是 2 – 否  98 – 不知道  99 – 拒绝回答 |
| 第4个孩子 | PNT2_4  岁  98 – 不知道  99 – 拒绝回答 | PNT2_4_FU  1 – 是 2 – 否  98 – 不知道  99 – 拒绝回答 |
| 第5个孩子 | PNT2_5  岁  98 – 不知道  99 – 拒绝回答 | PNT2_5_FU  1 – 是 2 – 否  98 – 不知道  99 – 拒绝回答 |
| 第6个孩子 | PNT2_6  岁  98 – 不知道  99 – 拒绝回答 | PNT2_6_FU  1 – 是 2 – 否  98 – 不知道  99 – 拒绝回答 |
| 第7个孩子 | PNT2_7  岁  98 – 不知道  99 – 拒绝回答 | PNT2_7_FU  1 – 是 2 – 否  98 – 不知道  99 – 拒绝回答 |
| 第8个孩子 | PNT2_8  岁  98 – 不知道  99 – 拒绝回答 | PNT2_8_FU  1 – 是 2 – 否  98 – 不知道  99 – 拒绝回答 |

DEM...EDU 1.5. 您的最高学历是？

1 – 研究生

2 – 大学本科

3 – 大专

4 – 高中/中专/技校

5 – 初中

6 — 小学

7 – 文盲

98 – 不知道

99 – 拒绝回答

DEM…STDT 1.6. 您现在是否是在读学生?

1 – 是 （跳至 第1.7题）*（在读学生包括在职攻读学位者）*

2 – 否

98 – 不知道

99 – 拒绝回答

DEM…STDT_GR 1.6.1 您是哪一年毕业或离开学校的（即使没有取得学位）？

__ __ __ __年

98 – 不知道

99 – 拒绝回答

DEM...EMPL 1.7. 您的就业状况是怎样的？

1 – 全职工作（每周工作时间在40小时及以上，包括个体经营） （跳至第1.7.3题）

2 – 兼职工作（每周工作时间少于40小时，包括个体经营） （跳至第1.7.3题）

3 – 没有就业（包括全职学生以及失业者）

4 – 家庭主妇（夫）

5 – 残疾且不能工作（跳至第1.8题）

6 – 其它情况（请填写）:_____________________________________ （跳至第1.7.4题）

98 – 不知道

99 – 拒绝回答

DEM...EMPL_FU1 1.7.1 您现在正在找工作（指的是要支付报酬的工作）吗？

1 – 是

2 – 否 （跳至第1.7.4题）

98 – 不知道 （跳至第1.7.4题）

99 – 拒绝回答 （跳至第1.7.4题）

DEM...EMPL_FU2 1.7.2 这将是您的第一份工作吗？

1 – 是 （跳至第1.8题）

2 – 否 （跳至第 1.7.4题）

98 – 不知道 （跳至第 1.7.4题）

99 – 拒绝回答 （跳至第1.7.4题）

DEM...EMPL_FU3 1.7.3这是您的第一份工作吗？

1 – 是

2 – 否

98 – 不知道

99 – 拒绝回答

DEM…EMPL_FU4.YR 1.7.4您是哪一年参加工作的（指的是有报酬的工作）? __ __ __ __年

97 – 我从来就没有工作过

98 – 不知道

99 – 拒绝回答

DEM…OCC 1.8 您的职业是？__ __（如果您现在没有工作，但以前参加过工作，请说明您以前的职业。如果您曾经有多个工作，请说明时间最长的那一份工作的时间。如果您正在寻找第一份工作，您想找哪类职业。）

| **1** | 军人（包括公安/交警/消防人员等） |
| --- | --- |
| **2** | 立法者/高级官员/管理者（国家机关和党群组织负责人，事业单位、各类企业单位管理者） |
| **3** | 专业人员  （包含科研人员，工程师，医护及辅助治疗人员，教师，商业及行政管理专业人员如财务、行政管理、销售和市场及公关人员，信息和通信技术人员如软件或程序开发及分析员、数据库和网络专业人员等，法律、社会和文化专业人员如法律工作者、图书管理员、档案管理员、作家、新闻记者等） |
| **4** | 技术员和辅助专业人员  （包含科研人员及工程师助理 如矿业/制造业/建筑业监理、程序控制员、船舶和飞行器控制员及技师，医辅人员，商业辅助专业人员及行政管理秘书，法律、社会和文化专业人员助理包括体育和健身人员，信息和通信技术员，等） |
| **5** | 文职人员  （包括办事员,打字员,顾客服务部人员,数字和材料记录员及其他文员） |
| **6** | 服务业人员/商店或市场销售员  （如导游、厨师、酒店服务员、调酒师、美发美容、建筑物物业管理员等个体化服务人员,商业服务人员,儿童护理员,卫生机构护工保安等） |
| **7** | 熟练的农林牧渔水利生产人员（如种植者/饲养者） |
| **8** | 工艺及相关人员  （包括建筑业相关人员—电工除外，金属和机械业相关人员，手工艺者及印刷工人，电子电气业相关人员，食品生产 、木材加工、服装业及其它相关行业人员） |
| **9** | 种植/机械操作人员/程序员  （包括各类设备及机器操作工，程序员，驾驶员和生产设备操作员） |
| **10** | 初级劳动者  （包括清洁工、护工和保姆，农林牧副渔业劳工，矿工、建筑工人、生产和运输工人，食品加工人员，街头小贩—食品除外, 垃圾工人以及其他初级劳动者） |
| **97** | 我从来就没有工作过 |
| 98 | 不知道 |
| 99 | 拒绝回答 |

…REL 1.9 您的宗教信仰是？

1 – 不可知论者或无神论者

2 – 佛教徒

3 – 基督徒

4 – 印度教教徒

5 – 犹太教徒

6 – 穆斯林

7 – 锡克教教徒

8 – 其他宗教信仰（请注明）: ___________________________________

98 – 不知道

99 – 拒绝回答

…RACE 1.10 您的民族是？

1 – 汉族

2 – 回族

3 – 满族

4 – 蒙古族

5 – 维吾尔族

6 – 其他（请注明）: ___________________________________

98 – 不知道

99 – 拒绝回答

# 模块2: 健康行为，包括饮酒状况

…ALC.EVER 2.1 您是否饮用过含酒精饮料（指啤酒、白酒、葡萄酒、果酒、米酒、马奶酒或其它任何含有酒精的饮料，不包括抿一、二小口酒）？

1 – 是

2 – 否 （跳至模块5的第5.3题）

98 – 不知道

99 – 拒绝回答

…ALC.STRT 2.2 您第一次喝酒是多大年龄（不包括抿一、二小口酒）？ __ __ __ 岁

98 – 不知道

99 – 拒绝回答

…ALC.DRUK 2.3 您第一次喝醉酒是多大年龄？（醉酒是指出现走路不稳、视线模糊、言语不清、恶心呕吐等任何一种现象）? __ __ __ 岁

97 – 我从未喝醉过

98 – 不知道

99 – 拒绝回答

…ALC.HVY.STRT 2.4 我们非常希望了解您喝酒最多的那一段时期的情况。您认为，您是从多大年龄开始，喝酒特别多？ __ __ 岁（如果调查对象回答说，没有喝得最多的那段时期，则以开始喝酒的那一年开始计算）

98 – 不知道

99 – 拒绝回答

…ALC.HVY.STOP 2.5 您又是从哪个年龄开始，不再像那样喝酒了？ __ __ 岁（如果调查对象没有喝得最多的那段时期，且一直喝酒，则填写目前年龄）

97 – 我现在依然是那样喝酒

98 – 不知道

99 – 拒绝回答

# 模块3：过去12个月的饮酒情况

## 3.1一般饮酒情况

…GEN.FQ 3.1.1在过去12个月里，您的饮酒频率是（即使是饮用很少量，也算在内）？

1 – 每天都喝

2 – 每周5-6次

3 – 每周3-4次

4 – 每周1-2次

5 – 每月2-3次

6 – 每月1次

7 – 6 – 11次/过去12个月

8 – 2 – 5次/过去12个月

9 – 1次/过去12个月

10 –在过去12个月里，我没有喝过酒（跳至模块5的第5.1题）

98 – 不知道

99 – 拒绝回答

…GEN.QY 3.1.2 在过去12个月里，当您饮酒时，您通常一天会喝多少杯酒？（此处指的是标准杯，图示）

1 – 25 杯及以上

2 – 19 - 24 杯

3 – 16 - 18 杯

4 – 12 - 15 杯

5 – 9 - 11 杯

6 – 7 - 8 杯

7 – 5 - 6 杯

8 – 3 - 4 杯

9 – 2 杯

10 – 1 杯

11 – 1杯以下 （如果问题3.1.1回答为“9”，并且这个问题选择此项，则跳至模块5的第5.1题）

98 – 不知道

99 – 拒绝回答

…GEN.MST.QY 3.1.3在过去12个月里，您最多的一次喝了多少杯酒？（此处指的是一天24小时内，标准杯，图示）

1 – 36杯及以上

2 – 25 - 35杯

3 – 19 - 24杯

4 – 16 - 18杯

5 – 12 - 15杯

6 – 9 - 11杯

7 – 7 - 8杯

8 – 5 - 6杯

9 – 3 - 4杯

10 –2杯

11 –1杯

12 – 不足一杯

98 – 不知道

99 – 拒绝回答

…GEN.MST.FQ 3.1.4在过去12个月里，类似您在问题3.1.3所说的情况，出现的频率是？

1 – 每天都是这样

2 – 每周5-6次

3 – 每周3-4次

4 – 每周1-2次

5 – 每月2-3次

6 – 每月1次

7 – 6 – 11次/过去12个月

8 – 2 – 5次/过去12个月

9 – 1次/过去12个月

98 – 不知道

99 – 拒绝回答

## 3.2 不同酒类的饮酒量和频率

下面我想问问您，在过去12个月里，不同种类的饮酒情况（如饮酒频率和饮酒量等）。

…BSFQ.BR.FQ 3.2.1在过去12个月里，您饮用啤酒的频率是（即使是饮用很少量，也算在内）?

1 – 每天都喝

2 – 每周5-6次

3 – 每周3-4次

4 – 每周1-2次

5 – 每月2-3次

6 – 每月1次

7 – 6 – 11次/过去12个月

8 – 2 – 5次/过去12个月

9 – 1次/过去12个月

10 –在过去12个月里，我没有喝过啤酒（跳至第3.2.3题）

98 – 不知道

99 – 拒绝回答

…BSFQ.BR.QY 3.2.2 那么，在您喝啤酒时，通常情况下您一天会喝多少杯啤酒？（此处指的是标准杯，图示）

__ __ __ *标准杯*

98 – 不知道

99 – 拒绝回答

…BSFQ.WN.FQ 3.2.3在过去12个月里，您饮用葡萄酒的频率是（即使是饮用很少量，也算在内）?

1 – 每天都喝

2 – 每周5-6次

3 – 每周3-4次

4 – 每周1-2次

5 – 每月2-3次

6 – 每月1次

7 – 6 – 11次/过去12个月

8 – 2 – 5次/过去12个月

9 – 1次/过去12个月

10 –在过去12个月里，我没有喝过葡萄酒（跳至第3.2.5题）

98 – 不知道

99 – 拒绝回答

…BSFQ.WN.QY 3.2.4 那么，在您喝葡萄酒时，通常情况下您一天会喝多少杯葡萄酒？（此处指的是标准杯，图示）

__ __ __ *标准杯*

98 – 不知道

99 – 拒绝回答

…BSFQ.SP.FQ 3.2.5 在过去12个月里，您饮用白酒的频率是（即使是饮用很少量，也算在内）?

1 – 每天都喝

2 – 每周5-6次

3 – 每周3-4次

4 – 每周1-2次

5 – 每月2-3次

6 – 每月1次

7 – 6 – 11次/过去12个月

8 – 2 – 5次/过去12个月

9 – 1次/过去12个月

10 –在过去12个月里，我没有喝过白酒（跳至第3.2.7题）

98 – 不知道

99 – 拒绝回答

…BSFQ.SP.QY 3.2.6 那么，在您喝白酒时，通常情况下您一天会喝多少杯白酒？（此处指的是标准杯，图示）

__ __ __ *标准杯*

98 – 不知道

99 – 拒绝回答

…BSFQ.OTR.FQ 3.2.7在过去12个月里，您饮用黄酒的频率是（即使是饮用很少量，也算在内）?

1 – 每天都喝

2 – 每周5-6次

3 – 每周3-4次

4 – 每周1-2次

5 – 每月2-3次

6 – 每月1次

7 – 6 – 11次/过去12个月

8 – 2 – 5次/过去12个月

9 – 1次/过去12个月

10 –在过去12个月里，我没有喝过黄酒（跳至第3.3.1题）

98 – 不知道

99 – 拒绝回答

…BSFQ.OTR.QY 3.2.8 那么，在您喝黄酒时，通常情况下您一天会喝多少杯黄酒？（此处指的是标准杯，图示）

__ __ __ *标准杯*

98 – 不知道

99 – 拒绝回答

## 3.3 醉酒或酒精中毒的经历

…DRUK.FQ 3.3.1在过去12个月里，您喝醉酒的频率是？（醉酒是指出现走路不稳、视线模糊、言语不清、恶心呕吐等任何一种现象）?

1 – 每天都喝

2 – 每周5-6次

3 – 每周3-4次

4 – 每周1-2次

5 – 每月2-3次

6 – 每月1次

7 – 6 – 11次/过去12个月

8 – 2 – 5次/过去12个月

9 – 1次/过去12个月

10 –在过去12个月里，我从未喝醉过（跳至第3.4.1题）

98 – 不知道

99 – 拒绝回答

…DRUK.NM 3.3.2 通常，您喝多少杯酒会出现上述酒醉的状况？（此处指的是标准杯，图示）

__ __ __ *标准杯*

98 – 不知道

99 – 拒绝回答

## 3.4 饮酒情景

3.4.1 在下列特定场景中，您喝酒的频率是……？（譬如，如果调查对象大约每周一次在餐馆晚餐，且每次都喝酒，则填写答案为“2 至少每周一次”；但如果只是有时喝酒，则填写答案为“3 – 至少每月一次，但少于每周一次”）

|  | 在过去12个月里，在下列场景中，您喝酒的频率是？ | 1 - 每天或几乎每天都喝 | 2 – 至少每周一次 | 3 – 至少每月一次，但少于每周一次 | 4 – 至少每年一次，但少于每月一次 | 5 – 在过去12个月中，从未在此场景中喝过酒 | 98 – 不知道 | 99 – 拒绝回答 |  | 3.4.2 那么，通常每次喝多少酒呢？（此处指的是标准杯，图示）（如果选择5，98，或99，则不回答此题） | | |
| --- | --- | --- | --- | --- | --- | --- | --- | --- | --- | --- | --- | --- |
| （1） | 在餐馆晚餐 |  |  |  |  |  |  |  |  | （1） |  |  |
|  |  |  |  |  |  |  |  |  |  |  | 98 | 99 |
| （2） | 在餐馆午餐 |  |  |  |  |  |  |  |  | （2） |  | |
|  |  |  |  |  |  |  |  |  |  |  | 98 | 99 |
| （3） | 在酒吧、鸡尾酒会 |  |  |  |  |  |  |  |  | （3） |  | |
|  |  |  |  |  |  |  |  |  |  |  | 98 | 99 |
| （4） | 在别人家中（包括参加聚会、派对等） |  |  |  |  |  |  |  |  | （4） |  | |
|  |  |  |  |  |  |  |  |  |  |  | 98 | 99 |
| （5） | 呆在自己家中 |  |  |  |  |  |  |  |  | （5） |  | |
|  |  |  |  |  |  |  |  |  |  |  | 98 | 99 |
| （6） | 朋友来家里时 |  |  |  |  |  |  |  |  | （6） |  | |
|  |  |  |  |  |  |  |  |  |  |  | 98 | 99 |
| （7） | 和朋友一起在公共场所，如公园、街上或者停车场及其它地方？ |  |  |  |  |  |  |  |  | （7） |  | |
|  |  |  |  |  |  |  |  |  |  |  | 98 | 99 |

…CXT.MEAL 3.4.3在过去12个月里，您所喝的酒中，大概有多大比例是伴随着就餐？

1 – 几乎或全部都是

2 – 多于一半以上的时候都是

3 – 一半的时候是

4 – 少于一半的时候是

5 – 几乎或从来都不是

98 – 不知道

99 – 拒绝回答

…CXT.SELF 3.4.4在过去12个月里，您所喝的酒中，大概有多大比例是您独自一个人时喝的？

1 – 几乎或全部都是

2 – 多于一半以上的时候都是

3 – 一半的时候是

4 – 少于一半的时候是

5 – 几乎或从来都不是

98 – 不知道

99 – 拒绝回答

# 模块4: 饮酒（或反对饮酒）的动机、饮酒影响的认知 – 饮酒者回答 （MTCD）

…EFCT 4.1 饮酒对人产生不同方面的影响。此处，我们希望了解饮酒可能会对您产生的影响。当您喝酒时，下列陈述在多大程度上符合您的情况？（非常符合/总是会这样，通常是符合的/通常会是这样，偶尔符合/有时候会这样，很少符合/很少会这样，不符合/从来不会这样）

|  | 当您喝酒时，下列的陈述在多大程度上符合您的情况？ | 1 –总是会这样 | 2 –通常会是这样 | 3 –有时候会这样 | 4 –很少会这样 | 5 –从来不会这样 | 98 – 不知道 | 99 – 拒绝回答 |
| --- | --- | --- | --- | --- | --- | --- | --- | --- |
| （1） | 您会感觉很放松 |  |  |  |  |  |  |  |
| （2） | 您会感觉很高兴 |  |  |  |  |  |  |  |
| （3） | 您会变得咄咄逼人（或者好斗） |  |  |  |  |  |  |  |
| （4） | 您会感觉自己更加友好活泼 |  |  |  |  |  |  |  |
| （5） | 您会发现比较容易讲出自己的感觉或困扰 |  |  |  |  |  |  |  |
| （6） | 您会忘记烦恼 |  |  |  |  |  |  |  |
| （7） | 您会做一些事后后悔的事情 |  |  |  |  |  |  |  |
| （8） | 会让性生活变得更加愉悦 |  |  |  |  |  |  |  |
| （9） | 您会感觉自己更加性感 |  |  |  |  |  |  |  |
| （10） | 您会与警察产生纠纷 |  |  |  |  |  |  |  |
| （11） | 您会获得很多乐趣 |  |  |  |  |  |  |  |
| （12） | 您会感觉身体不舒服 |  |  |  |  |  |  |  |
| （13） | 您会记不起当时发生的情况 |  |  |  |  |  |  |  |

…MOTV.FOR 4.2 人们通常因为不同的原因饮酒。对您个人而言，下列陈述的饮酒理由是否重要？（很重要，重要，不是很重要，一点都不重要）

|  | 对您个人而言，下列陈述的饮酒原因是否重要？ | 1 –很重要 | 2 –重要 | 3 –不是很重要 | 4 –一点都不重要 | 98 – 不知道 | 99 – 拒绝回答 |
| --- | --- | --- | --- | --- | --- | --- | --- |
| （1） | 处于社交或礼貌需要 |  |  |  |  |  |  |
| （2） | 因为其他人都在喝酒 |  |  |  |  |  |  |
| （3） | 为了增加进餐的乐趣 |  |  |  |  |  |  |
| （4） | 因为健康原因 |  |  |  |  |  |  |
| （5） | 让我感觉很好 |  |  |  |  |  |  |
| （6） | 使我放松 |  |  |  |  |  |  |
| （7） | 为了忘记烦恼 |  |  |  |  |  |  |
| （8） | 使我不那么拘谨或害羞 |  |  |  |  |  |  |
| （9） | 为了庆祝 |  |  |  |  |  |  |
| （10） | 因为酒的味道好 |  |  |  |  |  |  |
| （11） | 因为口渴 |  |  |  |  |  |  |

…MOTV.AGST 4.3 人们也会因为不同的原因少饮酒或不饮酒。对您个人而言，下列陈述的这些不喝酒的理由是否重要？（很重要，重要，不是很重要，一点都不重要）

|  |  | 1 –很重要 | 2 –重要 | 3 –不是很重要 | 4 –一点都不重要 | 98 – 不知道 | 99 – 拒绝回答 |
| --- | --- | --- | --- | --- | --- | --- | --- |
| （1） | 因为怀孕或准备怀孕而不喝酒或少喝  （男性调查对象不回答此问题） |  |  |  |  |  |  |
| （2） | 因为酒的口味而不喝酒或少喝 |  |  |  |  |  |  |
| （3） | 我不喜欢受到酒的影响 |  |  |  |  |  |  |
| （4） | 我曾看见一些由饮酒所导致的坏影响 |  |  |  |  |  |  |
| （5） | 我曾被喝酒的人伤害过 |  |  |  |  |  |  |
| （6） | 喝酒可能会影响我的工作或学校表现 |  |  |  |  |  |  |
| （7） | 喝酒太昂贵了或者说是浪费钱（经济原因） |  |  |  |  |  |  |
| （8） | 由于宗教原因而不喝酒或少喝 |  |  |  |  |  |  |
| （9） | 从小就被教育不要喝酒 |  |  |  |  |  |  |
| （10） | 曾因为饮酒陷入麻烦，或者担心自己变成酗酒者 |  |  |  |  |  |  |
| （11） | 因为自己还太年轻，而不喝酒或少喝 |  |  |  |  |  |  |
| （12） | 因为朋友或家人不同意我喝酒 |  |  |  |  |  |  |
| （13） | 因为酒精过敏、正在吃药或由于其他健康方面的原因 |  |  |  |  |  |  |
| （14） | 我只是对喝酒没有兴趣 |  |  |  |  |  |  |

所有回答完这个模块的对象，请跳至模块6。

# 模块5: 饮酒（或反对饮酒）的动机、饮酒产生结局的认知 – 曾饮酒者或非饮酒者回答 （MTND）

EFCT 5.1 饮酒对人产生不同方面的影响。此处，我们希望了解饮酒曾经对您产生的影响。在您喝酒时，下列陈述在多大程度上符合您的情况？（非常符合/总是这样，通常是符合的/通常是这样，偶尔符合/有时候这样，很少符合/很少这样，不符合/从未发生过）【问题5.1针对曾饮酒者（即过去饮酒，但在过去12个月内没有饮酒者），非饮酒者回答问题5.3】

|  | 当您喝酒时，下列陈述在多大程度上符合您的情况？ | 1 –总是这样 | 2 –通常是这样 | 3 –有时候这样 | 4 –很少这样 | 5 –从未出现过 | 98 – 不知道 | 99 – 拒绝回答 |
| --- | --- | --- | --- | --- | --- | --- | --- | --- |
| （1） | 您感觉到很放松 |  |  |  |  |  |  |  |
| （2） | 您感觉到很高兴 |  |  |  |  |  |  |  |
| （3） | 您变得咄咄逼人（或者好斗） |  |  |  |  |  |  |  |
| （4） | 您感觉到自己更加友好活泼 |  |  |  |  |  |  |  |
| （5） | 您发现比较容易讲出自己的感觉或困扰 |  |  |  |  |  |  |  |
| （6） | 您忘记了烦恼 |  |  |  |  |  |  |  |
| （7） | 您做了一些事后后悔的事情 |  |  |  |  |  |  |  |
| （8） | 让性生活变得更加愉悦 |  |  |  |  |  |  |  |
| （9） | 您感觉到自己更加性感 |  |  |  |  |  |  |  |
| （10） | 您与警察产生过纠纷 |  |  |  |  |  |  |  |
| （11） | 您获得了很多乐趣 |  |  |  |  |  |  |  |
| （12） | 您感觉到不舒服 |  |  |  |  |  |  |  |
| （13） | 您记不起当时发生的情况 |  |  |  |  |  |  |  |

MOTV.FOR 5.2 人们通常因为不同的原因饮酒。当您饮酒时，对您个人而言，下列陈述的饮酒理由是否重要？（很重要，重要，不是很重要，一点都不重要）【问题5.2针对曾饮酒者（即过去饮酒，但在过去12个月内没有饮酒者），非饮酒者回答问题5.3】

|  | 当您饮酒时，下列陈述的饮酒原因对您个人而言是否重要？ | 1 –很重要 | 2 –重要 | 3 –不是很重要 | 4 –一点都不重要 | 98 – 不知道 | 99 – 拒绝回答 |
| --- | --- | --- | --- | --- | --- | --- | --- |
| （1） | 处于社交或礼貌需要 |  |  |  |  |  |  |
| （2） | 因为其他人都在喝酒 |  |  |  |  |  |  |
| （3） | 为了增加进餐的乐趣 |  |  |  |  |  |  |
| （4） | 因为健康原因 |  |  |  |  |  |  |
| （5） | 让我感觉很好 |  |  |  |  |  |  |
| （6） | 使我放松 |  |  |  |  |  |  |
| （7） | 为了忘记烦恼 |  |  |  |  |  |  |
| （8） | 使我不那么拘谨或害羞 |  |  |  |  |  |  |
| （9） | 为了庆祝 |  |  |  |  |  |  |
| （10） | 因为酒的味道好 |  |  |  |  |  |  |
| （11） | 因为口渴 |  |  |  |  |  |  |

MOTV.AGST 5.3 人们也会因为不同的原因少饮酒或不饮酒。对您个人而言，下列陈述的这些不喝酒的理由是否重要？（很重要，重要，不是很重要，一点都不重要）【问题5.3针对曾饮酒者和非饮酒者】

|  |  | 1 –很重要 | 2 –重要 | 3 –不是很重要 | 4 –一点都不重要 | 98 – 不知道 | 99 – 拒绝回答 |
| --- | --- | --- | --- | --- | --- | --- | --- |
| （1） | 因为怀孕或准备怀孕而不喝酒或少喝  （男性调查对象不回答此问题） |  |  |  |  |  |  |
| （2） | 因为酒的口味而不喝酒或少喝 |  |  |  |  |  |  |
| （3） | 我不喜欢受到酒的影响 |  |  |  |  |  |  |
| （4） | 我曾看见一些由饮酒所导致的坏影响 |  |  |  |  |  |  |
| （5） | 我曾被喝酒的人伤害过 |  |  |  |  |  |  |
| （6） | 喝酒可能会影响我的工作或学校表现 |  |  |  |  |  |  |
| （7） | 喝酒太昂贵了或者说是浪费钱（经济原因） |  |  |  |  |  |  |
| （8） | 由于宗教原因而不喝酒或少喝 |  |  |  |  |  |  |
| （9） | 从小就被教育不要喝酒 |  |  |  |  |  |  |
| （10） | 曾因为饮酒陷入麻烦，或者担心自己变成酗酒者 |  |  |  |  |  |  |
| （11） | 因为自己还太年轻，而不喝酒或少喝 |  |  |  |  |  |  |
| （12） | 因为朋友或家人不同意我喝酒 |  |  |  |  |  |  |
| （13） | 因为健康方面的原因 |  |  |  |  |  |  |
| （14） | 我只是对喝酒没有兴趣 |  |  |  |  |  |  |

# 模块6: 对酒及饮酒行为的认知（PCPN）

...GEN 6.1 对于下列陈述，您是否同意？（非常同意，同意，无所谓同意或不同意，不同意，坚决不同意）

|  |  | 1 –非常同意 | 2 –同意 | 3 –无所谓同意或不同意 | 4 –不同意 | 5 –坚决不同意 | 98 – 不知道 | 99 – 拒绝回答 |
| --- | --- | --- | --- | --- | --- | --- | --- | --- |
| （1） | 饮酒是生活的乐趣之一 |  |  |  |  |  |  |  |
| （2） | 与别人一同喝酒是显示友善的一种方式 |  |  |  |  |  |  |  |
| （3） | 饮酒没有什么好处可言 |  |  |  |  |  |  |  |

*…SITS 6.2下面列出了一些情景。你觉得在这些情景中，一个人可以喝多少酒，而不至于招致他人反对？（不能喝酒，可以喝点但不会产生什么影响如1-2杯, 有影响到但不要喝醉或只要不喝醉就行，偶尔喝醉没有关系，常常喝醉也无妨? ）*

|  | 你觉得在下列情景中，一个人可以喝多少酒，而不至于招致他人反对？ | 1 –不能喝酒 | 2 –可以喝点但不会产生什么影响（1-2杯） | 3 –只要不喝醉就行 | 4 –偶尔喝醉没有关系 | 5 –即使常常喝醉也无妨 | 98 – 不知道 | 99 – 拒绝回答 |
| --- | --- | --- | --- | --- | --- | --- | --- | --- |
| （1） | 作为母亲，与幼龄子女在一起时 |  |  |  |  |  |  |  |
| （2） | 作为父亲，与幼龄子女在一起时 |  |  |  |  |  |  |  |
| （3） | 作为男性，与朋友一起去酒吧 |  |  |  |  |  |  |  |
| （4） | 作为女性，与朋友一起去酒吧 |  |  |  |  |  |  |  |
| （5） | 作为男性，与同事一起外出 |  |  |  |  |  |  |  |
| （6） | 作为女性，与同事一起外出 |  |  |  |  |  |  |  |
| （7） | 作为男性，与伴侣（配偶和同居者）一起在家中晚餐 |  |  |  |  |  |  |  |
| （8） | 作为女性，与伴侣（配偶和同居者）一起在家中晚餐 |  |  |  |  |  |  |  |

# 模块7:青少年和年轻成人的成年期倾向（ADYA）

*EMAD.PERC 7.1 对于下列陈述，请问您是否同意？（非常同意，同意，无所谓同意或不同意，不同意，坚决不同意）*

|  |  | 1 –非常同意 | 2 –同意 | 3 –无所谓同意或不同意 | 4 –不同意 | 5 –坚决不同意 | 98 – 不知道 | 99 – 拒绝回答 |
| --- | --- | --- | --- | --- | --- | --- | --- | --- |
| （1） | 您已经是成年人了 |  |  |  |  |  |  |  |
| （2） | 您的经济独立（不用父母/监护人/其他家庭成员的资助） |  |  |  |  |  |  |  |
| （3） | 您的情感独立（不需要父母/监护人/其他家庭成员的帮助） |  |  |  |  |  |  |  |

…STOP 记录调查结束时间（24小时制）: __ __ 时: __ __分

# 模块8: 调查对象参与度、招募和筛选（RCRT）

我们马上就要结束调查了。我还有几个小问题想问问您。

ENG1 8.1 用1到10之间的数字来打分，以衡量您对本次调查是否感兴趣，1表示完全不感兴趣，10表示很感兴趣，您会给几分？

__ __分

98 – 不知道

99 – 拒绝回答

ENG2 8.2 同样，用1到10之间的数字来打分，以衡量您是否喜欢参加本次调查，1表示完全不喜欢，10表示很喜欢，您会给几分？

__ __分

98 – 不知道

99 – 拒绝回答

如同我在开始提到的：您的参与对我们而言非常重要，能够帮助我们了解到城市年轻人对饮酒的认知和饮酒行为状况。谢谢您。

另外，我们希望筛选一部分人参加第二阶段的网上调查，为此需要再问您几个问题。当然，不是所有同意参加第二阶段调查的人，都需要完成网络调查，我们会进行再次筛选。

...PART 8.3 您是否有兴趣参加第二阶段的网上调查？

1 – 是，我对此有兴趣（跳至第8.4题）

2 – 否，我对此没有兴趣

98 – 不知道（跳至第8.4题）

99 – 拒绝回答（跳至第8.4题）

…PART.REF 8.3.1能否告诉我，您为什么没有兴趣参加第二阶段的调查？

______________________（开放式回答）（继续询问第8.4题即结束调查）

…SCRN... 8.4 在我们结束调查前，我还有最后几个问题：

…SCRN_STDN 8.4.1 如果我们将学生定义为：目前正在接受某些课程教育或培训的人。如何描述您的目前的状况？

1 – 我是一名全日制学生

2 – 我是一名兼职学生（如业余学生、半工半学等）

3 – 我不是学生（跳至第8.4.3题）

98 – 不知道

99 – 拒绝回答

…SCRN.EDUC 8.4.2 您现在正在完成的学业是？

1 – 高中或中专课程

2 – 继续教育课程（如社区大学、技术学院）

3 – 大学及以上教育课程

4 – 其它（请注明） _______________________________________

98 – 不知道

99 – 拒绝回答

…SCRN.INT 8.4.3 您使用网络的频度是?

1 – 每天都用

2– 每周都用

3 –每月都用

4 – 少于每月都用

5 – 从不使用（指有互联网，但不使用）

6 – 没有互联网络

98 – 不知道

99 – 拒绝回答

…SCRN.CHCK 8.4.4 您查收邮件的频度是?

1 – 每天都查

2– 每周都查

3 –每月都查

4 – 少于每月都查

5 – 从不查收

6 – 我没有电子邮箱

98 – 不知道

99 – 拒绝回答

（如果8.4.3题选择5或6，且8.4.4选择5或6，则调查结束；如果8.4.3题选择1-4，且8.4.4选择5或6，则跳至8.6题；如果8.4.3题选择1-4，且8.4.4选择1-4，则继续。

…PART.EMAIL 8.5 我们将通过电子邮箱告知第二阶段调查的情况，您能否告知电子邮箱？

1 – 可以，电子邮箱地址是:__________________________________（调查员再次确认）

98 – 不知道

99 – 拒绝回答

…PART.CNTC.OTH 8.6 （如果您没有电子邮箱，或者万一通过电子邮箱联系不上您），您能否提供一种其它联系方式，譬如电话号码或通讯地址？

1 – 是，我的电话是：__________________________________（调查员再次确认）

2 – 是，我的通讯地址是：______________________________________________________________________________________________________________________________________（调查员再次确认）

3 – 否，我没有别的联系方式

98 – 不知道

99 – 拒绝回答

[调查结束，谢谢您]
