# Supplementary material for: Context and culture associated with alcohol use amongst youth in major urban cities: A cross-country population based survey
Source: PLoS One. 2017 Nov 20;12(11):e0187812. doi: 10.1371/journal.pone.0187812 (PMC5695777; doi:10.1371/journal.pone.0187812)
Supplement: S2 Questionnaire — (DOCX) [file pone.0187812.s002.docx]

**LTACC Questionnaire**

Final Draft

**Contents**

[Module A: Household Identification and Census, Consent, and Administrative Variables (ADMN) 2](#_Toc394931724)

[MODULE 1: Demographics 5](#_Toc394931725)

[MODULE 9: Health Behaviors, Including Drinker Status (HTBX) 9](#_Toc394931726)

[MODULE 10: Alcohol Consumption, Last 12 Months (CONS) 10](#_Toc394931727)

[10.1 General Alcohol Consumption 10](#_Toc394931728)

[10.2 Beverage-Specific Quantity/Frequency 11](#_Toc394931729)

[10.3 Experience of Drunkenness/Intoxication 13](#_Toc394931730)

[10.4 Drinking Context 13](#_Toc394931731)

[MODULE 15: Motivations For/Against, and Effects of, Drinking – DRINKERS ONLY (MTCD) 15](#_Toc394931732)

[MODULE 16: Motivations For/Against, and Past Outcomes of, Drinking – NON-DRINKERS ONLY (MTND) 18](#_Toc394931733)

[MODULE 17: Own and Others’ Perceptions of Alcohol and Other Substances (PCPN) 21](#_Toc394931734)

[MODULE 20: Adolescents and Young Adults (ADYA) 23](#_Toc394931735)

[20.3 Emerging Adulthood 23](#_Toc394931736)

[MODULE 21: Respondent Engagement, Recruiting and Screening (RCRT) 24](#_Toc394931737)

# **Module A: Household Identification and Census, Consent, and Administrative Variables (ADMN)**

***A.0 Household Identification, Date*** *(Complete before approaching the household/respondent)*

LEV1… City/metropolitan area:

__ __ (see codebook)

LEV2… [country-specific, refer to comparison chart]:

__ __ (see codebook)

LEV3… [country-specific, refer to comparison chart]:

__ __ (see codebook)

LEV4… [country-specific, refer to comparison chart]:

__ __ (see codebook)

…LEV5 [country-specific, refer to comparison chart]:

__ __ (see codebook)

…LEV6 [country-specific, refer to comparison chart]:

__ __ (see codebook)

…DATE __ __ (dd) __ __ (mm) __ __ __ __ (yyyy)

***A.1 Household and Respondent Disposition*** *(Complete after contact with the household/respondent)*

…HDIS Household disposition:

__ __ (see codebook)

…RDIS: Respondent disposition:

__ __ (see codebook)

***A.2 Household Introduction, Census, Determination of Respondent Eligibility***

...INTR Hello, my name is [name]. I am a professional interviewer working with [local organization/institution] as part of a research study funded by the International Center for Alcohol Policies, in Washington, DC, USA. Your address was randomly selected for inclusion in our study on [description of study]. May I ask you a few questions to determine whether anyone living in this household is eligible to participate in the study?

*After obtaining consent:*

Thank you very much. Can you tell me the age and gender of each person currently living in this household?

*Complete Columns 1 and 2 of the matrix below by entering ages and circling the correct gender. For every person listed who is between 18 and 34 years of age, ask whether s/he has lived in [country-specific city/metropolitan area] for at least 6 months and complete Column 3 as appropriate.*

Household Census

|  | **Age** | **Gender** | **Lived in [city/area] for more than 6 months?** |  |  | **Age** | **Gender** | **Lived in [city/area] for more than 6 months?** |
| --- | --- | --- | --- | --- | --- | --- | --- | --- |
| **Person 1** |  | M / F | Y / N |  | **Person 7** |  | M / F | Y / N |
| **Person 2** |  | M / F | Y / N |  | **Person 8** |  | M / F | Y / N |
| **Person 3** |  | M / F | Y / N |  | **Person 9** |  | M / F | Y / N |
| **Person 4** |  | M / F | Y / N |  | **Person 10** |  | M / F | Y / N |
| **Person 5** |  | M / F | Y / N |  | **Person 11** |  | M / F | Y / N |
| **Person 6** |  | M / F | Y / N |  | **Person 12** |  | M / F | Y / N |

*If no eligible respondent, move to next household. If multiple eligible respondents, select respondent using last birthday method and ask to speak to selected respondent. Skip to A.1 and complete the questions below only after* interviewing (or attempting to interview) the selected respondent.

…LANG2 *Language in which interview was conducted:*

[COUNTRY-SPECIFIC OPTIONS]

…LANG3 *Rate the respondent’s apparent comfort with the interview language, on a scale of 1 to 5 (1 = respondent unable to understand, could not finish interview; 5 = respondent fluent, appeared to understand every question with ease): __*

***A.3 Respondent Introduction and Consent***

...INST1 Hello, my name is [name]. I am a professional interviewer working with [local organization/institution] as part of a research study funded by the International Center for Alcohol Policies, in Washington, DC, USA. Your address was randomly selected for inclusion in our study on [description of study]. It is crucial to the success of this study that we are able to collect information from people with a variety of attitudes, views, backgrounds, and experiences, including people like yourself. Participation in this study involves answering questions about [your health, your attitudes, and your behaviors, including alcohol consumption and drug use]. The survey takes between [time range] to complete. Your decision whether or not to participate in this research is completely voluntary. Should you decide to participate, your responses will be kept completely confidential. If any questions make you uncomfortable, we can skip them. You are also free to end the interview at any time. Will you agree to participate in our study?

________

Initials of interviewer, indicating

verbal consent of respondent

***A.4 Screening and Administrative Questions***

...INST2 Thank you. Before we begin, I would just like to ask you a few questions to make sure you are eligible to participate in this study.

...BYR In what year were you born?

__ __ __ __

98 – DON’T KNOW

99 – REFUSED

*Probe if, based on birth year, respondent may be younger than 18 (born in 1996) or older than 34 (born in 1980). Interview ends if respondent is not in desired age range, or age cannot be determined. [Code respondent as “Ineligible”.]*

…RESI And have you lived in [city/metropolitan area] for the past 6 months?

1 – YES

2 – NO

98 – DON’T KNOW

99 – REFUSED

*Interview ends if respondent has not resided in city/metropolitan area for at least the previous 6 months. [Code respondent as “Ineligible”.]*

…LANG1 What is your primary language?

[COUNTRY-SPECIFIC RESPONSE OPTIONS]

+

98 – DON’T KNOW

99 – REFUSED

*Interview is conducted in respondent’s primary language, if possible. If this is not possible, or the respondent’s primary language is not known, rate (on previous page) the respondent’s level of comfort with the language in which the interview was conducted upon the conclusion of the interview.*

***A.5 Introduction to Interview***

…INST3 If it is okay with you, I will now begin the interview. Remember, your responses will be kept in the strictest of confidence, **so please answer each question honestly**. If any question makes you uncomfortable, or you do not know the answer, please let me know and we can move on to the next question.

…STRT  *Record current time:*

__ __ : __ __ (HH:MM, 24:00 clock)

# MODULE 1: Demographics

…SEX *(Record gender from observation. Ask if necessary.)*

1 – MALE

2 – FEMALE

98 – DON’T KNOW

99 – REFUSED

...MAR What best describes your marital status?

1 – MARRIED

2 – DIVORCED OR SEPARATED

3 – WIDOWED

4 – NEVER MARRIED (Skip to …HH.AD)

98 – DON’T KNOW (Skip to …HH.AD)

99 – REFUSED (Skip to …HH.AD)

…MAR_FU What year were you (married/divorced or separated/widowed)?

__ __ __ __

98 – DON’T KNOW

99 – REFUSED

...HH.AD Not including yourself, how many adults 18 years or older live in this household?

__ __ ADULTS *(If 0, skip to DEM.HH.AD_FU2)*

98 – DON’T KNOW

99 – REFUSED

…HH.AD_FU1 Who are they? (Select all that apply)

1 – SPOUSE OR PARTNER

2 – PARENT OR GUARDIAN (*Skip to DEM.HH.PNT1)*

3 – OTHER FAMILY MEMBER

4 – FRIEND, ROOMMATE, OR

OTHER NON-RELATIVE

98 – DON’T KNOW

99 – REFUSED

…HH.AD_FU2 What year did you move out of your family home and start living independently?

__ __ __ __

98 – DON’T KNOW

99 – REFUSED

...PNT1 Do you have any children?

1 – YES

2 – NO *(Skip to DEM.EDU)*

98 – DON’T KNOW *(Skip to DEM.EDU)*

99 – REFUSED *(Skip to DEM.EDU)*

…PNT1_FU How many children do you have?

______ Children

98 – DON’T KNOW *(Skip to DEM.EDU)*

99 – REFUSED *(Skip to DEM.EDU)*

*…PNT2 …PNT2_FU*

| Child # | What are their ages? (starting with the youngest) | Does he or she live with you? |
| --- | --- | --- |
| 1 | PNT2_1  YEARS  98 – DON’T KNOW  99 – REFUSED | PNT2_1_FU  1 – YES 2 – NO  98 – DON’T KNO*W*  99 – REFUSED |
| 2 | PNT2_2  YEARS  98 – DON’T KNOW  99 – REFUSED | PNT2_2_FU  1 – YES 2 – NO  98 – DON’T KNO*W*  99 – REFUSED |
| 3 | PNT2_3  YEARS  98 – DON’T KNOW  99 – REFUSED | PNT2_3_FU  1 – YES 2 – NO  98 – DON’T KNO*W*  99 – REFUSED |
| 4 | PNT2_4  YEARS  98 – DON’T KNOW  99 – REFUSED | PNT2_4_FU  1 – YES 2 – NO  98 – DON’T KNO*W*  99 – REFUSED |
| 5 | PNT2_5  YEARS  98 – DON’T KNOW  99 – REFUSED | PNT2_5_FU  1 – YES 2 – NO  98 – DON’T KNO*W*  99 – REFUSED |
| 6 | PNT2_6  YEARS  98 – DON’T KNOW  99 – REFUSED | PNT2_6_FU  1 – YES 2 – NO  98 – DON’T KNO*W*  99 – REFUSED |
| 7 | PNT2_7  YEARS  98 – DON’T KNOW  99 – REFUSED | PNT2_7_FU  1 – YES 2 – NO  98 – DON’T KNO*W*  99 – REFUSED |
| 8 | PNT2_8  YEARS  98 – DON’T KNOW  99 – REFUSED | PNT2_8_FU  1 – YES 2 – NO  98 – DON’T KNO*W*  99 – REFUSED |

...EDU What is the highest level of education you have completed?

[COUNTRY-SPECIFIC RESPONSE OPTIONS]

+

98 – DON’T KNOW

99 – REFUSED

…STDT Are you currently a student?

1 – YES *(Skip to DEM.EMPL)*

2 – NO

98 – DON’T KNOW

99 – REFUSED

…STDT_GR What year did you graduate or leave school?

__ __ __ __

98 – DON’T KNOW

99 – REFUSED

...EMPL How would you describe your employment situation?

1 – EMPLOYED FULL-TIME (40 hours or more per week, including self-employment) *(Skip to EMPL_FU3)*

2 – EMPLOYED PART-TIME (fewer than 40 hours per week, including self-employment) *(Skip to EMPL_FU3)*

3 – UNEMPLOYED

4 – HOMEMAKER

5 – DISABLED AND UNABLE TO WORK *(Skip to DEM.OCC)*

6 – OTHER: _______________________________________________ *(Skip to EMPL_FU1)*

98 – DON’T KNOW

99 – REFUSED

...EMPL_FU1 Are you currently looking for paid employment?

1 – YES

2 – NO *(Skip to EMPL_FU4.YR)*

98 – DON’T KNOW *(Skip to EMPL_FU4.YR)*

99 – REFUSED *(Skip to EMPL_FU4.YR)*

...EMPL_FU2 Would this be your first paid job?

1 – YES *(Skip to DEM.REL)*

2 – NO *(Skip to EMPL_FU4)*

98 – DON’T KNOW *(Skip to EMPL_FU4.YR)*

99 – REFUSED *(Skip to EMPL_FU4.YR)*

...EMPL_FU3 Is this your first paid job?

1 – YES

2 – NO

98 – DON’T KNOW

99 – REFUSED

…EMPL_FU4 What year did you first start working for pay?

__ __ __ __

97 – I HAVE NEVER WORKED FOR PAY

98 – DON’T KNOW

99 – REFUSED

…OCC What is your occupation or profession? If you are not presently working but worked in the past, please indicate which occupation you were employed in. If you have worked in multiple occupations, please select the one in which you have spent the most time.

| **1** | ARMED FORCES |
| --- | --- |
| **2** | LEGISLATORS/SENIOR OFFICIALS/MANAGERS |
| **3** | PROFESSIONALS |
| **4** | TECHNICIANS AND ASSOCIATE PROFESSIONALS |
| **5** | CLERKS |
| **6** | SERVICE WORKERS/SHOP/MARKET SALES WORKERS |
| **7** | SKILLED AGRICULTURAL/FISH WORKERS |
| **8** | CRAFT AND RELATED WORKERS |
| **9** | PLANT/MACHINE OPERATORS/ASSEMBLERS |
| **10** | ELEMENTARY OCCUPATIONS |
| **97** | I HAVE NEVER BEEN EMPLOYED |
| **98** | DON’T KNOW |
| **99** | REFUSED |

…REL What is your religious affiliation?

1 – AGNOSTIC/ATHEIST

2 – BUDDHIST

3 – CHRISTIAN

4 – HINDU

5 – JEWISH

6 – MUSLIM

7 – SIKH

8 – ANOTHER RELIGION: ___________________________________

98 – DON’T KNOW

99 – REFUSED

…RACE What is your race/ethnicity?

[COUNTRY-SPECIFIC RESPONSE OPTIONS]

+

98 – DON’T KNOW

99 – REFUSED

# MODULE 9: Health Behaviors, Including Drinker Status (HTBX)

…ALC.EVER Have you ever consumed a drink that contains alcohol, such as [country-specific examples], etc.? Please do not include times when you only had a sip or two from someone else’s drink.

1 – YES

2 – NO *(Skip to MTND.MOTV.AGST)*

98 – DON’T KNOW

99 – REFUSED

…ALC.STRT How old were you the first time you had a drink of an alcoholic beverage? Please do not include any time when you only had a sip or two from a drink.

__ __ YEARS OLD

98 – DON’T KNOW

99 – REFUSED

…ALC.DRUK How old were you the first time you got drunk?

__ __ YEARS OLD

97 – I HAVE NEVER GOTTEN DRUNK

98 – DON’T KNOW

99 – REFUSED

…ALC.HVY.STRT We are interested in your ages at the beginning and end of the period in your life when you were drinking most heavily. About what age would you say you were when the period of heaviest drinking began?

__ __ YEARS OLD

98 – DON’T KNOW

99 – REFUSED

…ALC.HVY.STOP And about how old were you when it ended?

__ __ YEARS OLD

97 – IT HAS NOT ENDED

98 – DON’T KNOW

99 – REFUSED

# MODULE 10: Alcohol Consumption, Last 12 Months (CONS)

## 10.1 General Alcohol Consumption

…GEN.FQ During the past 12 months, how often did you drink beer, wine, spirits (e.g., vodka, gin, whisky, brandy), or any other alcoholic beverage, even in small amounts? *(Show card)*

1 – Every day

2 – 5 to 6 times a week

3 – 3 to 4 times a week

4 – 1 TO 2 TIMES A WEEK

5 – 2 to 3 times a month

6 – Once a month

7 – 6 to 11 times in the past 12 months

8 – 2 to 5 times in the past 12 months

9 – ONCE IN THE PAST 12 MONTHS

10 – I DID NOT DRINK ANY ALCOHOL IN THE PAST 12 MONTHS *(Skip to MTND.EFCT)*

98 – DON’T KNOW

99 – REFUSED

…GEN.QY During the past 12 months, how many alcoholic drinks did you have on a typical day when you drank alcohol? By one drink we mean [country-specific definition of a standard drink]. *(Show card and graphic illustration)*

1 – 25 drinks or more

2 – 19 to 24 drinks

3 – 16 to 18 drinks

4 – 12 to 15 drinks

5 – 9 to 11 drinks

6 – 7 to 8 drinks

7 – 5 to 6 drinks

8 – 3 to 4 drinks

9 – 2 drinks

10 – 1 drink

11 – LESS THAN 1 FULL DRINK *(If* ***both*** *CONS.GEN.FQ = 9 and GEN.QY=11, skip to MTND.EFCT)*

98 – DON’T KNOW

99 – REFUSED

…GEN.MST.QY During the past 12 months, what is the largest number of drinks containing alcohol that you drank within a 24-hour period? *(Show card)*

1 – 36 drinks or more

2 – 25 to 35 drinks

3 – 19 to 24 drinks

4 – 16 to 18 drinks

5 – 12 to 15 drinks

6 – 9 to 11 drinks

7 – 7 to 8 drinks

8 – 5 to 6 drinks

9 – 3 to 4 drinks

10 – 2 drinks

11 – 1 drink

12 – LESS THAN 1 FULL DRINK

98 – DON’T KNOW

99 – REFUSED

…GEN.MST.FQ During the past 12 months, how often did you drink [answer from CONS.GEN.MST.QY] within a 24-hour period? *(Show card)*

1 – Every day

2 – 5 to 6 times a week

3 – 3 to 4 times a week

4 – 1 TO 2 TIMES A WEEK

5 – 2 to 3 times a month

6 – Once a month

7 – 6 to 11 times in the past 12 months

8 – 2 to 5 times in the past 12 months

9 – ONCE IN THE PAST 12 MONTHS

98 – DON’T KNOW

99 – REFUSED

## 10.2 Beverage-Specific Quantity/Frequency

Now I would like to ask you how often you drank particular alcoholic beverages over the past 12 months, and how much you usually drank of each beverage on an average day when you drank.

…BSFQ.BR.FQ How often did you drink **beer** over the past 12 months? *(Show card)*

1 – Every day

2 – 5 to 6 times a week

3 – 3 to 4 times a week

4 – 1 TO 2 TIMES A WEEK

5 – 2 to 3 times a month

6 – Once a month

7 – 6 to 11 times in the past 12 months

8 – 2 to 5 times in the past 12 months

9 – ONCE IN THE PAST 12 MONTHS

10 – NEVER IN THE PAST 12 MONTHS *(Skip to CONS.BSFQ.WN.FQ)*

98 – DON’T KNOW

99 – REFUSED

…BSFQ.BR.QY And on a typical day when you drank beer, how much beer did you drink?

__ __ *(Show card for standard drink)*

98 – DON’T KNOW

99 – REFUSED

…BSFQ.WN.FQ How often did you drink **wine** over the past 12 months? *(Show card)*

1 – Every day

2 – 5 to 6 times a week

3 – 3 to 4 times a week

4 – 1 TO 2 TIMES A WEEK

5 – 2 to 3 times a month

6 – Once a month

7 – 6 to 11 times in the past 12 months

8 – 2 to 5 times in the past 12 months

9 – ONCE IN THE PAST 12 MONTHS

10 – NEVER IN THE PAST 12 MONTHS *(Skip to BSFQ.SP.FQ)*

98 – DON’T KNOW

99 – REFUSED

…BSFQ.WN.QY And on a typical day when you drank wine, how much wine did you drink?

__ __ *(Show card for standard drink)*

98 – DON’T KNOW

99 – REFUSED

…BSFQ.SP.FQ How often did you drink **spirits** over the past 12 months? *(Show card)*

1 – Every day

2 – 5 to 6 times a week

3 – 3 to 4 times a week

4 – 1 TO 2 TIMES A WEEK

5 – 2 to 3 times a month

6 – Once a month

7 – 6 to 11 times in the past 12 months

8 – 2 to 5 times in the past 12 months

9 – ONCE IN THE PAST 12 MONTHS

10 – NEVER IN THE PAST 12 MONTHS *(Skip to CONS.BSFQ.OTR.FQ)*

98 – DON’T KNOW

99 – REFUSED

…BSFQ.SP.QY And on a typical day when you drank spirits, how much spirits did you drink?

__ __ *(Show card for standard drink)*

98 – DON’T KNOW

99 – REFUSED

…BSFQ.OTR.FQ How often did you drink **[country-specific beverage]** over the past 12 months? *(Show card)*

1 – Every day

2 – 5 to 6 times a week

3 – 3 to 4 times a week

4 – 1 TO 2 TIMES A WEEK

5 – 2 to 3 times a month

6 – Once a month

7 – 6 to 11 times in the past 12 months

8 – 2 to 5 times in the past 12 months

9 – ONCE IN THE PAST 12 MONTHS

10 – NEVER IN THE PAST 12 MONTHS *(Skip to DRUK.FQ)*

98 – DON’T KNOW

99 – REFUSED

…BSFQ.OTR.QY And on a typical day when you drank [country-specific beverage], how much [country-specific beverage did you drink?

__ __ *(Show card for standard drink)*

98 – DON’T KNOW

99 – REFUSED

## 10.3 Experience of Drunkenness/Intoxication

…DRUK.FQ How often in the past 12 months did you drink enough alcohol to feel intoxicated or drunk – either you felt unsteady on your feet, or your vision was blurred, or your speech was slurred? *(Show card)*

1 – Every day

2 – 5 to 6 times a week

3 – 3 to 4 times a week

4 – 1 TO 2 TIMES A WEEK

5 – 2 to 3 times a month

6 – Once a month

7 – 6 to 11 times in the past 12 months

8 – 2 to 5 times in the past 12 months

9 – ONCE IN THE PAST 12 MONTHS

10 – NEVER IN THE PAST 12 MONTHS *(Skip to CONS.CXT1)*

98 – DON’T KNOW

99 – REFUSED

…DRUK.NM How many drinks does it usually take for you feel intoxicated or drunk? (1 drink is *(Show card for standard drink)*.)

__ __ DRINKS

98 – DON’T KNOW

99 – REFUSED

## 10.4 Drinking Context

I am now going to ask you how often you drink alcohol in specific situations.

|  | During the past 12 months, how often did you drink alcohol when… | 1- EVERY DAY OR NEARLY EVERYDAY | 2 - AT LEAST ONCE A WEEK | 3 - AT LEAST ONCE A MONTH BUT LESS THAN ONCE A WEEK | 4 - AT LEAST ONCE IN THE PAST 12 MONTHS BUT LESS THAN ONCE A MONTH | 5 - NEVER DURING THE PAST 12 MONTHS | 98 – DON’T KNOW | 99 - REFUSED |  | And how many drinks did you usually have? *(Skip if preceding answer is 5, 98, or 99)* | | |
| --- | --- | --- | --- | --- | --- | --- | --- | --- | --- | --- | --- | --- |
| …CXT1 | Out for an evening meal at a restaurant? |  |  |  |  |  |  |  |  | 1.NM |  | |
|  |  |  |  |  |  |  |  |  |  |  | 98 | 99 |
| …CXT2 | Out for lunch at a restaurant? |  |  |  |  |  |  |  |  | 2.NM |  | |
|  |  |  |  |  |  |  |  |  |  |  | 98 | 99 |
| …CXT3 | At bars, taverns, or cocktail lounges? |  |  |  |  |  |  |  |  | 3.NM |  | |
|  |  |  |  |  |  |  |  |  |  |  | 98 | 99 |
| …CXT4 | Spending time in someone else’s home, including parties? |  |  |  |  |  |  |  |  | 4.NM |  | |
|  |  |  |  |  |  |  |  |  |  |  | 98 | 99 |
| …CXT5 | Spending a quiet evening at home? |  |  |  |  |  |  |  |  | 5.NM |  | |
|  |  |  |  |  |  |  |  |  |  |  | 98 | 99 |
| …CXT6 | Friends visited you in your home? |  |  |  |  |  |  |  |  | 6.NM |  | |
|  |  |  |  |  |  |  |  |  |  |  | 98 | 99 |
| …CXT7 | Spending time with friends in a public place, such as a park, street, or parking lot? |  |  |  |  |  |  |  |  | 7.NM |  | |
|  |  |  |  |  |  |  |  |  |  |  | 98 | 99 |

…CXT.MEAL During the past 12 months, approximately how much of your drinking occurred during meals? Was it…

1 – All or almost all?

2 – More than half?

3 – Half?

4 – Less than half?

5 – None or almost none?

98 – DON’T KNOW

99 – REFUSED

…CXT.SELF During the past 12 months, about how much of your drinking occurred when you were by yourself? Would you say…

1 – All or almost all?

2 – More than half?

3 – Half?

4 – Less than half?

5 – None or almost none?

98 – DON’T KNOW

99 – REFUSED

# MODULE 15: Motivations For/Against, and Effects of, Drinking – DRINKERS ONLY (MTCD)

…EFCT Drinking alcohol affects people in many different ways. We would like to learn what effects drinking may have for you. When you drink alcohol, how true would you say each of these statements is for you – very often true, often true, sometimes true, rarely true, or never true?

|  | How true is it that when you drink alcohol… | 1 – V ERY OFTEN TRUE | 2 – OFTEN TRUE | 3 – SOMETIMES TRUE | 4 – RARELY TRUE | 5 – NEVER TRUE | 98 – DON’T KNOW | 99 – REFUSED |
| --- | --- | --- | --- | --- | --- | --- | --- | --- |
| _1 | You feel relaxed? |  |  |  |  |  |  |  |
| _2 | You feel happy? |  |  |  |  |  |  |  |
| _3 | You become more aggressive toward other people? |  |  |  |  |  |  |  |
| _4 | You feel more friendly and outgoing? |  |  |  |  |  |  |  |
| _5 | You find it easier to talk about your feelings or problems? |  |  |  |  |  |  |  |
| _6 | You forget your problems? |  |  |  |  |  |  |  |
| _7 | You do something you later regret? |  |  |  |  |  |  |  |
| _8 | Sexual activity is more pleasurable for you? |  |  |  |  |  |  |  |
| _9 | You feel more sexually attractive? |  |  |  |  |  |  |  |
| _10 | You get into trouble with police? |  |  |  |  |  |  |  |
| _11 | You have a lot of fun? |  |  |  |  |  |  |  |
| _12 | You feel sick? |  |  |  |  |  |  |  |
| _13 | You don’t remember what happened (you black out)? |  |  |  |  |  |  |  |

…MOTV.FOR People have different reasons for drinking alcohol. How important would you say each of the following reasons is for you, personally? Would you say very important, important, not very important, or not at all important?

|  |  | 1 – V ERY IMPORTANT | 2 – IMPORTANT | 3 – NOT VERY IMPORTANT | 4 – NOT AT ALL IMPORTANT | 98 – DON’T KNOW | 99 – REFUSED |
| --- | --- | --- | --- | --- | --- | --- | --- |
| _1 | To be sociable or polite? |  |  |  |  |  |  |
| _2 | Because others are drinking? |  |  |  |  |  |  |
| _3 | To add to the enjoyment of meals? |  |  |  |  |  |  |
| _4 | For health reasons? |  |  |  |  |  |  |
| _5 | To feel good? |  |  |  |  |  |  |
| _6 | To help you relax? |  |  |  |  |  |  |
| _7 | To forget worries? |  |  |  |  |  |  |
| _8 | To help you feel less inhibited or shy? |  |  |  |  |  |  |
| _9 | To celebrate? |  |  |  |  |  |  |
| _10 | Because of the taste? |  |  |  |  |  |  |
| _11 | Because of thirst? |  |  |  |  |  |  |

…MOTV.AGST People also have different reasons for limiting their drinking, or not drinking alcohol at all. How important would you say each of the following reasons is for you, personally? Would you say very important, important, not very important, or not at all important?

|  |  | 1 – V ERY IMPORTANT | 2 –IMPORTANT | 3 – NOT VERY IMPORTANT | 4 – NOT AT ALL IMPORTANT | 98 – DON’T KNOW | 99 – REFUSED |
| --- | --- | --- | --- | --- | --- | --- | --- |
| _1 | [Skip if respondent is male]  Because you were or are pregnant, or trying to become pregnant? |  |  |  |  |  |  |
| _2 | Because of the taste? |  |  |  |  |  |  |
| _3 | Because you don’t like the effect it has on you? |  |  |  |  |  |  |
| _4 | Because you have seen bad examples of what alcohol can do? |  |  |  |  |  |  |
| _5 | Because you have been hurt by someone else’s drinking? |  |  |  |  |  |  |
| _6 | Because drinking could affect your work or school performance? |  |  |  |  |  |  |
| _7 | Because drinking is too expensive or is a waste of money? |  |  |  |  |  |  |
| _8 | Because of religious reasons? |  |  |  |  |  |  |
| _9 | Because you were brought up not to drink? |  |  |  |  |  |  |
| _10 | Because you have had alcohol problems or are afraid of becoming an alcoholic? |  |  |  |  |  |  |
| _11 | Because you are too young? |  |  |  |  |  |  |
| _12 | Because your friends and/or family members disapprove of you drinking? |  |  |  |  |  |  |
| _14 | For health reasons? |  |  |  |  |  |  |
| _15 | Because you are just not interested? |  |  |  |  |  |  |

*All respondents completing this module skip to Module 17.*

# MODULE 16: Motivations For/Against, and Past Outcomes of, Drinking – NON-DRINKERS ONLY (MTND)

***[This question is for Past-Drinkers (Drank alcohol in the past but not in the past 12 months). Never drinkers go to MOTV.AGST]***

…EFCT Drinking affects people in many different ways. We would like to learn what effects drinking may have had on you. When you used to drink, how true would you say each of these statements was for you – very often true, often true, sometimes true, rarely true, or never true?

|  | How true is it that when you used to drink… | 1 – V ERY OFTEN TRUE | 2 – OFTEN TRUE | 3 – SOMETIMES TRUE | 4 – RARELY TRUE | 5 – NEVER TRUE | 98 – DON’T KNOW | 99 – REFUSED |
| --- | --- | --- | --- | --- | --- | --- | --- | --- |
| _1 | You felt relaxed? |  |  |  |  |  |  |  |
| _2 | You felt happy? |  |  |  |  |  |  |  |
| _3 | You became more aggressive toward other people? |  |  |  |  |  |  |  |
| _4 | You felt more friendly and outgoing? |  |  |  |  |  |  |  |
| _5 | You found it easier to talk about your feelings or problems? |  |  |  |  |  |  |  |
| _6 | You forgot your problems? |  |  |  |  |  |  |  |
| _7 | You did something you later regretted? |  |  |  |  |  |  |  |
| _8 | Sexual activity was more pleasurable for you? |  |  |  |  |  |  |  |
| _9 | You felt more sexually attractive? |  |  |  |  |  |  |  |
| _10 | You got into trouble with police? |  |  |  |  |  |  |  |
| _11 | You had a lot of fun? |  |  |  |  |  |  |  |
| _12 | You felt sick |  |  |  |  |  |  |  |
| _13 | You didn’t remember what happened (you blacked out)? |  |  |  |  |  |  |  |

***[This question is for Past-Drinkers (Drank alcohol in the past but not in the past 12 months). Never drinkers go to MOTV.AGST]***

…MOTV.FOR People have different reasons for drinking alcohol. When you used to drink, how important would you say each of the following reasons was for you, personally? Would you say very important, important, not very important, or not at all important?

|  |  | 1 – V ERY IMPORTANT | 2 –IMPORTANT | 3 – NOT VERY IMPORTANT | 4 – NOT AT ALL IMPORTANT | 98 – DON’T KNOW | 99 – REFUSED |
| --- | --- | --- | --- | --- | --- | --- | --- |
| _1 | To be sociable or polite? |  |  |  |  |  |  |
| _2 | Because others were drinking? |  |  |  |  |  |  |
| _3 | To add to the enjoyment of meals? |  |  |  |  |  |  |
| _4 | For health reasons? |  |  |  |  |  |  |
| _5 | To feel good? |  |  |  |  |  |  |
| _6 | To help you relax? |  |  |  |  |  |  |
| _7 | To forget worries? |  |  |  |  |  |  |
| _8 | To help you feel less inhibited or shy? |  |  |  |  |  |  |
| _9 | To celebrate? |  |  |  |  |  |  |
| _10 | Because of the taste? |  |  |  |  |  |  |
| _11 | Because of thirst? |  |  |  |  |  |  |

***[This question is for both Past-Drinkers and Never Drinkers]***

…MOTV.AGST People also have different reasons for limiting their drinking, or not drinking alcohol at all. How important would you say each of the following reasons is for you, personally? Would you say very important, important, not very important, or not at all important?

|  |  | 1 – V ERY IMPORTANT | 2 –IMPORTANT | 3 – NOT VERY IMPORTANT | 4 – NOT AT ALL IMPORTANT | 98 – DON’T KNOW | 99 – REFUSED |
| --- | --- | --- | --- | --- | --- | --- | --- |
| _1 | [Skip if respondent is male]  Because you were or are pregnant, or trying to become pregnant? |  |  |  |  |  |  |
| _2 | Because of the taste? |  |  |  |  |  |  |
| _3 | Because you don’t like the effect it has on you? |  |  |  |  |  |  |
| _4 | Because you have seen bad examples of what alcohol can do? |  |  |  |  |  |  |
| _5 | Because you have been hurt by someone else’s drinking? |  |  |  |  |  |  |
| _6 | Because drinking could affect your work or school performance? |  |  |  |  |  |  |
| _7 | Because drinking is too expensive or is a waste of money? |  |  |  |  |  |  |
| _8 | Because of religious reasons? |  |  |  |  |  |  |
| _9 | Because you were brought up not to drink? |  |  |  |  |  |  |
| _10 | Because you have had alcohol problems or are afraid of becoming an alcoholic? |  |  |  |  |  |  |
| _11 | Because you are too young? |  |  |  |  |  |  |
| _12 | Because your friends and/or family members disapprove of you drinking? |  |  |  |  |  |  |
| _14 | For health reasons? |  |  |  |  |  |  |
| _15 | Because you are just not interested? |  |  |  |  |  |  |

# MODULE 17: Own and Others’ Perceptions of Alcohol and Other Substances (PCPN)

...GEN Please tell me whether you strongly agree, agree, neither agree nor disagree, disagree, or strongly disagree with each of the following statements. *(Show card)*

...GEN_1 Having a drink is one of the pleasures of life.

1 – STRONGLY AGREE

2 – AGREE

3 – NEITHER AGREE NOR DISAGREE

4 – DISAGREE

5 – STRONGLY DISAGREE

98 – DON’T KNOW

99 – REFUSED

...GEN_2 Having a drink with someone is a way of being friendly.

1 – STRONGLY AGREE

2 – AGREE

3 – NEITHER AGREE NOR DISAGREE

4 – DISAGREE

5 – STRONGLY DISAGREE

98 – DON’T KNOW

99 – REFUSED

...GEN_3 There is nothing good to be said about drinking.

1 – STRONGLY AGREE

2 – AGREE

3 – NEITHER AGREE NOR DISAGREE

4 – DISAGREE

5 – STRONGLY DISAGREE

98 – DON’T KNOW

99 – REFUSED

…SITS Next I am going to describe situations that people sometimes find themselves in. For each one, please tell me how much a person in that situation should feel free to drink – 0 drinks, some drinking but not enough to feel the effects (1 or 2 drinks), enough to feel effects but not become drunk, getting drunk is sometimes alright, or getting drunk is always alright? *(Show card)*

|  |  | 1 – 0 DRINKS | 2 – SOME DRINKING BUT NOT ENOUGH TO FEEL THE EFFECTS (1 OR 2 DRINKS) | 3 – ENOUGH TO FEEL THE EFFECTS BUT NOT BECOME DRUNK | 4 – GETTING DRUNK IS SOMETIMES ALRIGHT | 5 – GETTING DRUNK IS ALWAYS ALRIGHT | 98 – DON’T KNOW | 99 – REFUSED |
| --- | --- | --- | --- | --- | --- | --- | --- | --- |
| _2 | As a mother, spending time with small children |  |  |  |  |  |  |  |
| _3 | As a father, spending time with small children |  |  |  |  |  |  |  |
| _6 | For a man out at a bar with friends |  |  |  |  |  |  |  |
| _7 | For a woman out at a bar with friends |  |  |  |  |  |  |  |
| _8 | For a woman out with co-workers |  |  |  |  |  |  |  |
| _9 | For a man out with co-workers |  |  |  |  |  |  |  |
| _12 | For a man having dinner at home with his spouse or partner |  |  |  |  |  |  |  |
| _13 | For a woman having dinner at home with her spouse or partner |  |  |  |  |  |  |  |

# MODULE 20: Adolescents and Young Adults (ADYA)

## 20.3 Emerging Adulthood

EMAD.PERC Please tell me whether you strongly agree, agree, neither agree nor disagree, disagree, or strongly disagree with each of the following statements. *(Show card)*

...EMAD.PERC_1 You have reached adulthood.

1 – STRONGLY AGREE

2 – AGREE

3 – NEITHER AGREE NOR DISAGREE

4 – DISAGREE

5 – STRONGLY DISAGREE

98 – DON’T KNOW

99 – REFUSED

...EMAD.PERC_2 You are financially independent of your parents or other family members.

1 – STRONGLY AGREE

2 – AGREE

3 – NEITHER AGREE NOR DISAGREE

4 – DISAGREE

5 – STRONGLY DISAGREE

98 – DON’T KNOW

99 – REFUSED

...EMAD.PERC_3 You are emotionally independent of your parents or guardians.

1 – STRONGLY AGREE

2 – AGREE

3 – NEITHER AGREE NOR DISAGREE

4 – DISAGREE

5 – STRONGLY DISAGREE

98 – DON’T KNOW

99 – REFUSED

…STOP  *Record current time:*

__ __ : __ __ (HH:MM, 24:00 clock)

# MODULE 21: Respondent Engagement, Recruiting and Screening (RCRT)

We are now nearing the end of this interview. I would just like to ask you a few more questions.

…ENG1 On a scale of 1 to 10, with 1 being not at all interested and 10 being very interested, how interested would you say you were in this survey?

__ __

98 – DON’T KNOW

99 – REFUSED

…ENG2 And, also on a scale of 1 to 10, with 1 being not at all and 10 being very much, how much would you say you enjoyed taking part in this survey?

__ __

98 – DON’T KNOW

99 – REFUSED

Now, as I mentioned at the beginning it’s important to the success of this study that we are able to collect information from people with a variety of attitudes, views, and experiences - so thank you. We hope that many people who have taken part in this interview will consider taking part in the second stage. The second part of the study involves being asked to answer some questions online. Not everyone who agrees to take part in the second stage will be contacted .

...PART Would you like to be considered for part two of the study?

1 – YES, I AM INTERESTED IN PARTICIPATING IN PART 2 OF THE STUDY (Skip to …SCRN)

2 – NO, I WOULD NOT LIKE TO PARTICIPATE IN PART 2 OF THE STUDY

98 – DON’T KNOW

99 – REFUSED

 …PART.REF Would you please tell me why you do not wish to participate?

______________________ (open-ended response)

98 – DON’T KNOW

99 – REFUSED

(IF PART=2, GO TO END.)

…SCRN... Before we finish the interview I have a few final questions:

…SCRN.STDN If we think about 'students' as people who are currently undertaking some form of educational course or training what statement best describes you.

1 – I AM A FULL-TIME STUDENT

2 – I AM A PART-TIME STUDENT

3 – I AM NOT A STUDENT (Skip to …SCREEN.INT)

98 – DON’T KNOW

99 – REFUSED

…SCRN.EDUC What best describes the education or training you are enrolled in:

1 – HIGH SCHOOL COURSE (e.g. high school, secondary school)

2 – FURTHER EDUCATION COURSE (e.g. community college, technical college)

3 – HIGHER EDUCATION COURSE (e.g. university)

4 – OTHER (please specify) _______________________________________

98 – DON’T KNOW

99 – REFUSED

…SCRN.INT In general, how often do you use the internet?

1 – DAILY

2– WEEKLY

3 –MONTHLY

4 – LESS THAN MONTHLY

5 – NEVER

6 – I DO NOT HAVE ACCESS TO THE INTERNET

98 – DON’T KNOW

99 – REFUSED

…SCRN.CHCK In general, how often do you check your email account?

1 – DAILY

2 –WEEKLY

3 –MONTHLY

4 – LESS THAN MONTHLY

5 – NEVER

6 – I DO NOT HAVE AN EMAIL ACCOUNT

98 – DON’T KNOW

99 – REFUSED

(IF SCRN.INT = 5 OR 6 **AND** SCRN.CHCK = 5 OR 6, THEN END.)

(IF SCRN.INT = 1-4 **AND** SCRN.CHCK = 5 OR 6, THEN GO TO PART.CNTC.OTH)

(IF SCRN.INT = 1-4 **AND** SCRN.CHCK = 1-4, CONTINUE)

…PART.EMAIL We will be contacting people by email about the second stage of the study; do you have an email address where we can contact you?

1 – YES, MY EMAIL ADDRESS IS: ______________________________________________

***(Interviewer: Verify email address by asking respondent to repeat address.)***

2 – NO, I DO NOT HAVE AN EMAIL ADDRESS

98 – DON’T KNOW

99 – REFUSED

…PART.CNTC.OTH Could you provide us with an alternative point of contact for you in case we are unable to reach you by email, such as a phone number or an address?

1 – YES, PHONE NUMBER:_____________________________

***(Interviewer: Verify by asking respondent to repeat.)***

***2 – YES, ADDRESS: ____________________________________________***

***(Interviewer: Verify by asking respondent to repeat.)***

2 – NO, I DO NOT HAVE ANOTHER POINT OF CONTACT

98 – DON’T KNOW

99 – REFUSED

[THANK YOU AND END]
