# Supplementary material for: Context and culture associated with alcohol use amongst youth in major urban cities: A cross-country population based survey
Source: PLoS One. 2017 Nov 20;12(11):e0187812. doi: 10.1371/journal.pone.0187812 (PMC5695777; doi:10.1371/journal.pone.0187812)
Supplement: S3 Questionnaire — (DOC) [file pone.0187812.s003.doc]

**LTACC Questionnaire (Hausa Language)**

Final Draft

Contents

[Module A: Household Identification and Census, Consent, and Administrative Variables (ADMN) 3](#__RefHeading___Toc394931724)

[MODULE 1: Demographics](#__RefHeading___Toc394931725) 6

[MODULE 9: Health Behaviors, Including Drinker Status (HTBX)](#__RefHeading___Toc394931726) 10

[MODULE 10: Alcohol Consumption, Last 12 Months (CONS)](#__RefHeading___Toc394931727) 11

[10.1 General Alcohol Consumption 11](#__RefHeading___Toc394931728)

[10.2 Beverage-Specific Quantity/Frequency](#__RefHeading___Toc394931729) 12

[10.3 Experience of Drunkenness/Intoxication](#__RefHeading___Toc394931730) 14

[10.4 Drinking Context](#__RefHeading___Toc394931731) 14

[MODULE 15: Motivations For/Against, and Effects of, Drinking – DRINKERS ONLY (MTCD)](#__RefHeading___Toc394931732) 16

[MODULE 16: Motivations For/Against, and Past Outcomes of, Drinking – NON-DRINKERS ONLY (MTND)](#__RefHeading___Toc394931733) 19

[MODULE 17: Own and Others’ Perceptions of Alcohol and Other Substances (PCPN)](#__RefHeading___Toc394931734) 22

[MODULE 20: Adolescents and Young Adults (ADYA) 24](#__RefHeading___Toc394931735)

[20.3 Emerging Adulthood](#__RefHeading___Toc394931736) 24

[MODULE 21: Respondent Engagement, Recruiting and Screening (RCRT) 25](#__RefHeading___Toc394931737)

# Module A: Household Identification and Census, Consent, and Administrative Variables (ADMN)

***A.0 Household Identification, Date*** *(Complete before approaching the household/respondent)*

LEV1… City/metropolitan area:

__ __ (see codebook)

LEV2… [LGA ]:

__ __ (see codebook)

LEV3… [Wards ]:

__ __ (see codebook)

LEV4… [Neighbourhood/settlement ]:

__ __ (see codebook)

…LEV5 [Street ]:

__ __ (see codebook)

…LEV6 [Household ]:

__ __ (see codebook)

…INTID __ __ __ __

…DATE __ __ (dd) __ __ (mm) __ __ __ __ (yyyy)

***A.1 Household and Respondent Disposition*** *(Complete after contact with the household/respondent)*

…HDIS Household disposition:

__ __ (see codebook)

…RDIS: Respondent disposition:

__ __ (see codebook)

…RDIS_TXT Dalili nda ze hana mu da cigaba da wana tambaya (i.e., ADMN.RDIS = 25 or 26)

________________________________________________________________________

________________________________________________________________________

***A.2 Household Introduction, Census, Determination of Respondent Eligibility***

...INTR Sanu, Suna na […………..]. Ni maikata Dr A.B Makanjuola muna dindigi maska ajikin wandada sai muzu tambayoyi akan shaye shaye da karfi kuyiwa azakani matasa da suke cikin birni Ilorin. Yansu, zanyi maka tambaya nda akwai wanda ze iya shiga araka ajikin wana bincike.

*After obtaining consent:*

Na gode. Zaka iya gayamana ainiyi shekaru kona miji ko maje nda suke gidan na?

*Complete Columns 1 and 2 of the matrix below by entering ages and circling the correct gender. For every person listed who is between 18 and 34 years of age, ask whether s/he has lived in [country-specific city/metropolitan area] for at least 6 months and complete Column 3 as appropriate.*

Household Census

|  | **Nawan ne shekara nka** | Miji ko maje | Kataba sauna a birni bayan wata shida suwa yau? |  |  | **Nawan ne shekara nka** | Miji ko maje | Kataba sauna a birni bayan wata shida suwa yau |
| --- | --- | --- | --- | --- | --- | --- | --- | --- |
| **Mutun 1** |  | Miji /maje | Hee /Haha |  | **Mutun 7** |  | Miji /maje | Hee /Haha |
| **Mutun 2** |  | Miji /maje | Hee /Haha |  | **Mutun 8** |  | Miji /maje | Hee /Haha |
| **Mutun 3** |  | Miji /maje | Hee /Haha |  | **Mutun 9** |  | Miji /maje | Hee /Haha |
| **Mutun 4** |  | Miji /maje | Hee /Haha |  | **Mutun 10** |  | Miji /maje | Hee /Haha |
| **Mutun 5** |  | Miji /maje | Hee /Haha |  | **Mutun 11** |  | Miji /maje | Hee /Haha |
| **Mutun 6** |  | Miji /maje | Hee /Haha |  | **Mutun 12** |  | Miji /maje | Hee /Haha |

*If no eligible respondent, move to next household. If multiple eligible respondents, select respondent using last birthday method and ask to speak to selected respondent. Skip to A.1 and complete the questions below only after* interviewing (or attempting to interview) the selected respondent.

…LANG2 *Language in which interview was conducted:*

1. Yoruba
2. Hausa
3. Ibo
4. English

…LANG3 *Rate the respondent’s apparent comfort with the interview language, on a scale of 1 to 5 (1 = respondent unable to understand, could not finish interview; 5 = respondent fluent, appeared to understand every question with ease): __*

***A.3 Respondent Introduction and Consent***

...INST1 Sannu, nine suna [suna]. Ni kwararren matabiya wan da na ke aiki da [Dr A.B Makanjuola] chikin binchike-binchike wanda International Center for Alcohol Policies, in Washington, DC, USA take dauki nauyin. An zabi addresin ka ne ido-a-rufe don ka zama a chikin wadanda za ayi musu tambayoyi [bayani akan binchike-binchiken]. Yana da muhimmanchi korai da mu yi nasara a wananan binchiken da zamu iya tara baiyane daga mutane wanda halayin su, da tunaninsu, da dabiyarsu, da jin ya zama dalili nda mutane karnuka. Wannan binceke-binciken yana nema ka ansa tamboyoyi gameda (dadin jikinki, halayen ka, rayuwan ka, tara shan giya da gwayoyi). Binchike-binchiken zai dauki lokaci [ ranaku ] domin a chikita su. Raayinka ne ko zaka yarda ko kuwa baka amince ba. In ka yard aka chigaba da binchike-binchiken nan, raayinka zamu tsareshi musamman mu kuwa kyabe su. In akwai tambayo da baka ji dadi da suba zaka iya barsu. Zaka iya bar chikita wannan tambayoyin a kowani lokaci ka so. Ka yarda ka jona wannan binchike-binchiken nan ?

________

Initials of interviewer, indicating

verbal consent of respondent

***A.4 Screening and Administrative Questions***

...INST2 Mu gode maka. Kafin mu je gaba, Ina so in yi maka tamboyi kalilan dan in sani ko kai dai-dai ka chikita wannan binchike-binchiken.

...BYR Wani shekara aka haife ka?

__ __ __ __

98 – Ban sani ba

99 – Naki ansan

*Probe if, based on birth year, respondent may be younger than 18 (born in 1996) or older than 34 (born in 1980). Interview ends if respondent is not in desired age range, or age cannot be determined. [Code respondent as “Ineligible”.]*

…RESI Kataba sauna a birni bayan wata shida suwa yau?

1 – A

2 – AA

98 – BAN SANI BA

99 – NA KI

*Interview ends if respondent has not resided in city/metropolitan area for at least the previous 6 months. [Code respondent as “Ineligible”.]*

…LANG1 Yarenka?

1. Yoruba
2. Hausa
3. Ibo
4. Sauran yare

98– Ban sani ba

99 – Naki ansan

*Interview is conducted in respondent’s primary language, if possible. If this is not possible, or the respondent’s primary language is not known, rate (on previous page) the respondent’s level of comfort with the language in which the interview was conducted upon the conclusion of the interview.*

***A.5 Introduction to Interview***

…INST3 In ya gamsheka, zan fara da tamboyin. Ka tuna fa ba zan bar wanni ya ga ansoshinka ba, sai ka ansa tamboyin yadda ya kamata. In baka yarda da tambayan ba ko kuwa baka san ansan ba, sai ka gaya mini don mu jag aba zuwa tambaya na biye.

…STRT  *Rubuta locaci:*

__ __ : __ __ (HH:MM, 24:00 clock)

# MODULE 1: Demographics

…Sex Mace ko miji *(Ka chikita ko kai na mace ne ko na miji.)*

1 – Miji

2 – Mace

98 – Ban sani ba

99 – Naki ansan

...MAR Wannene ya zo dai – dai da aurantakarka?

1 – INA DA AURE

2 – NAYI AURE AMA MUM RABU

3 – NA BIYU NA TA/YA RASU

4 – BAN TABA AURE BA (Ka yi challe zuwa …HH.AD)

98 – Ban sani ba (Ka yi challe zuwa …HH.AD)

99 – Naki ansan (Ka yi challe zuwa …HH.AD)

…MAR_FU Wani shakara (kayi aure/ka sake na biyun ka/ki ko kuwa rabuwa/rasuwar)?

__ __ __ __

98 – BAN SANI BA

99 – NAKI ANSAN

...HH.AD Banda kai, nawa ne wanda sun fi saka sha takwas (18) a gidan ka?

__ __ ADULTS *(If 0, skip to DEM.HH.AD_FU2)*

98 – Ban sani ba

99 – Naki ansan

…HH.AD_FU1 Su wanene su? (Ka zaba wadda ya dache)

1 – NA BIYU NA

2 – IYAYE NA(Je zuwa *DEM.HH.PNT1)*

3 – WADANSU YAN IYALAI NA

4 – ABOKANE, YAN ZAMA TARE,

SAURA WANDA BA IYALAI NA BANE

98 – Ban sani ba

99 – Naki ansan

…HH.AD_FU2 Wani shekara ka bar kadan iyayenka ka fara zama kai kadai?

__ __ __ __

98 – NA MANTA

99 – NAKI ANSAN

...PNT1 Kanada yara?

1 – NA AM, INA DA YARA

2 – AA BANI DA YARA *(Je zuwa DEM.EDU)*

98 – BAN SAN IBA *(Je zuwa DEM.EDU)*

99 – NAKI ANSAN *(Je zuwa DEM.EDU)*

…PNT1_FU Yaran ka nawa?

Yara ______

98 – NA MANTA *(Je zuwa DEM.EDU)*

99 – NA KI *(Je zuwa DEM.EDU)*

…PNT2 …PNT2_FU

| Yaro/yarinya # | Shekarun su? (Ka fara da karamin) | Yana ko tana zama tare da kai? |
| --- | --- | --- |
| 1 | PNT2_1  SHEKARU  98 – BAN SANI BA  99 – NA KI | PNT2_1_FU  1 – A 2 – AA  98 – BAN SANI BA  99 – NA KI |
| 2 | PNT2_2  SHEKARU  98 – BAN SANI BA  99 – NA KI | PNT2_2_FU  1 – A 2 – AA  98 – BAN SANI BA  99 – NA KI |
| 3 | PNT2_3  SHEKARU  98 – BAN SANI BA  99 – NA KI | PNT2_3_FU  1 – A 2 – AA  98 – BAN SANI BA  99 – NA KI |
| 4 | PNT2_4  SHEKARU  98 – BAN SANI BA  99 – NA KI | PNT2_4_FU  1 – A 2 – AA  98 – BAN SANI BA  99 – NA KI |
| 5 | PNT2_5  SHEKARU  98 – BAN SANI BA  99 – NA KI | PNT2_5_FU  1 – A 2 – AA  98 – BAN SANI BA  99 – NA KI |
| 6 | PNT2_6  SHEKARU  98 – BAN SANI BA  99 – NA KI | PNT2_6_FU  1 – A 2 – AA  98 – BAN SANI BA  99 – NA KI |
| 7 | PNT2_7  SHEKARU  98 – BAN SANI BA  99 – NA KI | PNT2_7_FU  1 – A 2 – AA  98 – BAN SANI BA  99 – NA KI |
| 8 | PNT2_8  SHEKARU  98 – BAN SANI BA  99 – NA KI | PNT2_8_FU  1 – A 2 – AA  98 – BAN SANI BA  99 – NA KI |

...EDU Yaya girman karatun da ka yi?

1. Ba n je makaranta ba, ba n gama makaranta ba, na gama makaranta
2. Ba n gama sekondiri, na gama sekondiri (6 – 12 years)
3. Ba ngama fasiti, nna da OND, College of Education i.e NCE, and Nursing (12-15yrs)
4. Na gama fasiti (e.g., e.g university graduate, polytechnic graduate (HND), post-graduate degree (16+ of education)

98 – BAN SANI BA

99 – NA KI ANSAN

…STDT Ka na makaranta ne?

1 – A *(Je zuwa DEM.EMPL)*

2 – AA

98 – BAN SANI BA

99 – NA KI

…STDT_GR Wani shakara ka kalmasa ko ka beri karatun ka?

__ __ __ __

98 – NA MANTA

99 – NA KI

...EMPL Yaya zaka bayyana sanaarka?

1 – INA AIKi CHIKA (saa 40 ko fiye a mako, kuma aiki na wo ni) *(Je zuwa EMPL_FU3)*

2 – AIKI LOKACE-LOKACE (saa kasa da 40 a mako, kuma aiki na wo ni) *(Je zuwa EMPL_FU3)*

3 – BANA AIKI

4 – AIKIN KIGA

5 – INA RAUNI, BAN IYA AIKI BA *(Je zuwa DEM.OCC)*

6 – WASU: _______________________________________________ *(je zuwa DEM.EMPL.FU1)*

98 – BAN SANI BA

99 – NA KI

...EMPL_FU1 Ka neman aiki yanzu?

1 – A

2 – AA *(Je zuwa EMPL_FU4.YR)*

98 – BAN SANI BA *(Je zuwa_FU4.YR)*

99 – NAKI *(Je zuwa EMPL_FU4.YR)*

...EMPL_FU2 Wanna zai zan aikin farko da aka biyaka?

1 – A *(Je zuwa DEM.OCC)*

2 – AA *(Je zuwa EMPL_FU4)*

98 – BAN SANI BA *(Je zuwa EMPL_FU4.YR)*

99 – NA KI *(Je zuwa EMPL_FU4.YR)*

...EMPL_FU3 Wannane aiki na farko da zaa biya ka?

1 – A

2 – AA

98 – BAN SANI BA

99 – NA KI

…EMPL_FU4. Wani shekara ka fara aiki wonda an bia kudi?

__ __ __ __

97 – BAN TABA YI AIKI WADDA AKA BIYANI BA

98 – BAN SANI BA

99 – NA KI

…OCC Menene sanaar ka? In baka aiki yanzu amma ka taba aiki, ka bayana sanaar da ka yi aiki a chiki. In dai ka yi aiki a wurare da yawa, ka zaba wanda ka dade a chiki.

| **1** | ARMED FORCES (Dan yaki) |
| --- | --- |
| **2** | LEGISLATORS/SENIOR OFFICIALS/MANAGERS (Siasa, Mayan maikata) |
| **3** | PROFESSIONALS (Likita, Inginia loyoyi, alkali) |
| **4** | TECHNICIANS AND ASSOCIATE PROFESSIONALS (Sanaar anu) |
| **5** | CLERKS (maikata) |
| **6** | SERVICE WORKERS/SHOP/MARKET SALES WORKERS (Dan kasua) |
| **7** | SKILLED AGRICULTURAL/FISH WORKERS (manome) |
| **8** | CRAFT AND RELATED WORKERS (Sanaar anu kadan) |
| **9** | PLANT/MACHINE OPERATORS/ASSEMBLERS (Makanike) |
| **10** | ELEMENTARY OCCUPATIONS (Labora) |
| **97** | I HAVE NEVER BEEN EMPLOYED (ba aiki) |
| **98** | DON’T KNOW (Ban sani ba) |
| **99** | REFUSED Na ki |

…REL Addinin ka?

1 – AGNOSTIC/ATHEIST NE

2 – BUDDHIST NE

3 – KRISTA NE

4 – HINDU NE

5 – JEWISH NE

6 – MUSULUMI NE

7 – SIKH NE

8 – WASU ADDINI: ___________________________________

98 – BAN SANI BA

99 – NA KI

…RACE Kabilan ka?

1. Yoruba
2. Hausa
3. Ibo
4. Wasu………

98 – BAN SANI BA

99 – NA KI

# MODULE 9: Health Behaviors, including Drinker Status (HBTX)

# Dadin kiji da yanayin shaye-shaye (HTBX)

…ALC.EVER Ka taba shaye-shaye wadda yake da giya, kamar [giyar kasa], da saura? Kada ka sa lokotai da aka baka ka kurba daya ko biyu ba.

1 – A

2 – AA *(Je zuwao MTND.MOTV.AGST)- for non-drinkers*

98 – BAN SANI BA

99 – NA KI

…ALC.STRT Shekarun ka nawa ne da ka fara shan giya? Kada ka sa lokatai da ka kurba daya ko biyu.

SHEKARU __ __

98 – BAN SANI BA

99 – NA KI

…ALC.DRUK Shakarun ka nawa ne lokachin da ka fara maye?

SHEKARU__ __

97 –BAN TABA MAYE BA

98 – BAN SANI BA

99 – NA KI

…ALC.HVY.STRT Ina so in sani shekarun ka a lokachin da ka fara shan giya sosai do kuma lokachinda ka rage. Kana shekarun ka nawa ka fara shan giya sosai.

SHEKARU __ __

98 – BAN SANI BA

99 – NA KI

…ALC.HVY.STOP Shekarun ka nawa lokachin da ka rage shan giya sosai?

SHEKARU__ __

97 – BAN TSAYAR BA

98 – BAN SANI BA

99 – NA KI

# MODULE 10: Alcohol Consumption, last 12 months (CONS)

# Shan Giya, Watanne 12 da sun wuce (CONS)

## 10.1 General Alcohol Consumption (Shan giya)

…GEN.FQ Watanne 12 da sun wuce, yaya kake shan giya, wine, spirits (kamar vodka, gin, whisky, brandy) ko wasu shaye-shayen giya ko kadan ne *(ka nuna kati)*

1 – KULLUM

2 – 5 ZUWA 6 A MAKO

3 – 3 ZUWA 4 A MAKO

4 – 1 ZUWA 2 A MAKO

5 – 2 ZUWA 3 A WATA

6 – DAYA A WATA

7 – 6 ZUWA 11 A WATANNE 12 DA TA WUCE

8 – 2 ZUWA 5 A WATANNE 12 DA TA WUCE

9 – DAYA A WATANNE 12 DA TA WUCE

10 – BAN SHA GIYA BA A WATANNE 12 DA TA WUCE *(Je zuwa MTND.EFCT)*

98 – BAN SANI BA

99 – NAKI

…GEN.QY A watanne 12 da sun wuce, nawa shaye-shayen giya da ka sha a rana? Shaye-shaye nada ke nuna [SHAYE-SHAYE NA KASAN KA]

1 – GIYA 25 drinks KO FIYE

2 – GIYA 19 ZUWA 24

3 – GIYA 16 ZUWA 18

4 – GIYA 12 ZUWA 15

5 – GIYA 9 ZUWA 11

6 – GIYA 7 ZUWA 8

7 – GIYA 5 ZUWA 6

8 – GIYA 3 ZUWA 4

9 – GIYA 2

10 – GIYA 1

11 – KARA DA GIYA 1 CHIKE *(In dukka CONS.GEN.FQ = 9 da GEN.QY=11, Je zuwa MTND.EFCT)*

98 – BAN SANI BA

99 – NAKI

…GEN.MST.QY A wane 12 da sun wuce, nawane shaye-shaye da ke da giya ka sha a saa 24? *(Show card)*

1 – GIYA 36 drinks KO FIYE

2 – GIYA 25 ZUWA 35

3 – GIYA 19 ZUWA 24

4 – GIYA 16 ZUWA 18

5 – GIYA 12 ZUWA 15

6 – GIYA 9 ZUWA 11

7 – GIYA 7 ZUWA 8

8 – GIYA 5 ZUWA 6

9 – GIYA 2

10 – GIYA 1

11 – KARA DA GIYA 1 CHIKE

98 – BAN SANI BA

99 – NAKI

…GEN.MST.FQ A watanne 12 da sun wuce, yaya ka ke shan giya [ansa daga CONS.GEN.MST.QY] chinkin saa 24? *(Show card)*

1 – KULLUM

2 – 5 ZUWA 6 A MAKO

3 – 3 ZUWA 4 A MAKO

4 – 1 ZUWA 2 A MAKO WEEK

5 – 2 ZUWA 3 A MAKO

6 – DAYA A WATA

7 – 6 ZUWA 11 A WATANNE 12 DA SUN WUCE

8 – 2 ZUWA 5 A WATANNE 12 DA SUN WUCE

9 – DAYA A WATANNE 12 DA SUN WUCE

98 – BAN SANI BA

99 – NAKI

## 10.2 (Beverage- specific quantity/ frequency) YAWAN KAYAN SHAYE-SHAYE

Yanzu ina so in tambaye ka nawane kake shan wani kayan shaye-shaye a watanne 12 to sun wuce da kuma yaya yawan da kake sha a rana.

…BSFQ.BR.FQ Yaya kake shan **beer** a watanne 12 da sun wuce? *(Show card)*

1 – KULLUM

2 – 5 ZUWA 6 A MAKO

3 – 3 ZUWA 4 A MAKO

4 – 1 ZUWA 2 A MAKO WEEK

5 – 2 ZUWA 3 A MAKO

6 – DAYA A WATA

7 – 6 ZUWA 11 A WATANNE 12 DA SUN WUCE

8 – 2 ZUWA 5 A WATANNE 12 DA SUN WUCE

9 – DAYA A WATANNE 12 DA SUN WUCE

10 – BAN SHA BA A WATANNE 12 DA SUN WUCE *(Je zuwa CONS.BSFQ.WN.FQ)*

98 – BAN SANI BA

99 – NAKI

…BSFQ.BR.QY A ranan da ka sha bear, nawa bear da kake sha?

__ __ *(Show card for standard drink)*

98 – BAN SANI BA

99 – NAKI

…BSFQ.WN.FQ Yaya kake shan **wine** a watanne 12 da sun wuce? *(Show card)*

1 – KULLUM

2 – 5 ZUWA 6 A MAKO

3 – 3 ZUWA 4 A MAKO

4 – 1 ZUWA 2 A MAKO WEEK

5 – 2 ZUWA 3 A MAKO

6 – DAYA A WATA

7 – 6 ZUWA 11 A WATANNE 12 DA SUN WUCE

8 – 2 ZUWA 5 A WATANNE 12 DA SUN WUCE

9 – DAYA A WATANNE 12 DA SUN WUCE

10 – BAN SHA BA A WATANNE 12 DA SUN WUCE *(Je zuwa BSFQ.SP.FQ)*

98 – BAN SANI BA

99 – NAKI

…BSFQ.WN.QY A rana in kana shan wine, wine nawa ka ke sha?

__ __ *(Show card for standard drink)*

98 – BAN SANI BA

99 – NAKI

…BSFQ.SP.FQ Yaya kake shan **spirits** a watanne 12 da sun wuce *(Show card)*

1 – KULLUM

2 – 5 ZUWA 6 A MAKO

3 – 3 ZUWA 4 A MAKO

4 – 1 ZUWA 2 A MAKO WEEK

5 – 2 ZUWA 3 A MAKO

6 – DAYA A WATA

7 – 6 ZUWA 11 A WATANNE 12 DA SUN WUCE

8 – 2 ZUWA 5 A WATANNE 12 DA SUN WUCE

9 – DAYA A WATANNE 12 DA SUN WUCE

10 – BAN SHA BA A WATANNE 12 DA SUN WUCE *(je zuwa to CONS.BSFQ.OTR.FQ)*

98 – BAN SANI BA

99 – NAKI

…BSFQ.SP.QY A rana in kana shan spirit, spirit nawa ka ke sha?

__ __ *(Show card for standard drink)*

98 – BAN SANI BA

99 – NAKI

…BSFQ.OTR.FQ Yaye ka ke shan **[KAYAN SHAYE-SHAYEN KASANKA]** a watanne 12 da sun wuce? *(Show card)*

1 – KULLUM

2 – 5 ZUWA 6 A MAKO

3 – 3 ZUWA 4 A MAKO

4 – 1 ZUWA 2 A MAKO WEEK

5 – 2 ZUWA 3 A MAKO

6 – DAYA A WATA

7 – 6 ZUWA 11 A WATANNE 12 DA SUN WUCE

8 – 2 ZUWA 5 A WATANNE 12 DA SUN WUCE

9 – DAYA A WATANNE 12 DA SUN WUCE

10 – BAN SHA BA A WATANNE 12 DA SUN WUCE *(je zuwa DRUK.FQ)*

98 – BAN SANI BA

99 – NAKI

…BSFQ.OTR.QY A rana in ka sha [country-specific beverage], [country-specific beverage] nawa ka ke sha?

__ __ *(Show card for standard drink)*

98 – BAN SANI BA

99 – NAKI

## 10.3 Experience of Drunkenness/Intoxication

…DRUK.FQ Yaya a watanne 12 da sun wuce ka sha giya sosai har ka yi maye – ko ka ji kamar jiri, ko idanunka basu gani sosai, ko kuwa kalmomin k aba maana*(Show card)*

1 – KULLUM

2 – 5 ZUWA 6 A MAKO

3 – 3 ZUWA 4 A MAKO

4 – 1 ZUWA 2 A MAKO WEEK

5 – 2 ZUWA 3 A MAKO

6 – DAYA A WATA

7 – 6 ZUWA 11 A WATANNE 12 DA SUN WUCE

8 – 2 ZUWA 5 A WATANNE 12 DA SUN WUCE

9 – DAYA A WATANNE 12 DA SUN WUCE

10 – BABU A WATANNE 12 DA SUN WUCE *(je zuwa CONS.CXT1)*

98 – BAN SANI BA

99 – NAKI

…DRUK.NM Giya nawa ne ke sa ka maye? (1 drink is *(Show card for standard drink)*.)

GIYA__ __

98 – BAN SANI BA

99 – NAKI

## 10.4 Drinking Context

Yanzu zan tambaye ka yadda kake shan giya a wasu yanayin.

|  | A watanne 12 do sun wuse, yaye kake shan giya bayan… | 1- KO WANI RANA KO KUSA DA KO WANI RANE | 2 – DAYA KO FIYE A MAKO | 3 – DAYA KO FIYE A WATA AMMA KASA DAYA A MAKO | 4 – FIYE DA DAYA A WATANNE 12 DA SUN WUCE KO KASA DA DAYA A WATA | 5 – BABU A WATANNE 12 DA SUN WUCE | 98 – BAN SANI BA | 99 – NA KI |  | Nawa giya ka ke sha? *(ka tsallaka in 5, 98, ko 99)* | | |
| --- | --- | --- | --- | --- | --- | --- | --- | --- | --- | --- | --- | --- |
| …CXT1 | Bayan abinkin yamma? |  |  |  |  |  |  |  |  | 1.NM |  | |
|  |  |  |  |  |  |  |  |  |  |  | 98 | 99 |
| …CXT2 | Bayan abinkin rana? |  |  |  |  |  |  |  |  | 2.NM |  | |
|  |  |  |  |  |  |  |  |  |  |  | 98 | 99 |
| …CXT3 | A kadan shaye-shaye, ko kuwa buki? |  |  |  |  |  |  |  |  | 3.NM |  | |
|  |  |  |  |  |  |  |  |  |  |  | 98 | 99 |
| …CXT4 | Zama da wasu a gida, tara do buki. |  |  |  |  |  |  |  |  | 4.NM |  | |
|  |  |  |  |  |  |  |  |  |  |  | 98 | 99 |
| …CXT5 | A gida lokacin hutawa? |  |  |  |  |  |  |  |  | 5.NM |  | |
|  |  |  |  |  |  |  |  |  |  |  | 98 | 99 |
| …CXT6 | In abokane sun kawo ziyara? |  |  |  |  |  |  |  |  | 6.NM |  | |
|  |  |  |  |  |  |  |  |  |  |  | 98 | 99 |
| …CXT7 | Zama da abokane a wurin hutawan jamaa? |  |  |  |  |  |  |  |  | 7.NM |  | |
|  |  |  |  |  |  |  |  |  |  |  | 98 | 99 |

…CXT.MEAL A watanne 12 da sun wuce, kamar nawane ka sha giya bayan abinchi? Kamar …

1 – DUKKA KO SUSA DO DUKKA?

2 – FIYE DA RABI?

3 – RABI?

4 – KASA DA RABI?

5 – BABU KO KUSA DA BABU?

98 – BAN SANI BA

99 – NAKI

…CXT.SELF Zan tambaye ka yaya kake shan giya da wasu. A watanne 12 da sun wuce, nawa ne ka yi shaye-shayen giya kai kadai? Za iya ce…

1 – DUKKA KO SUSA DO DUKKA?

2 – FIYE DA RABI?

3 – RABI?

4 – KASA DA RABI?

5 – BABU KO KUSA DA BABU?

98 – BAN SANI BA

99 – NAKI

# MODULE 15: Motivations for/against and effects of drinking- DRINKERS ONLY (MTCD)

# Abinda ke sa ko hana da amfanin giya – MASU MAYE KADAI (MTCD)

…EFCT Shan giya ke chanja mutane daban-daban. Muna so mu koyi abinda shan giya zai kawo maka. In ka sha giya, yaya gaskiyan za ka wadannan kalmomin sun yi daidai maka – dai dai kullum, wasu lokatain gaskiya, ba kullum ba gaskiya?

|  | Yaya gaskiyan in ka sha giya… | 1 – KULLUM GASKIYA NE | 2 – KULLUM KASKIYA | 3 – WASU LOKATAI GASKIYA | 4 – BA KULLUM BA GASKIYA | 5 – BABU GASKIYA | 98 – BAN SANI BA | 99 – NA KI |
| --- | --- | --- | --- | --- | --- | --- | --- | --- |
| _1 | Zaka ji ka huta? |  |  |  |  |  |  |  |
| _2 | Zaka ji dadi? |  |  |  |  |  |  |  |
| _3 | Zaka yi kunaguni da wasu? |  |  |  |  |  |  |  |
| _4 | Zaki ji kana da farin jinni da mutanne? |  |  |  |  |  |  |  |
| _5 | Zaki ji saukin Magana da mutanne? |  |  |  |  |  |  |  |
| _6 | Zaka manta da domuwanka? |  |  |  |  |  |  |  |
| _7 | Zak aka yi abu wanda baza ji dadi ba bayan? |  |  |  |  |  |  |  |
| _8 | Zaka ji dadin ayukan jamai ? |  |  |  |  |  |  |  |
| _9 | Zaka ji dadin jamai? |  |  |  |  |  |  |  |
| _10 | Zaka shiga damuwa da yansanda? |  |  |  |  |  |  |  |
| _11 | Zaka ji dadi korai? |  |  |  |  |  |  |  |
| _12 | Zaka ji rashinlafiya |  |  |  |  |  |  |  |
| _13 | Ba zaka tuna da abubuwan ba (zaki ji tas)? |  |  |  |  |  |  |  |

…MOTV.FOR Mutane suna da abubuwa dabam dabam wanda ke sa su shan giya. Yaya amfanin wayan nan zaka ce gareka? Zaka ce amfani sosai, amfani, ba amfani sosai ba, ko ba amfani?

|  |  | 1 – AMFANI SOSAI | 2 – AMFANI | 3 – BA AMFANI SOSAI | 4 – BA AMFANI | 98 – BAN SANI BA | 99 – NA KI |
| --- | --- | --- | --- | --- | --- | --- | --- |
| _1 | Zaka yi faraa da abokane? |  |  |  |  |  |  |
| _2 | Domin wasu ke shan giya? |  |  |  |  |  |  |
| _3 | Zai kara dadin abinci? |  |  |  |  |  |  |
| _4 | Don magini? |  |  |  |  |  |  |
| _5 | Don jin dadi? |  |  |  |  |  |  |
| _6 | Don taimakon hutawa? |  |  |  |  |  |  |
| _7 | Don manta da damuwa? |  |  |  |  |  |  |
| _8 | Don ya hana kunya? |  |  |  |  |  |  |
| _9 | Don buki? |  |  |  |  |  |  |
| _10 | Don dadi a baki? |  |  |  |  |  |  |
| _11 | Don kishin? |  |  |  |  |  |  |

…MOTV.AGST Mutane da dalilai dabam-dabam da ya sa sun rage shaye-shaye ko barin shaye-shaye gabakindayanta. Yaya zaka ce da wadannan dalilai rareka? Zaka iya ce da amfani sosai, da amfani, ba amfani sosai, ko ba amfani?

|  |  | 1 – AMFANI SOSAI | 2 – AMFANI | 3 – BA AMFANI SOSAI | 4 – BA AMFANI | 98 – BAN SANI BA | 99 – NA KI |
| --- | --- | --- | --- | --- | --- | --- | --- |
| _1 | Domin ina da chiki, ko kuwa ina so in yi chiki? |  |  |  |  |  |  |
| _2 | Domin dadin a baki? |  |  |  |  |  |  |
| _3 | Domin baka so yanda yake maida kai? |  |  |  |  |  |  |
| _4 | Domin ka ga barnan giya? |  |  |  |  |  |  |
| _5 | Domin ka ga yanda giya ke wasu? |  |  |  |  |  |  |
| _6 | Domin shan giya zai sa matsala a aikin ka ko maranta? |  |  |  |  |  |  |
| _7 | Domin giya na da tsada ko kuwa ke batarda kudi? |  |  |  |  |  |  |
| _8 | Domin addini? |  |  |  |  |  |  |
| _9 | Domin baka girma da shan giya ba? |  |  |  |  |  |  |
| _10 | Domin ka ji barnan giya ko baka so ka zama mashayi? |  |  |  |  |  |  |
| _11 | Domin ina yaro harzanzu? |  |  |  |  |  |  |
| _12 | Domin abokanenka do iyalinka basaso? |  |  |  |  |  |  |
| _14 | Don rashin lafiya? |  |  |  |  |  |  |
| _15 | Don baka son? |  |  |  |  |  |  |

*Duk wanda shun chika wannan sassi su je Module 17.*

**MODULE 16: Motivations for/against ad past outcomes of drinking- NON-DRINKERS ONLY (MTND)**

# (This question is for past drinkers (Drank alcohol in the past but not in the past 12 months). Never drinkers go to MOTV.AGST)

# Dalilin da Sa/Ya hana, da Sanadiyan Shaye-Shaye– MARASA SHAYE-SHEYE ONLY (MTND)

*[Wannan tambaye don wadanda sun barayo shaye-shaye (Wanda suna shan giya amma sun bari a watanne 12 da sun wuce). Wadanda sun bari shan giya su wuce MOTV.AGST]*

…EFCT Shaye-shaye ke damu mutanne a hanyoyi dawaya. Muna so mu koya daga yadday ke dame ka. Lokachin da ka ke shan giya, yaya gaskiyan yadda wadannan ke dameki – kullayomi, kullum, wasu lokatai, ba kullum ba, babu?

|  | Yaya gasgiyan lokachi da kake shan giya… | 1 – KULLAYOMI | 2 – KULLUM | 3 – WASU LOKACHI | 4 – WASU LOKACHI KALILAN | 5 – BA KULLUM BA | 98 – BAN SANI BA | 99 – NA KI |
| --- | --- | --- | --- | --- | --- | --- | --- | --- |
| _1 | Ina jin wutawa? |  |  |  |  |  |  |  |
| _2 | Na ji dadi? |  |  |  |  |  |  |  |
| _3 | Na yi kunaguni da wasu? |  |  |  |  |  |  |  |
| _4 | Na ji kana da farin jinni da mutanne? |  |  |  |  |  |  |  |
| _5 | Na ji saukin Magana da mutanne? |  |  |  |  |  |  |  |
| _6 | Na manta da domuwanka? |  |  |  |  |  |  |  |
| _7 | Na aka yi abu wanda baza ji dadi ba bayan? |  |  |  |  |  |  |  |
| _8 | Na ji dadin ayukan jamai ? |  |  |  |  |  |  |  |
| _9 | Na ji dadin jamai? |  |  |  |  |  |  |  |
| _10 | Na shiga damuwa da yansanda? |  |  |  |  |  |  |  |
| _11 | Na ji dadi korai? |  |  |  |  |  |  |  |
| _12 | Na ji rashinlafiya |  |  |  |  |  |  |  |
| _13 | Ban tuna da abubuwan ba (zaki ji tas)? |  |  |  |  |  |  |  |

**[This question is for Past Drinkers (Drank alcohol in the past but not in the past 12 months). Never drinkers go to MOTV.AGST]**

***[Wannan tambayoyin don wanda sun taba shan giya (Ka sha giya amma baka sha ba watanna 12 do sun wuce). Wanda basu sha ba su wuce MOTV.AGST]***

…MOTV.FOR Mutanne suna da dalilai da ya sa suna shan giya. Lokachin da kana sha, yaya zakace da wadanna amfani gare ka? Zaka iya ce ne da amfani sosai, da amfani, ba amfani sosai, ba amfani?

|  |  | 1 – DA AMFANI SOSAI | 2 –DA AMFANI | 3 – BA AMFANI SOSAI | 4 – BA AMFANI | 98 – BAN SANI BA | 99 – NA KI |
| --- | --- | --- | --- | --- | --- | --- | --- |
| _1 | Zaka yi faraa da abokane? |  |  |  |  |  |  |
| _2 | Domin wasu ke shan giya? |  |  |  |  |  |  |
| _3 | Zai kara dadin abinci? |  |  |  |  |  |  |
| _4 | Don magini? |  |  |  |  |  |  |
| _5 | Don jin dadi? |  |  |  |  |  |  |
| _6 | Don taimakon hutawa? |  |  |  |  |  |  |
| _7 | Don manta da damuwa? |  |  |  |  |  |  |
| _8 | Don ya hana kunya? |  |  |  |  |  |  |
| _9 | Don buki? |  |  |  |  |  |  |
| _10 | Don dadi a baki? |  |  |  |  |  |  |
| _11 | Don kishin? |  |  |  |  |  |  |

**[This question is for both Past Drinkers and Never Drinkers]**

***[Wannan tamboyi don wadan sun bar shan giya do wanda basu taba sha ba]***

…MOTV.AGST Mutane da dalilai dabam-dabam da ya sa sun rage shaye-shaye ko barin shaye-shaye gabakindayanta. Yaya zaka ce da wadannan dalilai rareka? Zaka iya ce da amfani sosai, da amfani, ba amfani sosai, ko ba amfani?

|  |  | 1 – DA AMFANI SOSAI | 2 –DA AMFANI | 3 – BA AMFANI SOSAI | 4 – BA AMFANI | 98 – BAN SANI BA | 99 – NA KI |
| --- | --- | --- | --- | --- | --- | --- | --- |
| _1 | Domin ina da chiki, ko kuwa ina so in yi chiki? |  |  |  |  |  |  |
| _2 | Domin dadin a baki? |  |  |  |  |  |  |
| _3 | Domin baka so yanda yake maida kai? |  |  |  |  |  |  |
| _4 | Domin ka ga barnan giya? |  |  |  |  |  |  |
| _5 | Domin ka ga yanda giya ke wasu? |  |  |  |  |  |  |
| _6 | Domin shan giya zai sa matsala a aikin ka ko maranta? |  |  |  |  |  |  |
| _7 | Domin giya na da tsada ko kuwa ke batarda kudi? |  |  |  |  |  |  |
| _8 | Domin addini? |  |  |  |  |  |  |
| _9 | Domin baka girma da shan giya ba? |  |  |  |  |  |  |
| _10 | Domin ka ji barnan giya ko baka so ka zama mashayi? |  |  |  |  |  |  |
| _11 | Domin ina yaro harzanzu? |  |  |  |  |  |  |
| _12 | Domin abokanenka do iyalinka basaso? |  |  |  |  |  |  |
| _13 | Domin bana son giya, ko kana shan magani, ko don rashin lafiya? |  |  |  |  |  |  |
| _14 | Don rashin lafiya? |  |  |  |  |  |  |
| _15 | Don baka son? |  |  |  |  |  |  |

# MODULE 17: Own and Others’ Perception of Alcohol and other Substances (PCPN)

# Dalilanka and na wasu akan kayan shaye-shaye

...GEN Ka gaya mini ko ka yarda sosai, ka yarda, baka yarda ko ki ba, ka ki, ka ki sosai akan wayanna bayyanai.

...GEN_1 Shan giya ke kautata raayuwa?

1 – NA YARDA SOSAI

2 – NA YARDA

3 – BAN YARDA KO KI BA

4 – NA KI

5 – NA KI SO SAI

98 – BAN SANI BA

99 – NA KI ANSA

...GEN_2 Shan giya da wasu yanya ne na nuna abokantaka?

1 – NA YARDA SOSAI

2 – NA YARDA

3 – BAN YARDA KO KI BA

4 – NA KI

5 – NA KI SO SAI

98 – BAN SANI BA

99 – NA KI ANSA

...GEN_3 Ba wani abu mai kyau da shaye-shaye?

1 – NA YARDA SOSAI

2 – NA YARDA

3 – BAN YARDA KO KI BA

4 – NA KI

5 – NA KI SO SAI

98 – BAN SANI BA

99 – NA KI ANSA

*…SITS Biye, zan bayyana maka yanayi da mutane a wasu lokaci su kan samu kansu a chiki. A kowani yaniyi, ka gayamini yaya wanda yaki a wannan yanayin zai ji sailin da zai sha giya – 0 shaye-shaye, wasu zasu sha amma ba zasu ji komai ba (shaye-shaye 1 ko 2), ya isa ya ji shi amma ba zai yi maiye ba, maye wasu lokutan daidai ne, ko yin maye daidai ne? (Show card)*

|  |  | 1 – 0 SHAYE-SHAYE | 2 – WASU ZASU SHA AMMA BA ZASU JI KOMAI BA (SHAYE-SHAYE 1 KO 2) | 3 – YA ISA YA JISHI AMMA BA ZAI YI MAIYE BA | 4 – MAYE WASU LOKUTAN DAIDAI NE | 5 – YIN MAYE DAIDAI NE | 98 – BAN SANI BA | 99 – NAKI |
| --- | --- | --- | --- | --- | --- | --- | --- | --- |
| _2 | A mazaman mama, samun zarafi tare the yara |  |  |  |  |  |  |  |
| _3 | A mazaman baba, samun zarafi tare the yara |  |  |  |  |  |  |  |
| _6 | A mazaman mutum a shaye-shaye da abokane |  |  |  |  |  |  |  |
| _7 | A mazaman mace a shaye-shaye da abokane |  |  |  |  |  |  |  |
| _8 | A mazaman mace tare da maaikatan wurin aikin ta |  |  |  |  |  |  |  |
| _9 | A mazaman miji tare da maaikatan wurin aikin sa |  |  |  |  |  |  |  |
| _12 | A mazaman miji wanda ke chin abinchin yamma a gidansa tare da na biyun sa |  |  |  |  |  |  |  |
| _13 | A mazaman mace wanda ke chin abinchin yamma a gidanta tare da na biyun ta |  |  |  |  |  |  |  |

# MODULE 20: Don yara da Samarai

## 20.3 Zamani ga Mazantaka

EMAD.PERC Ka gaya mini ko ka yarda so sai, ka yarda, baka yarda ko ki ba, ka ki ko ka ki so sai akan wadannan maganganu. *(Show card)*

...EMAD.PERC_1 Na isa mazantaka.

1 – NA YARDA SOSAI

2 – NA YARDA

3 – BAN YARDA KO KI BA

4 – NA KI

5 – NA KI SO SAI

98 – BAN SANI BA

99 – NA KI ANSA

...EMAD.PERC_2 Bana bukachi kudi daga iyaye na ba.

1 – NA YARDA SOSAI

2 – NA YARDA

3 – BAN YARDA KO KI BA

4 – NA KI

5 – NA KI SO SAI

98 – BAN SANI BA

99 – NA KI ANSA

...EMAD.PERC_3 Bana bukachi iyaye ne akan damuwata.

1 – NA YARDA SOSAI

2 – NA YARDA

3 – BAN YARDA KO KI BA

4 – NA KI

5 – NA KI SO SAI

98 – BAN SANI BA

99 – NA KI ANSA

**MODULE 21: Respondent Engagement, Recruiting and Screening (RCRT)**

Yansu mu nkusa gaman tamboyo yi mu. A koi wayansu tambaya kuma.

…ENG1 A mataki daya zua goma, za aiya gayama ka na so wanna binjike da mukayi.?

__ __

98 – BAN SANI BA

99 – NA KI ANSA

…ENG2 A mataki daya zua goma, za aiya gayama ka ji dadi wanna binjike da mukayi.?

__ __

98 – BAN SANI BA

99 – NA KI ANSA

Yanzu muna kusa the karshe a wannan tambayoyin. Kamar yadda na fada daga fari, muhimmanci korai mu yi nasara a wannan binchike-binchiken wanda mun iya debo bayyanai daga mutane daban-daban masu halaye, da dabiya, da tunayi sun banbanta – mun gode maka. Muna bida akan mutane masu yawa zasu chigaba a sashi na biyu. Sashi na biyu yana bukata ka ansa tambayoyin online. Ba kowa ne ya yarda ya chigaba ba da zaa tuntube shi ba – amma muna so mu samu bayyanu daga mutane dabandaban.

...PART Kana so a saka a sashi na biyu?

1 – A – INA SO IN ZAMA CHIKIN SASHI NA BIYU AKAN BINCHIKE-BINCHIKEN NAN

2 – AA – BANA NA SO IN ZAMA CHIKIN SASHI NA BIYU AKAN BINCHIKE-BINCHIKEN NAN

(JE ZUWA/Skip …SCRN)

98 – BAN SANI BA

99 – NA KI

 …PART.REF Ka gaya mana dalilin da ya sa baka so ka ansa tamboyin nan.

______________________ (open-ended response)

98 – BAN SANI BA

99 – NA KI

…SCRN... Kafin mu gama, I na so in tambaye ka tamboyi na karshe:

…SCRN_STDN In ka mani cewa ‘yan makaranta’ sune mutanen da yanzu suke karatu or koyaswa, yaya zaka yi bayani a kanka..

1 –NI DALIBI NE

2 – NI DALIBI NE NA YAMMA

3 – NI BA DALIBI BANE (Skip to …SCREEN.INT)

98 – BAN SANI BA

99 – NA KI

…SCRN.EDUC Yaya zaka bayyana karatu ko koyaswa da ka ke chiki yanzu:

1 – GABA DA SECONDARY (e.g. high school, secondary school)

2 – COLLEJIN ILIMI (e.g. community college, technical college)

3 – JAMIA (e.g. university)

4 – WASU (please specify) _______________________________________

98 – BAN SANI BA

99 – NA KI

…SCRN.INT Yaya kake amfani da internet?

1 – KULLUM

2– DAYA A MAKO

3 – DAYA A WATA

4 – KASA DA DAYA A WATA

5 -- BABU

98 – BAN SANI BA

99 – NA KI

…SCRN.CHCK Yaya kake duba email naka?

1 – KULLUM

2– DAYA A MAKO

3 – DAYA A WATA

4 – KASA DA DAYA A WATA

5 – BABU

6 – BANI DA EMAIL

98 – BAN SANI BA

99 – NA KI

(IF PART=2 (DOES NOT WANT TO PARTICIPATE) END INTERVIEW

(IF SCRN.INT = 5 OR 6 **AND** SCRN.CHCK = 5 OR 6, THEN END.)

(IF SCRN.INT = 1-4 **AND** SCRN.CHCK = 5 OR 6, THEN GO TO PART.CNTC.OTH)

(IF SCRN.INT = 1-4 **AND** SCRN.CHCK = 1-4, CONTINUE)

…PART.EMAIL Za mu tuntubi mutanen da zasu yi sashi na biyu ta wurin email nasu, kana da email da zamu iya tuntubanka dashi?

1 – A, EMAIL NAWA SHINE: ______________________________________________

***(Interviewer: Verify email address by asking respondent to repeat address.)***

2 – AA, BANI DA EMAIL ADDRESS

98 – BAN SANI BA

99 – NA KI

…PART.CNTC.OTH Za ka iya bamu number wayan ka alhali in email ya ki?

1 – A, LAMBA WAYA NA SHINE: __________________________________

***(Interviewer: Verify by asking respondent to repeat.)***

*2 – A, ADDRESS na ne: ____________________________________________*

***(Interviewer: Verify by asking respondent to repeat.)***

3 – AA, BANI DA LAMBAN WAYA

98 – BAN SANI BA

99 – NA KI

[MUN GODE KARSHEN KENAN] (THANK YOU AND END)
