# Supplementary material for: Context and culture associated with alcohol use amongst youth in major urban cities: A cross-country population based survey
Source: PLoS One. 2017 Nov 20;12(11):e0187812. doi: 10.1371/journal.pone.0187812 (PMC5695777; doi:10.1371/journal.pone.0187812)
Supplement: S4 Questionnaire — (DOC) [file pone.0187812.s004.doc]

**LTACC Questionnaire (Ibo Language)**

Final Draft

Contents

[Module A: Household Identification and Census, Consent, and Administrative Variables (ADMN) 3](#__RefHeading___Toc394931724)

[MODULE 1: Demographics](#__RefHeading___Toc394931725) 6

[MODULE 9: Health Behaviors, Including Drinker Status (HTBX)](#__RefHeading___Toc394931726) 10

[MODULE 10: Alcohol Consumption, Last 12 Months (CONS)](#__RefHeading___Toc394931727) 11

[10.1 General Alcohol Consumption 11](#__RefHeading___Toc394931728)

[10.2 Beverage-Specific Quantity/Frequency](#__RefHeading___Toc394931729) 12

[10.3 Experience of Drunkenness/Intoxication](#__RefHeading___Toc394931730) 14

[10.4 Drinking Context](#__RefHeading___Toc394931731) 14

[MODULE 15: Motivations For/Against, and Effects of, Drinking – DRINKERS ONLY (MTCD)](#__RefHeading___Toc394931732) 16

[MODULE 16: Motivations For/Against, and Past Outcomes of, Drinking – NON-DRINKERS ONLY (MTND)](#__RefHeading___Toc394931733) 19

[MODULE 17: Own and Others’ Perceptions of Alcohol and Other Substances (PCPN)](#__RefHeading___Toc394931734) 22

[MODULE 20: Adolescents and Young Adults (ADYA) 24](#__RefHeading___Toc394931735)

[20.3 Emerging Adulthood](#__RefHeading___Toc394931736) 24

[MODULE 21: Respondent Engagement, Recruiting and Screening (RCRT) 25](#__RefHeading___Toc394931737)

# Module A: Household Identification and Census, Consent, and Administrative Variables (ADMN)

***A.0 Household Identification, Date*** *(Complete before approaching the household/respondent)*

LEV1… City/metropolitan area:

__ __ (see codebook)

LEV2… [LGA ]:

__ __ (see codebook)

LEV3… [Wards ]:

__ __ (see codebook)

LEV4… [Neighbourhood/settlement ]:

__ __ (see codebook)

…LEV5 [Street ]:

__ __ (see codebook)

…LEV6 [Household ]:

__ __ (see codebook)

…INTID __ __ __ __

…DATE __ __ (dd) __ __ (mm) __ __ __ __ (yyyy)

***A.1 Household and Respondent Disposition*** *(Complete after contact with the household/respondent)*

…HDIS Household disposition:

__ __ (see codebook)

…RDIS: Respondent disposition:

__ __ (see codebook)

…RDIS_TXT Reason for early termination of interview, if applicable (i.e., ADMN.RDIS = 25 or 26)

________________________________________________________________________

________________________________________________________________________

***A.2 Household Introduction, Census, Determination of Respondent Eligibility***

...INTR Daalu nu o, aha m bu (name). Oru m bu onye na aju ajuju. Ana m aru Dr A.B Makanujola orunke etiti mba nwa nile nke gbasara nmanya, nke di International Centre for Alcohol Policies, Washinton DC, USA. Achorom imata na ndi agbata obi gi ona anubiga mmanya oke, ma obu na ha ona ahuo mmanya obere obere. Achorom iju ndi agbata obi gi ajuju maka mmuta.

*After obtaining consent:*

Imela nke oma. Ebe ibi ugbugbua gwam afor ndi nwoke maa ndi nwayin, ntakiri, agadi, maa ndi nwayin, ntakiri, agadi, maa ndi obere na eba ha ibi?

*Complete Columns 1 and 2 of the matrix below by entering ages and circling the correct gender. For every person listed who is between 18 and 34 years of age, ask whether s/he has lived in [country-specific city/metropolitan area] for at least 6 months and complete Column 3 as appropriate.*

Household Census

|  | **Afor** | **Ibu nwoke/nwanyi** | **Ebee ka ibi onwa isii garaga?** |  |  | **Afor** | **Ibu nwoke/nwanyi** | **Ebee ka ibi onwa isii garaga?** |
| --- | --- | --- | --- | --- | --- | --- | --- | --- |
| **Oye mbu** |  | Nwoke/nwanyi | Eee/Mba |  | **Oye n ke asa** |  | Nwoke/nwanyi | Eee/Mba |
| **Oyen ke abua** |  | Nwoke/nwanyi | Eee/Mba |  | **Oye n ke asato** |  | Nwoke/nwanyi | Eee/Mba |
| **Oye n ke ato** |  | Nwoke/nwanyi | Eee/Mba |  | **Oye n ke tolu** |  | Nwoke/nwanyi | Eee/Mba |
| **Oye n ke ano** |  | Nwoke/nwanyi | Eee/Mba |  | **Oye n ke iri** |  | Nwoke/nwanyi | Eee/Mba |
| **Oye n ke ise** |  | Nwoke/nwanyi | Eee/Mba |  | **Oye n ke iri na nna** |  | Nwoke/nwanyi | Eee/Mba |
| **Oye n ke isii** |  | Nwoke/nwanyi | Eee/Mba |  | **Oye n ke iri na abua** |  | Nwoke/nwanyi | Eee/Mba |

*If no eligible respondent, move to next household. If multiple eligible respondents, select respondent using last birthday method and ask to speak to selected respondent. Skip to A.1 and complete the questions below only after* interviewing (or attempting to interview) the selected respondent.

…LANG2 Kedi asusu eji ajuo gi ajuju*:*

1. Yoruba
2. Hausa
3. Ibo
4. English

…LANG3 *Rate the respondent’s apparent comfort with the interview language, on a scale of 1 to 5 (1 = respondent unable to understand, could not finish interview; 5 = respondent fluent, appeared to understand every question with ease): __*

***A.3 Respondent Introduction and Consent***

...INST1 Ndewo, aha m bu (Dr A.B Makanjuola) A bum onye oka mmuta azuru nkeoma n’ ihe gbasara ijupu ta ajuju dika mpaghara ihe nchoputa agumakwukwo nke ndi (International Centre for Alcohol Policies, USA): A hotara ekele nkowa yi dika nke a ga etinyenaihe agumakwukwo anyi N’ ihe agumakwukwo anyia, odi oke mkpa n’anyi ga enwetagasi echiche obi akparamagwa, obibi ndu ndi mmadu na kwa ihe ha hutarala, gunyekwera ndidi ka gi. Ihe nleba anya N ewe diak (20-30min) I ji meecha ya. Mkpebi gi gbasara isonye m’ ob u esonyeghi n’ihe nchoputa bu m’ o masikwa gi i buru n’enwere ajujun’ akwadohi umegi iru ala, anyi nwekwara ike gafere ya iwu ejighikwa gi ma ikwusi ajuju onu oge o bula

________

Initials of interviewer, indicating

verbal consent of respondent

***A.4 Screening and Administrative Questions***

...INST2 Eke lem gi, tupu ayi amalite, oga a ma sim I jugi ajulu ole na ole I ji kwam ma itu zuru iso kpa ko ri taa nkata na ihe omumu aa.

...BYR Ke du aho a mu ru gi?

__ __ __ __

98 – Amaghi

99 – Ekweghi

*Probe if, based on birth year, respondent may be younger than 18 (born in 1996) or older than 34 (born in 1980). Interview ends if respondent is not in desired age range, or age cannot be determined. [Code respondent as “Ineligible”.]*

…RESI I bi e la na obodo aa ma obu na gburu gburu aa ke mgbe gara aga?

1 – Eee

2 – Mba

98 – Amaghi

99 – Ekweghi

*Interview ends if respondent has not resided in city/metropolitan area for at least the previous 6 months. [Code respondent as “Ineligible”.]*

…LANG1 Ke du I he bu asusu mbu gi?

1. Yoruba
2. Hausa
3. Ibo
4. A susu ndi ozo di iche iche

98– Amaghi

99– Ekweghi

*Interview is conducted in respondent’s primary language, if possible. If this is not possible, or the respondent’s primary language is not known, rate (on previous page) the respondent’s level of comfort with the language in which the interview was conducted upon the conclusion of the interview.*

***A.5 Introduction to Interview***

…INST3 Ka yin ga na ajuju ayin. O buru na oma si gi, ka m bi do a juju m, cheta na ihe obu na I zara m ga a bu ihe nzuzo din a etiti mu na gi, biko, ji ri obi ocha gi ni le zam ajuju aa. Obu run a I nab a ta ha ajuju obuna, ma o bun a I magi I he I ga azam, biko me ka mma ra ka yi we ga na ajuju nke ozo.

…STRT  *Detue oge:*

__ __ : __ __ (HH:MM, 24:00 clock)

# MODULE 1: Demographics

…SEX Detuo ihe o bu site na ihe I hutara, juo ma o buru na o daputa

1 – Nwoke

2 – Nwanyi

98 –Amaghi

99 – Ekweghi

...MAR Kedu ihe ga akowaputa din a nwunye onodu?

1 –Alu

2 –Gbara alukwaghi m ma o bu iche

3 –Isi mkpe

4 – **Alubeghi** (Skip to …HH.AD)

98 –Amaghi (Skip to …HH.AD)

99 –Ekweghi (Skip to …HH.AD)

…MAR_FU Kedu afo (I luru/gbara alukwaghi m ma o bu gbasaa/isi mkpe)

__ __ __ __

98 –Amaghi

99 –Ekwehi

...HH.AD Efinyeghi onwegi, ndi toro ebo ole gaferela afo iri na asato ma o bu kana bi na ezi na ulo a (ogbe ulo a)

__ __ Ndi toro eto *(If 0, skip to DEM.HH.AD_FU2)*

98 – Amaghi

99 –Ekweghi

…HH.AD_FU1 Kedu ndi ha bu? (Select all that apply)

1 –Onye out olulu ma o bu onye mmeko

2 – **Nne na nna ma o bu onye nlekota** (*Skip to DEM.HH.PNT1)*

3 – Ndi out ezi na ulo ndi ozo

4 – Enyi onye obibi ma o bu onye a abughi onye akuku abu ezi na ulo

98 –Amaghi

99 –Ekweghi

…HH.AD_FU2 Kedu afo I hapuru ezi nay lo unu ma bin na nke onwe gi?

__ __ __ __

98 – Amaghi

99 – Ekweghi

...PNT1 Do you have any children? I nwere umu-aka ala o obula?

1 – Eee

2 – Mba *(Skip to DEM.EDU)*

98 –amaghi *(Skip to DEM.EDU)*

99 –Ekweghi *(Skip to DEM.EDU)*

…PNT1_FU Umu aka ole ka I nwere?

______ Umuaka

98 – Amaghi *(Skip to DEM.EDU*

99 –Ekweghi *(Skip to DEM.EDU*

…PNT2 …PNT2_FU

| Nwata | Kedu afo ha gasi? (bido na onye nke pekanchara) | Gi nay a o bi? |
| --- | --- | --- |
| 1 | PNT2_1  YEARS  98 – Amaghi  99 – Ekweghi | PNT2_1_FU  1 – Eee 2–Mba  98 – Amaghi  99 – Ekweghi |
| 2 | PNT2_2  Afo  98 –Amaghi  99 –Ekweghi | PNT2_2_FU  1 – Eee 2- mba  98 –*Amahi*  99 –Ekweghi |
| 3 | PNT2_3  Afo  98 –Amaghi  99 –Ekweghi | PNT2_3_FU  1 – Eee 2- Mba  98 –Amaghi  99 –Ekweghi |
| 4 | PNT2_4  Afo  98 – Amaghi  99 –Ekweghi | PNT2_4_FU  1 – Eee 2- Mba  98 –Amaghi  99 –Ekweghi |
| 5 | PNT2_5  Afo  98 – Amaghi  99 –Ekweghi | PNT2_5_FU  1 – Eee 2- Mba  98 –Amaghi  99 –Ekweghi |
| 6 | PNT2_6  Afo  98 –Amaghi  99 –Ekweghi | PNT2_6_FU  1 – Eee 2- Mba  98 – Amaghi  99 –Ekweghi |
| 7 | PNT2_7  Afo  98 – Amaghi  99 –Ekweghi | PNT2_7_FU  1 – Eee 2- Mba  98 –Amaghi  99 –Ekweghi |
| 8 | PNT2_8  Afo  98 – Amaghi  99 –Ekweghi | PNT2_8_FU  1 – Eee 2- Mba  98 – Amaghi  99 –Ekweghi |

...EDU Kedu ogo agumakwukwo kachasi elu I mezurula.

1. E je gim akwukwo, a gu chagim akwukwo mbu, ma obu a gu charam akwukwo mbu
2. I gu cha akwukwo mbu, ma obu gu cha akwukwo mbu (6 – 12 years)
3. A guchagi akwukwo mahadum, I ji de asam bodo nke nka na uzu agba nke mbu, college nke mmuta, akwukwo nke itozu oke iburu oye nku zi, na nosu (12-15yrs)
4. I gu cha agwukwo di elu (di ka) I bu oye gu chara mahadum, nkanauzu (HND) nwata akwukwo mahadum ndi omini (16+ of education)

98 –Amaghi

99 –Ekweghi

…STDT Ugbua, I bu nwata akwukwo?

1 – Eee *(Skip to DEM.EMPL)*

2 –Mba

98 –Amaghi

99 –Ekweghi

…STDT_GR Kedu afo I guchara ma o bu imechara ihe gbasara agumakwukwo gi?

__ __ __ __

98 –Amaghi

99 –Ekweghi

...EMPL Kedu ka I ga esi kowaa onodu inweta oru gi?

1 –O ru oge zuru ezu (40 hours or more per week) *(Skip toEMPL_FU3, self employed)*

2 –Oru okara oge(fewer than 40 hours per week) *(Skip to EMPL_FU3, self employed)*

3 –Enwghi ulo

4 –Onye ndozi ulo

5 –Nkwuru na enweghi ike iru oru *(Skip to DEM.OCC)*

6 – Ndi ozo_________________ *(Skip to EMPL.FU1)*

98 – Amaghi

99 – Ekweghi

Draft (20 May 2014) – obughi maka okike

...EMPL_FU1 Ugbu a, I’ na acho oru a ga akwu gi ugwo

1 –Eee

2 –Mba *(Skip to EMPL_FU4.YR)*

98 – Amaghi *(Skip to EMPL_FU4.YR)*

99 – Ekweghi *(Skip to EMPL_FU4.YR)*

...EMPL_FU2 Nke o ga abu oru mbu a ga akwi gi ugwo?

1 –Eee *(Skip to DEM.OCC)*

2 –Mba *(Skip to EMPL_FU4)*

98 –Amaghi *(Skip to EMPL_FU4.YR)*

99 –Ekweghi *(Skip to EMPL_FU4.YR)*

...EMPL_FU3 Nke a o bu oru mbu a ga akwu gi ugwo?

1 –Eee

2 –Mba

98 –Amaghi

99 –Ekweghi

…EMPL_FU4 What year did you start working for pay Kedu afo I bidoro oru mpata egoi?

__ __ __ __

97 –Anubeghi m oru a na akwu ugwo mbu

98 –Amaghi

99 –Ekweghi

…OCC Kedu ihe I na ani ma o bu aka oru gi o buru na I naghi aru oru ugbu a, mana I ruru oru n’ oge gra aga, biko gosiputa oru mee e were gin a ya. O buru na I ruola oru buru ibu, biko hota nke tinyere oge gin a ya karichaa:

| **1** | Ochi agha |
| --- | --- |
| **2** | Ome iwu, oye isi no ru oyibo/ o ye nchi ko ta |
| **3** | Oka mmuta |
| **4** | Ncana uzu, na ndi na eso te ndi oka mmuta |
| **5** | O de akwukwo |
| **6** | Ndi na eresi mma du a hia/ebe ana azu ahia/ahia/ ndi ore ahia |
| **7** | Ndi nwere mmuta na oru ugbo/oku azu |
| **8** | Uzu na ihe gba sa ra ndi oru |
| **9** | Akara nkwa eji a ko ugbo/ Ndi na ayaya |
| **10** | Mmalite oru mbu |
| **97** | E we tu be hem nolu |
| **98** | Amaghi |
| **99** | Ekweghi |

97 –Arubeghi m oru a na akwu ugwo mbu

98 –Amaghi

99 –Ekweghi

…REL Kedu okpukpe I na esonye?

1 – Ezie/Ekweghi na chineke AGNOSTIC/ATHEIST

2 – Buddha BUDDHIST

3 – Ndi nke chineke CHRISTIAN

4 – HINDU Hindu

5 – Ndi Juu JEWISH

6 – Alakuba MUSLIM

7 – Okpukpere chi ozo SIKH

8 – Ndi sikh ANOTHER RELIGION:

98 – Amaghi DON’T KNOW

99 – Ekweghi REFUSED

…RACE Kedu ogburu gi

1. Yoruba

2. Hausa

3. Ibo

4. A susu ndi ozo di iche iche

98 –Amaghi

99 –Ekweghi

# MODULE 9: Health Behaviors, Including Drinker Status (HTBX)

Usoro ahu ike tinyere onodu inu mmanya

…ALC.EVER Inutula mmanya na-aba n’anya dika akpuru achia? Biko etinyekwala oge inyturu nke mmadu ma o bu onye ozo na nau

1 –Eee

2 –Mba *(Skip to MTND.MOTV.AGST- for non-drinkers)*

98 –Amaghi

99 –Ekweghi

…ALC.STRT Afo ole ka I di oge I nuru mmanya n’ aba n’anya mbu? Biko, ehnyekwala oge I mesara ya onu out ugboro ma o bu ugboro abuo site na mmanya.

__ __ YEARS OLD

98 –Amaghi

99 –Ekweghi

…ALC.DRUK Afo ole ka I di mgbe mbu mmanya gburu gi

__ __ YEARS OLD

97 –Anubeghi m mmanya, nubiga ya oke

98 –Amaghi

99 –Ekwehi

…ALC.HVY.STRT Anyi nwere mmasi n’afo ole i di na mbido nakwa na ngwa na ngwucha ndu gi oge I na anu mmanya nke ukwuu. Ihe diko afo ole ka i nwere ike i kwu na idi oge inu mmanya nke ukwuu bidoro

__ __ YEARS OLD

98 –Amaghi

99 –Ekweghi

…ALC.HVY.STOP Ihe dika afo ole ka I di oge o gabigara?

__ __ YEARS OLD

97 –O kwubigeghi

98 –Amaghi

99 –Enweghi

# MODULE 10: Alcohol Consumption, Last 12 Months (CONS)

## 10.1 General Alcohol Consumption

…GEN.FQ N’ onwa iri na abuo gara aga, otutu mgbe ole ka I nuni biaa, mmanya, mmanya oku (dika vodka, gin, whisky, brandy) ma o bu mmanya o bula na aba n’anya o bubadi ebe o pere mpe? *(Show card)*

1 –Ubochi o bula

2 –Ugboro ise ruo isi ni izu uka

3 –Ugboro ato ruo ano n’izu uka

4 –Otu ugboro ruo gbuo n’ izu uka

5 –Ugboro abuo ruo afo n’ onwa

6 –Otu ugboro n’ onwa

7 –Ugboro isii ruo ugoro iri na otu n’onwa in na abuo gara oga

8 –Ugboro abuo nlo ugboo ise n’ onwa iri na abuo gara aga

9 –Otu ugboro n’ onwa in na abuo gara aga

10 – Ariughi m mmanya n’ aba n’ anya n’ onwa *(Skip to MTND.EFCT)*

98 –Amaghi

99 –Enweghi

…GEN.QY N’ onwa in na abuo gara aga, mmanya ni aba na-anya ole ka I kuru n’ olu ubochi I nuru mmanya n’ aba na-anya? Na out mmanya, ihe o pytara bu (mmanya obodo gi na – agunye na mmanya ruru ogo).

*(Show card and graphic illustration)*

1 –mmanya iri abuo na ise ma o bu kana

2 –mmanya iri na otoolu ruo iri abuo na ano

3 –mmanya iri na isii ruo iri na asato

4 –mmanya iri na ubuo ruo iri na ise

5 –mmanya iri na otu

6 –mmanya Asaa ruo asto

7 –mmanya ise ruo isii

8 –mmanya ato wo ano

9 –mmanya abuo

10 –Out mmanya

11 –Opeka mpe out mmanya furu eju *(If* ***both*** *CONS.GEN.FQ = 9 and GEN.QY=11, skip to*

*MTND.EFCT)*

98 –Amaghi

99 –Enweghi

…GEN.MST.QY N’ime onwa iri na abuo yara aga, gini bu ogo mmanya na- abu n’ anya I furu n’etiti awa iri abuo na-ano?

*(Show card)*

1 –Mmanya iri ato na isii ma obu kana

2 –Mmanya iri abuo n’ ato ruo iri ato na ise

3 –Mmanya iri na itoolu ruo iri abuo na ano

4 –Mmanya iri na isi ruo iri na asato

5 –Mmanya iri na abuoitoolu ruo iri na out

6 –Mmanya itoolu ruo iri na o tu

7 –Mmanya assa ruo asato

8 –Mmanya ise ruo isii

9 –mmanya ato wo ano

10 –Mmanya abuo

11 –Out mmanya

12- Ihe na enighi out mmanya juru eju

98 –Amaghi

99 –Ekweghi

…GEN.MST.FQ N’ime onwa iri na abuo gara oga [answer from CONS.GEN.MST.QY], kwamgbe kwamgbe ole ka I riru mmanya n’ime awa iri abuo n’ano? *(Show card)*

1 –Ubochi o bula

2 –Ugboro ise ruo isii n’izu uka

3 –Ugboro ato ruo ano n’ izu uka

4 –Out ugboro ruo abuo n’izu uka

5 –Ugboro abuo kuo ato n’onwa

6 –Otu ugboro n’ onwa

7 –Ugboro isii ruo iri na out n’onwa iri na abuo gara aga

8 –Ugboro abuo ruo ugboro ise n’ onwa iri na abuo gara aga

9 –Otu ugboro n’onwa iri na abuo gara aga

98 –Amaghi

99 –Ekweghi

## 10.2 Beverage-Specific Quantity/Frequency

Ugbu a, o ga amasi m ju gi kedu kwamgbe kwamgbe I furu otu udi mmanya n’egbu egbu n’ime onwa iri na abuo gara aga

**[30]**…BSFQ.BR.FQ Kwambe kwamgbe ole ka I furu mmanya na-aba na-anya n’ ime onwa iri na abuo gara aga? *(Show card)*

1 –Ubochi obula

2 –Ugboro ise ruo isi n’ime out izu uta

3 –Ugboro ato ruo ano n’ ime out izu uka

4 –Otu ugboro ruo abuo n’ime out izu uta

5 –Ugboro abuo ruo ato n’onwa

6 –Otu ugboro n’onwa

7 –Ugboro iri na-owi n’ onwa iri na abuo gara aga

8 –Ugboro abuo nio ugboro ise n’onwa iri na abuo gara aga

9 –Otu ugboro n’ onwa iri na abuo gara aga

10 –Enwebeghi n’ime onwa iri n’abuo gara aga *(Skip to CONS.BSFQ.WN.FQ)*

98 –Amaghi

99 –Ekweghi

…BSFQ.BR.QY Mana n’ime otu ubochi I na anu mmanya n’aba n’ anya, o dika ole k’o ina anu

__ __ *(Show card for standard drink)*

98 –Amaghi

99 –Ekweghi

…BSFQ.WN.FQ Kwamgbe kwamgbe ole ka I furu mmanya n’ime onwa iri na abuo gara aga? *(Show card)*

1 –Ubochi obula

2 –Ugboro ise wo isu n’ otu izu uka

3 –Ugboro afo ruo ano n’ out izu uka

4 –Otu ugboro ruo abuo n’ otu izu uka

5 –Ugboro abuo ruo ugboro ato n’ime otu onwa

6 –Otu ugboro n’ onwa

7 –Ugboro isii ruo ugboro iri-na-otu n’ ime onwa

8 –Ugboro abuo ruo ise n’ onwa iri na abuo gara aga

9 –Otu ugboro n’ ime onwa iri-na-abuo

10 –Enwebegbi n’ ime onwa iri n’abuo gara aga *(Skip to BSFQ.SP.FQ)*

98 –Amaghi

99 –Ekweghi

…BSFQ.WN.QY Mana n’ ime otu ubochi i na anu biaa, o dika ole k inu anu?------

__ __ *(Show card for standard drink)*

98 –Amaghi

99 –Ekweghi

…BSFQ.SP.FQ Kwamgbe kwamgbe ole k’ ifiuru mmanya oku n’ime onwa iri na abuo gara aga? *(Show card)*

1 –Ubochi obula

2 –Ugboro ise wo isu n’ otu izu uka

3 –Ugboro afo ruo ano n’ out izu uka

4 –Otu ugboro ruo abuo n’ otu izu uka

5 –Ugboro abuo ruo ugboro ato n’ime otu onwa

6 –Otu ugboro n’ onwa

7 –Ugboro isii ruo ugboro iri-na-otu n’ ime onwa

8 –Ugboro abuo ruo ise n’ onwa iri na abuo gara aga

9 –Otu ugboro n’ ime onwa iri-na-abuo

10 –Enwebegbi n’ ime onwa iri n’abuo gara aga *(Skip to CONS.BSFQ.OTR.FQ)*

98 –Amaghi

99 –Ekweghi

…BSFQ.SP.QY Mbochi inu ru ogogoro o fu ma, ogogoro o le ka I na a ghu ma?

__ __ *(Show card for standard drink)*

98 – Amaghi

99 – Ekweghi

…BSFQ.OTR.FQ U gboro one na one ka ina a ghu ngwu elu ke mgbe onwa iri na a buo ngara aga

*(Show card)*

1 –Ubochi obula

2 –Ugboro ise wo isu n’ otu izu uka

3 –Ugboro afo ruo ano n’ out izu uka

4 –Otu ugboro ruo abuo n’ otu izu uka

5 –Ugboro abuo ruo ugboro ato n’ime otu onwa

6 –Otu ugboro n’ onwa

7 –Ugboro isii ruo ugboro iri-na-otu n’ ime onwa

8 –Ugboro abuo ruo ise n’ onwa iri na abuo gara aga

9 –Otu ugboro n’ ime onwa iri-na-abuo

10 –Enwebegbi n’ ime onwa iri n’abuo gara aga *(Skip to DRUK.FQ)*

98 –Amaghi

99 –Ekweghi

…BSFQ.OTR.QY Mbochi inu ru mmaya elu o fu ma, nkwu e lu ole ka I na anu?

__ __ *(Show card for standard drink)*

98 – Amaghi

99 – Ekweghi

## 10.3 Experience of Drunkenness/Intoxication

…DRUK.FQ Ugboro ole n’ime onwa iri-na abuo gara aga, k’ifiuru mmanya nubiga ya oke nke mere k’khoputa na ukwu yi n’ ama jijiji ma o bu ahumauzo gi n’ agba itiri ma o bu kwa ekwunima uka gi a buru nke amaghi aghota s

*(Show card)*

1 –Ubochi obula

2 –Ugboro ise wo isu n’ otu izu uka

3 –Ugboro afo ruo ano n’ out izu uka

4 –Otu ugboro ruo abuo n’ otu izu uka

5 –Ugboro abuo ruo ugboro ato n’ime otu onwa

6 –Otu ugboro n’ onwa

7 –Ugboro isii ruo ugboro iri-na-otu n’ ime onwa

8 –Ugboro abuo ruo ise n’ onwa iri na abuo gara aga

9 –Otu ugboro n’ ime onwa iri-na-abuo

10 –Enwebegbi n’ ime onwa iri n’abuo gara aga *(Skip to CONS.CXT1)*

98 –Amaghi

99 –Ekweghi

…DRUK.NM O dika mmanya ole ka I ga anu tupu mmanya egbuwe gi? (1 drink is *(Show card for standard drink)*.

__ __ DRINKS

98 –Amaghi

99 –Ekweghi

## 10.4 Drinking Context

Ugbua, a ga m aju gi ugboro ole I na anu mmanya n’ aba n’anya na onodu kponkwem

|  | N’ime onwa iri na abuo gara aga, otutu mgbe ole ka I nurula mmanya na-aba n’anya. | 1-Ubochi obula ma obu tuo nke nta ka oruoo otu ubauchi | 2 - opekaria mpe ofu ugboro n? izu uka | 3 - opekaria mpe, ofu ugboro n’onwa mana pekaria ofu ugboro n’izu | 4.opekaria mpe ofu ugboro n’me onwa iri na abuo gara uga mana pekaria mfe out ugboro n’onwa | 5 - enewbeghi n’ime onwa iri na abuo gara aga | 98 – amaghi | 99 - enweghi |  | *Na ole ka I na anukan?*  *(Skip if preceding answer is 5, 98, or 99)* | | |
| --- | --- | --- | --- | --- | --- | --- | --- | --- | --- | --- | --- | --- |
| …CXT1 | Oge I puru ka I rie nri mgbede n’ulo onri na onunu? |  |  |  |  |  |  |  |  | 1.NM |  | |
|  |  |  |  |  |  |  |  |  |  |  | 98 | 99 |
| …CXT2 | Oge I puru ka I rie nri ehibie n’ ulo oriri na onunu? |  |  |  |  |  |  |  |  | 2.NM |  | |
|  |  |  |  |  |  |  |  |  |  |  | 98 | 99 |
| …CXT3 | Na ulo ebe a na anu |  |  |  |  |  |  |  |  | 3.NM |  | |
|  |  |  |  |  |  |  |  |  |  |  | 98 | 99 |
| …CXT4 | Anokoo n’ulo onye ozo nke gunyere ikpori ndu n’okwa di elu |  |  |  |  |  |  |  |  | 4.NM |  | |
|  |  |  |  |  |  |  |  |  |  |  | 98 | 99 |
| …CXT5 | I no nani gi oge mgbede n’ulo gi? |  |  |  |  |  |  |  |  | 5.NM |  | |
|  |  |  |  |  |  |  |  |  |  |  | 98 | 99 |
| …CXT6 | Ndi enyi chotara gin’ ulo gi? |  |  |  |  |  |  |  |  | 6.NM |  | |
|  |  |  |  |  |  |  |  |  |  |  | 98 | 99 |
| …CXT7 | Inoko onodu gin a ndi enyi gin a ebe oha na eze no, dika ebe ogbako, ikporo uzo, ma o bu ebe ugbo ala n’ ano |  |  |  |  |  |  |  |  | 7.NM |  | |
|  |  |  |  |  |  |  |  |  |  |  | 98 | 99 |

…CXT.MEAL N’ime onwa iri na abuo gara aga, odika inu mmanya gi ole daputara oge I na eri nri?

1 –O oge o bula ma o bu o nodu ka o bu oge obula

2 –O Karin okara

3 –Okara

4 –Ihe n’ erughi okara

5 –Odighi ma obu foro nke nta ka o hapu

98 –Amaghi

99 –Ekweghi

…CXT.SELF Ke mgbe owa iri na abua gara aga, mgbe obula I no ru owegi, ugbo ro ole ka inu mmaya gin a a kpa lit e I ga si na…….

1 –O oge o bula ma o bu o nodu ka o bu oge obula

2 –O Karin okara

3 –Okara

4 –Ihe n’ erughi okara

5 –Odighi ma obu foro nke nta ka o hapu

98 –Amaghi

99 –Ekweghi

# MODULE 15: Motivations For/Against, and Effects of, Drinking – DRINKERS ONLY (MTCD)

…EFCT I nu mmanya n’aba n’ anya n’emetuta ndi mmadu n’uzo di iche. O ga-amasi gi I nu mmanya nwere ike ime gi. Mgbe I na-anu mmanya n’aba na-anya, kedu ezickwu ufodu oge o buchaghi eziokmi, o bughi eziokwu o bula?

|  | O bu eziokwu n’a I nuo mmanya…….. | 1 – O dika chasin mkpa | 2 – O di mkpa | 3 – dichaghi mkpa | 4 – O dighi mkpa chaa chaa | 5 – mba mba | 98 – Amaghi m | 99 – Enweghi m |
| --- | --- | --- | --- | --- | --- | --- | --- | --- |
| _1 | I noro nnoo juu |  |  |  |  |  |  |  |
| _2 | I nwere nnoo obi oma |  |  |  |  |  |  |  |
| _3 | I nwere oke esemokwu n’ ebe ndi ozo no? |  |  |  |  |  |  |  |
| _4 | I nwere mmuo enyi na opu pu karia? |  |  |  |  |  |  |  |
| _5 | O din gi mfe I kwugbasara echiche obi gi ma o bu nsogbu gi? |  |  |  |  |  |  |  |
| _6 | I chefuru nsigbu gi? |  |  |  |  |  |  |  |
| _7 | I na-eme ihe o ga-abu emesia, kaa onwegi uta? |  |  |  |  |  |  |  |
| _8 | Mmeko nwoke na nwanyi o na ato giuto karichaa? |  |  |  |  |  |  |  |
| _9 | Ihe gbasara nwoke n’nwanyi o di gi mma? |  |  |  |  |  |  |  |
| _10 | I danyela na nsogbu ndi uwe ojii |  |  |  |  |  |  |  |
| _11 | I nwere otutu ihe anuri? |  |  |  |  |  |  |  |
| _12 | Oria o dara gi |  |  |  |  |  |  |  |
| _13 | I chetaghi ihe mere gi? |  |  |  |  |  |  |  |

**[44]**…MOTV.FOR Ndi mmadu nwere ihe di iche iche kpatara ha jio anu mmanya na-aba n’anya kedu uru iga-enweta na ihe ndia, na nke gi? I ga – asi na odi ezigbo mkpa, o dihi ezigbo mkpa ka o bu o dighimkpa o bula?

|  |  | 1 – O di okemkpa | 2 – O di mkpa | 3 – O dighi oke mkpa | 4 –O dighi mkpa obula | 98 –Amaghi m | 99 –Ekweghi m |
| --- | --- | --- | --- | --- | --- | --- | --- |
| _1 | O na-eme ka I soro na ihe ana-eme ma o bu garagara |  |  |  |  |  |  |
| _2 | O bu maka na ndi ozo n’anu mmanya? |  |  |  |  |  |  |
| _3 | Ona-enyere gi aka I nweta afo ojuju na nu? |  |  |  |  |  |  |
| _4 | O bu maka ahu ike? |  |  |  |  |  |  |
| _5 | O na-eme gi ka idi mma? |  |  |  |  |  |  |
| _6 | o na-eme gi ka I noro nke oma? |  |  |  |  |  |  |
| _7 | O na-eme gi ka ichefuo nsogbu gi? |  |  |  |  |  |  |
| _8 | O na-eme ka ihere puo gi n’anya? |  |  |  |  |  |  |
| _9 | Ime mmemme? |  |  |  |  |  |  |
| _10 | O bu maka ulo ya? |  |  |  |  |  |  |
| _11 | O bu maka akpiri ikpo nku? |  |  |  |  |  |  |

**[45]**…MOTV.AGST Ndi mmadu na enwe he di iche iche mere ha ji nwee ogo ha ga anudebe mmanya ma o bu anughi mmanya ma o bula. Kedu k’o si di mkpa k’I kwuo na che ndi na-eso-nu mere bu mak gi? I ga asi na o dikan charamkpu, o di mkpa, o dichaghi mkpa ma o bun a o dighi mkpa?

|  |  | 1 – O dkikacha siri mkpa | 2 –O di mkpa | 3 – O dichaghi mkpa | 4 –O dighi mkpa o bu la | 98 – Amaghi m | 99 –Enweghi m |
| --- | --- | --- | --- | --- | --- | --- | --- |
| _1 | N’ihi na I dibu ime ma o bun a idi ime ma o bun a I chooro idi ime |  |  |  |  |  |  |
| _2 | N’ ihi uto ya? |  |  |  |  |  |  |
| _3 | N’ ihi na mmetuta o nwwere r’ ebe I no amasighi gi? |  |  |  |  |  |  |
| _4 | N’ ihi na I hula imaahu ojoo mmanya naaba na-anya n’emi? |  |  |  |  |  |  |
| _5 | N’ ihi na onye ozo na anu mmanya emeruola gi ahu? |  |  |  |  |  |  |
| _6 | N’ ihi na inu mmanuya nwere ike imeru oru gi ma o bu agumakwukwo gi? |  |  |  |  |  |  |
| _7 | N’ ihi na I nu mmanya di oke onu mo o bu iwufu ego? |  |  |  |  |  |  |
| _8 | N’ibi okpukpere chigi |  |  |  |  |  |  |
| _9 | N’ihi na a zulitere m dika onye na adighi |  |  |  |  |  |  |
| _10 | N’ ihi na I nwetago nsogbu sitere na I nu mmanya ma o bu na-ujo na-atu gi ka ihapu I bu onye onu mmanya. |  |  |  |  |  |  |
| _11 | N’ ihi na I peta mpe? |  |  |  |  |  |  |
| _12 | N’ ihi na ndi enyi gin u ndi otu ezi n’ ulo anabatahi I nu mmanya |  |  |  |  |  |  |
| _13 | N’ ihi na ahu gi angbtaghi ya, I na anu ogini, ma o bu na maka ogoro ahu ike ndi ozo mere ya? |  |  |  |  |  |  |
| _14 | Usoro ahu ike mere ya? |  |  |  |  |  |  |
| _15 | N’ ihi na inweghi mmasi o bula? |  |  |  |  |  |  |

*All respondents completing this module skip to Module 17.*

# MODULE 16: Motivations For/Against, and Past Outcomes of, Drinking – NON-DRINKERS ONLY (MTND)

***[This question is for Past-Drinkers (Drank alcohol in the past but not in the past 12 months). Never drinkers go to MOTV.AGST]***

…EFCT I nu mmanya na emetuta ndi mmadu n’uzo di iche iche, o ga amasi aniyi I muta ka I nu mmanya siri menita gi. Oge I na-anu mmanya, kedu kaigaesi ekwu na ihe ndi ekwuru bu makagi-Eziokwu oge obula; o na abukan ezichwu, mgbe ufodu o na abu ezi okwu, o nahi abukari ezi okwu, ma o bun a o bughi ezickwu

|  | O bu eziokwu ma I nuo mmanya | 1 –O na-abu eziokw oge obula | 2 – O ra-abu eziokw | 3 – Ona-abu eziokw oge ufodu | 4 – O naghi abukan eziokwu | 5 – O naghi obu eziukwu | 98 –Amaghi m | 99 –Enweghi m |
| --- | --- | --- | --- | --- | --- | --- | --- | --- |
| _1 | I noro nnoo juu? |  |  |  |  |  |  |  |
| _2 | I nwere nnoo obi oma? |  |  |  |  |  |  |  |
| _3 | I nwere oke esemokwu n’ebe nid ozo na? |  |  |  |  |  |  |  |
| _4 | I nwere mmuo enyi na opupu karia |  |  |  |  |  |  |  |
| _5 | I nwere mmasi/o din gi mfe I kwu gbasara echiche obi yi ma o bu nsogbu gi |  |  |  |  |  |  |  |
| _6 | I chefuru nsogbu gi? |  |  |  |  |  |  |  |
| _7 | I n’eme ihe oga-abu emesia, I taa onwe gi uta? |  |  |  |  |  |  |  |
| _8 | Mmeko nwoke na nwanyi, o na ato gi uro karichaa? |  |  |  |  |  |  |  |
| _9 | Ihe gasara nwoke ra nwanyi o di gi mma? |  |  |  |  |  |  |  |
| _10 | I danyere na nsogbu ndi uwe ojii? |  |  |  |  |  |  |  |
| _11 | I nwere otutu ihe ariuri? |  |  |  |  |  |  |  |
| _12 | Oria o daara gi? |  |  |  |  |  |  |  |
| _13 | I chetaghi ihe meregi? |  |  |  |  |  |  |  |

***[This question is for Past-Drinkers (Drank alcohol in the past but not in the past 12 months). Never drinkers go to MOTV.AGST]***

…MOTV.FOR Ndi mmadu n’enwe ihe di iche iche n’akpata ha iru mmanya n’-aba n’anya. Oge I na anubu mmanya kedu uru I g’ asi ihe ndia kpatara iji anu mmanya bara gi? I g’ asi o di gi oke mkpa, o di gi mkpa, ma o bu o dighi gi mkpa, m’ obu o dighi gi mkpa o bula?

|  |  | 1 – Odi oke mkpa | 2 –O di mkpa | 3 – O dichaghi mkpa | 4 – O dighi mkpa o bu la | 98 –Amaghi | 99 –Enweghi |
| --- | --- | --- | --- | --- | --- | --- | --- |
| _1 | I me ka iwusapu aka ma mara k’esi agwa mmadu okwa |  |  |  |  |  |  |
| _2 | N’ ihi na ndi ozo n’ anu |  |  |  |  |  |  |
| _3 | Ona agbakwunye aka ka na too uto? |  |  |  |  |  |  |
| _4 | N’ ihi usoro ahu ike |  |  |  |  |  |  |
| _5 | I me k’idi mma |  |  |  |  |  |  |
| _6 | O na enyere gi aka n’a izu ike |  |  |  |  |  |  |
| _7 | I chefu mkpa gi |  |  |  |  |  |  |
| _8 | O n’ enyere gi aka ikpochapu ihere |  |  |  |  |  |  |
| _9 | I sonye n’ anyri |  |  |  |  |  |  |
| _10 | N’ ihi uto ya? |  |  |  |  |  |  |
| _11 | N’ ihi akpin nku |  |  |  |  |  |  |

***[This question is for both Past-Drinkers and Never Drinkers]***

…MOTV.AGST **Ndi mmadu n’enwu ihe di icheiche n’ akpata ha ji enwe oge ha n’ anudebe mmanya m’ o bu anughi mmanya o bula kedu mkpa I gbasi n’ ihe a na ekwu di gbasara ebe I no I g’asi oke mkpa, o di mkpa, odicheghi mkpa o dighi mkpa o bula**

|  |  | 1 – Odikachasiri mkpa | 2 –O di mkpa | 3 – O dichaghi mkpa | 4 – O dighi mkpa o bu la | 98 –Amaghi | 99 –Ekweghi |
| --- | --- | --- | --- | --- | --- | --- | --- |
| _1 | N’ ihi na I dibu ime ma o bun a I di ime ma o bun a I choro idi ime? |  |  |  |  |  |  |
| _2 | N’ ihi uto ya? |  |  |  |  |  |  |
| _3 | N’ ihi na mmetuta o nwere n’ ebe I no amasighi gi? |  |  |  |  |  |  |
| _4 | N’ ihi na I hula imaatu ojoo mmanya na-aba n’anya n’ eme |  |  |  |  |  |  |
| _5 | N’ ihi n’ onye ozo n’anya n’eme |  |  |  |  |  |  |
| _6 | N’ ihi n’ inu mmanya nwere ike imeru oru gi m’ o bu aguma kwukwo gi? |  |  |  |  |  |  |
| _7 | N’ ihi n’ I nu mmanya di oke onu m’ o bu iwufu ego |  |  |  |  |  |  |
| _8 | N’ ihi okpukperechi gi? |  |  |  |  |  |  |
| _9 | N’ ihi n’azulitere m dka onye n’ adighi anu mmanya |  |  |  |  |  |  |
| _10 | N’ ihi n’ I nwetala nsogbusitere n’ I nu mmanya m’ o bu n’ ujo n’ atu gi k’ I hapu I bu onye o nu mmanya? |  |  |  |  |  |  |
| _11 | N’ ihi n’ I pere mpe? |  |  |  |  |  |  |
| _12 | N’ ihi n’ ndi enyi m ndi out ezi n’ ulo m anabataghi I nu mmanya |  |  |  |  |  |  |
| _13 | N’ ihi n’ a hu gi anabataghi ya/ n’ anu ogwu ma o bu na maka esoro ahu ike ndi ga mere ya |  |  |  |  |  |  |
| _14 | Usere ahu ike mere ya? |  |  |  |  |  |  |
| _15 | N’ ihi n’ inweghi mmasi o bula/ |  |  |  |  |  |  |

# MODULE 17: Own and Others’ Perceptions of Alcohol and Other Substances (PCPN)

...GEN Biko, gwa anyi m’ I kwenyesiri ike, I kwenyere, I choghi I si eee m’ o bu oo, I kwenyeghi, m’ o bu I kwanyeghi sie ike n’ okwu ndi n’ esote  *(Show card)*

...GEN_1 I nu mmanya sin a out uto di n’ ime ndu

1 –Kwenyesiri ike

2 –Kwenyere

3 –Ichoghi ikwenye m’ o bu ekwenyeghi

4 –I kwenyeghi

5 –I kwenyeghisie ike

98 –Amaghi

99 –Ekweghi

...GEN_2 Gina mmadu inuko mmanya bu uzo e si egesi enyi.

1 –Kwenyesiri ike

2 –Kwenyere

3 –Ichoghi ikwenye m’ o bu ekwenyeghi

4 –I kwenyeghi

5 –I kwenyeghisie ike

98 –Amaghi

99 –Ekweghi

...GEN_3 Ihe dim ma e kwesiri I kwu gbasara / nu mmanya

1 –Kwenyesiri ike

2 –Kwenyere

3 –Ichoghi ikwenye m’ o bu ekwenyeghi

4 –I kwenyeghi

5 –I kwenyeghisie ike

98 –Amaghi

99 –Ekweghi

*…SITS Nke ozo a ga m egosi/kowaputara gi onodu ndi mmadu n’ enweta onwe ha na ya’ N’ime nke o bula, biko gwam mmanya ole onye- Nkiti mmanya, ufodu mmanya mana o gaghi ezu, I mesa ha n’ ahu (otu mmanya m’ o bu abuo), ebe ozuni ezu imesa ha n’ahu mana o buhi nke mmanya igbu ha, mmanya ogbugbu d mma mgbe ufodu mana mmanya ogbugbu o dim man oge ebula?* *(Show card)*

|  |  | 1 – Nkit mmanya | 2 –. ufodu nmapya mana o gagha ezu imesa, ha n atru loru mmanya m’o bu bu ghio | 3 – ebe ozuru eze I meja ha n’ ahu mana o,bughi nke mmanya igbu ha | 4 – mmanya ogbugbu dim ma oge o bula | 5 – Inu bi ga mmaya oke mgbe obu na dim ma | 98 – amaghi | 99 – 6. ekweghi |
| --- | --- | --- | --- | --- | --- | --- | --- | --- |
| _2 | Dika onye nne, inwee nnoko gi n’ umuntakiri |  |  |  |  |  |  |  |
| _3 | Dika onye naa, inwee nnoko gi n’ umuntakiri |  |  |  |  |  |  |  |
| _6 | Gbasara nwoke n’ ulo mmanya na ndi enyi ya |  |  |  |  |  |  |  |
| _7 | Gbasara nwanyi ro n’ ulo mmanya an ndi enyi ya |  |  |  |  |  |  |  |
| _8 | Gbasara nwanyi puru apu na ndi oru ibe ya |  |  |  |  |  |  |  |
| _9 | Gbasara nwoke puru apu na ndi oru ibe ya |  |  |  |  |  |  |  |
| _12 | Gbasara nwoke na nwunye m’ o bu onye mmeko ya nay a n’ eri nri anyesi n’ ulo ya |  |  |  |  |  |  |  |
| _13 | Gbasara nwanyi na di ya m’ o bu onye mmeko ya nay a n’ eri nti anyasi n’ ulo ya |  |  |  |  |  |  |  |

# MODULE 20: Adolescents and Young Adults (ADYA)

## 20.3 Emerging Adulthood

EMAD.PERC Biko gwa m na ikwenyesin ike, ikwenyere, ichogji ikwenye m’ o bu ekwenyeghi, ikwenyeghi m’ o bu ikwemyeghisie ike n’ ihe okwu ndi a n’ esote *(Show card)*

...EMAD.PERC_1 E wola m igho ndi dimkpa

1 –Kwenyesiri ike

2 –Kwenyere

3 –Ichoghi ikwenye m’ o bu ekwenyeghi

4 –I kwenyeghi

5 –I kwenyeghisie ike

98 –Amaghi

99 –Ekweghi

...EMAD.PERC_2 N’ okwo gbasara ego, a noro m nke onwe m n’ ebe nne na nna m no

1 – Kwenyesiri ike

2 – Kwenyere

3 – Ichoghi ikwenye m’ o bu ekwenyeghi

4 –I kwenyeghi

5 –I kwenyeghisie ike

98 –Amaghi

99 –Ekweghi

...EMAD.PERC_3 N’ olwu gbasara o nodu, a noro ni nke onwe m n’ ebe nne na nna m no.

1 –Kwenyesiri ike

2 –Kwenyere

3 –Ichoghi ikwenye m’ o bu ekwenyeghi

4 –I kwenyeghi

5 –I kwenyeghisie ike

98 –Amaghi

99 –Ekweghi

# MODULE 21: Respondent Engagement, Recruiting and Screening (RCRT)

Ugbu a ayin na a bia na nje debe ajujua. M ga a cho iju gi o bere a juju ndi ozo.

…ENG1 Bido n’otu ruo na iri, ofu onye etinyegbi. Uche mana mmadu iri tinyere uche nke oma, kedi out ihe mmataa siri ma sigi?

__ __

98 – Amaghi

99 – Ekweghi

…ENG2 Bido, kwa n’out ruo na iri, ofu onye etinyeghi uche mana mmadu iri tinyere uche nke oma. Kedi out ihe mmutaa siri masi gi?

__ __

98 – Amaghi

99 – Ekweghi

Anyi enitewala n’ ngwucha nke ajuju onu a; dik m kwuputara na mbido o di oke mkpa n’ agamnihu ihe omumu a n’ anyig’ anakota ozi site n’aka ndi mmadu nwere akpuramagwa di iche iche, otu ha si ahygasi ihe n’ out ha si hutakwata ya n’ oge gara aga – ya bu ndewo. Anyi n’ ele eniya n’ ndi sonyere anyi n’ ajuju onu g’ etinyekwa ya n’ uche isonyekwa any, n’ agba nke abuo. Agba nke abise gunyere iza ajuju online. O buhi onye o bula kwenyere n’ isonye oniyi n’ agba nke abuo k’a ga akpo, mana anyi chooro I nweta ozi site na nghota ndi di iche iche

...PART O g’ amasi gi isonye anyi n’ agbanke abuo

1- eee, o masiri m isonye n’ agba nke abuo nke ihe omumu a

2 – Mba o gaghi amasi m isoniye n’ agba nke abuo n’ ne omumu a (Skip to …SCRN)

98 –Amaghi

99 –Ekweghi

…PART.REF ke di ihe kpatara na I ohoro I so ro ayin nokorita?)

______________________ (open-ended response)

98 –Amaghi

99 –Ekweghi

…SCRN...

…SCRN_STDN Kedu udili onye akwukwo ibu:

1 –abum nwata akwukwo oge juru ebu)

2 –abum nwata akwukwo akuku oge)

3 –abu rum nwa ta akwukwo) (Skip to …SCREEN.INT)

98 –Amaghi

99 –Ekweghi

…SCRN.EDUC kedi ihe kacha mma aga eji tureye ihe mmu taa nke iti yere aka na ya

1 –ulo akwukwo agba nke abua) (e.g. high school, secondary school)

2 –akwukwo nke ndi obodo nka na uzu) FURTHER EDUCATION COURSE (e.g. community college, technical college)

3 – ulo akwukwo di elu (ma ha dum)HIGHER EDUCATION COURSE (e.g. university)

4 – (Ndi ozo) _______________________________________

98 – Amaghi

99 – Ekweghi

…SCRN.INT ugboro ole ka ina e ti ye internet no lu)

1 –Ubochi ni le

2– Otu mgbe na I zu uka

3 – Otu mgbe na owa

4 – o na ha e ru o tu mgbe na o wa

5 –o we ro rom

98 –Amaghi

99 –Ekweghi

…SCRN.CHCK U gbo ro ole ka I na a sa pe email)

1 –Ubochi obula

2 –Otu ugboro izu uka

3 –Otu ugboro n’ onwa

4 –Ihe na otu ugboro n’ onwa

5 –Enwebegbi n

6 – mba email

98 –Amaghi

99 –Ekweghi

(IF PART=2 (DOES NOT WANT TO PARTICIPATE) END INTERVIEW

(IF SCRN.INT = 5 OR 6 **AND** SCRN.CHCK = 5 OR 6, THEN END.)

(IF SCRN.INT = 1-4 **AND** SCRN.CHCK = 5 OR 6, THEN GO TO PART.CNTC.OTH)

(IF SCRN.INT = 1-4 **AND** SCRN.CHCK = 1-4, CONTINUE)

…PART.EMAIL Anyi ga n’ akpoturu ndi mmadu site na “e-mail “ gbasra agba nke abuo ihe omumu a, inwere e-mail adresi ebe anyi nwere ike ikpoturu gi

1 –Eh, email bu:____________________________________________

***(Interviewer: Verify email address by asking respondent to repeat address.)***

2 – mba email adresi)

98 –Amaghi

99 –Ekweghi

…PART.CNTC.OTH I were ike I ye a yin uzo ozo a yin we ike iji we ta e be ibi a di a ma ama obu na a yi e we si ike I we te gin a email gi gi (ekwenti) or Ewerem phone number

1 – Eh, phone number m bu:__________________________________

***(Interviewer: Verify by asking respondent to repeat.)***

*2 –Eh, adresi mbu: ________________________________________****____***

***(Interviewer: Verify by asking respondent to repeat.)***

3 – mba phone number

98 –Amaghi

99 –Ekweghi

[THANK YOU AND END]
