# Supplementary material for: Context and culture associated with alcohol use amongst youth in major urban cities: A cross-country population based survey
Source: PLoS One. 2017 Nov 20;12(11):e0187812. doi: 10.1371/journal.pone.0187812 (PMC5695777; doi:10.1371/journal.pone.0187812)
Supplement: S5 Questionnaire — (DOCX) [file pone.0187812.s005.docx]

**Опросник исследования LTACC**

**Содержание**

[Модуль A: Идентификация и профиль домовладения, согласие, скрининговые вопросы (ADMN) 2](#_Toc407633186)

[МОДУЛЬ 1: Демография 4](#_Toc407633187)

[МОДУЛЬ 9: Отношение к здоровью, включая статус употребления алкоголя (HTBX) 8](#_Toc407633188)

[МОДУЛЬ 10: Потребление алкоголя, последние 12 месяце (CONS) 9](#_Toc407633189)

[10.1 Общая характеристика употребления алкоголя 9](#_Toc407633190)

[10.2 Количество/частота употребления конкретных видов алкогольных напитков 10](#_Toc407633191)

[10.3 Опыт состояния опьянения / интоксикации 12](#_Toc407633192)

[10.4 Контекст употребления алкоголя 13](#_Toc407633193)

[МОДУЛЬ 15: Мотивация за и против употребления алкоголя, ожидаемые результаты употребления. Только для употребляющих алкоголь (MTCD) 14](#_Toc407633194)

[МОДУЛЬ 16: Мотивация за и против употребления алкоголя и опыт употребления алкоголя. Только для не употребляющих алкоголь (MTND) 16](#_Toc407633195)

[МОДУЛЬ 17: Восприятие алкоголя и других веществ опрашиваемым и окружающими (PCPN) 18](#_Toc407633196)

[МОДУЛЬ 20: Подростки и молодые взрослые (ADYA) 19](#_Toc407633197)

[20.3 Взросление 19](#_Toc407633198)

[МОДУЛЬ 21: Вступление в контакт с респондентом, привлечение к исследованию и скрининг (RCRT) 19](#_Toc407633199)

# **Модуль A: Идентификация и профиль домовладения, согласие, скрининговые вопросы (ADMN)**

***A.0 Идентификация домовладения и интервьюера, дата*** *(Заполнить до посещения домовладения/респондента)*

…LEV1 Город/район города:__ __ (см. список кодов)

…LEV2 [В зависимости от страны, см. сравнительную таблицу]: __ __ (см. список кодов)

…LEV3 [В зависимости от страны, см. сравнительную таблицу]:__ __ (см. список кодов)

…LEV4 [В зависимости от страны, см. сравнительную таблицу]:__ __ (см. список кодов)

…INTID __ __ __ __ (цифровая подпись интервьюера)

…DATE __ __ (ДД) __ __ (ММ) __ __ __ __ (ГГГГ)

***A.1 Отношение к интервью в домовладении и у респондента*** *(Заполнить после посещения домовладения/респондента)*

…HDIS Отношение в домовладении:__ __ (см. список кодов)

…RDIS Отношение респондента: __ __ (см. список кодов)

…RDIS_TXT Причина досрочного прекращения интервью, если было (напр., ADMN.RDIS = 25 or 26) ________________________________________________________________________

________________________________________________________________________

***A.2 Представление и согласие, определение приемлемости респондента***

...INTR Здравствуйте. Меня зовут (назовите свое имя). Я – профессиональный интервьюер, сотрудник Центра Юрия Левады, которым сейчас осуществляет международное исследование, посвященное изучению особенностей употребления алкоголя. Ваш адрес был выбран случайным образом для включения в данное исследование. Можно задать Вам несколько вопросов, чтобы определить, подходит ли что кто-то из проживающих в домовладении под критерии нашего исследования?

*После получения согласия:*

Большое спасибо. Сообщите, пожалуйста, возраст и пол всех, кто в настоящее время проживает в этом домохозяйстве?

*В соответствии с ответами, заполните столбцы 1 и 2 предложенной формы, внеся туда возраст и отметив кружком пол. Про каждого из перечисленных лиц, находящихся в возрасте от 18 до 34 лет, уточните, жили ли он/она в городе в течение не менее чем 6 месяцев и заполните соответствующим образом 3-й столбец.*

Состав домохозяйства

|  | **Возраст** | **Пол** | **Проживает в [городе / районе] более 6 мес.?** |  |  | **Возраст** | **Пол** | **Проживает в [городе / районе] более 6 мес.?** |
| --- | --- | --- | --- | --- | --- | --- | --- | --- |
| **Человек 1** |  | M / Ж | ДА / НЕТ |  | **Человек 7** |  | M / Ж | ДА / НЕТ |
| **Человек 2** |  | M / Ж | ДА / НЕТ |  | **Человек 8** |  | M / Ж | ДА / НЕТ |
| **Человек 3** |  | M / Ж | ДА / НЕТ |  | **Человек 9** |  | M / Ж | ДА / НЕТ |
| **Человек 4** |  | M / Ж | ДА / НЕТ |  | **Человек 10** |  | M / Ж | ДА / НЕТ |
| **Человек 5** |  | M / Ж | ДА / НЕТ |  | **Человек 11** |  | M / Ж | ДА / НЕТ |
| **Человек 6** |  | M / Ж | ДА / НЕТ |  | **Человек 12** |  | M / Ж | ДА / НЕТ |

*В случае отсутствия подходящего по критериям исследования респондента, переходите к домохозяйству, следующему по списку. В случае если в домохозяйстве несколько респондентов, соответствующих критериям, выберите одного из них, используя метод «последнего дня рождения» и попросите дать возможность с ним/c ней побеседовать. Перейдите к пункту А 1 и заполните ответы на приведенные ниже вопросы только после завершения интервью (или попытки интервью) с избранным респондентом.*

…LANG2 *Язык проведения интервью: _______________________________*

…LANG3 *Оцените комфортность языка, на котором проводилось интервью по шкале от 1 до 5 (1 = интервьюируемый не понимает вопросов, не может закончить интервью; 5 = интервьюируемый легко понимает все вопросы и отвечает на них):__*

***A.3 Представление респонденту и получение согласия***

...INST1 Здравствуйте. Меня зовут (назовите свое имя). Я – профессиональный интервьюер, сотрудник Центра Юрия Левады. Наш Центр проводит международное исследование, посвященное изучению особенностей употребления алкоголя. Ваш адрес был выбран случайным образом для включения в данное исследование. Для успеха исследования мы должны собрать информацию от людей, отличающихся по своему отношению, опыту и взглядам, включая и таких людей, как Вы. Для участия в исследовании Вы должны ответить на вопросы о (здоровье, отношение к алкоголю и поведение, включая потребление алкоголя). Для заполнения опросника потребуется от 20 до 35 минут. Ваше решение принять участие в опросе или отказаться является абсолютно добровольным. Если Вы согласитесь принять участие в исследовании, Ваши ответы будут сохранены в полной конфиденциальности. Если Вы будете испытывать неудобство, отвечая на какой-либо вопрос, мы можем его пропустить. Вы также можете в любой момент прервать интервью. Согласны ли Вы принять участие в исследовании?

________

подпись интервьюера,

означающая устное согласие респондента

***A.4 Скрининговые и административные вопросы***

...INST2 Спасибо. Перед тем, как начать, хотелось бы задать Вам несколько вопросов, чтобы уточнить, подходите ли Вы для участия в исследовании.

...BYR В каком году Вы родились?

__ __ __ __

98 – НЕ ЗНАЮ

99 – ОТКАЗ

*Исполнилось ли Вам 18 лет (1996 год рождения)? Нет ли Вам ещё 35-ти лет (1980 год рождения)? Если респондент не находится в требуемом возрасте, или возраст невозможно установить, интервью заканчивается [Кодируется «не подходит»].*

…RESI Вы живёте в городе в течение последних 6 месяцев?

1 – ДА

2 – НЕТ

98 – НЕ ЗНАЮ

99 – ОТКАЗ

*Интервью заканчивается, если респондент не проживал в городе / районе на протяжении, как минимум, 6-ти месяцев. [Закодируйте респондента «Не подходит».]*

…LANG1 Каков Ваш основной язык?

_________________________________

98 – НЕ ЗНАЮ

99 – ОТКАЗ

*Интервью, по возможности, проводится на основном языке респондента. Если это невозможно, или основной язык респондента неизвестен, по завершении интервью оцените уровень комфортности использования для респондента языка, на котором интервью проводилось (см. предыдущую страницу).*

***A.5 Введение к интервью***

…INST3 Если Вы согласны, я начну интервью. Запомните, конфиденциальность ваших ответов будет соблюдаться строжайшим образом, так что, пожалуйста, постарайтесь честно ответить на все вопросы. Если Вы почувствуете неудобство, отвечая на какой-либо вопрос, скажите мне, и мы перейдем к следующему.

…STRT  *Зафиксируйте время начала интервью:*

__ __ : __ __ (ЧЧ:ММ, 24-часовой формат)

# МОДУЛЬ 1: Демография

…SEX *(Укажите пол, исходя из собственных наблюдений. При необходимости уточните)*

1 – МУЖЧИНА

2 – ЖЕНЩИНА

98 – НЕ ЗНАЮ 99 – ОТКАЗ

...MAR Как лучше всего охарактеризовать Ваше семейное положение?

1 – Женат/замужем

2 – Разведен ИЛИ НЕ ПРОЖИВАЕТЕ ВМЕСТЕ

3 – Вдовец/вдова

4 – Никогда не состоял в браке (Перейти к …HH.AD)

98 – НЕ ЗНАЮ (Перейти к …HH.AD)

99 – ОТКАЗ (Перейти к …HH.AD)

…MAR_FU В каком году вы (женились/развелись или разошлись/овдовели)?

__ __ __ __

98 – НЕ ЗНАЮ 99 – ОТКАЗ

...HH.AD Не считая Вас, сколько взрослых (старше 18 лет) проживают в этом домохозяйстве?

__ __ ВЗРОСЛЫХ *(если 0, Перейти к DEM.HH.AD_FU2)*

98 – НЕ ЗНАЮ 99 – ОТКАЗ

…HH.AD_FU1 Кто они? (выберите все подходящее)

1 – Супруг/супруга или партнер

2 – Родитель или опекун (*Перейти к DEM.HH.PNT1)*

3 – Другой член семьи

4 – Приятель, сосед по комнате

или другой не родственник

98 – НЕ ЗНАЮ 99 – ОТКАЗ

…HH.AD_FU2 С какого года Вы покинули родительский дом и живете самостоятельно?

__ __ __ __

98 – НЕ ЗНАЮ 99 – ОТКАЗ

...PNT1 Есть ли у Вас дети?

1 – ДА

2 – НЕТ *(Перейти к DEM.EDU)*

98 – НЕ ЗНАЮ *(Перейти к DEM.EDU)*

99 – ОТКАЗ *(Перейти к DEM.EDU)*

…PNT1_FU Сколько у вас детей?

______ детей

98 – НЕ ЗНАЮ *(Перейти к DEM.EDU)*

99 – ОТКАЗ *(Перейти к DEM.EDU)*

*…PNT2 …PNT2_FU*

| Ребёнок № | Сколько Вашим детям лет? (начиная с более младших)  98 – НЕ ЗНАЮ  99 – ОТКАЗ | Он или она живёт с Вами?  98 – НЕ ЗНАЮ  99 – ОТКАЗ |
| --- | --- | --- |
| 1 | PNT2_1  _____ _____ ЛЕТ | PNT2_1_FU  1 – ДА 2 – НЕТ |
| 2 | PNT2_2  _____ _____ ЛЕТ | PNT2_2_FU  1 – ДА 2 – НЕТ |
| 3 | PNT2_3  _____ _____ ЛЕТ | PNT2_3_FU  1 – ДА 2 – НЕТ |
| 4 | PNT2_4  _____ _____ ЛЕТ | PNT2_4_FU  1 – ДА 2 – НЕТ |
| 5 | PNT2_5  _____ _____ ЛЕТ | PNT2_5_FU  1 – ДА 2 – НЕТ |
| 6 | PNT2_6  _____ _____ ЛЕТ | PNT2_6_FU  1 – ДА 2 – НЕТ |
| 7 | PNT2_7  _____ _____ ЛЕТ | PNT2_7_FU  1 – ДА 2 – НЕТ |
| 8 | PNT2_8  _____ _____ ЛЕТ | PNT2_8_FU  1 – ДА 2 – НЕТ |

...EDU Ваше образование?

1 – 9 (8) классов средней школы или менее

2 – 11 (10) классов средней школы

3 – Неоконченное среднее профессиональное или специальное

4 – Оконченное среднее профессиональное или специальное

5 – Неоконченное высшее

6 – Оконченное высшее

7 – Другое: _____________________________________________________

98 – НЕ ЗНАЮ 99 – ОТКАЗ

___________________________________ [В ЗАВИСИМОСТИ ОТ СТРАНЫ]

98 – НЕ ЗНАЮ 99 – ОТКАЗ

…STDT Вы сейчас учитесь?

1 – ДА *(Перейти к DEM.EMPL)*

2 – НЕТ

98 – НЕ ЗНАЮ 99 – ОТКАЗ

…STDT_GR В каком году вы получили диплом или прервали образование?

__ __ __ __

98 – НЕ ЗНАЮ 99 – ОТКАЗ

...EMPL Как Вы можете охарактеризовать свою занятость?

1 – Полная занятость (40 и более часов в неделю, включая самозанятость) *(Переход к EMPL_FU3)*

2 – Частичная занятость (менее 40 часов в неделю, включая самозанятость) *(Перейти к EMPL_FU3)*

3 – Безработный

4 – ДОМОХОЗЯЙКА

5 – Инвалид или нетрудоспособный *(Перейти к DEM.OCC)*

6 – ИНОЕ: _______________________________________________ *(Перейти к EMPL_FU1)*

98 – НЕ ЗНАЮ 99 – ОТКАЗ

...EMPL_FU1 Вы сейчас заняты поиском оплачиваемой работы?

1 – ДА

2 – НЕТ *(Перейти к EMPL_FU4.YR)*

98 – НЕ ЗНАЮ *(Перейти к EMPL_FU4.YR)*

99 – ОТКАЗ *(Перейти к EMPL_FU4.YR)*

...EMPL_FU2 Это будет Ваша первая оплачиваемая работа?

1 – ДА *(Перейти к DEM.REL)*

2 – НЕТ *(Перейти к EMPL_FU4)*

98 – НЕ ЗНАЮ *(Перейти к EMPL_FU4.YR)*

99 – ОТКАЗ *(Перейти к EMPL_FU4.YR)*

...EMPL_FU3 Это Ваша первая оплачиваемая работа?

1 – ДА

2 – НЕТ

98 – НЕ ЗНАЮ 99 – ОТКАЗ

…EMPL_FU4 В каком году Вы начали работать (за плату)?

__ __ __ __

97 – Я НИКОГДА НЕ РАБОТАЛ(А) ЗА ПЛАТУ

98 – НЕ ЗНАЮ 99 – ОТКАЗ

…OCC Каков Ваш род занятий или профессия? (если Вы в настоящее время не работаете, укажите, где работали раньше. Если Вы работали в нескольких местах, укажите, где работали дольше всего).

| **1** | Военнослужащий |
| --- | --- |
| **2** | ЮРИСТ / УПРАВЛЯЮЩИЙ / МЕНЕДЖЕР |
| **3** | КВАЛИФИЦИРОВАННЫЙ СПЕЦИАЛИСТ |
| **4** | ТехниЧЕСкиЙ и вспомогательный персонал |
| **5** | ОФИСНАЯ РАБОТА |
| **6** | сфера услуг / продавцы в магазинах и на рынках |
| **7** | КвалифицированнАЯ сельхозработА / рыбаЛОВСТВО |
| **8** | РемесленнАЯ РАБОТА |
| **9** | РАБОТА НА ЗАВОДЕ / оператор оборудования / сборКА |
| **10** | Неквалифицированная работа |
| **97** | Никогда не работал |
| **98** | НЕ ЗНАЮ |
| **99** | ОТКАЗ |

…REL Ваше отношение к религии?

1 – Агностик/атеист

2 – БуддиЗМ

3 – ислам

4 – Иудаизм

5 – ПРАВОСЛАВНОЕ ХРИСТИАНСТВО

6 – ХРИСТИАНСТВО – ДРУГИЕ ТЕЧЕНИЯ (КАТОЛИЦИЗМ, ПРОТЕСТАНТИЗМ)

7 – ЯЗЫЧЕСКАЯ, ВЕДИЧЕСКАЯ, СЛАВЯНСКАЯ ИЛИ СКАНДИНАВСКАЯ ТРАДИЦИЯ

8 – ДРУГАЯ РЕЛИГИЯ: ___________________________________

98 – НЕ ЗНАЮ 99 – ОТКАЗ

…RACE Ваша национальность или этническая принадлежность?

____________________________________________

98 – НЕ ЗНАЮ 99 – ОТКАЗ

# МОДУЛЬ 9: Отношение к здоровью, включая статус употребления алкоголя (HTBX)

…ALC.EVER Вы когда-нибудь употребляли спиртные напитки (пиво, вино, водку, алкогольные коктейли и т.д.)? Пожалуйста, не учитывайте случаи, если Вы только отпили пару глотков у кого-то.

1 – ДА

2 – НЕТ *(Перейти к MTND.MOTV.AGST)*

98 – НЕ ЗНАЮ 99 – ОТКАЗ

…ALC.STRT В каком возрасте Вы впервые попробовали алкогольный напиток? Пожалуйста, не учитывайте случаи, если Вы только отпили пару глотков у кого-то

В __ __ ЛЕТ

98 – НЕ ЗНАЮ 99 – ОТКАЗ

…ALC.DRUK В каком возрасте Вы впервые напились?

В __ __ ЛЕТ

97 – ТАКОГО НИКОГДА НЕ БЫЛО

98 – НЕ ЗНАЮ 99 – ОТКАЗ

…ALC.HVY.STRT Нас интересует, в какой период жизни Вы пили больше всего? В каком возрасте начался этот период?

В __ __ ЛЕТ

98 – НЕ ЗНАЮ 99 – ОТКАЗ

…ALC.HVY.STOP В каком возрасте этот период закончился?

В __ __ ЛЕТ

97 – ЭТОТ ПЕРИОД НЕ ЗАКОНЧИЛСЯ

98 – НЕ ЗНАЮ 99 – ОТКАЗ

# МОДУЛЬ 10: Потребление алкоголя, последние 12 месяце (CONS)

## 10.1 Общая характеристика употребления алкоголя

…GEN.FQ В течение последних 12 месяцев, как часто Вы употребляли пиво, вино, крепкие алкогольные напитки (водка, джин, виски, бренди) или любые другие алкогольные напитки, даже в малых количествах? (показать карточку)

1 — ЕЖЕДНЕВНО

2 — 5-6 РАЗ В НЕДЕЛЮ

3 — 3-4 РАЗА В НЕДЕЛЮ

4 — 1-2 РАЗА В НЕДЕЛЮ

5 — 2-3 РАЗА В МЕСЯЦ

6 — РАЗ В МЕСЯЦ

7 — 6-11 РАЗ ЗА ПРОШЕДШИЙ ГОД

8 — 2-5 РАЗ ЗА ПРОШЕДШИЙ ГОД

9 — 1 РАЗ ЗА ПРОШЕДШИЙ ГОД

10 — НИ РАЗУ ЗА ПРОШЕДШИЙ ГОД *(Перейти к MTND.EFCT)*

98 – НЕ ЗНАЮ 99 – ОТКАЗ

…GEN.QY — За последние 12 месяцев, сколько порций алкоголя Вы обычно выпивали в тот день, когда пили? Порция алкоголя – это бутылка, банка или кружка пива (500 мл), бокал вина или шампанского (150 мл), банка тонизирующего алкогольного напитка (500 мл), рюмка крепкого напитка (50 мл) или алкогольный коктейль, приготовленный на основе крепкого алкогольного напитка (показать карточку)

1 — 25 порций И БОЛЕЕ

2 — 19-24 ПОРЦИЙ

3 — 16-18 ПОРЦИЙ

4 — 12-15 ПОРЦИЙ

5 — 9-11 ПОРЦИЙ

6 — 7-8 ПОРЦИЙ

7 — 5-6 ПОРЦИЙ

8 — 3-4 ПОРЦИЙ

9 — 2 ПОРЦИИ

10 — 1 ПОРЦИЮ

11 — МЕНЕЕ 1 ПОЛНОЙ ПОРЦИИ *(если* ***одновременно*** *CONS.GEN.FQ = 9 и GEN.QY=11, перейти к MTND.EFCT)*

98 – НЕ ЗНАЮ 99 – ОТКАЗ

…GEN.MST.QY — За последние 12 месяцев, какое наибольшее количество порций алкоголя Вы выпили за 24 часа? *(Покажите карточку)*

1 — 36 ПОРЦИЙ И БОЛЕЕ

2 — 25-35 ПОРЦИЙ

3 — 19-24 ПОРЦИЙ

4 — 16-18 ПОРЦИЙ

5 — 12-15 ПОРЦИЙ

6 — 9-11 ПОРЦИЙ

7 — 7-8 ПОРЦИЙ

8 — 5-6 ПОРЦИЙ

9 — 3-4 ПОРЦИЙ

10 — 2 ПОРЦИИ

11 — 1 ПОРЦИЮ

12 — МЕНЕЕ 1 ПОЛНОЙ ПОРЦИИ

98 – НЕ ЗНАЮ 99 – ОТКАЗ

…GEN.MST.FQ — Сколько раз за последние 12 месяцев Вы выпивали [назовите число порций - ответ, данный в предыдущем вопросе GEN.MST.QY] в течение 24 часов? *(Покажите карточку)*

1 — ЕЖЕДНЕВНО

2 — 5-6 РАЗ В НЕДЕЛЮ

3 — 3-4 РАЗА В НЕДЕЛЮ

4 — 1-2 РАЗА В НЕДЕЛЮ

5 — 2-3 РАЗА В МЕСЯЦ

6 — РАЗ В МЕСЯЦ

7 — 6-11 РАЗ ЗА ПРОШЕДШИЙ ГОД

8 — 2-5 РАЗ ЗА ПРОШЕДШИЙ ГОД

9 — ОДИН РАЗ ЗА ПРОШЕДШИЙ ГОД

98 – НЕ ЗНАЮ 99 – ОТКАЗ

## 10.2 Количество/частота употребления конкретных видов алкогольных напитков

Теперь мне хотелось бы узнать, как часто в течение последних 12 месяцев Вы употребляли конкретные виды алкогольных напитков, и как много каждого из этих напитков Вы выпивали в тот день, когда Вы их употребляли

…BSFQ.BR.FQ Как часто за последние 12 месяцев Вы пили **пиво**? *(Покажите карточку)*

1 — ЕЖЕДНЕВНО

2 — 5-6 РАЗ В НЕДЕЛЮ

3 — 3-4 РАЗА В НЕДЕЛЮ

4 — 1-2 РАЗА В НЕДЕЛЮ

5 — 2-3 РАЗА В МЕСЯЦ

6 — РАЗ В МЕСЯЦ

7 — 6-11 РАЗ ЗА ПРОШЕДШИЙ ГОД

8 — 2-5 РАЗ ЗА ПРОШЕДШИЙ ГОД

9 — ОДИН РАЗ ЗА ПРОШЕДШИЙ ГОД

10 — НИ РАЗУ ЗА ПРОШЕДШИЙ ГОД *(Перейти к CONS.BSFQ.WN.FQ)*

98 – НЕ ЗНАЮ 99 – ОТКАЗ

…BSFQ.BR.QY В типичный день, когда Вы пили пиво, сколько пива вы выпивали?

__ __ *(Покажите карточку для стандартных порций)*

98 – НЕ ЗНАЮ 99 – ОТКАЗ

…BSFQ.WN.FQ Как часто за последние 12 месяцев Вы пили **вино**? *(Покажите карточку)*

1 — ЕЖЕДНЕВНО

2 — 5-6 РАЗ В НЕДЕЛЮ

3 — 3-4 РАЗА В НЕДЕЛЮ

4 — 1-2 РАЗА В НЕДЕЛЮ

5 — 2-3 РАЗА В МЕСЯЦ

6 — РАЗ В МЕСЯЦ

7 — 6-11 РАЗ ЗА ПРОШЕДШИЙ ГОД

8 — 2-5 РАЗ ЗА ПРОШЕДШИЙ ГОД

9 — ОДИН РАЗ ЗА ПРОШЕДШИЙ ГОД

10 — НИ РАЗУ ЗА ПРОШЕДШИЙ ГОД *(Перейти к BSFQ.SP.FQ)*

98 – НЕ ЗНАЮ 99 – ОТКАЗ

…BSFQ.WN.QY В типичный день, когда Вы пили вино, сколько вина Вы выпивали?

__ __ *(Покажите карточку для стандартных порций)*

98 – НЕ ЗНАЮ 99 – ОТКАЗ

…BSFQ.SP.WH За последние 12 какие **крепкие напитки** Вы пили? *(Отметьте все, которые Вы пили)*

1 — ВОДКА

2 — КОНЬЯК / БРЕНДИ

3 – ВИСКИ

4 — САМОГОН

5 — КОНТРАФАКТНАЯ ВОДКА

6 — ДРУГОЙ КРЕПКИЙ НАПИТОК (укажите) ________________________

…BSFQ.SP.FQ Как часто за последние 12 месяцев Вы пили **крепкие напитки**? *(Покажите карточку)*

1 — ЕЖЕДНЕВНО

2 — 5-6 РАЗ В НЕДЕЛЮ

3 — 3-4 РАЗА В НЕДЕЛЮ

4 — 1-2 РАЗА В НЕДЕЛЮ

5 — 2-3 РАЗА В МЕСЯЦ

6 — РАЗ В МЕСЯЦ

7 — 6-11 РАЗ ЗА ПРОШЕДШИЙ ГОД

8 — 2-5 РАЗ ЗА ПРОШЕДШИЙ ГОД

9 — ОДИН РАЗ ЗА ПРОШЕДШИЙ ГОД

10 — НИ РАЗУ ЗА ПРОШЕДШИЙ ГОД *(Перейти к CONS.BSFQ.OTR.FQ)*

98 – НЕ ЗНАЮ 99 – ОТКАЗ

…BSFQ.SP.QY В типичный день, когда Вы пили крепкие напитки, сколько Вы их выпивали?

__ __ *(Покажите карточку для стандартных порций)*

98 – НЕ ЗНАЮ 99 – ОТКАЗ

…BSFQ. OTR.WH За последние 12 какие слабоалкогольные **энергетические напитки** Вы пили? *(Отметьте все, которые Вы пили)*

1 — ЯГУАР (JAGUAR)

2 — ТЭН СТРАЙК (TEN STRIKE) – скай или дарк

3 — РЕД ДЕВЛ (RED DEVIL)

4 — Икс-Икс-Эль АЛКО ПАУЭР (XXL ALCO POWER)

5 — ОТВЕРТКА

6 — ДРУГОЙ АЛКОГОЛЬНЫЙ ЭНЕРГЕТИЧЕСКИЙ НАПИТОК (укажите) ________________________

…BSFQ.OTR.FQ Как часто за последние 12 месяцев Вы пили слабоалкогольные энергетические напитки (Ягуар, Тэн Страйк, Рэд Дэвл и др.)? *(Покажите карточку)*

1 — ЕЖЕДНЕВНО

2 — 5-6 РАЗ В НЕДЕЛЮ

3 — 3-4 РАЗА В НЕДЕЛЮ

4 — 1-2 РАЗА В НЕДЕЛЮ

5 — 2-3 РАЗА В МЕСЯЦ

6 — РАЗ В МЕСЯЦ

7 — 6-11 РАЗ ЗА ПРОШЕДШИЙ ГОД

8 — 2-5 РАЗ ЗА ПРОШЕДШИЙ ГОД

9 — ОДИН РАЗ ЗА ПРОШЕДШИЙ ГОД

10 — НИ РАЗУ ЗА ПРОШЕДШИЙ ГОД *(Перейти к DRUK.FQ)*

98 – НЕ ЗНАЮ 99 – ОТКАЗ

…BSFQ.OTR.QY В типичный день, когда Вы пили слабоалкогольные энергетические напитки (Ягуар, Тэн Страйк, Рэд Дэвл и др.), сколько таких напитков Вы выпивали?

__ __ *(Покажите карточку для стандартных порций)*

98 – НЕ ЗНАЮ 99 – ОТКАЗ

##

## 10.3 Опыт состояния опьянения / интоксикации

…DRUK.FQ Как часто за последние 12 месяцев Вы выпивали столько спиртного, что почувствовали алкогольное опьянение/интоксикацию – нетвердо держались на ногах, неясно видели, отмечали нарушения речи? *(Покажите карточку)*

1. ЕЖЕДНЕВНО

2. 5-6 РАЗ В НЕДЕЛЮ

3. 3-4 РАЗА В НЕДЕЛЮ

4. 1-2 РАЗА В НЕДЕЛЮ

5. 2-3 РАЗА В МЕСЯЦ

6. РАЗ В МЕСЯЦ

7. 6-11 РАЗ ЗА ПРОШЕДШИЙ ГОД

8. 2-5 РАЗ ЗА ПРОШЕДШИЙ ГОД

9. ОДИН РАЗ ЗА ПРОШЕДШИЙ ГОД

10. НИ РАЗУ ЗА ПРОШЕДШИЙ ГОД *(Перейти к CONS.CXT1)*

98 – НЕ ЗНАЮ 99 – ОТКАЗ

…DRUK.NM Сколько порций Вам обычно нужно выпить, чтобы почувствовать алкогольное опьянение? (1 порция - это *(Покажите карточку для стандартных порций)*.)

__ __ ПОРЦИЙ

98 – НЕ ЗНАЮ 99 – ОТКАЗ

##

## 10.4 Контекст употребления алкоголя

Теперь мне хотелось бы узнать, как часто Вы употребляете алкоголь в конкретных ситуациях.

|  | Как часто за последние 12 месяцев Вы выпивали, когда Вы … | 1 - Каждый раз или почти каждый раз | 2 - По крайней мере, раз в неделю | 3 - По крайней мере, раз в месяц, но реже, чем раз в неделю | 4 - По крайней мере, раз в год, но реже, чем раз в месяц | 5 - Ни разу за пос­ледние 12 месяцев | 98 – НЕ ЗНАЮ | 99 - ОТКАЗ |  | А сколько порций Вы обычно выпивали *(пропустить, если ответ был 5, 98 или 99) Впишите число порций* | | |
| --- | --- | --- | --- | --- | --- | --- | --- | --- | --- | --- | --- | --- |
| …CXT1 | ужинали в ресторане? |  |  |  |  |  |  |  |  | 1.NM |  | |
|  |  |  |  |  |  |  |  |  |  |  | 98 | 99 |
| …CXT2 | обедали в ресторане? |  |  |  |  |  |  |  |  | 2.NM |  | |
|  |  |  |  |  |  |  |  |  |  |  | 98 | 99 |
| …CXT3 | были в баре, закусочной, коктейль-баре, пабе? |  |  |  |  |  |  |  |  | 3.NM |  | |
|  |  |  |  |  |  |  |  |  |  |  | 98 | 99 |
| …CXT4 | были дома у кого-то, в том числе в больших компаниях? |  |  |  |  |  |  |  |  | 4.NM |  | |
|  |  |  |  |  |  |  |  |  |  |  | 98 | 99 |
| …CXT5 | спокойно проводили вечер дома? |  |  |  |  |  |  |  |  | 5.NM |  | |
|  |  |  |  |  |  |  |  |  |  |  | 98 | 99 |
| …CXT6 | принимали у себя дома друзей? |  |  |  |  |  |  |  |  | 6.NM |  | |
|  |  |  |  |  |  |  |  |  |  |  | 98 | 99 |
| …CXT7 | с друзьями в общественных местах (парк, улица, автостоянка)? |  |  |  |  |  |  |  |  | 7.NM |  | |
|  |  |  |  |  |  |  |  |  |  |  | 98 | 99 |

…CXT.MEAL Как часто за последние 12 месяцев Ваша выпивка сопровождалась едой? Такое бывало…

1 – ВСЕГДА ИЛИ Почти всегда?

2 – Больше, чем в половине случаев?

3 –в половине случаев?

4 – МЕНЕЕ, ЧЕМ В ПОЛОВИНЕ СЛУЧАЕВ?

5 – Никогда или почти никогода?

98 – НЕ ЗНАЮ

99 – ОТКАЗ

…CXT.SELF Как часто за последние 12 месяцев Вы употребляли спиртное наедине? Такое было …

1 – ВСЕГДА ИЛИ Почти всегда?

2 – Больше, чем в половине случаев?

3 –в половине случаев?

4 – МЕНЕЕ, ЧЕМ В ПОЛОВИНЕ СЛУЧАЕВ?

5 – Никогда или почти никогода?

98 – НЕ ЗНАЮ

99 – ОТКАЗ

# МОДУЛЬ 15: Мотивация за и против употребления алкоголя, ожидаемые результаты употребления. Только для употребляющих алкоголь (MTCD)

…EFCT Употребление алкоголя воздействует на людей различным образом. Мы хотели бы узнать, какое воздействие оно могло оказывать на Вас. Когда Вы выпиваете, насколько справедливы для Вас следующие утверждения? Можно сказать, что с Вами это бывает очень часто, часто, иногда, редко или никогда?

|  | **Когда Вы пьете спиртные напитки, насколько справедливы в отношении Вас утверждения, что …** | **1 – ОЧЕНЬ ЧАСТО** | **2 – ЧАСТО** | **3 – ИНОГДА** | **4 – РЕДКО** | **5 – НИКОГДА** | **98 – НЕ ЗНАЮ** | **99 – ОТКАЗ** |
| --- | --- | --- | --- | --- | --- | --- | --- | --- |
| _1 | Вы расслабляетесь? |  |  |  |  |  |  |  |
| _2 | Вы чувствуете себя счастливым? |  |  |  |  |  |  |  |
| _3 | Вы становитесь более агрессивным к окружающим? |  |  |  |  |  |  |  |
| _4 | Вы чувствуете себя более дружелюбным? |  |  |  |  |  |  |  |
| _5 | Вам проще говорить о своих чувствах или проблемах? |  |  |  |  |  |  |  |
| _6 | Вы забываете о своих проблемах? |  |  |  |  |  |  |  |
| _7 | Вы делаете что-то, о чем позже сожалеете? |  |  |  |  |  |  |  |
| _8 | Вы получаете большее удовольствие от секса? |  |  |  |  |  |  |  |
| _9 | Вы чувствуете себя более сексуально привлекательным? |  |  |  |  |  |  |  |
| _10 | У Вас случаются проблемы с полицией? |  |  |  |  |  |  |  |
| _11 | Вам очень весело? |  |  |  |  |  |  |  |
| _12 | Вы плохо себя чувствуете? |  |  |  |  |  |  |  |
| _13 | Не можете вспомнить происходившее (провалы в памяти)? |  |  |  |  |  |  |  |

…MOTV.FOR У людей бывают различные причины, чтобы употреблять алкоголь. Насколько важны приведенные причины лично для Вас? Можно сказать, что для Вас это очень важно, важно, не очень важно или совсем не важно?

|  |  | **1 – ОЧЕНЬ ВАЖНО** | **2 – ВАЖНО** | **3 – НЕ ОЧЕНЬ ВАЖНО** | **4 – СОВСЕМ НЕ ВАЖНО** | **98 – НЕ ЗНАЮ** | **99 – ОТКАЗ** |
| --- | --- | --- | --- | --- | --- | --- | --- |
| _1 | Чтобы быть более коммуникабельным и вежливым? |  |  |  |  |  |  |
| _2 | Потому что все остальные пьют? |  |  |  |  |  |  |
| _3 | Чтобы получать большее удовольствие от еды? |  |  |  |  |  |  |
| _4 | Для здоровья? |  |  |  |  |  |  |
| _5 | Чтобы хорошо себя чувствовать? |  |  |  |  |  |  |
| _6 | Чтобы помочь себе расслабиться? |  |  |  |  |  |  |
| _7 | Чтобы забыть о неприятностях? |  |  |  |  |  |  |
| _8 | Чтобы не чувствовать себя замкнутым и стеснительным? |  |  |  |  |  |  |
| _9 | Чтобы что-то отметить? |  |  |  |  |  |  |
| _10 | Потому что нравится вкус? |  |  |  |  |  |  |
| _11 | Чтобы утолить жажду? |  |  |  |  |  |  |

…MOTV.AGST Также у людей есть целый ряд причин, по которым они ограничивают своё потребление спиртных напитков или не пьют вообще. Насколько важны приведенные причины для Вас? Можно сказать, что для Вас это очень важно, важно, не очень важно или совсем не важно?

|  |  | **1 – ОЧЕНЬ ВАЖНО** | **2 – ВАЖНО** | **3 – НЕ ОЧЕНЬ ВАЖНО** | **4 – СОВСЕМ НЕ ВАЖНО** | **98 – НЕ ЗНАЮ** | **99 – ОТКАЗ** |
| --- | --- | --- | --- | --- | --- | --- | --- |
| _1 | (Пропустить, если мужчина) Если Вы беременны/хотите забеременеть? |  |  |  |  |  |  |
| _2 | Вам не нравится вкус? |  |  |  |  |  |  |
| _3 | Вам не нравится, как на Вас действует спиртное? |  |  |  |  |  |  |
| _4 | Вам приходилось видеть плохие последствия алкоголя? |  |  |  |  |  |  |
| _5 | Пьянство другого человека причинило Вам горе? |  |  |  |  |  |  |
| _6 | Пьянство может отрицательно сказаться на Вашей работе, учебе? |  |  |  |  |  |  |
| _7 | Пьянство слишком дорого и является пустой тратой денег? |  |  |  |  |  |  |
| _8 | По религиозным причинам? |  |  |  |  |  |  |
| _9 | Потому что Вы так воспитаны? |  |  |  |  |  |  |
| _10 | У Вас были проблемы со спиртным, Вы боитесь стать алкоголиком? |  |  |  |  |  |  |
| _11 | Потому что Вы слишком молоды? |  |  |  |  |  |  |
| _12 | Ваши друзья и (или) члены семьи не одобряют Ваше пьянство? |  |  |  |  |  |  |
| _14 | Из-за здоровья? |  |  |  |  |  |  |
| _15 | Вам это просто не интересно? |  |  |  |  |  |  |

*Все респонденты, заполнившие этот модуль, переходят к Модулю 17.*

# МОДУЛЬ 16: Мотивация за и против употребления алкоголя и опыт употребления алкоголя. Только для не употребляющих алкоголь (MTND)

***[Этот вопрос для тех, кто больше не пьет (употребляли алкоголь раньше, но не в последние 12 месяцев). Вообще никогда не пившие отвечают на MOTV.AGST]***

…EFCT Употребление алкоголя воздействует на людей различным образом. Мы хотели бы узнать, какое воздействие оно могло оказывать на Вас. Когда Вы выпивали, насколько следующие утверждения были справедливы для Вас - очень часто, часто, иногда, редко или никогда?

|  | **Когда Вы пили спиртные напитки, насколько были справедливы для Вас следующие утверждения …** | **1 – ОЧЕНЬ ЧАСТО** | **2 – ЧАСТО** | **3 – ИНОГДА** | **4 – РЕДКО** | **5 – НИКОГДА** | **98 – НЕ ЗНАЮ** | **99 – ОТКАЗ** |
| --- | --- | --- | --- | --- | --- | --- | --- | --- |
| _1 | Вы расслабляетесь? |  |  |  |  |  |  |  |
| _2 | Вы чувствали себя счастливым? |  |  |  |  |  |  |  |
| _3 | Вы становились более агрессивным к окружающим? |  |  |  |  |  |  |  |
| _4 | Вы чувствовали себя более дружелюбным? |  |  |  |  |  |  |  |
| _5 | Было проще говорить о своих чувствах и проблемах? |  |  |  |  |  |  |  |
| _6 | Вы забывали о своих проблемах? |  |  |  |  |  |  |  |
| _7 | Вы делали что-то, о чем позже сожалели? |  |  |  |  |  |  |  |
| _8 | Вы получали большее удовольствие от секса? |  |  |  |  |  |  |  |
| _9 | Вы чувствовали себя более сексуально привлекательным? |  |  |  |  |  |  |  |
| _10 | У Вас случались проблемы с полицией? |  |  |  |  |  |  |  |
| _11 | Вам бывало очень весело? |  |  |  |  |  |  |  |
| _12 | Вы плохо себя чувствовали? |  |  |  |  |  |  |  |
| _13 | Не могли вспомнить, что происходило (провалы в памяти)? |  |  |  |  |  |  |  |

***[Этот вопрос для тех, кто больше не пьет (употребляли алкоголь раньше, но не за последние 12 месяцев). Вообще никогда не пившие отвечают на MOTV.AGST]***

…MOTV.FOR У людей бывают различные причины, чтобы употреблять алкоголь. Насколько важны приведенные причины лично для Вас? Можно сказать, что для Вас это очень важно, важно, не очень важно или совсем не важно?

|  |  | **1 – ОЧЕНЬ ВАЖНО** | **2 – ВАЖНО** | **3 – НЕ ОЧЕНЬ ВАЖНО** | **4 – СОВСЕМ НЕ ВАЖНО** | **98 – НЕ ЗНАЮ** | **99 – ОТКАЗ** |
| --- | --- | --- | --- | --- | --- | --- | --- |
| _1 | Чтобы быть более коммуникабельным и вежливым? |  |  |  |  |  |  |
| _2 | Потому что все остальные пьют? |  |  |  |  |  |  |
| _3 | Чтобы получать большее удовольствие от еды? |  |  |  |  |  |  |
| _4 | Для здоровья? |  |  |  |  |  |  |
| _5 | Чтобы хорошо себя чувствовать? |  |  |  |  |  |  |
| _6 | Чтобы помочь себе расслабиться? |  |  |  |  |  |  |
| _7 | Чтобы забыть о неприятностях? |  |  |  |  |  |  |
| _8 | Не чувствовать себя замкнутым и стеснительным? |  |  |  |  |  |  |
| _9 | Чтобы что-то отметить? |  |  |  |  |  |  |
| _10 | Потому, что нравится вкус? |  |  |  |  |  |  |
| _11 | Чтобы утолить жажду? |  |  |  |  |  |  |

***[Этот вопрос и для тех, кто больше не пьет, и для тех, кто никогда не пил]***

…MOTV.AGST У людей может быть целый ряд причин, чтобы ограничить своё потребление спиртных напитков или не пить вообще. Как Вы считаете, насколько важны приведенные причины лично для Вас? Можно сказать, что для Вас это очень важно, важно, не очень важно или совсем не важно?

|  |  | **1 – ОЧЕНЬ ВАЖНО** | **2 – ВАЖНО** | **3 – НЕ ОЧЕНЬ ВАЖНО** | **4 – СОВСЕМ НЕ ВАЖНО** | **98 – НЕ ЗНАЮ** | **99 – ОТКАЗ** |
| --- | --- | --- | --- | --- | --- | --- | --- |
| _1 | (Пропустить, если мужчина) Если Вы беременны/хотите забеременеть? |  |  |  |  |  |  |
| _2 | Вам не нравится вкус? |  |  |  |  |  |  |
| _3 | Вам не нравится, как на Вас действует спиртное? |  |  |  |  |  |  |
| _4 | Вам приходилось видеть плохие последствия алкоголя? |  |  |  |  |  |  |
| _5 | Потому что пьянство другого человека причинило Вам горе? |  |  |  |  |  |  |
| _6 | Пьянство может отрицательно сказаться на Вашей работе или учебе? |  |  |  |  |  |  |
| _7 | Пьянство слишком дорого или является пустой тратой денег? |  |  |  |  |  |  |
| _8 | По религиозным причинам? |  |  |  |  |  |  |
| _9 | Потому что Вы так воспитаны? |  |  |  |  |  |  |
| _10 | У Вас были проблемы со спиртным, и Вы боитесь стать алкоголиком? |  |  |  |  |  |  |
| _11 | Потому что Вы слишком молоды? |  |  |  |  |  |  |
| _12 | Ваши друзья и (или) члены семьи не одобряют Ваше пьянство? |  |  |  |  |  |  |
| _14 | Из-за здоровья? |  |  |  |  |  |  |
| _15 | Вам это просто не интересно? |  |  |  |  |  |  |

# МОДУЛЬ 17: Восприятие алкоголя и других веществ опрашиваемым и окружающими (PCPN)

...GEN Укажите, в какой степени Вы согласны или не согласны со следующими утверждениями. *(Показать карточку)*

...GEN_1 Выпивка – это одна из радостей жизни.

1 – СОВЕРШЕННО согласен

2 – согласен

3 – НИ ТО, НИ ДРУГОЕ

4 – НЕ согласен

5 – СОВЕРШЕННО НЕ СОГЛАСЕН

98 – НЕ ЗНАЮ 99 – ОТКАЗ

...GEN_2 Выпивая с кем-то, демонстрируешь ему или ей свои дружеские чувства.

1 – СОВЕРШЕННО согласен

2 – согласен

3 – НИ ТО, НИ ДРУГОЕ

4 – НЕ согласен

5 – СОВЕРШЕННО НЕ СОГЛАСЕН

98 – НЕ ЗНАЮ 99 – ОТКАЗ

...GEN_3 Про выпивку нельзя сказать ничего хорошего.

1 – СОВЕРШЕННО согласен

2 – согласен

3 – НИ ТО, НИ ДРУГОЕ

4 – НЕ согласен

5 – СОВЕРШЕННО НЕ СОГЛАСЕН

98 – НЕ ЗНАЮ 99 – ОТКАЗ

…SITS Далее я приведу примеры различных жизненных ситуаций, в которых бывают люди. Пожалуйста, сообщите по каждой из них своё мнение: сколько в каждой из них человеку допустимо выпить: не пить вообще; выпить столько, чтобы не чувствовать эффекта (1-2 порции); столько, чтобы почувствовать опьянение, но не напиться; иногда можно и напиться; напиться всегда приемлемо. *(Показать карточку)*

|  |  | **1 – Не пить вообще** | **2 –чтобы не ПОчувство­вать эффекта (1-2 порции)** | **3 –почувствовать опьяне­ние, но не напиться** | **4 – Иногда можно и напиться** | **5 – Напиться всегда приемлемо** | **98 – НЕ ЗНАЮ** | **99 – ОТКАЗ** |
| --- | --- | --- | --- | --- | --- | --- | --- | --- |
| _2 | Мать, проводящая время с маленькими детьми |  |  |  |  |  |  |  |
| _3 | Отец, проводящий время с маленькими детьми |  |  |  |  |  |  |  |
| _6 | Мужчина, встречающийся в баре с друзьями |  |  |  |  |  |  |  |
| _7 | Женщина, встречающаяся в баре с подругами |  |  |  |  |  |  |  |
| _8 | Женщина, вечером вне дома с коллегами по работе |  |  |  |  |  |  |  |
| _9 | Мужчина, вечером вне дома с коллегами по работе |  |  |  |  |  |  |  |
| _12 | Мужчина, ужинающий дома с женой/партнером |  |  |  |  |  |  |  |
| _13 | Женщина, ужинающая дома с мужем/партнером |  |  |  |  |  |  |  |

# МОДУЛЬ 20: Подростки и молодые взрослые (ADYA)

## 20.3 Взрослость

EMAD.PERC Пожалуйста, сообщите мне, в какой степени Вы согласны или не согласны с каждый из приведенных ниже утверждений. *(Показать карточку)*

...EMAD.PERC_1 Вы уже взрослый.

1 – СОВЕРШЕННО согласен

2 – согласен

3 – НИ ТО, НИ ДРУГОЕ

4 – НЕ согласен

5 – СОВЕРШЕННО НЕ СОГЛАСЕН

98 – НЕ ЗНАЮ 99 – ОТКАЗ

...EMAD.PERC_2 Вы финансово независимы от своих родителей.

1 – СОВЕРШЕННО согласен

2 – согласен

3 – НИ ТО, НИ ДРУГОЕ

4 – НЕ согласен

5 – СОВЕРШЕННО НЕ СОГЛАСЕН

98 – НЕ ЗНАЮ 99 – ОТКАЗ

...EMAD.PERC_3 Вы эмоционально независимы от своих родителей или попечителей.

1 – СОВЕРШЕННО согласен

2 – согласен

3 – НИ ТО, НИ ДРУГОЕ

4 – НЕ согласен

5 – СОВЕРШЕННО НЕ СОГЛАСЕН

98 – НЕ ЗНАЮ 99 – ОТКАЗ

…STOP  *СТОП. Зафиксируйте время:*

__ __ : __ __ (ЧЧ:ММ, 24-часовой формат)

# МОДУЛЬ 21: Привлечение к исследованию и скрининг (RCRT)

Мы заканчиваем интервью, и мне хотелось бы задать Вам еще несколько вопросов.

…ENG1 Оцените по шкале от 1 до 10, насколько интересным для Вас было участие в исследовании. 1 – не интересно, 10 – крайне интересно?

__ __

98 – НЕ ЗНАЮ 99 – ОТКАЗ

…ENG2 По такой же шкале от 1 до 10: насколько Вам понравилось участвовать в исследовании?

__ __

98 – НЕ ЗНАЮ 99 – ОТКАЗ

…SCRN... И, наконец, несколько завершающих вопросов перед окончанием интервью:

…SCRN.STDN Если считать «учащимся» человека, который в настоящий момент проходит какое-либо обучение или подготовку, что в наилучшей степени описывает Вашу ситуацию:

1 – Я – учащийся дневного отделения

2 – Я – заочник или учащийся вечернего отделения

3 – Я СЕЙЧАС не учусь (Перейти к …SCREEN.INT)

98 – НЕ ЗНАЮ 99 – ОТКАЗ

…SCRN.EDUC Как наилучшим образом можно описать Вашу учебу в настоящее время:

1 – СреднЕЕ ОБРАЗОВАНИЕ

2 – Профессиональное ОБРАЗОВАНИЕ

3 – Высшее ОБРАЗОВАНИЕ

4 – ДРУГОЕ (укажите) _______________________________________

98 – НЕ ЗНАЮ 99 – ОТКАЗ

…SCRN.INT Обычно как часто Вы пользуетесь интернетом?

1 – ЕЖЕДНЕВНО

2 – ЕЖЕНЕДЕЛЬНО

3 – ЕЖЕМЕСЯЧНО

4 – РЕЖЕ, ЧЕМ РАЗ В МЕСЯЦ

5 – НИКОГДА

6 – У МЕНЯ НЕТ ДОСТУПА В ИНТЕРНЕТ

98 – НЕ ЗНАЮ 99 – ОТКАЗ

…SCRN.CHCK Обычно как часто Вы проверяете свою электронную почту?

1 – ЕЖЕДНЕВНО

2 – ЕЖЕНЕДЕЛЬНО

3 – ЕЖЕМЕСЯЧНО

4 – РЕЖЕ, ЧЕМ РАЗ В МЕСЯЦ

5 – НИКОГДА

6 – У МЕНЯ НЕТ АДРЕСА ЭЛЕКТРОННОЙ ПОЧТЫ

98 – НЕ ЗНАЮ 99 – ОТКАЗ

(ЕСЛИ SCRN.STDN=3 (сейчас не учится), ТО ЗАВЕРШЕНИЕ)

(ЕСЛИ SCRN.INT = 5 ИЛИ 6 (никогда не пользуется интернетом или нет доступа к интернету) **И (ИЛИ)** SCRN.CHCK = 5 ИЛИ 6 (никогда не проверяет электронную почту или не имеет адреса электронной почты), **ТО** ЗАВЕРШЕНИЕ)

В ИНОМ СЛУЧАЕ ПРОДОЛЖИТЬ

К я уже говорил(а) в начале беседы, для успеха этого исследования очень получить ответы людей с различными отношениями, представлениями и опытом – спасибо Вам. Мы надеемся, что многие из тех, кто принял участие в этом опросе, рассмотрят возможность принять участие во втором его этапе. Второй этап исследования включает в себя ответы на вопросы анонимного онлайн-опросника. Если Вы желаете принять участие в онлайн-опросе, я дам вам адрес сайта исследования.

...PART Хотели бы Вы принять участие во второй части исследования?

1 – ДА, МНЕ БЫЛО БЫ ИНТЕРЕСНО ПРИНЯТЬ УЧАСТИЕ ВО 2-Й ЧАСТИ ИССЛЕДОВАНИЯ (ДАТЬ АДРЕС САЙТА ИССЛЕДОВАНИЯ, ПОСЛЕ ЭТОГО - ЗАВЕРШЕНИЕ)

2 – НЕТ, МЕНЯ НЕ ИНТЕРЕСУЕТ УЧАСТИЕ ВО 2-Й ЧАСТИ ИССЛЕДОВАНИЯ

98 – НЕ ЗНАЮ 99 – ОТКАЗ

(ЗАДАТЬ, ЕСЛИ .PART=2)

 …PART.REF Не могли бы Вы сказать мне, почему Вы не желаете участвовать?

_________________________________________________________ (открытый вопрос)

98 – НЕ ЗНАЮ 99 – ОТКАЗ

[СПАСИБО И ЗАВЕРШЕНИЕ]
