# Supplementary material for: Context and culture associated with alcohol use amongst youth in major urban cities: A cross-country population based survey
Source: PLoS One. 2017 Nov 20;12(11):e0187812. doi: 10.1371/journal.pone.0187812 (PMC5695777; doi:10.1371/journal.pone.0187812)
Supplement: S6 Questionnaire — (DOCX) [file pone.0187812.s006.docx]

**
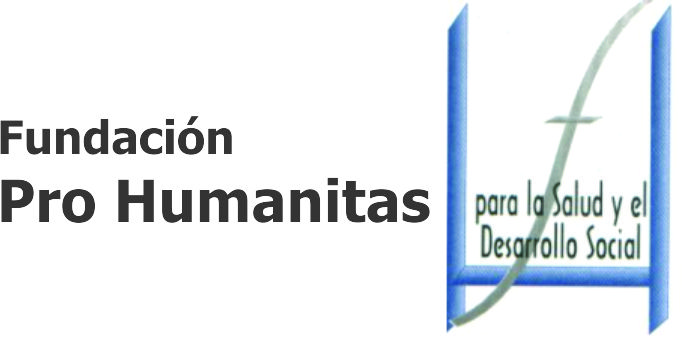

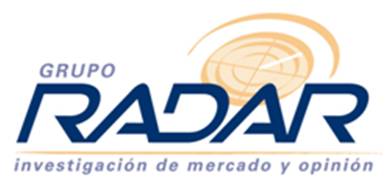
**

Nº Formulario: |__|__|__|__|

PM: |__|__|__|

**Life Transitions and Alcohol Consumption in Context (LTACC) – Uruguay.**

**International Center for Alcohol Policies – Fundación Pro Humanitas**

**Módulo A: Identificación de hogares, consentimiento, y variables administrativas (ADMN)**

***A.0 Identificación del hogar y encuestador. Fecha (completar antes de acercarse a la casa / encuestado)***

***LEV1… Ciudad / área metropolitana: ___ ___ (Ver Libro de códigos)***

***LEV2 ... Barrio o localidad de Montevideo, Canelones o San José ___ ___ (Ver Libro de códigos)***

***LEV3 ... N° de manzana: ___ ___***

***LEV4 ... Calle__________________________ Entre________________________ y ___________________________***

***N° de puerta. __________ / Apto. ___________***

***N° de encuestador: ___ ___ ___ ___***

***FECHA: __ __ (dd) __ __ (mm) __ __ __ __ (aaaa)***

***A.1 Disposición del hogar y el demandado (completar después del contacto con el hogar/encuestado).***

*…HDIS* Disposición del hogar:

___ ___ ***(Ver libro de códigos)***

*…RDIS* Disposición del encuestado:

___ ___ ***(Ver libro de códigos)***

*…RDIS_TXT* Motivo de la terminación anticipada de la entrevista, en su caso (es decir, ADMN.RDIS = 25 o 26)

________________________________________________________________________

________________________________________________________________________

***A.2 Censo del hogar. Determinación de elegibilidad del encuestado.***

*...INTR* Hola, mi nombre es ________________________. Soy un encuestador profesional que trabaja con Fundación Pro Humanitas como parte de una investigación financiado por el Centro Internacional de Políticas sobre Alcohol (ICAP). Tu hogar fue seleccionado al azar para ser incluido en nuestro estudio sobre consumo de bebidas alcohólicas y otras drogas. ¿Puedo hacerle algunas preguntas para determinar si alguna de las personas que viven en este hogar es elegible para participar en el estudio?

Después de obtener el consentimiento:

Muchas gracias. ¿Me puede decir la edad y el sexo de cada una de las personas que viven actualmente en este hogar?

***Encuestador:*** Complete las columnas 1 y 2 de la matriz de abajo introduciendo las edades y rodeando el género correcto. Por cada persona que se menciona que tiene entre 18 y 34 años de edad, pregunte si él/ella ha vivido en Montevideo y su área metropolitana durante los últimos 6 meses y completa la columna 3 según corresponda.

Censo de Hogares

|  | **Edad** | **Sexo** | **¿Ha vivido en Montevideo o su área metropolitana durante los últimos 6 meses?** |  |  | **Edad** | **Sexo** | **¿Ha vivido en Montevideo o su área metropolitana durante los últimos 6 meses?** |
| --- | --- | --- | --- | --- | --- | --- | --- | --- |
| **Persona 1** |  | (1) M / (2) F | (1) Si / (2) No |  | **Persona 7** |  | (1) M / (2) F | (1) Si / (2) No |
| **Persona 2** |  | (1) M / (2) F | (1) Si / (2) No |  | **Persona 8** |  | (1) M / (2) F | (1) Si / (2) No |
| **Persona 3** |  | (1) M / (2) F | (1) Si / (2) No |  | **Persona 9** |  | (1) M / (2) F | (1) Si / (2) No |
| **Persona 4** |  | (1) M / (2) F | (1) Si / (2) No |  | **Persona 10** |  | (1) M / (2) F | (1) Si / (2) No |
| **Persona 5** |  | (1) M / (2) F | (1) Si / (2) No |  | **Persona 11** |  | (1) M / (2) F | (1) Si / (2) No |
| **Persona 6** |  | (1) M / (2) F | (1) Si / (2) No |  | **Persona 12** |  | (1) M / (2) F | (1) Si / (2) No |

***Persona seleccionada Nº: _____***

***Encuestador:*** *Si no hay encuestado elegible, pasar al siguiente hogar. Si existen varios encuestados elegibles, seleccione uno utilizando el método del último cumpleaños y pida hablar con la persona seleccionada. Saltar a A.1 y completar las siguientes preguntas sólo después de entrevistar (o intentar entrevistar a) la persona seleccionada.*

# ***A.3 Introducción y consentimiento del encuestado.***

...INST Hola, mi nombre es ________________________. Soy un encuestador profesional que trabaja con Fundación Pro Humanitas como parte de una investigación financiado por el Centro Internacional de Políticas sobre Alcohol (ICAP). Tu hogar fue seleccionado al azar para ser incluido en nuestro estudio sobre consumo de bebidas alcohólicas y otras drogas.

La participación en este estudio consiste en responder preguntas acerca de tu salud, tus comportamientos y opiniones acerca del consumo de bebidas alcohólicas y otras drogas. La encuesta dura de 20 a 30 minutos. Tus respuestas son totalmente confidenciales y tu decisión de participar o no en este estudio es completamente voluntaria. Si alguna pregunta te hace sentir incómodo, podemos omitirla. También eres libre de terminar la entrevista en cualquier momento. ¿Estás de acuerdo en participar en esta encuesta?

____________________

Iniciales del entrevistador, lo que indica

consentimiento verbal del entrevistado

***A.4 Asuntos Administrativos***

*...INST2* *Gracias.* Antes de comenzar, me gustaría hacerte algunas preguntas para asegurarme de que sos elegible para participar en este estudio.

*...BYR*

| 1. **¿En qué año naciste?** |  |
| --- | --- |
| *98 - NO SABE* |  |
| *99 - NO CONTESTA* |  |

***Encuestador:*** Determine si, en base al año de nacimiento, el demandado puede ser menor de 18 años (nacido en 1996 o después), o más de 34 años (nacidos en 1980 o antes). La entrevista termina si el entrevistado no está en el rango de edad de 18 a 34 años, o la edad no se puede determinar.

*…RESI* ¿Has vivido en Montevideo o su área metropolitana durante los últimos 6 meses?

1 - SI

2 - NO

98 - NO SABE

99 - RECHAZADO

***Encuestador:*** La entrevista termina si el encuestado no ha residido en Montevideo o su área metropolitana durante los últimos 6 meses. [Encuestado Código como "no elegible".]

***A.5 Introducción a la Entrevista***

…INST3 Si estás de acuerdo, voy a comenzar la entrevista. Recuerda que tus respuestas se mantendrán en la más estricta confidencialidad, así que puedes responder a cada pregunta con total honestidad. Si alguna pregunta te hace sentir incómodo, o no sabes la respuesta, por favor dímelo y podemos pasar a la siguiente pregunta.

*…STRT* Registre la hora actual _____ : _____ (HH:MM, 24:00 clock)

| 1. **SEXO (Registre según su observación. Pregunte si es necesario.)** | |
| --- | --- |
| 1 - Hombre | |
| 2 - Mujer | |
| 98 - NO SABE | |
| 99– NO CONTESTA | |
| 1. **¿Cuál de estas categorías describe mejor tu estado civil?** | |
| 1 - casado | |
| 2 – unión libre | |
| 3 - divorciado o separado | |
| 4 – viudo | |
| 5– nunca se ha casado ***(Pasar a pregunta 5)*** | |
| 98 - NO SABE | |
| 99– NO CONTESTA | |
| 1. **¿Desde qué año te encuentras [Casado, en unión libre, divorciado o separado / viudo]?** | |
| AÑO: |  |
| 98 – NO SABE | |
| 99– NO CONTESTA | |

| MÓDULO 1: Inicio del consumo y período de mayor consumo. | | |
| --- | --- | --- |
| 1. **¿Alguna vez consumiste alguna bebida alcohólica (por ejemplo vino, whisky, cerveza, licor, vermut, sidra, grappa, grappamiel, vodka, ron, u otra)? Por favor, no incluyas las veces que sólo tomaste uno o dos sorbos de la bebida de otra persona.** | |  |
| 1 - SÍ | |  |
| 2 - NO ***(Pasar a pregunta 31 )*** | |  |
| 98 - NO SABE | |  |
| 99 - NO CONTESTA | |  |
| 1. **¿Qué edad tenías la primera vez que tomaste una bebida alcohólica? Por favor, no incluyas las veces en que sólo tomaste uno o dos sorbos de una bebida.** | |  |
|  | AÑOS |  |
| 98 - NO SABE | |  |
| 99 - NO CONTESTA | |  |
| 1. **¿Qué edad tenías la primera vez que te emborrachaste?** | |  |
|  | AÑOS |  |
| 97 - NUNCA me he emborrachado | |  |
| 98 - NO SABE | |  |
| 99 - NO CONTESTA | |  |
| 1. **Estamos interesados ​​en el período de tu vida en el que más bebiste alcohol. ¿Qué edad dirías que tenías cuando comenzó el período de mayor consumo de bebidas alcohólicas?** | |  |
|  | AÑOS |  |
| 98 - NO SABE | |  |
| 99 - NO CONTESTA | |  |
| 1. **¿Y qué edad tenías cuando terminó este período?** | |  |
|  | AÑOS |  |
| 97 - NO HA TERMINADO | |  |
| 98 - NO SABE | |  |
| 99 - NO CONTESTA | |  |

# MÓDULO 2: Consumo de alcohol en los últimos 12 meses.

# 2.1 Generalidades sobre el consumo de alcohol.

| 1. **Durante los últimos 12 meses, ¿con qué frecuencia tomaste bebidas alcohólicas, incluso en pequeñas cantidades? (por ejemplo vino, whisky, cerveza, licor, vermut, sidra, grappa, grappamiel, vodka, ron, u otra) *(MOSTRAR TARJETA)*** |
| --- |
| 1 - todos los días |
| 2- 5 a 6 veces a la semana |
| 3 - 3 a 4 veces por semana |
| 4 - 1 a 2 veces por semana |
| 5 - 2 a 3 veces al mes |
| 6 – 1 vez al mes |
| 7 - 6 a 11 veces en los últimos 12 meses |
| 8 - 2 a 5 veces en los últimos 12 meses |
| 9 - 1 vez en los últimos 12 meses ***(Pasar a pregunta 29 sólo si tanto pregunta 10 = 9 y pregunta 11 = 11)*** |
| 10 - No tome nada de alcohol en los últimos 12 meses ***(Pasar a pregunta 29)*** |
| 98 - NO SABE |
| 99 - NO CONTESTA |
|  |
| 1. **Durante los últimos 12 meses, ¿cuánto tomaste en un día típico en que bebiste alcohol? Por un trago nos referimos a una copa o vaso de vino (140 ml), una lata de cerveza (335 ml), una medida de whisky u otras bebidas destiladas (50 ml). *(MOSTRAR LA TARJETA GRÁFICA Y LA ILUSTRACIÓN)*** |
| 1- 25 a 35 tragos |
| 2- 19 a 24 tragos |
| 3- 16 a 18 tragos |
| 4- 12 a 15 tragos |
| 5- 9 a 11 tragos |
| 6- 7 a 8 tragos |
| 7- 5 a 6 tragos |
| 8- 3 a 4 tragos |
| 9- 2 tragos |
| 10- 1 trago |
| 11- Menos de 1 trago completo ***(Pasar a pregunta 29 sólo si tanto pregunta 10 = 9 y pregunta 11 = 11)*** |
| 98 - NO SABE |
| 99 - NO CONTESTA |
| 1. **Durante los últimos 12 meses, ¿cuál es la máxima cantidad de tragos de bebidas alcohólicas que tomaste en un plazo de 24 horas? Por un trago nos referimos a una copa o vaso de vino (140 ml), una lata de cerveza (335 ml), una medida de whisky u otras bebidas destiladas (50 ml). *(MOSTRAR TARJETA)*** |
| 1- 36 ó más tragos |
| 2- 25 a 35 tragos |
| 3- 19 a 24 tragos |
| 4- 16 a 18 tragos |
| 5- 12 a 15 tragos |
| 6- 9 a 11 tragos |
| 7- 7 a 8 tragos |
| 8- 5 a 6 tragos |
| 9- 3 a 4 tragos |
| 10- 2 tragos |
| 11- 1 trago |
| 12- Menos de 1 trago completo |
| 98 - NO SABE |
| 99 - NO CONTESTA |
| 1. **Durante los últimos 12 meses, ¿con qué frecuencia has tomado (*insertar respuesta a pregunta 12*) en un plazo de 24 horas? *(MOSTRAR TARJETA)*** |
| 1 - todos los días |
| 2 - 5 a 6 veces por semana |
| 3 - 3 a 4 veces por semana |
| 4 - 1 a 2 veces por semana |
| 5 - 2 a 3 veces al mes |
| 6 - 1 vez al mes |
| 7 - 6 a 11 veces en los últimos 12 meses |
| 8 - 2 a 5 veces en los últimos 12 meses |
| 9 - 1 vez en los últimos 12 meses |
| 98 - NO SABE |
| 99 - NO CONTESTA |

# 2.2 Bebidas Específicas, Cantidad / Frecuencia

# Ahora me gustaría preguntarte con qué frecuencia tomaste algunas bebidas alcohólicas durante los últimos 12 meses, y la cantidad que usualmente tomaste de cada una en un día típico.

| 1. **¿Con qué frecuencia bebiste cerveza con alcohol en los últimos 12 meses? *(MOSTRAR TARJETA)*** | | |
| --- | --- | --- |
| 1 - todos los días | | |
| 2 - 5 a 6 veces por semana | | |
| 3 - 3 a 4 veces por semana | | |
| 4 - 1 a 2 veces por semana | | |
| 5 - 2 a 3 veces al mes | | |
| 6 - 1 vez al mes | | |
| 7 - 6 a 11 veces en los últimos 12 meses | | |
| 8 - 2 a 5 veces en los últimos 12 meses | | |
| 9 - 1 vez en los últimos 12 meses | | |
| 10 - nunca en los últimos 12 meses ***(Pasar a pregunta 16)*** | | |
| 98 - NO SABE | | |
| 99 - NO CONTESTA | | |
| 1. **Y en un día típico en el que bebiste cerveza con alcohol, ¿qué cantidad de cerveza tomaste?** | | |
| CANTIDAD DE TRAGOS= una lata de cerveza =335 ml | |  |
| 98 - NO SABE | | |
| 99 - NO CONTESTA | | |
| 1. **¿Con qué frecuencia bebiste vino (incluyendo champagne y espumantes) en los últimos 12 meses? *(MOSTRAR TARJETA)*** | | |
| 1 - todos los días | | |
| 2 - 5 a 6 veces por semana | | |
| 3 - 3 a 4 veces por semana | | |
| 4 - 1 a 2 veces por semana | | |
| 5 - 2 a 3 veces al mes | | |
| 6 – 1 vez al mes | | |
| 7 - 6 a 11 veces en los últimos 12 meses | | |
| 8 - 2 a 5 veces en los últimos 12 meses | | |
| 9 - 1 vez en los últimos 12 meses | | |
| 10 - nunca en los últimos 12 meses ***(Pasar a pregunta 18)*** | | |
| 98 - NO SABE | | |
| 99 - NO CONTESTA | | |
| 1. **Y en un día típico en el que bebiste vino (incluyendo champagne y espumantes), ¿qué cantidad vino tomaste?** | | |
| CANTIDAD DE TRAGOS= una copa o un vaso=140 ml | |  |
| 98 - NO SABE | | |
| 99 - NO CONTESTA | | |
|  | | |
| 1. **¿Con qué frecuencia tomaste bebidas espirituosas (destiladas: whisky, vodka, ron, caña, grappa, etc.) en los últimos 12 meses? (Mostrar tarjeta)** | | |
| 1 - todos los días | | |
| 2 - 5 a 6 veces por semana | | |
| 3 - 3 a 4 veces por semana | | |
| 4 - 1 a 2 veces por semana | | |
| 5 - 2 a 3 veces al mes | | |
| 6 - 1 vez al mes | | |
| 7 - 6 a 11 veces en los últimos 12 meses | | |
| 8 - 2 a 5 veces en los últimos 12 meses | | |
| 9 - 1 vez en los últimos 12 meses | | |
| 10 - nunca en los últimos 12 meses ***(Pasar a pregunta 20)*** | | |
| 98 - NO SABE | | |
| 99 - NO CONTESTA | | |
| 1. **Y en un día típico en el que tomaste bebidas destiladas, ¿cuántos tragos bebidas destiladas tomaste?** | | |
| CANTIDAD DE TRAGOS = una medida |  | |
| 98 - NO SABE | | |
| 99 - NO CONTESTA | | |

# 2.3 Experiencia de embriaguez / intoxicación

| 1. **¿Cuántas veces en los últimos 12 meses, tomaste lo suficiente para sentirte intoxicado o borracho - ya sea que te sentiste inestable al caminar, tu visión se volvió borrosa, o tu habla se distorsionó? *(MOSTRAR TARJETA)*** | |
| --- | --- |
| 1 - todos los días | |
| 2 - 5 a 6 veces por semana | |
| 3 - 3 a 4 veces por semana | |
| 4 - 1 a 2 veces por semana | |
| 5 - 2 a 3 veces al mes | |
| 6 - 1 vez al mes | |
| 7 - 6 a 11 veces en los últimos 12 meses | |
| 8 - 2 a 5 veces en los últimos 12 meses | |
| 9 - 1 vez en los últimos 12 meses | |
| 10 - nunca en los últimos 12 meses ***(Pasar a pregunta 22)*** | |
| 98 - NO SABE | |
| 99 - NO CONTESTA | |
| 1. **¿Cuántos tragos te hacen sentir borracho o embriagado?** | |
| (1 trago equivale a una lata de cerveza, una copa o vaso de vino, o una medida de whisky u otra bebida destilada) |  |
| 98 - NO SABE | |
| 99 - NO CONTESTA | |

# 2.4 Beber en Contexto. Ahora voy a preguntarte con qué frecuencia bebes alcohol en situaciones específicas.

| 1. **Durante los últimos 12 meses, ¿con qué frecuencia tomaste alcohol cuando ... (*MOSTRAR TARJETA*)** | | | | | | | | | | | |
| --- | --- | --- | --- | --- | --- | --- | --- | --- | --- | --- | --- |
|  |  | Todos los días o casi todos los días | Por lo menos una vez a la semana | Al menos una vez al mes pero menos de una vez por semana | Al menos una vez en los últimos 12 meses pero menos de una vez al mes | Nunca durante los últimos 12 meses | NO SABE | NO CONTESTA | 1. **¿Y cuánto tomaste ? (NO PREGUNTAR SI EN 22 CONTESTÓ 5)** | | |
|  | **22.1- Cenabas en un restaurante?** | 1 | 2 | 3 | 4 | 5 | 98 | 99 | **23.1** |  | |
|  |  |  |  |  |  |  |  |  |  | 98 | 99 |
|  | **22.2- Almorzabas en un restaurante?** | 1 | 2 | 3 | 4 | 5 | 98 | 99 | **23.2** |  | |
|  |  |  |  |  |  |  |  |  |  | 98 | 99 |
|  | **22.3- En bares, cantinas o pubs?** | 1 | 2 | 3 | 4 | 5 | 98 | 99 | **23.3** |  | |
|  |  |  |  |  |  |  |  |  |  | 98 | 99 |
|  | **22.4- Estando en casa de otra persona? (incluyendo fiestas)** | 1 | 2 | 3 | 4 | 5 | 98 | 99 | **23.4** |  | |
|  |  |  |  |  |  |  |  |  |  | 98 | 99 |
|  | **22.5- Estando tranquilo en tu casa a la noche?** | 1 | 2 | 3 | 4 | 5 | 98 | 99 | **23.5** |  | |
|  |  |  |  |  |  |  |  |  |  | 98 | 99 |
|  | **22.6- Con amigos que te visitan en tu casa?** | 1 | 2 | 3 | 4 | 5 | 98 | 99 | **23.6** |  | |
|  |  |  |  |  |  |  |  |  |  | 98 | 99 |
|  | **22.7- Estando con amigos en un lugar público, como un parque, una plaza, la rambla, la playa o la calle?** | 1 | 2 | 3 | 4 | 5 | 98 | 99 | **23.7** |  | |
|  |  |  |  |  |  |  |  |  |  | 98 | 99 |

| 1. **Durante los últimos 12 meses, ¿qué cantidad de tu consumo de alcohol se produjo durante las comidas?** |  | 1. **Durante los últimos 12 meses, ¿qué cantidad de tu consumo de alcohol se produjo estando solo?** |
| --- | --- | --- |
| 1 - todo o casi todo |  | 1 - todo o casi todo |
| 2 - más de la mitad |  | 2 - más de la mitad |
| 3 - la mitad |  | 3 - la mitad |
| 4 - menos de la mitad |  | 4 - menos de la mitad |
| 5 - nada o casi nada |  | 5 - nada o casi nada |
| 98 - NO SABE |  | 98 - NO SABE |
| 99 – NO CONTESTA |  | 99 – NO CONTESTA |

# MÓDULO 3: Motivaciones a favor y en contra de beber alcohol y sus efectos. (*SÓLO PARA LOS BEBEDORES ACTUALES. LOS NO BEBEDORES Y LOS EX BEBEDORES PASAR A PREGUNTA 29*)

| 1. **El consumo de alcohol afecta a las personas de muchas maneras diferentes. Nos gustaría saber qué efectos puede tener la bebida en vos. Cuando tomas alcohol, qué tan ciertas dirías que son cada una de las siguientes afirmaciones. *(MOSTRAR TARJETA)*. ¿Con que frecuencia es cierto que cuando tomas alcohol ...** | | | | | | | | |  |
| --- | --- | --- | --- | --- | --- | --- | --- | --- | --- |
|  |  | Casi siempre es cierto | Con frecuencia es cierto | A veces es cierto | Raras veces es cierto | Nunca es cierto | NO SABE | NO CONTESTA |  |
|  | | **26.1- Te sientes relajado (flojo, distendido)?** | 1 | 2 | 3 | 4 | 5 | 98 | 99 |
|  | | **26.2- Te sientes feliz?** | 1 | 2 | 3 | 4 | 5 | 98 | 99 |
|  | | **26.3- Te vuelves más agresivo hacia otras personas?** | 1 | 2 | 3 | 4 | 5 | 98 | 99 |
|  | | **26.4- Te sientes más amable y abierto?** | 1 | 2 | 3 | 4 | 5 | 98 | 99 |
|  | | **26.5- Te resulta más fácil hablar acerca de tus sentimientos o problemas?** | 1 | 2 | 3 | 4 | 5 | 98 | 99 |
|  | | **26.6- Te olvidas de tus problemas?** | 1 | 2 | 3 | 4 | 5 | 98 | 99 |
|  | | **26.7- Haces algo de lo que te arrepientes?** | 1 | 2 | 3 | 4 | 5 | 98 | 99 |
|  | | **26.8- La actividad sexual te resulta más placentera?** | 1 | 2 | 3 | 4 | 5 | 98 | 99 |
|  | | **26.9- Te sientes más atractivo sexualmente?** | 1 | 2 | 3 | 4 | 5 | 98 | 99 |
|  | | **26.10- Te metes en problemas con la policía?** | 1 | 2 | 3 | 4 | 5 | 98 | 99 |
|  | | **26.11- Te diviertes mucho?** | 1 | 2 | 3 | 4 | 5 | 98 | 99 |
|  | | **26.12- Te sientes enfermo?** | 1 | 2 | 3 | 4 | 5 | 98 | 99 |
|  | | **26.13- No recuerdas lo que pasó?** | 1 | 2 | 3 | 4 | 5 | 98 | 99 |

| 1. **La gente tiene diferentes razones para beber alcohol. ¿Qué tan importante dirías que son cada una de las siguientes razones para vos? (*MOSTRAR TARJETA*)** | | | | | | | |
| --- | --- | --- | --- | --- | --- | --- | --- |
|  |  | Muy importante | Importante | No muy importante | Nada importante | No sabe | No contesta |
| **27.1- Ser sociable y educado?** | | 1 | 2 | 3 | 4 | 98 | 99 |
| **27.2- Debido a que los demás están bebiendo?** | | 1 | 2 | 3 | 4 | 98 | 99 |
| **27.3- Para agregar al disfrute de las comidas?** | | 1 | 2 | 3 | 4 | 98 | 99 |
| **27.4- Por razones de salud?** | | 1 | 2 | 3 | 4 | 98 | 99 |
| **27.5- Para sentirte bien?** | | 1 | 2 | 3 | 4 | 98 | 99 |
| **27.6- Para ayudarte a relajarte?** | | 1 | 2 | 3 | 4 | 98 | 99 |
| **27.7- Para olvidar las preocupaciones?** | | 1 | 2 | 3 | 4 | 98 | 99 |
| **27.8- Para ayudarte a sentirte menos inhibido o tímido?** | | 1 | 2 | 3 | 4 | 98 | 99 |
| **27.9- Para celebrar?** | | 1 | 2 | 3 | 4 | 98 | 99 |
| **27.10- Debido a su sabor?** | | 1 | 2 | 3 | 4 | 98 | 99 |
| **27.11- Para sacarte la sed?** | | 1 | 2 | 3 | 4 | 98 | 99 |

| 1. **Las personas también tienen diferentes razones por las que limitan su consumo de alcohol, o no beben en absoluto. ¿Qué tan importante dirías que son cada una de las siguientes razones para vos? *(MOSTRAR TARJETA)*** | | | | | | |
| --- | --- | --- | --- | --- | --- | --- |
|  | Muy importante | Importante | No muy importante | Nada importante | NO SABE | NO CONTESTA |
| **28.1- *(SOLO SI ES MUJER)* Porque usted estaba o está embarazada, o tratando de quedar embarazada?** | 1 | 2 | 3 | 4 | 98 | 99 |
| **28.2- Debido al sabor?** | 1 | 2 | 3 | 4 | 98 | 99 |
| **28.3- Porque no te gusta el efecto que tiene en vos?** | 1 | 2 | 3 | 4 | 98 | 99 |
| **28.4- Porque has visto malos ejemplos de lo que el alcohol puede hacer?** | 1 | 2 | 3 | 4 | 98 | 99 |
| **28.5- Debido a que has sido perjudicado por la bebida de otra persona?** | 1 | 2 | 3 | 4 | 98 | 99 |
| **28.6- Debido a que la bebida puede afectar tu rendimiento laboral o en los estudios?** | 1 | 2 | 3 | 4 | 98 | 99 |
| **28.7- Debido a que la bebida es demasiado cara o es una pérdida de dinero?** | 1 | 2 | 3 | 4 | 98 | 99 |
| **28.8- Debido a razones religiosas?** | 1 | 2 | 3 | 4 | 98 | 99 |
| **28.9- Porque te educaron para no beber?** | 1 | 2 | 3 | 4 | 98 | 99 |
| **28.10- Debido a que has tenido problemas con el alcohol o tienes miedo de convertirte en un alcohólico?** | 1 | 2 | 3 | 4 | 98 | 99 |
| **28.11- Debido a que sos demasiado joven?** | 1 | 2 | 3 | 4 | 98 | 99 |
| **28.12- Debido a que tus amigos y / o familiares desaprueban las bebidas alcohólicas?** | 1 | 2 | 3 | 4 | 98 | 99 |
| **28.13- Por razones de salud?** | 1 | 2 | 3 | 4 | 98 | 99 |
| **28.14- Debido a que no estás interesado?** | 1 | 2 | 3 | 4 | 98 | 99 |

# MÓDULO 4: Motivaciones a favor y en contra de beber alcohol y sus efectos. (*SÓLO PARA LOS NO BEBEDORES Y LOS EX -BEBEDORES. LOS BEBEDORES ACTUALES PASAR A PREGUNTA 32)*

| ***Pregunta 29 es para bebedores pasados (tomaron alcohol en el pasado pero no en los últimos 12 meses). Los que nunca tomaron alcohol pasan a la pregunta 31.***   1. **El consumo de alcohol afecta a las personas de muchas maneras diferentes. Nos gustaría saber qué efectos puede tener la bebida en vos. Cuando bebiste alcohol, qué tan ciertas dirías que son cada una de las siguientes afirmaciones. *(MOSTRAR TARJETA)*** | | | | | | | |  |
| --- | --- | --- | --- | --- | --- | --- | --- | --- |
|  | Casi siempre es cierto | Con frecuencia es cierto | A veces es cierto | Raras veces es cierto | Nunca es cierto | NO SABE | NO CONTESTA |  |
| **29.1- Te sentiste relajado (flojo, distendido)?** | | 1 | 2 | 3 | 4 | 5 | 98 | 99 |
| **29.2- Te sentiste feliz?** | | 1 | 2 | 3 | 4 | 5 | 98 | 99 |
| **29.3- Te volviste más agresivo hacia otras personas?** | | 1 | 2 | 3 | 4 | 5 | 98 | 99 |
| **29.4- Te sentiste más amable y abierto?** | | 1 | 2 | 3 | 4 | 5 | 98 | 99 |
| **29.5- Te resultó más fácil hablar acerca de tus sentimientos o problemas?** | | 1 | 2 | 3 | 4 | 5 | 98 | 99 |
| **29.6- Te olvidaste de tus problemas?** | | 1 | 2 | 3 | 4 | 5 | 98 | 99 |
| **29.7- Hiciste algo de lo que te arrepentiste?** | | 1 | 2 | 3 | 4 | 5 | 98 | 99 |
| **29.8- La actividad sexual te resultó más placentera?** | | 1 | 2 | 3 | 4 | 5 | 98 | 99 |
| **29.9- Te sentiste más atractivo sexualmente?** | | 1 | 2 | 3 | 4 | 5 | 98 | 99 |
| **29.10- Te metiste en problemas con la policía?** | | 1 | 2 | 3 | 4 | 5 | 98 | 99 |
| **29.11- Te divertiste mucho?** | | 1 | 2 | 3 | 4 | 5 | 98 | 99 |
| **29.12- Te sentiste enfermo?** | | 1 | 2 | 3 | 4 | 5 | 98 | 99 |
| **29.13- No recuerdas lo que pasó?** | | 1 | 2 | 3 | 4 | 5 | 98 | 99 |

***Pregunta 30 es para bebedores pasados (tomaron alcohol en el pasado pero no en los últimos 12 meses). LOS QUE NUNCA TOMARON ALCOHOL PASAN A LA PREGUNTA 31.***

| 1. **La gente tiene diferentes razones para beber alcohol. ¿Qué tan importante dirías que fueron cada una de las siguientes razones para vos? (*MOSTRAR TARJETA*)** | | | | | | |  |
| --- | --- | --- | --- | --- | --- | --- | --- |
|  | Muy importante | Importante | No muy importante | Nada importante | NO SABE | NO CONTESTA | |
| **30.1- Ser sociable y educado?** | 1 | 2 | 3 | 4 | 98 | 99 | |
| **30.2- Debido a que los demás estában bebiendo?** | 1 | 2 | 3 | 4 | 98 | 99 | |
| **30.3- Para agregar al disfrute de las comidas?** | 1 | 2 | 3 | 4 | 98 | 99 | |
| **30.4- Por razones de salud?** | 1 | 2 | 3 | 4 | 98 | 99 | |
| **30.5- Para sentirte bien?** | 1 | 2 | 3 | 4 | 98 | 99 | |
| **30.6- Para ayudarte a relajarte?** | 1 | 2 | 3 | 4 | 98 | 99 | |
| **30.7- Para olvidar las preocupaciones?** | 1 | 2 | 3 | 4 | 98 | 99 | |
| **30.8- Para ayudarte a sentirte menos inhibido o tímido?** | 1 | 2 | 3 | 4 | 98 | 99 | |
| **30.9- Para celebrar?** | 1 | 2 | 3 | 4 | 98 | 99 | |
| **30.10- Debido a su sabor?** | 1 | 2 | 3 | 4 | 98 | 99 | |
| **30.11- Para sacarte la sed?** | 1 | 2 | 3 | 4 | 98 | 99 | |

***Pregunta 31 es para bebedores pasados y los que nunca bebieron alcohol.***

| 1. **Las personas también tienen diferentes razones por las que limitan su consumo de alcohol, o no beben en absoluto. ¿Qué tan importante dirías que son cada una de las siguientes razones para vos? *(MOSTRAR TARJETA)*** | | | | | | |  |
| --- | --- | --- | --- | --- | --- | --- | --- |
|  | Muy importante | Importante | No muy importante | Nada importante | NO SABE | NO CONTESTA | |
| **31.1- (*SOLO SI ES MUJER*) Porque usted estaba o está embarazada, o tratando de quedar embarazada?** | 1 | 2 | 3 | 4 | 98 | 99 | |
| **31.2- Debido al sabor?** | 1 | 2 | 3 | 4 | 98 | 99 | |
| **31.3- Porque no te gusta el efecto que tiene en vos?** | 1 | 2 | 3 | 4 | 98 | 99 | |
| **31.4- Porque has visto malos ejemplos de lo que el alcohol puede hacer?** | 1 | 2 | 3 | 4 | 98 | 99 | |
| **31.5- Debido a que has sido perjudicado por la bebida de otra persona?** | 1 | 2 | 3 | 4 | 98 | 99 | |
| **31.6- Debido a que la bebida puede afectar tu rendimiento laboral o en los estudios?** | 1 | 2 | 3 | 4 | 98 | 99 | |
| **31.7- Debido a que la bebida es demasiado cara o es una pérdida de dinero?** | 1 | 2 | 3 | 4 | 98 | 99 | |
| **31.8- Debido a razones religiosas?** | 1 | 2 | 3 | 4 | 98 | 99 | |
| **31.9- Porque te educaron para no beber?** | 1 | 2 | 3 | 4 | 98 | 99 | |
| **31.10- Debido a que has tenido problemas con el alcohol o tienes miedo de convertirte en un alcohólico?** | 1 | 2 | 3 | 4 | 98 | 99 | |
| **31.11- Debido a que sos demasiado joven?** | 1 | 2 | 3 | 4 | 98 | 99 | |
| **31.12- Debido a que tus amigos y / o familiares desaprueban las bebidas alcohólicas?** | 1 | 2 | 3 | 4 | 98 | 99 | |
| **31.13- Por razones de salud?** | 1 | 2 | 3 | 4 | 98 | 99 | |
| **31.14- Debido a que no estás interesado?** | 1 | 2 | 3 | 4 | 98 | 99 | |

# MÓDULO 5: Percepciones sobre el alcohol y otras sustancias

| 1. **Por favor, dime si estás de totalmente de acuerdo, de acuerdo, ni de acuerdo ni en desacuerdo, en desacuerdo o totalmente en desacuerdo con cada una de las siguientes afirmaciones. (*MOSTRAR TARJETA*)** | | | | | | | |
| --- | --- | --- | --- | --- | --- | --- | --- |
|  | Totalmente de acuerdo | De acuerdo | Ni de acuerdo ni en desacuerdo | En desacuerdo | Totalmente en desacuerdo | NO SABE | NO CONTESTA |
| **32.1- Tomar una bebida es uno de los placeres de la vida.** | 1 | 2 | 3 | 4 | 5 | 98 | 99 |
| **32.2- Tomar una copa con alguien es una forma de ser amable.** | 1 | 2 | 3 | 4 | 5 | 98 | 99 |
| **32.3- No hay nada bueno que decir acerca de la bebida.** | 1 | 2 | 3 | 4 | 5 | 98 | 99 |

| 1. **A continuación voy a describir situaciones en las que a veces se encuentran las personas. Por favor dime ¿en qué medida una persona en estas situaciones debe sentirse libre para tomar bebidas alcohólicas? (*MOSTRAR TARJETA*)** | | | | | | | | |
| --- | --- | --- | --- | --- | --- | --- | --- | --- |
|  | 0 bebidas | Beber un poco pero no lo suficiente para sentir los efectos (1 ó 2 tragos) | Lo suficiente para sentir los efectos pero no quedar borracho | Emborracharse está bien a veces | Emborracharse está bien siempre | NO SABE | NO CONTESTA |  |
| **33.1- Como madre, pasando tiempo con los niños pequeños** | 1 | 2 | 3 | 4 | 5 | 98 | 99 |  |
| **33.2- Como padre, pasando tiempo con los niños pequeños** | 1 | 2 | 3 | 4 | 5 | 98 | 99 |  |
| **33.3- Un hombre en un bar con los/as amigos/as** | 1 | 2 | 3 | 4 | 5 | 98 | 99 |  |
| **33.4- Una mujer en un bar con las/os amigas/os** | 1 | 2 | 3 | 4 | 5 | 98 | 99 |  |
| **33.5- Un hombre con los/as compañeros/as de trabajo** | 1 | 2 | 3 | 4 | 5 | 98 | 99 |  |
| **33.6- Una mujer con las/os compañeras/os de trabajo** | 1 | 2 | 3 | 4 | 5 | 98 | 99 |  |
| **33.7- Un hombre cenando en casa con su cónyuge o pareja** | 1 | 2 | 3 | 4 | 5 | 98 | 99 |  |
| **33.8- Una mujer cenando en casa con su cónyuge o pareja** | 1 | 2 | 3 | 4 | 5 | 98 | 99 |  |

| 1. **Durante los últimos 12 meses, ¿con qué frecuencia consumiste alguna de estas sustancias? *(MOSTRAR TARJETA)*** | | | | | | | | | | | | | | | | | | | | | | | | | | | | |
| --- | --- | --- | --- | --- | --- | --- | --- | --- | --- | --- | --- | --- | --- | --- | --- | --- | --- | --- | --- | --- | --- | --- | --- | --- | --- | --- | --- | --- |
|  | | Todos los días | | 5 a 6 veces a la semana | | 3 a 4 veces a la semana | | 1 a 2 veces a la semana | | 2 a 3 veces al mes | | Una vez al mes | | 6 a 11 veces en los últimos 12 meses | | 2 a 5 veces en los últimos 12 meses | | Una vez en los últimos 12 meses | | No consumió en el último año, pero sí en el pasado | | Nunca consumió | | NO SABE | | NO CONTESTA | |  |
| **34.1- Tabaco** | | 1 | | 2 | | 3 | | 4 | | 5 | | 6 | | 7 | | 8 | | 9 | | 10 | | 11 | | 98 | | 99 | |  |
| **34.2- Uso indebido de medicamentos. Uso sin indicación médica de tranquilizantes, hipnóticos (medicamentos para dormir), antidepresivos o estimulantes.** | | 1 | | 2 | | 3 | | 4 | | 5 | | 6 | | 7 | | 8 | | 9 | | 10 | | 11 | | 98 | | 99 | |  |
| **34.3- Marihuana** | | 1 | | 2 | | 3 | | 4 | | 5 | | 6 | | 7 | | 8 | | 9 | | 10 | | 11 | | 98 | | 99 | |  |
| **34.4- Cocaína** | | 1 | | 2 | | 3 | | 4 | | 5 | | 6 | | 7 | | 8 | | 9 | | 10 | | 11 | | 98 | | 99 | |  |
| **34.5- Pasta Base** | | 1 | | 2 | | 3 | | 4 | | 5 | | 6 | | 7 | | 8 | | 9 | | 10 | | 11 | | 98 | | 99 | |  |
| **34.6- Otras drogas (hashis, alucinógenos, inhalantes, extasis, anfetaminas, ketamina, crack, heroína, morfina, metanfetaminas, opio). *ESPECIFICAR:*** | | 1 | | 2 | | 3 | | 4 | | 5 | | 6 | | 7 | | 8 | | 9 | | 10 | | 11 | | 98 | | 99 | |  |

# MÓDULO 6: Adultez emergente.

| 1. **Por favor, dime si estás totalmente de acuerdo, de acuerdo, ni de acuerdo ni en desacuerdo, en desacuerdo o totalmente en desacuerdo con cada una de las siguientes afirmaciones. (*MOSTRAR TARJETA*)** | | | | | | | |
| --- | --- | --- | --- | --- | --- | --- | --- |
|  | Totalmente de acuerdo | De acuerdo | Ni de acuerdo ni en desacuerdo | En desacuerdo | Totalmente en desacuerdo | NO SABE | NO CONTESTA |
| **35.1- Has llegado a la edad adulta.** | 1 | 2 | 3 | 4 | 5 | 98 | 99 |
| **35.2- Sos financieramente independiente de tus padres u otros miembros de la familia.** | 1 | 2 | 3 | 4 | 5 | 98 | 99 |
| **35.3- Sos emocionalmente independiente de tus padres o tutores.** | 1 | 2 | 3 | 4 | 5 | 98 | 99 |

| 1. **Para cada frase que te voy a leer sobre la etapa de tu vida actual, por favor dime en que grado estás de acuerdo. *(MOSTRAR TARJETA)*** | | | | | | | | |
| --- | --- | --- | --- | --- | --- | --- | --- | --- |
| **Este período de tu vida es un ...** | | Totalmente de acuerdo | De acuerdo | Ni de acuerdo ni en desacuerdo | En desacuerdo | Totalmente en desacuerdo | NO SABE | NO CONTESTA |
| **36.1- Tiempo de descubrir quién eres?** | | 1 | 2 | 3 | 4 | 5 | 98 | 99 |
| **36.2- Tiempo de separarte de tus padres?** | | 1 | 2 | 3 | 4 | 5 | 98 | 99 |
| **36.3- Tiempo de decidir de acuerdo a tus propias creencias y valores?** | | 1 | 2 | 3 | 4 | 5 | 98 | 99 |
| **36.4- Tiempo de planificar el futuro?** | | 1 | 2 | 3 | 4 | 5 | 98 | 99 |
| **36.5- Tiempo de muchas posibilidades?** | | 1 | 2 | 3 | 4 | 5 | 98 | 99 |
| **36.6- Tiempo de probar cosas nuevas?** | | 1 | 2 | 3 | 4 | 5 | 98 | 99 |
| **36.7- Tiempo de mucho stress?** | | 1 | 2 | 3 | 4 | 5 | 98 | 99 |
| **36.8- Tiempo de imprevisibilidad?** | | 1 | 2 | 3 | 4 | 5 | 98 | 99 |
| **36.9- Tiempo de muchas preocupaciones?** | | 1 | 2 | 3 | 4 | 5 | 98 | 99 |
| **36.10- Tiempo de sentar cabeza?** | | 1 | 2 | 3 | 4 | 5 | 98 | 99 |
| **36.11- Tiempo de ser responsable por tus seres queridos?** | | 1 | 2 | 3 | 4 | 5 | 98 | 99 |
| **36.12- Tiempo de libertad personal?** | | 1 | 2 | 3 | 4 | 5 | 98 | 99 |
| **36.13- Tiempo de optimismo?** | | 1 | 2 | 3 | 4 | 5 | 98 | 99 |
| **36.14- Tiempo de ser autosuficiente?** | | 1 | 2 | 3 | 4 | 5 | 98 | 99 |
| **36.15- Tiempo de centrarte en ti mismo?** | | 1 | 2 | 3 | 4 | 5 | 98 | 99 |
| **36.16- Tiempo de sentirte adulto en algunos aspectos pero no en otros?** | | 1 | 2 | 3 | 4 | 5 | 98 | 99 |

#

# MÓDULO 7: Demografía

| 1. **Sin incluirse usted , ¿cuántos adultos de 18 años o más viven en este hogar** | | | | | |
| --- | --- | --- | --- | --- | --- |
| ADULTOS ***( SI ES 0 , PASAR A PREGUNTA 39* )** | | | |  | |
| 98 - NO SABE | | | | | |
| 99 - NO CONTESTA | | | | | |
| 1. **¿Quiénes son? (*SELECCIONE TODAS LAS QUE APLIQUEN*)** | | | | | |
| 1 - cónyuge o pareja | | | | | |
| 2 - padres o tutores **(*PASAR A PREGUNTA 40*)** | | | | | |
| 3 - demás miembros de la familia | | | | | |
| 4 - otras personas que no son familiares. | | | | | |
| 1. **¿En qué año dejaste la casa de tus padres (o tutores) y comenzaste a vivir de forma independiente? (independencia de la casa)** | | | | | |
| AÑO: | | | |  | |
| 97 - Nunca dejó la casa de los padres. | | | | | |
| 98 - NO SABE | | | | | |
| 99 - NO CONTESTA | | | | | |
| 1. **¿Tienes hijos?** | | | | | |
| 1 – SÍ | | | | | |
| 2 - NO **(*PASAR A PREGUNTA 44*)** | | | | | |
| 98 - NO SABE **(*PASAR A PREGUNTA 44*)** | | | | | |
| 99 - NO CONTESTA **(*PASAR A PREGUNTA 44*)** | | | | | |
| 1. **¿Cuántos hijos tienes?** | | | | | |
| NÚMERO DE HIJOS: | | | |  | |
| 98 – NO SABE ***(PASAR A PREGUNTA 44)*** | | | | | |
| 99 – NO CONTESTA ***(PASAR A PREGUNTA 44)*** | | | | | |
| **Hijo**  **#** | 1. **¿Cuáles son sus edades? (*EMPEZANDO POR EL MÁS JOVEN*)** | | 1. **¿Él o ella vive contigo?** | | |
| 1 | EDAD: |  | 1-Si | | 2- No |
|  | 98 – NO SABE | | 98 – NO SABE | | |
|  | 99 – NO CONTESTA | | 99 – NO CONTESTA | | |
| 2 | EDAD: |  | 1-Si | | 2- No |
|  | 98 – NO SABE | | 98 – NO SABE | | |
|  | 99 – NO CONTESTA | | 99 – NO CONTESTA | | |
| 3 | EDAD: |  | 1-Si | | 2- No |
|  | 98 – NO SABE | | 98 – NO SABE | | |
|  | 99 – NO CONTESTA | | 99 – NO CONTESTA | | |
| 4 | EDAD: |  | 1-Si | | 2- No |
|  | 98 – NO SABE | | 98 – NO SABE | | |
|  | 99 – NO CONTESTA | | 99 – NO CONTESTA | | |
| 5 | EDAD: |  | 1-Si | | 2- No |
|  | 98 – NO SABE | | 98 – NO SABE | | |
|  | 99 – NO CONTESTA | | 99 – NO CONTESTA | | |
| 6 | EDAD: |  | 1-Si | | 2- No |
|  | 98 – NO SABE | | 98 – NO SABE | | |
|  | 99 – NO CONTESTA | | 99 – NO CONTESTA | | |
| 7 | EDAD: |  | 1-Si | | 2- No |
|  | 98 – NO SABE | | 98 – NO SABE | | |
|  | 99 – NO CONTESTA | | 99 – NO CONTESTA | | |
| 8 | EDAD: |  | 1-Si | | 2- No |
|  | 98 – NO SABE | | 98 – NO SABE | | |
|  | 99 – NO CONTESTA | | 99 – NO CONTESTA | | |

| 1. **¿Cuál es el nivel educativo más alto que alcanzaste?** |
| --- |
| 1- No tiene estudios, primaria completa o incompleta |
| 2- Secundaria incompleta o completa |
| 3- Enseñanza técnica (UTU o similar) completa o incompleta |
| 4- Estudios terciarios incompletos (incluye magisterio, profesorado, carreras terciarias no universitarias y carreras de grado universitarias, sin tener el título habilitante de ninguna de ellas) |
| 5- Estudios terciarios de grado completos (incluye magisterio, profesorado, carreras terciarias no universitarias o carreras universitarias de grado y tener el título habilitante en al menos alguna de ellas) |
| 6- Posgrado (requiere tener estudios terciarios completos) |
| 98 - NO SABE |
| 99 - NO CONTESTA |

| 1. **¿Te encuentras estudiando actualmente?** | |
| --- | --- |
| 1 - SI | |
| 2 - NO **(*PASAR A PREGUNTA 47*)** | |
| 98 - NO SABE | |
| 99 - NO CONTESTA | |
| 1. **¿Qué tiempo dedicas a estudiar?** | |
| 1 - Estudiante de tiempo completo **(*PASAR A PREGUNTA 48*)** | |
| 2 - Estudiante a tiempo parcial **(*PASAR A PREGUNTA 48*)** | |
| 98 - NO SABE **(*PASAR A PREGUNTA 48*)** | |
| 99 - NO CONTESTA **(*PASAR A PREGUNTA 48*)** | |
| 1. **¿En qué año te graduaste o dejaste de estudiar?** | |
| AÑO: |  |
| 98 – NO SABE | |
| 99 – NO CONTESTA | |
| 1. **¿Cuál es tu situación laboral actualmente? *(LEER OPCIONES)*** | |
| 1- Trabaja a tiempo completo (40 horas o más por semana) (incluir trabajo por cuenta propia) ***(PASAR A PREGUNTA 51)*** | |
| 2- Trabaja a tiempo parcial (menos de 40 horas por semana) (incluir trabajo por cuenta propia) ***(PASAR A PREGUNTA 51)*** | |
| 3- Desempleado. | |
| 4- Trabajo doméstico no remunerado. | |
| 5- Discapacitado y no puede trabajar ***(PASAR A PREGUNTA 53)*** | |
| 6- Otra situación ***(ESPECIFICAR)***:  ______________________________________ | |
| 98- NO SABE. | |
| 99- NO CONTESTA. | |
| 1. **¿Actualmente estás buscando un trabajo remunerado?** | |
| 1 - SÍ | |
| 2 - NO ***(PASAR A PREGUNTA 52)*** | |
| 98- NO SABE. ***(PASAR A PREGUNTA 52)*** | |
| 99- NO CONTESTA. ***(PASAR A PREGUNTA 52)*** | |
|  | |
| 1. **¿Sería éste tu primer trabajo remunerado?** | |
| 1 - SÍ ***(PASAR A PREGUNTA 54)*** | |
| 2 - NO ***(PASAR A PREGUNTA 52)*** | |
| 98- NO SABE. ***(PASAR A PREGUNTA 52)*** | |
| 99- NO CONTESTA. ***(PASAR A PREGUNTA 52)*** | |
| 1. **¿Este es tu primer trabajo remunerado?** | |
| 1 - SÍ | |
| 2 - NO | |
| 98 – NO SABE | |
| 99 – NO CONTESTA | |
| 1. **¿En qué año comenzaste a trabajar?** | |
| AÑO: |  |
| 97 – Nunca tuvo un trabajo remunerado. | |
| 98 – NO SABE | |
| 99 – NO CONTESTA | |

| 1. **¿Cuál es tu ocupación o profesión? Si no estás trabajando actualmente, pero trabajaste en el pasado, por favor indica cual fue la ocupación en la que estuviste empleado. Si has trabajado en múltiples ocupaciones, por favor selecciona en la que has estado empleado por mayor tiempo. *(MOSTRAR TARJETA)*** | |
| --- | --- |
| 1- Directores y gerentes. | |
| 2- Profesionales, científicos e intelectuales. | |
| 3- Técnicos y profesionales de nivel medio. | |
| 4- Personal de apoyo administrativo. | |
| 5- Trabajadores de los servicios y vendedores de comercios y mercados. | |
| 6- Agricultores y trabajadores calificados agropecuarios, forestales y pesqueros. | |
| 7- Oficiales, operarios y artesanos de artes mecánicas y de otros oficios. | |
| 8- Operadores de instalaciones, maquinas y ensambladores. | |
| 9- Ocupaciones elementales. | |
| 10- Ocupaciones militares. | |
| 97 - Nunca ha sido empleado. | |
| 98 - NO SABE | |
| 99 - NO CONTESTA | |
|  |  |
| 1. **¿Cómo te defines en materia religiosa?** | |
| 1 - Agnóstico / Ateo | |
| 2 - Budista | |
| 3 - Cristiano | |
| 4 - Hindú | |
| 5 - Judío | |
| 6 - Musulmán | |
| 7 - Sikh | |
| 8- Otra religión ***(ESPECIFICAR)***:  ___________________________________________ | |
| 1. **¿Cuál crees que es tu ascendencia étnico racial principal? *(LEER OPCIONES)*** | |
| 1 - Afro o Negra | |
| 2 - Asiática o amarilla | |
| 3 - Blanca | |
| 4 - Indígena | |
| 5 – Otra ***(ESPECIFICAR):***  ___________________________________________ | |
| 98 – NO SABE | |
| 99 – NO CONTESTA | |

# MÓDULO 8: Índice de Nivel Socio Económico. Datos de clasificación.

| 1. **Barrio de Montevideo (según nomenclatura del INE) o Departamento del Interior:______________________ Ptos. ( )** |
| --- |

| 1. **¿Cuántas personas viven habitualmente en este hogar?** | **ESTE HOGAR, ¿CUENTA CON…** | | |
| --- | --- | --- | --- |
| (1) Una persona 0 | 1. **Automóvil (sólo para uso del hogar)** | | |
| (2) Dos o tres personas 3 | (1) No hay 0 | | |
| (3) Cuatro personas 4 | (2) Uno 4 | | |
| (4) Cinco o más personas 6 | (3) Más de uno 7 | | |
| 1. **¿Y cuántos niños menores de 10 años, incluyendo recién nacidos, viven habitualmente en este hogar?** | 1. **TV color** | | |
|  | (1) Ninguno 0 | | |
| (1) Un menor o ninguno 2 | (2) Uno 2 | | |
| (2) Dos menores 1 | (3) Dos 4 | | |
| (3) Más de dos menores 0 | (4) Más de dos 4 | | |
| 1. **¿Cuál es el máximo nivel educativo alcanzado por el principal sostenedor del hogar?** | 1. **Heladera con o sin freezer** | | |
|  | (1) Tiene 4 | | |
| (1) Primaria completa o menos 0 | (2) No tiene 0 | | |
| (2) Enseñanza media completa o incompleta 1 | 1. **Computadora (No incluye CEIBAL)** | | |
| (3) Enseñanza técnica (UTU o similar) completa o incompleta 1 | (1) Una 2 | | |
| (4) Terciaria incompleta (Mag/Profesorado) 1 | (2) Dos 3 | | |
| (5) Universitario Incompleto 1 | (3) Más de dos 4 | | |
| (6) Terciaria completa (Mag/Profesorado) 3 | (4) No tiene 0 | | |
| (7) Universitario Completo 3 | 1. **Teléfono fijo** | | |
| (8) Post Grado 6 | (1) Tiene 3 | | |
| 1. **¿Cuántos miembros del hogar tienen estudios universitarios (completos o incompletos)?** | (2) No tiene 0 | | |
|  | 1. **TV para abonados** | | |
| (1) No hay 0 | (1) Tiene 2 | | |
| (2) Una persona o más 3 | (2) No tiene 0 | | |
| 1. **¿En cuál de las siguientes instituciones de asistencia a la salud se atiende el principal sostenedor del hogar?** | 1. **Aire Acondicionado** | | |
|  | (1) Tiene 2 | | |
| (1) Hospital Público 0 | (2) No tiene 0 | | |
| (2) Hospital Policial o Militar 5 | 1. **Microondas** | | |
| (3) Mutualista 7 | (1) Tiene 2 | | |
| (4) Seguro Privado 8 | (2) No tiene 0 | | |
| 1. **¿Cuántas personas perciben ingresos en el hogar?** | 1. **Lavarropas** | | |
| (1) Una persona 0 | (1) Tiene 2 | | |
| (2) Dos personas 5 | (2) No tiene 0 | | |
| (3) Tres personas 9 | 1. **Lavavajilla** | | |
| (4) Más de tres personas 11 | (1) Tiene 2 | | |
| 1. **El material predominante del techo es:** | (2) No tiene 0 | | |
| (1) De chapa u otro material precario 0 | 1. **U. DVD** | | |
| (2) De material (planchada u otro tipo) 3 | (1) Tiene 1 | | |
| 1. **¿Cuántos baños tiene la vivienda?** | (2) No tiene 0 | | |
| (1) Uno o ninguno 0 | ***PUNTOS DE CORTE INSE:*** | | ***PUNTAJE TOTAL:*** |
| (2) Dos 3 | *(1) B-* | *De 0 a 23* |  |
| (3) Más de dos 7 | *(2) B+* | *De 24 a 32* |  |
| 1. **¿El hogar cuenta con servicio doméstico?** | *(3) M-* | *De 33 a 39* |  |
| (1) No tiene 0 | *(4) M* | *De 40 a 46* |  |
| (2) Por hora 4 | *(5) M+* | *De 47 a 54* |  |
| (3) Por día 7 | *(6) A-* | *De 55 a 65* |  |
| (4) Con cama 11 | *(7) A+* | *De 66 a 100* |  |

*…STOP* **Registre la hora actual** _____ : _____  **(HH:MM, 24:00 clock)**

# MÓDULO 9: Reclutamiento y selección.

Estamos llegando al final de esta encuesta. Me gustaría hacerle algunas preguntas más.

*ENG1 ...*

1. **En una escala de 1 a 10, siendo 1 nada interesado y 10 muy interesado, ¿qué tan interesado has estado en esta encuesta?**

“*Nada interesado*” **1 2 3 4 5 6 7 8 9 10** “*Muy interesado*”

98 - NO SABE

99 - NO CONTESTA

*ENG2 ...*

1. **Y, también en una escala de 1 a 10, donde 1 es nada y 10 es mucho, ¿cuánto dirías que te ha gustado participar en esta encuesta?**

“*Nada*” **1 2 3 4 5 6 7 8 9 10** “*Mucho*”

98 - NO SABE

99 - NO CONTESTA

***Como ya he mencionado al principio, es importante para el éxito de este estudio, recoger información de personas con diferentes opiniones, puntos de vista y experiencias, así que, muchas gracias. Esperamos que muchas personas que han respondido esta encuesta deseen participar en la segunda etapa, la cual consiste en responder algunas preguntas. No todo el que se compromete a participar en la segunda etapa será contactado.***

| 1. **¿Te gustaría ser considerado para la segunda parte del estudio?** |
| --- |
| 1 – SÍ ***(PASAR A PREGUNTA 81)*** |
| 2 – NO |
| 98 – NO SABE |
| 99 – NO CONTESTA |
| 1. **¿Quieres decirme por qué no deseas participar?** |
| (Respuesta abierta)  __________________________________________ |
| 1. **¿Con qué frecuencia usas Internet?** |
| 1 - Diariamente |
| 2 - Semanalmente |
| 3 - Mensualmente |
| 4 - Menos de una vez al mes |
| 5 - Nunca |
| 6 - No tengo acceso a Internet |
| 98 - NO SABE |
| 99 - NO CONTESTA |
|  |
| 1. **¿Con qué frecuencia visitas tu cuenta de correo electrónico?** |
| 1 - Diariamente |
| 2 - Semanalmente |
| 3 - Mensualmente |
| 4 - Menos de una vez al mes |
| 5 - Nunca |
| 6 - No tengo cuenta de correo electrónico |
| 98 - NO SABE |
| 99 - NO CONTESTA |
| **Si pregunta 81 = 5 ó 6 y pregunta 82 = 5 ó 6 *FINALIZAR ENTREVISTA*.**  **Si pregunta 81 = 1 a 4 y pregunta 82 = 5 o 6 *PASAR A PREGUNTA 84.***  **Si pregunta 81 = 1 a 4 y pregunta 82 = 1 a 4 *CONTINÚE LA ENTREVISTA.*** |
|  |
| 1. **¿Tienes una dirección de correo electrónico donde podamos comunicarnos contigo?** |
| 1 - Sí, mi dirección de correo electrónico:  _____________________________________________  ***(ENCUESTADOR: VERIFIQUE LA DIRECCIÓN DE CORREO ELECTRÓNICO SOLICITANDO AL ENCUESTADO QUE LA REPITA)*** |
| 2 - NO tengo una dirección de correo electrónico |
| 98 - NO SABE |
| 99 - NO CONTESTA |
| 1. **¿Podrías proporcionarnos otro modo de contacto por si no podemos comunicarnos contigo por correo electrónico, como un número de teléfono o una dirección?** |
| 1 - Sí, mi otra información de contacto es:N° TEL:__________________________________ ***(ENCUESTADORR: VERIFIQUE SOLICITANDO REPETIR LA INFORMACIÓN).***  DIRECCIÓN:____________________________________  ***(ENCUESTADORR: VERIFIQUE SOLICITANDO REPETIR LA INFORMACIÓN).*** |
| 2 - NO, no tengo otro modo de contacto. |
| 98 - NO SABE |
| 99 - NO CONTESTA |

**FIN DE LA ENCUESTA.**

**MUCHAS GRACIAS.**

HORA FINALIZACIÓN DE LA ENCUESTA: _____ : _____  **(HH:MM, 24:00 clock)**

| **ENCUESTADOR(A):** ¿Cómo evaluaría la actitud del encuestado hacia la entrevista? |
| --- |
| 1. Muy buena |
| 2. Buena |
| 3. Ni buena, ni mala |
| 4. Mala |
| 5. Muy mala |

**COMENTARIOS: ________________________________________________________________________________________________**

**________________________________________________________________________________________________________________________________________________________________________________________________________________________________________________**

| **SOLO A MODO DE SUPERVISIÓN, NOS PODRÍAS DAR TU NOMBRE Y NÚMERO DE TELEFÓNO:** | |
| --- | --- |
| **NOMBRE ENTREVISTADO/A:** |  |
| **TELÉFONO ENTREVISTADO/A:** |  |

|  | **NOMBRE** | **FECHA** |
| --- | --- | --- |
| **ENCUESTADOR(A)** |  | **/ /** |
| **REPORTE** |  | **/ /** |
| **EDICIÓN** |  | **/ /** |
| **SUPERVISIÓN CALLE** |  | **/ /** |
| **SUPERVISIÓN TELF.** |  | **/ /** |
| **CODIFICACIÓN** |  | **/ /** |
| **DIGITACIÓN** |  | **/ /** |
