# Supplementary material for: Context and culture associated with alcohol use amongst youth in major urban cities: A cross-country population based survey
Source: PLoS One. 2017 Nov 20;12(11):e0187812. doi: 10.1371/journal.pone.0187812 (PMC5695777; doi:10.1371/journal.pone.0187812)
Supplement: S7 Questionnaire — (DOCX) [file pone.0187812.s007.docx]

**LTACC Questionnaire (Yoruba Language)**

Final Draft

**Contents**

[Module A: Household Identification and Census, Consent, and Administrative Variables (ADMN) 3](#_Toc394931724)

[MODULE 1: Demographics](#_Toc394931725) 6

[MODULE 9: Health Behaviors, Including Drinker Status (HTBX)](#_Toc394931726) 10

[MODULE 10: Alcohol Consumption, Last 12 Months (CONS)](#_Toc394931727) 11

[10.1 General Alcohol Consumption 11](#_Toc394931728)

[10.2 Beverage-Specific Quantity/Frequency](#_Toc394931729) 12

[10.3 Experience of Drunkenness/Intoxication](#_Toc394931730) 14

[10.4 Drinking Context](#_Toc394931731) 14

[MODULE 15: Motivations For/Against, and Effects of, Drinking – DRINKERS ONLY (MTCD)](#_Toc394931732) 16

[MODULE 16: Motivations For/Against, and Past Outcomes of, Drinking – NON-DRINKERS ONLY (MTND)](#_Toc394931733) 19

[MODULE 17: Own and Others’ Perceptions of Alcohol and Other Substances (PCPN)](#_Toc394931734) 22

[MODULE 20: Adolescents and Young Adults (ADYA) 24](#_Toc394931735)

[20.3 Emerging Adulthood](#_Toc394931736) 24

[MODULE 21: Respondent Engagement, Recruiting and Screening (RCRT) 25](#_Toc394931737)

# **Module A: Household Identification and Census, Consent, and Administrative Variables (ADMN)**

***A.0 Household Identification, Date*** *(Complete before approaching the household/respondent)*

LEV1… City/metropolitan area:

__ __ (see codebook)

LEV2… [LGA ]:

__ __ (see codebook)

LEV3… [Wards ]:

__ __ (see codebook)

LEV4… [Neighbourhood/settlement ]:

__ __ (see codebook)

…LEV5 [Street ]:

__ __ (see codebook)

…LEV6 [Household ]:

__ __ (see codebook)

…INTID __ __ __ __

…DATE __ __ (dd) __ __ (mm) __ __ __ __ (yyyy)

***A.1 Household and Respondent Disposition*** *(Complete after contact with the household/respondent)*

…HDIS Household disposition:

__ __ (see codebook)

…RDIS: Respondent disposition:

__ __ (see codebook)

…RDIS_TXT Reason for early termination of interview, if applicable (i.e., ADMN.RDIS = 25 or 26)

________________________________________________________________________

________________________________________________________________________

***A.2 Household Introduction, Census, Determination of Respondent Eligibility***

...INTR E karo Sir/Ma, Oruko mi ni [name]. Mo je eniti nsise ibanisoro ti on si nsise pelu [DR A.B Makanjuola] ati International Center for Alcohol Policies, in Washington, DC, USA. Adiresi yin je okan ninu awon ile ti ati fe baa won eyan soro nipa ise iwadi ati imo injinle ti an se nipa oti mimu lerin awon odo ni ilu Ilorin. N je mo le bere awon ibere kan lati mo boya enikeni ti o ngbe inu ile yin le kopa ninu iwadi yi?

*After obtaining consent:*

E se pupo. Nje e le so fun mi nipa ojo ori enikokan ti o ngbe inu ile yi?

*Complete Columns 1 and 2 of the matrix below by entering ages and circling the correct gender. For every person listed who is between 18 and 34 years of age, ask whether s/he has lived in [country-specific city/metropolitan area] for at least 6 months and complete Column 3 as appropriate.*

Household Census

|  | **Ojo ori yin** | **Okunrin tabi obirin** | **Nje e ti gbe Ilorin tabi adugbo yi fun osu mefa tabi ju be lo?** |  |  | **Ojo ori yin** | **Okunrin tabi obirin** | **Nje e ti gbe Ilorin tabi adugbo yi fun osu mefa tabi ju be lo?** |
| --- | --- | --- | --- | --- | --- | --- | --- | --- |
| **Eni kini** |  | Okunrin/obirin | Beni/Beko |  | **Eni keje** |  | Okunrin/obirin | Beni/Beko |
| **Eni keji** |  | Okunrin/obirin | Beni/Beko |  | **Eni kejo** |  | Okunrin/obirin | Beni/Beko |
| **Eni keta** |  | Okunrin/obirin | Beni/Beko |  | **Eni kesan** |  | Okunrin/obirin | Beni/Beko |
| **Eni kerin** |  | Okunrin/obirin | Beni/Beko |  | **Eni kewa** |  | Okunrin/obirin | Beni/Beko |
| **Eni karun** |  | Okunrin/obirin | Beni/Beko |  | **Eni kokanla** |  | Okunrin/obirin | Beni/Beko |
| **Eni kefa** |  | Okunrin/obirin | Beni/Beko |  | **Eni kejila** |  | Okunrin/obirin | Beni/Beko |

*If no eligible respondent, move to next household. If multiple eligible respondents, select respondent using last birthday method and ask to speak to selected respondent. Skip to A.1 and complete the questions below only after* interviewing (or attempting to interview) the selected respondent.

…LANG2 *Language in which interview was conducted:*

1. Yoruba
2. Hausa
3. Ibo
4. English

…LANG3 *Rate the respondent’s apparent comfort with the interview language, on a scale of 1 to 5 (1 = respondent unable to understand, could not finish interview; 5 = respondent fluent, appeared to understand every question with ease): __*

***A.3 Respondent Introduction and Consent***

...INST1 E karo Sir/Ma, Oruko mi ni [name]. Mo je eniti nsise ibanisoro ti on si nsise pelu [DR A.B Makanjuola] ati International Center for Alcohol Policies, in Washington, DC, USA. Adiresi yin je okan ninu awon ile ti ati fe baa won eyan soro nipa ise iwadi ati imo injinle ti an se nipa oti mimu lerin awon odo ni ilu Ilorin. N je mo le bere awon ibere kan lati mo boya enikeni ti o ngbe inu ile yin le kopa ninu iwadi yi? Fun aseyori ise yi a nfe oniruru idahun lati odo yin ati awon elomiran. A o bere nipa ogun tabi alafia yin, ise si yin ati awon ero yin nipa oti mimu. Ibere yi yio to bi iseju ogun tabi ogbon. E ni ase lati kopa tabi lai kopa ninu iwadi yi. Gbogbo ohun ti e ba so ni yio je asiri. Ti ibere kan o ba te yin lorun, dandan ko ni ki e dahun. E le da nu duro ni igbakigba ninu iwadi yi. Nje e fe kopa ninu iwadi yi?

________

Initials of interviewer, indicating

verbal consent of respondent

***A.4 Screening and Administrative Questions***

...INST2 E se pupo. Ki a to bere, ma fe bere nipa awon nkakan lati ni idaniloju wipe e le kopa ninu iwadi yi.

...BYR Odun wo ni won bi yi yin?

__ __ __ __

98 – Ko moo

99 – Ko dahun

*Probe if, based on birth year, respondent may be younger than 18 (born in 1996) or older than 34 (born in 1980). Interview ends if respondent is not in desired age range, or age cannot be determined. [Code respondent as “Ineligible”.]*

…RESI N je e ti ngbe ni ilu Ilorin fun osu mefa?

1 – Beni

2 – Beko

98 – Ko moo

99 – Ko dahun

*Interview ends if respondent has not resided in city/metropolitan area for at least the previous 6 months. [Code respondent as “Ineligible”.]*

…LANG1 Kini ede abinibi yin?

1. Yoruba
2. Hausa
3. Ibo
4. Ede geesi
5. Ko moo
6. – Ko dahun

*Interview is conducted in respondent’s primary language, if possible. If this is not possible, or the respondent’s primary language is not known, rate (on previous page) the respondent’s level of comfort with the language in which the interview was conducted upon the conclusion of the interview.*

***A.5 Introduction to Interview***

…INST3 Ti e ko ba lodi si, a o bere sib ere oro lowo yin. E ranti wipe gbogbo idahun yin ni yio je asiri. Nitorina, mo fe ki e dahun ibere wa nitoto. Ti ibere Kankan ko ba te yin lorun, tabi e ko mo idahun re, e so fun mi ki a le lo si ibere miran.

…STRT  *Agogo melo ni o lu:*

__ __ : __ __ (HH:MM, 24:00 clock)

# MODULE 1: Demographics

[1]…SEX *(Koo kale boya okunrin tabi obirin ni. Bere ti ko ba dao loju.)*

1 – Okunrin

2 – Obirin

98 – Ko moo

99 – Ko dahun

[2]...MAR E wo ninu awon nkan wonyi ni o to si yin?

1 – Iyawo wa ni ile mi tabi mo wa ni ile oko

2 – Mo ti se igbeyawo ri sugbon a ko gbe pelu ara wa mo

3 – oko tabi iyawo mi ti se alaisi

4 –Mi o ti se igbeyawo ri (Skip to …HH.AD) Q4

98 – Ko moo (Skip to …HH.AD) Q4

99 – Ko dahun (Skip to …HH.AD) Q4

[3]…MAR_FU Inu odun wo ni ipo ti e dahun yi sele (married/divorced or separated/widowed)?

__ __ __ __

98 – KO moo

99 –Ko dahun

[4]...HH.AD Awon agbalagba (18 years or older) melo ni ngbe ni inu ile odo yin?

__ __ ADULTS *(If 0, skip to DEM.HH.AD_FU2)* Q6

98 – Ko moo

99 – Ko dahun

[5]…HH.AD_FU1 Bawo ni won se je si iyin? (Select all that apply)

1 – oko, iyawo tabi ololufe

2 – obi tabi eni ti o n tomi sona (*Skip to DEM.HH.PNT1) Q7*

3 – ebi

4 – ore, alabagbe tabi ara

98 – Ko moo

99 – Ko dahun

[6]…HH.AD_FU2 Odun wo ni e bere si ndagbe?

__ __ __ __

98 – Ko moo

99 – Ko dahun

[7]...PNT1 Nje e ni awon omo?

1 – Beni

2 – Beko *(Skip to DEM.EDU) Q9*

98 – Ko moo *(Skip to DEM.EDU) Q9*

99 – Ko dahun *(Skip to DEM.EDU) Q9*

[8]…PNT1_FU Melo ni awon omo yin?

______ Children

98 – Ko moo *(Skip to DEM.EDU) Q9*

99 – Ko dahun *(Skip to DEM.EDU) Q9*

*…PNT2 …PNT2_FU*

| Omo # | Kini ojo ori won? (bere pelu e ni ojo ori re kere ju) | Nje o ngbe pelu re? |
| --- | --- | --- |
| 1 | PNT2_1  YEARS  98 – Ko moo  99 – Kodahun | PNT2_1_FU  1 – Beni 2 –Beko  98 – Ko moo  99 – Ko dahun |
| 2 | PNT2_2  YEARS  98 – KO moo  99 –Ko dahun | PNT2_2_FU  1 – BENI 2 – BEKO  98 – KO moo  99 –Ko dahun |
| 3 | PNT2_3  YEARS  98 – KO moo  99 –Ko dahun | PNT2_3_FU  1 – BENI 2 – BEKO  98 – KO moo  99 –Ko dahun |
| 4 | PNT2_4  YEARS  98 – KO moo  99 –Ko dahun | PNT2_4_FU  1 – BENI 2 – BEKO  98 – KO moo  99 –Ko dahun |
| 5 | PNT2_5  YEARS  98 – KO moo  99 –Ko dahun | PNT2_5_FU  1 – BENI 2 – BEKO  98 – KO moo  99 –Ko dahun |
| 6 | PNT2_6  YEARS  98 – KO moo  99 –Ko dahun | PNT2_6_FU  1 – BENI 2 – BEKO  98 – KO moo  99 –Ko dahun |
| 7 | PNT2_7  YEARS  98 – KO moo  99 –Ko dahun | PNT2_7_FU  1 – BENI 2 – BEKO  98 – KO moo  99 –Ko dahun |
| 8 | PNT2_8  YEARS  98 – KO moo  99 –Ko dahun | PNT2_8_FU  1 – BENI 2 – BEKO  98 – KO moo  99 –Ko dahun |

[9]...EDU Ile iwe ti o ga julo wo ni e lo ti e si pari eko?

1. Mi o lo ile iwe rara, mi o pari iwe mefa, mo pari iwe mefa
2. Mi o pari iwe mewa, mo pari iwe mewa (6 – 12 years)
3. Mi o pari fasiti, tabi mo pari OND, College of Education i.e NCE, and Nursing (12-15yrs)
4. Mo pari fasit tabi ile iwe poli (e.g., e.g university graduate, polytechnic graduate (HND), post-graduate degree (16+ of education)
5. 98 – Ko moo
6. 99 – Ko dahun

[10]…STDT Nje e n lo ile iwe nisinsinyi?

1 – Beni *(Skip to DEM.EMPL) Q12*

2 – Beko

98 – Ko moo

99 – Ko dahun

[11]…STDT_GR Odun wo ni e pari ile iwe lilo?

__ __ __ __

98 – Ko moo

99 – Ko dahun

[12]...EMPL Iru ise wo ni e nse?

1 –Ise osu odindi tabi aladani (EMPLOYED FULL-TIME (ogoji wakati tabi jubeelo lose) *(Skip to EMPL_FU3) Q15*

2 –Ise abo (EMPLOYED PART-TIME tabi aladani (okere ju ogoji wakati lose) *(Skip to EMPL_FU3) Q15*

3 –Mi o ti ri ise (UNEMPLOYED)

4 – Olutoju ile (HOMEMAKER)

5 – Mo ni abo ara ati ailera ti ko je kin le sise (DISABLED AND UNABLE TO WORK *(Skip to DEM.OCC) Q17*

6 – Awon idi miran (OTHER): _______________________________________________ *(Skip to EMPL_FU1) Q13*

98 – KO moo

99 –Ko dahun

[13]...EMPL_FU1 Nje e n wa ise nisinsinyi?

1 – Beni

2 – Beko *(Skip to EMPL_FU4.YR) Q16*

98 – Ko moo *(Skip to EMPL_FU4.YR) Q16*

99 – Ko dahun *(Skip to EMPL_FU4.YR) Q16*

[14]...EMPL_FU2 Nje ise yi ni yio je akoko ti won yio san owo fun yin?

1 – Beni *(Skip to DEM.OCC) Q17*

2 – Beko *(Skip to EMPL_FU4) Q16*

98 – Ko moo *(Skip to EMPL_FU4.YR) Q16*

99 – Ko dahun *(Skip to EMPL_FU4.YR) Q16*

[15]...EMPL_FU3 Se Ise akoko ti won yio san owo fun yin niiyi?

1 – Beni

2 – Beko

98 –Ko moo

99 – Ko dahun

[16]…EMPL_FU4 Odun wo ni e koko bere ise ti won nsanwo fun?

__ __ __ __

97 – Mi o ti sise ti won nsanwo funmi ri

98 – Ko moo

99 – Ko dahun

[17]…OCC Iru ise wo ni e ko? Ti e ko ba sise nisinyi, sugbon e ti sise tele ri, iru ise wo ni e se nigbana. Ti e ba se ise ti o po lorisirisi, iru ise wo ni e se fun igba ti o po ju.

| **1** | Ise ologun |
| --- | --- |
| **2** | Ose lu, oludari ile ise |
| **3** | Omowe gegebi dokita, agbejoro, imo nipa owo |
| **4** | A moju ero, |
| **5** | Akowe |
| **6** | Omo ile ise tabi itaja |
| **7** | Agbe nkan ogbin tabi nkan osin |
| **8** | Onise owo |
| **9** | Ise lilo tabi tito ero |
| **10** | Ise pepepee |
| **97** | Won koi ti gba mi si ise ri |
| **98** | Ko moo |
| **99** | Ko dahun |

[18]…REL Kini esin yin?

1 – AGNOSTIC/ATHEIST

2 – BUDDHIST

3 – CHRISTIAN (Esin Kristiani)

4 – HINDU

5 – JEWISH

6 – MUSLIM (Esin Musulumi)

7 – SIKH

8 – ANOTHER RELIGION (e.g Esin Adayeba:_____________________

98 – Ko moo

99 – Ko dahun

[]19…RACE Omo bibi ile bo ni yin?

1. Yoruba
2. Hausa
3. Ibo
4. Ilu miran……………….

98 – KO moo

99 –Ko dahun

# MODULE 9: Health Behaviors, Including Drinker Status (HTBX)

[20]…ALC.EVER Nje e ti mu oti ri?, gege bi [country-specific examples], etc.? Ma se so nipa igba ti e kan fi oti elomiran kan enu die.

1 – BENI

2 – BEKO *(Skip to MTND.MOTV.AGST) Q47*

98 – KO moo

99 –Ko dahun

[21]…ALC.STRT E to omo odun melo ni igba ti e koko mu oti? Ma se so nipa igba ti e kan fi oti elomiran kan enu die.

__ __ YEARS OLD

98 – KO moo

99 –Ko dahun

[22]…ALC.DRUK E to omo odun melo ni igba ti e koko mu otin yo?

__ __ YEARS OLD

97 – Mi o ti mu oti yo ri

98 – KO moo

99 –Ko dahun

[23]…ALC.HVY.STRT A fe mo nipa ojo ori yin ni igbati e n mu oti ti o po julo. Kini iye ojo ori yin ni igbati e bere si mu oti pupo?

__ __ odun (YEARS)

98 – KO moo

99 –Ko dahun

[24]…ALC.HVY.STOP Kini ojo ori yin ni igbati e dekun ati ma mu oti pupo?

__ __ odun (YEARS OLD)

97 – N ko iti dekun re

98 – KO moo

99 –Ko dahun

# MODULE 10: Alcohol Consumption, Last 12 Months (CONS)

## 10.1 General Alcohol Consumption

[25]…GEN.FQ Ni osu mejila seyin (past 12 months), e melo ni e nmu oti beer, wine, spirits (e.g., vodka, gin, whisky, brandy), tabi iru oti Kankan, bi o ti le se kere to? *(Show card)*

1 –Ojojumo

2 – 5 to 6 ni ose

3 – 3 to 4 ni ose

4 – 1 TO 2 ni ose kan

5 – 2 to 3 ni osu

6 – Ekan losu

7 – 6 to 11 ni osu mejila seyin

8 – 2 to 5 ni osu mejila seyin

9 – Ekan ni osu mejila seyin

10 – Mi o mu oti ni osu mejila seyin *(Skip to MTND.EFCT) Q45*

98 – KO moo

99 –Ko dahun

[26]…GEN.QY Ni osu mejila seyin (past 12 months), iwon (units) oti melo ni e ma nmu lojojumo? {By one drink we mean [country-specific definition of a standard drink]. *(Show card and graphic illustration)}*

1 – 25 drinks or more (iwon oti)

2 – 19 to 24 drinks (iwon oti)

3 – 16 to 18 drinks (iwon oti)

4 – 12 to 15 drinks (iwon oti)

5 – 9 to 11 drinks (iwon oti)

6 – 7 to 8 drinks (iwon oti)

7 – 5 to 6 drinks (iwon oti)

8 – 3 to 4 drinks (iwon oti)

9 – 2 drinks (iwon oti)

10 – 1 drink (iwon oti)

11 – LESS THAN 1 FULL DRINK *(If* ***both*** *CONS.GEN.FQ = 9 and GEN.QY=11, skip to MTND.EFCT) Q45*

98 – KO moo

99 –Ko dahun

[27]…GEN.MST.QY Ni osu mejila seyin (past 12 months), iwon oti melo ni o poju ti e mu lerin wakati merinlelogun? *(Show card)*

1 – 36 drinks or more (iwon oti)

2 – 25 to 35 drinks (iwon oti)

3 – 19 to 24 drinks (iwon oti)

4 – 16 to 18 drinks (iwon oti)

5 – 12 to 15 drinks (iwon oti)

6 – 9 to 11 drinks (iwon oti)

7 – 7 to 8 drinks (iwon oti)

8 – 5 to 6 drinks (iwon oti)

9 – 2 drinks (iwon oti)

10 – 1 drink (iwon oti)

11 – LESS THAN 1 FULL DRINK (iwon oti)

98 – KO moo

99 –Ko dahun

[28]…GEN.MST.FQ Ni osu mejila seyin, e melo ni e ma nmu oti lerin wakati mejilelelogun months [answer from CONS.GEN.MST.QY] *(Show card)*

1 – lojojumo

2 – 5 si 6 ni ose kan

3 –3 si 4 ni ose kan

4 –1 si 2 ni ose kan

5 – 2 si 3 ni osu kan

6 –ekan losu

7 – 6 si 11 ni osu mejila seyin

8 – 2 si 5 ni osu mejila seyin

9 – E kan ni osu mejila seyin

98 – KO moo

99 –Ko dahun

## 10.2 Beverage-Specific Quantity/Frequency

Niisinyi, a o bere nipa iye igba ti e n mu oti lerin osu mejila seyin ati nipa iye iwon oti na ti e nmu ni ojojumo.

[29]…BSFQ.BR.FQ Iye igba wo ni e mu oti **beer** lati osu mejila si isinyi? *(Show card)*

1 – lojojumo

2 – 5 si 6 ni ose kan

3 – 3 si 4 ni ose kan

4 – 1 si 2 ni ose kan

5 – 2 si 3 ni osu kan

6 – ekan losu

7 – 6 si 11 ni osu mejila seyin

8 – 2 si 5 ni osu mejila seyin

9 – Ekan ni Osu mejila seyin

10 – Rara ati rara ni osu mejila seyin *(Skip to CONS.BSFQ.WN.FQ) Q31*

98 – KO moo

99 –Ko dahun

[30]…BSFQ.BR.QY Ni ojo kan ti e ba mu beer, iye iwon beer melo ni e n mu?

__ __ *(Show card for standard drink)*

98 – KO DAJU

99 –Ko dahun

[31]…BSFQ.WN.FQ E melo ni e ti mu oti **wine** lerin oju mejila ti o koja? *(Show card)*

1 – lojojumo

2 – 5 si 6 ni ose kan

3 – 3 si 4 ni ose kan

4 – 1 si 2 ni ose kan

5 – 2 si 3 ni osu kan

6 – ekan losu

7 – 6 si 11 ni osu mejila seyin

8 – 2 si 5 ni osu mejila seyin

9 – Ekan ni Osu mejila seyin

10 – Rara ati rara ni osu mejila seyin *(Skip to BSFQ.SP.FQ)* [Q33]

98 – KO moo

99 –Ko dahun

[32]…BSFQ.WN.QY Ni ojo kan ti e ba mu **wine**, iye iwon melo ni e n mu?

__ __ *(Show card for standard drink)*

98 – KO moo

99 –Ko dahun

[33]…BSFQ.SP.FQ E melo ni e ti mu oti **spirit** lerin oju mejila ti o koja? *(Show card)*

1 – lojojumo

2 – 5 si 6 ni ose kan

3 – 3 si 4 ni ose kan

4 – 1 si 2 ni ose kan

5 – 2 si 3 ni osu kan

6 – ekan losu

7 – 6 si 11 ni osu mejila seyin

8 – 2 si 5 ni osu mejila seyin

9 – Ekan ni Osu mejila seyin

10 –Rara ati rara ni osu mejila seyin *(Skip to CONS.BSFQ.OTR.FQ) Q35*

98 – KO moo

99 –Ko dahun

[34]…BSFQ.SP.QY Ni ojo kan ti e ba mu **spirits**, iye iwon melo ni e n mu?

__ __ *(Show card for standard drink)*

98 – KO moo

99 –Ko dahun

[35]…BSFQ.OTR.FQ E melo ni e ti mu oti Emu fufun lerin osu mejila ti o koja (past 12 months)? *(Show card)*

1 – lojojumo

2 – 5 si 6 ni ose kan

3 – 3 si 4 ni ose kan

4 – 1 si 2 ni ose kan

5 – 2 si 3 ni osu kan

6 – ekan losu

7 – 6 si 11 ni osu mejila seyin

8 – 2 si 5 ni osu mejila seyin

9 – Ekan ni Osu mejila seyin

10 – Rara ati rara ni osu mejila seyin *(Skip to DRUK.FQ) Q37*

98 – KO moo

99 –Ko dahun

[36]…BSFQ.OTR.QY Ni ojo kan ti e ba mu emu fufun, iye iwon melo ni e n mu?

__ __ *(Show card for standard drink)*

98 – KO moo

99 –Ko dahun

## 10.3 Experience of Drunkenness/Intoxication

[37]…DRUK.FQ E melo ni osu mejila ti o koja ni e ti mu oti yo – boya ese yin ngbon tabi e n riran baibai tabi oro siso ko ja gere mo? *(Show card)*

1 – lojojumo

2 – 5 si 6 ni ose kan

3 – 3 si 4 ni ose kan

4 – 1 si 2 ni ose kan

5 – 2 si 3 ni osu kan

6 – ekan losu

7 – 6 si 11 ni osu mejila seyin

8 – 2 si 5 ni osu mejila seyin

9 – Ekan ni Osu mejila seyin

10 – Rara ati rara ni osu mejila seyin *(Skip to CONS.CXT1) Q39*

98 – KO moo

99 –Ko dahun

[38]…DRUK.NM Iwon oti melo ni e ma nmu ti e o fi yo? (1 drink is… *(Show card for standard drink)*)

__ __ DRINKS

98 – KO moo

99 –Ko dahun

## 10.4 Drinking Context

[39 ]Wayi, a o bere nipa iye igba ti e nmu oti ni awon ibi ti a se akajuwe won yi.

|  | Ni osu mejila seyin, e melo ni e mu oti ni igba ti…. | 1- lojojumo | 2 – Ekan lose | 3 – Ekan losu sugbon ki n se osose | 4 – Ekan ni osu mejila seyin sugbon kin se ososu | 5 – Rara ati rara ni osu mejila seyin | 98 – KO moo | 99 -Ko dahun |  | And how many drinks did you usually have? *(Skip if preceding answer is 5, 98, or 99)* | | |
| --- | --- | --- | --- | --- | --- | --- | --- | --- | --- | --- | --- | --- |
| …CXT1 | E lo je ounje ale ni ile ounje? |  |  |  |  |  |  |  |  | 1.NM |  | |
|  |  |  |  |  |  |  |  |  |  |  | 98 | 99 |
| …CXT2 | E lo je ounje osan ni ile ounje? |  |  |  |  |  |  |  |  | 2.NM |  | |
|  |  |  |  |  |  |  |  |  |  |  | 98 | 99 |
| …CXT3 | Ni beer parlour, bars, taverns, or cocktail lounges? |  |  |  |  |  |  |  |  | 3.NM |  | |
|  |  |  |  |  |  |  |  |  |  |  | 98 | 99 |
| …CXT4 | Ni ile elomiran tabi ni ibi apeje Party? |  |  |  |  |  |  |  |  | 4.NM |  | |
|  |  |  |  |  |  |  |  |  |  |  | 98 | 99 |
| …CXT5 | Ni ile re ni igba ale? |  |  |  |  |  |  |  |  | 5.NM |  | |
|  |  |  |  |  |  |  |  |  |  |  | 98 | 99 |
| …CXT6 | Ti awon ore ba wa ki e ni ile re? |  |  |  |  |  |  |  |  | 6.NM |  | |
|  |  |  |  |  |  |  |  |  |  |  | 98 | 99 |
| …CXT7 | Ti e ba jo wa pelu awon ore ni ibi isere ita gbangba? |  |  |  |  |  |  |  |  | 7.NM |  | |
|  |  |  |  |  |  |  |  |  |  |  | 98 | 99 |

[40]…CXT.MEAL Ni osu mejila seyin, e melo ni igbati e ba n njeun ni e nmu oti?

1 –gbogbo igba?

2 –oju idaji igba lo?

3 – Idaji?

4 – ko to idaji?

5 – ko si igba kankan?

98 – KO moo

99 –Ko dahun

[41]…CXT.SELF Ni osu mejila seyin, e melo ni eyin nikan ma ndamu oti?

1 –gbogbo igba?

2 –oju idaji igba lo?

3 – Idaji?

4 – ko to idaji?

5 – ko si igba kankan?

98 – KO moo

99 –Ko dahun

# MODULE 15: Motivations For/Against, and Effects of, Drinking – DRINKERS ONLY (MTCD)

[42]…EFCT Oti a ma da eniyan lamu ni orisirisi ona. Ti e ba mu oti, ewo ninu awon nkan wonyi ni o n sele si yin?

|  | Ti e ba mu oti tan… | 1 –Otito ni ni gbogbo igba | 2 – otito ni ni opolopo igba | 3 – otito ni ni igba miran | 4 – Ki n se otito ni opolopo igba | 5 –Ki n se otito ni igba kankan | 98 – KO moo | 99 –Ko dahun |
| --- | --- | --- | --- | --- | --- | --- | --- | --- |
| _1 | Nje ara yin ma n bale? |  |  |  |  |  |  |  |
| _2 | Nje inu yin ma ndun? |  |  |  |  |  |  |  |
| _3 | Nje oma nda bi ki e baa awon eyan ja lai ni idi |  |  |  |  |  |  |  |
| _4 | Nje o ma ndabi ki e jade lati lo sire pelu awon ore? |  |  |  |  |  |  |  |
| _5 | O rorun lati soro nipa isoro ati edun okan yin |  |  |  |  |  |  |  |
| _6 | E ma ngbagbe nipa isoro yin |  |  |  |  |  |  |  |
| _7 | E ma nse nkan miran ti e yio ka bamo ti o ba ya |  |  |  |  |  |  |  |
| _8 | Ibalo po a ma se dede bi e ti fe |  |  |  |  |  |  |  |
| _9 | Awon okunrin tabi obirin a ma ni ife mi pupo? |  |  |  |  |  |  |  |
| _10 | E ma nte ofin ni oju ti yio fi di oro ti olopa |  |  |  |  |  |  |  |
| _11 | E ma n gbadun ara yin dada |  |  |  |  |  |  |  |
| _12 | O ma ndabi pe ara yin ko ni Alafia |  |  |  |  |  |  |  |
| _13 | E ko ni ranti awon nkan miran ti o sele |  |  |  |  |  |  |  |

[43]…MOTV.FOR. Orisirisi idi ni awon eniyan fi n mu oti. Bawo ni awon nkan wonyi se se Pataki fun yin to?

|  |  | 1 – O se Pataki pupo | 2 –O se Pataki | 3 – Ki n se Pataki | 4 – Ki n se Pataki rara ati rara | 98 – KO moo | 99 –Ko dahun |
| --- | --- | --- | --- | --- | --- | --- | --- |
| _1 | Lati le gbajumo? |  |  |  |  |  |  |
| _2 | Nitori wipe awon eniyan miran n muu? |  |  |  |  |  |  |
| _3 | Lati le gbadun ounje daradara? |  |  |  |  |  |  |
| _4 | Fun Alafia ara? |  |  |  |  |  |  |
| _5 | Fun igbadun ara? |  |  |  |  |  |  |
| _6 | Ki ara le bale dada? |  |  |  |  |  |  |
| _7 | Lati pa ironu re? |  |  |  |  |  |  |
| _8 | Lati mu itiju kuro ati lati je akikanju? |  |  |  |  |  |  |
| _9 | Fun ajoyo? |  |  |  |  |  |  |
| _10 | Nitori wipe oti dunn lenu |  |  |  |  |  |  |
| _11 | Lati tan oungbe? |  |  |  |  |  |  |

[44]…MOTV.AGST Orisirisi idi ni owa ti awon eniyan fi ma n din oti mimu ku tabi lai mu oti rara. Bawo ni awon idi wonyi se je Pataki fun yin?

|  |  | 1 – O se Pataki pupo | 2 –O se Pataki | 3 – Kin se Pataki | 4 – Ki n se Pataki rara ati rara | 98 – KO moo | 99 –Ko dahun |
| --- | --- | --- | --- | --- | --- | --- | --- |
| _1 | Nitori mo fe ni oyun tabi tori wipe mo ti ni oyun? (ibere fun obirin nikan) |  |  |  |  |  |  |
| _2 | Nitori wipe oti dun lenu? |  |  |  |  |  |  |
| _3 | Nitori wipe mi o feran bi oti se n dami lamu? |  |  |  |  |  |  |
| _4 | Nitori wipe mo ti ri awon alebu ti oti le se? |  |  |  |  |  |  |
| _5 | Nitori wipe oti mimu elomiran ti se ipalara fun mi? |  |  |  |  |  |  |
| _6 | Nitori wipe oti le se akoba fun ise mi tabi eko ni ile iwe mi? |  |  |  |  |  |  |
| _7 | Nitori wipe oti mimu ti won ju o si n fa inawo danu? |  |  |  |  |  |  |
| _8 | Oti mimu lodi si esin? |  |  |  |  |  |  |
| _9 | Nitori wipe lati kekere mi ni won ti ko mi wipe n ko gbudo mu oti? |  |  |  |  |  |  |
| _10 | Nitori wipe oti ti se ipalara funmi ati wipe mi o fe di omutin? |  |  |  |  |  |  |
| _11 | Nitori wipe ojo ori mi si kere? |  |  |  |  |  |  |
| _12 | Nitori wipe awon ore ati ebi mi korira oti mimu? |  |  |  |  |  |  |
| _13 | Nitori wipe mo n lo ogun? |  |  |  |  |  |  |
| _14 | Nitori wipe moni are ara? |  |  |  |  |  |  |
| _15 | Oti mimu ko wumi rara? |  |  |  |  |  |  |

*All respondents completing this module skip to Module 17.*

# MODULE 16: Motivations For/Against, and Past Outcomes of, Drinking – NON-DRINKERS ONLY (MTND)

***[This question is for Past-Drinkers (Drank alcohol in the past but not in the past 12 months). Never drinkers go to MOTV.AGST]***

[45]…EFCT O ti ma n sise fun awon eniyan ni orisirisi ona. A fe mo nipa bi oti se nsise fun yin. Ni igba ti e nmu oti, bawo ni awon ibere yi se je otito si fun yin – Otito ni igbagbogbo, otito ni opo igba, otito ni igba miran, ki ise otito ni opo igba, ki ise otito ni igba kankan?

|  | Ti e ba mu oti tan… | 1 – Otito nigbagbogbo | 2 – Otito ni opo igba | 3 –Otito ni igba miran | 4 – Ki ise otito ni opo igba | 5 –ki ise otito ni iba kankan | 98 – KO moo | 99 –Ko dahun |
| --- | --- | --- | --- | --- | --- | --- | --- | --- |
| _1 | Nje ara yin ma n bale? |  |  |  |  |  |  |  |
| _2 | Nje inu yin ma ndun? |  |  |  |  |  |  |  |
| _3 | Nje oma nda bi ki e baa awon eyan ja lai ni idi |  |  |  |  |  |  |  |
| _4 | Nje o ma ndabi ki e jade lati lo sire pelu awon ore? |  |  |  |  |  |  |  |
| _5 | O rorun lati soro nipa isoro ati edun okan yin |  |  |  |  |  |  |  |
| _6 | E ma ngbagbe nipa isoro yin |  |  |  |  |  |  |  |
| _7 | E ma nse nkan miran ti e yio ka bamo ti o ba ya |  |  |  |  |  |  |  |
| _8 | Ibalo po a ma se dede bi e ti fe |  |  |  |  |  |  |  |
| _9 | A ma je ki awon okunrin tabi obirin feran yin? |  |  |  |  |  |  |  |
| _10 | E ma nte ofin ni oju ti yio fi di oro ti olopa |  |  |  |  |  |  |  |
| _11 | E ma n gbadun ara yin dada |  |  |  |  |  |  |  |
| _12 | O ma ndabi pe ara yin ko ni Alafia |  |  |  |  |  |  |  |
| _13 | E ko tile ni ranti awon nkan miran ti o sele |  |  |  |  |  |  |  |

***[This question is for Past-Drinkers (Drank alcohol in the past but not in the past 12 months). Never drinkers go to MOTV.AGST]***

[46]…MOTV.FOR Orisirisi idi ni o wa ti a fi n mu oti. Ni igba ti e n mu oti, bawo ni awon ibere wonyi se se Pataki si fun yin?

|  |  | 1 – O se Pataki pupo | 2 – IO se Pataki | 3 – Ko se Pataki pupo | 4 –Ko se Pataki rara ati rara | 98 – KO moo | 99 –Ko dahun |
| --- | --- | --- | --- | --- | --- | --- | --- |
| _1 | Lati le gbajumo? |  |  |  |  |  |  |
| _2 | Nitori wipe awon e lo miran n muu? |  |  |  |  |  |  |
| _3 | Lati le gbadun ounje daradara? |  |  |  |  |  |  |
| _4 | Fun Alafia ara? |  |  |  |  |  |  |
| _5 | Fun igbadun ara? |  |  |  |  |  |  |
| _6 | Ki ara le bale dada? |  |  |  |  |  |  |
| _7 | Lati pa ironu re? |  |  |  |  |  |  |
| _8 | Lati mu itiju kuro ati lati je akikanju? |  |  |  |  |  |  |
| _9 | Fun ajoyo? |  |  |  |  |  |  |
| _10 | Nitori wipe oti dunn lenu |  |  |  |  |  |  |
| _11 | Lati tan oungbe? |  |  |  |  |  |  |

***[This question is for both Past-Drinkers and Never Drinkers]***

[47]…MOTV.AGST People also have different reasons for limiting their drinking, or not drinking alcohol at all. How important would you say each of the following reasons is for you, personally? Would you say very important, important, not very important, or not at all important?

|  |  | 1 – Ose Pataki pupo | 2 –O se Pataki | 3 –Ko se Pataki pupo | 4 – Ko se ppataki rara ati rara | 98 – KO moo | 99 –Ko dahun |
| --- | --- | --- | --- | --- | --- | --- | --- |
| _1 | Nitori mo fe ni oyun tabi tori wipe mo ti ni oyun? (ibere fun obirin nikan) |  |  |  |  |  |  |
| _2 | Nitori wipe oti dun lenu? |  |  |  |  |  |  |
| _3 | Nitori wipe mi o feran bi oti se n dami lamu? |  |  |  |  |  |  |
| _4 | Nitori wipe mo ti ri awon alebu ti oti le se? |  |  |  |  |  |  |
| _5 | Nitori wipe oti mimu elomiran ti se ipalara fun mi? |  |  |  |  |  |  |
| _6 | Nitori wipe oti le se akoba fun ise mi tabi eko ni ile iwe mi? |  |  |  |  |  |  |
| _7 | Nitori wipe oti mimu ti won ju o si n fa inawo danu? |  |  |  |  |  |  |
| _8 | Oti mimu lodi si esin? |  |  |  |  |  |  |
| _9 | Nitori wipe lati kekere mi ni won ti ko mi wipe n ko gbudo mu oti? |  |  |  |  |  |  |
| _10 | Nitori wipe oti ti se ipalara funmi ati wipe mi o fe di omutin? |  |  |  |  |  |  |
| _11 | Nitori wipe ojo ori mi si kere? |  |  |  |  |  |  |
| _12 | Nitori wipe awon ore ati ebi mi korira oti mimu? |  |  |  |  |  |  |
| _14 | Nitori alafia ara? |  |  |  |  |  |  |
| _15 | Oti mimu ko wumi rara? |  |  |  |  |  |  |

# MODULE 17: Own and Others’ Perceptions of Alcohol and Other Substances (PCPN)

...GEN Bawo ni e se ro wipe awon nkan wonyi je otito *(Show card)*

[48]...GEN_1 Mimu oti je okan ninu awon igbadun aye.

1 – O damiloju pe beni

2 – beni

3 –ki ise iro ki si se otito

4 –Beko

5 – O damiloju pe beko

98 –KO moo

99 –Ko dahun

[49]...GEN_2 Mimu oti pelu eniyan je bi a se le fihan wipe a feran eni naa.

1 – O damiloju pe beni

2 – beni

3 –ki ise iro ki si se otito

4 –Beko

5 – O damiloju pe beko

98 – KO moo

99 –Ko dahun

[50]...GEN_3 Ko si ohun ti o dara ninu oti mimu.

1 – O damiloju pe beni

2 – beni

3 –ki ise iro ki si se otito

4 –Beko

5 – O damiloju pe beko

98 – KO moo

99 –Ko dahun

[51]…SITS Ni awon asiko wonyi, bawo ni o se ye ki a mu oti si– Ma se mu oti rara, Oti die sugbon ko gbudo fa idamu rara (1 or 2 drinks), oti die ti o fa idamu sugbon ti ko ni je ki eyan yo, ki eyan yo dara ni igbamiran, odara ki eyan ma yo ni igba gbogbo? *(Show card)*

|  |  | 1 – 0 iwon oti | 2 – iwon oti die ti ko le se nkankan fun eniyan (1 OR 2 DRINKS) | 3 – iwon oti ti o le jeki eniyan se bakan sugbon ki nse ki eyan yo | 4 – ki eyan mu oti yo je ohun ti o dara nigba miran | 5 – ki eyan mu oti yo dara ni opo igba | 98 – KO moo | 99 –Ko dahun |
| --- | --- | --- | --- | --- | --- | --- | --- | --- |
| _2 | Gege bi iya, ti o n ba awon omo sere |  |  |  |  |  |  |  |
| _3 | Gege bi baba, ti o n ba awon omo sere |  |  |  |  |  |  |  |
| _6 | Gege bi omokunrin ti o lo si ile oti pelu awon ore |  |  |  |  |  |  |  |
| _7 | Gege obirin ti o lo si ile oti pelu awon ore |  |  |  |  |  |  |  |
| _8 | Gege bi obirin ti o lo si ode pelu awon ti won jo nsise |  |  |  |  |  |  |  |
| _9 | Gege bi okunrin ti o lo si ode pelu awon ti won jo nsise |  |  |  |  |  |  |  |
| _12 | Fun okunrin ti o n je ounje ale pelu iyawo re |  |  |  |  |  |  |  |
| _13 | Fun obirin ti o n je ounje ale pelu oko re |  |  |  |  |  |  |  |

# MODULE 20: Adolescents and Young Adults

## 20.3 Emerging Adulthood

EMAD.PERC Bawo ni awon ibere won yi se je ododo si (otito ni dajudaju, otito ni, ko damiloju, beko, rara ati beko). *(Show card)*

[52]...EMAD.PERC_1 E ti dagba, e si ti to ojubo.

1 – otito ni dajudaju

2 – otito ni

3 – ko damiloju

4 – beko

5 – rara ati beko

98 – KO moo

99 –Ko dahun

[53]...EMAD.PERC_2 E ko nbere owo mo ni owo awon obi yin.

1 – otito ni dajudaju

2 – otito ni

3 – ko damiloju

4 – beko

5 – rara ati beko

98 – KO moo

99 –Ko dahun

[54]...EMAD.PERC_3 Opolopo igba ni e nda se akoso edun ati ayo okan mi lai so fun awon obi mi.

1 – otito ni dajudaju

2 – otito ni

3 – ko damiloju

4 – beko

5 – rara ati beko

98 – KO moo

99 –Ko dahun

JOWO KO AGOGO TI O LU NISINYI SILE HH…… MM………. (24hr clock)

# Jowo te siwaju pelu awon ibere ti o kuMODULE 21: Respondent Engagement, Recruiting and Screening (RCRT)

Ati n sumo ipari ibere ati idahun wonyi. Mo fe bere awon ibere die si.

[55]…ENG1 Ni ipele okan de mewa, ti okan je nko nife si rara, ti mewa je mo nife si lopolopo, bawo ni ele so wipe e ni ife si ise iwadi yi?

__ __

98 – ko moo

99 – ko dahun

[56]…ENG2 Pelu, ni ipele mewa, ti okan je rara ati rara, ti mewa je lopolopo, bawo ni e se gbadun bi e se kopa ninu ise iwadi yi?

__ __

98 – ko moo

99 – ko dahun

Gege bi mo ti so lati eyin wa, o se Pataki fun ayorisi rere ise yi ki a gba imo lati owo opo eniyan niipa oririsirisi ise si, iwa ati iriri won – E se pupo. A ni ero wipe awon ti oko pa ni ipele yi yio kopa ni ipele keji. Ni ipele keji, a o bere ibere ni ori ero ayelujara. Ki ise gbogbo awon ti o fe kopa ni a o basoro.

[57]...PART Nje e fe kopa ninu ipele keji ibere ati idahun yi?

1 – Beni, mo fe kopa ninu ipele keji (skip to ..SCRN) Q59

2 – Beko, mi o fe kopa ninu ipele keji

98 – Ko moo

99 – Ko dahun

[58] …PART.REF Nje e le so idi ti eko fe fi kopa ni nu ipele keji idahun ati ibere yi

______________________ (open-ended response)

98 – KO moo

99 –Ko dahun

…SCRN... Ki a to pari, mo ni awon ibere kukuru lati bere:

[59]…SCRN_STDN Ti a ba so wipe akeko je eniti owa ni ile iwe kan tabi ekeji, ewo ni a le fi se akajuwe yin.

1 – Mo nlo ile iwe gegebi akeko nikan ni

2 – Mo nlo ile iwe ni apa kan, mo sin se nkan miran ni apakeji

3 – Mi o nse omo ile iwe rara (Skip to …SCREEN.INT) Q61

98 – Ko moo

99 –Ko dahun

[60]…SCRN.EDUC Iru ile iwe wo ni e nlo:

1 –Secondary tabi grammar ( HIGH SCHOOL COURSE (e.g. high school, secondary school)

2 – Ile iwe giga (FURTHER EDUCATION COURSE (e.g. community college, technical college)

3 –University (HIGHER EDUCATION COURSE (e.g. university)

4 – OTHER (please specify) _______________________________________

98 – KO moo

99 –Ko dahun

[61]…SCRN.INT E me lo ni e nlo ero ayelujara?

1 – Ojojumo

2– Ekan lose

3 –Ekan losu

4 –Ekan losu meji

5 –ko to ekan losu

6- mi o ni anfani si ero aiyelujara

98 – Ko moo

99 – kodahun

[62]…SCRN.CHCK Iye igba melo ni e n wo email yin?

1 – Ojojumo

2– Ekan lose

3 –Ekan losu

4 – Ekan losu meji

5 –Mi o kin wo rara

6-Mi o ni email

98 – Ko moo

99 – kodahun

(IF PART=2 (DOES NOT WANT TO PARTICIPATE) END INTERVIEW

(IF SCRN.INT = 5 OR 6 **AND** SCRN.CHCK = 5 OR 6, THEN END.)

(IF SCRN.INT = 1-4 **AND** SCRN.CHCK = 5 OR 6, THEN GO TO PART.CNTC.OTH)

(IF SCRN.INT = 1-4 **AND** SCRN.CHCK = 1-4, CONTINUE)

[63]…PART.EMAIL A o fe ba yin soro lori email address yin. Nje e ni?

1 – Beni, EMAIL ADDRESS mi niyi: ______________________________________________

***(Interviewer: Verify email address by asking respondent to repeat address.)***

2 – Beko, mi o ni email address

98 –Ko moo

99 – Rara

[64]…PART.CNTC.OTH Nje e le fun wa ni ona miran ti a le fi ba yin soro yato si email address gegebi number phone yin?

1 – Beni, Phone number mi niyi: __________________________________

***(Interviewer: Verify by asking respondent to repeat.)***

2.Beni, adresi mi ni yi______________________________________

***(Interviewer: Verify by asking respondent to repeat.)***

3 – Rara, mi o ni ona miran ti e le fi bami soro

98 – Ko moo

99 – Ko dahun

[E se pupo. Ati pari.]
