# Supplementary material for: Context and culture associated with alcohol use amongst youth in major urban cities: A cross-country population based survey
Source: PLoS One. 2017 Nov 20;12(11):e0187812. doi: 10.1371/journal.pone.0187812 (PMC5695777; doi:10.1371/journal.pone.0187812)
Supplement: S1 Table — (DOCX) [file pone.0187812.s008.docx]

Supporting Table 1 - Additional details on sampling

Wuhan (China)

| **Description** | **Administrative Districts in Wuhan** | **Population distribution**  **%** | **Estimated sample size required** |
| --- | --- | --- | --- |
| **Wuhan** is divided into 13 Administrative Districts (AD). The first stage of sampling involved stratifying the sample by the AD proportionally to population size. The next stage involved randomly selecting areas within each AD; followed by the final stage of randomly selecting n households per community with a random starting point (after determining the number of households in the community). | Jiang-An | 8.3 | 132 |
|  | Jiang-han | 5.8 | 93 |
|  | Qiao-kou | 6.4 | 103 |
|  | Han-yang | 6.7 | 108 |
|  | Wu-chang | 14.0 | 223 |
|  | Qing-shan | 5.4 | 87 |
|  | Hong-shan | 11.1 | 177 |
|  | Han-nan | 1.3 | 21 |
|  | Dong-Xi-Hu | 3.2 | 52 |
|  | Cai-dian | 5.4 | 86 |
|  | Jiang-xia | 7.1 | 113 |
|  | Huang-pi | 13.7 | 219 |
|  | Xin-zhou | 11.6 | 186 |
|  | Total | 100 | 1600 |

Initial sample size - Moscow

|  | **Moscow Administrative Districts** | **Population distribution**  **%** | **Estimated sample size required** |
| --- | --- | --- | --- |
| **Moscow** is divided into 12 major Administrative Districts (AD); 10 AD of so-called ‘Old Moscow’, with administrative borders as before July 1, 2012 were included in the sample. The first stage of the sample selection involved stratification of the sample by the 10 ADs proportionally to population size. The next stage involves randomly selecting Statistical Areas within each AD; followed by randomly selecting a start point every n-th household per Statistical Area; followed by the final stage which was randomly selecting 1 person within each household. | Central | 6.0 | 96 |
|  | Northern | 9.9 | 159 |
|  | North-Eastern | 12.0 | 191 |
|  | Eastern | 12.5 | 199 |
|  | South-Eastern | 11.5 | 185 |
|  | Southern | 14.6 | 231 |
|  | South-Western | 12.2 | 195 |
|  | Western | 11.0 | 177 |
|  | North-Western | 7.9 | 128 |
|  | Zelenograd | 2.4 | 39 |
|  | **Total** | 100 | 1 600 |

Initial sample size - llorin

|  | **LGAs in Ilorin** | **Population distribution**  **%** | **Estimated sample size required** |
| --- | --- | --- | --- |
| **Ilorin** is divided into 3 major administrative areas called LGAs. Each LGA is further divided into political wards The sample was stratified by the 3 LGAs proportionally to population size. The next stage involves randomly selecting 5 wards within each LGA; followed by randomly selecting settlement/neighbourhoods within each ward proportional to population size, followed by randomly selecting 3 streets within each settlement, with the final stage randomly selecting a starting point, then every house on either side of the street until the required sample size for that neighbourhood was obtained or exhausted. | Ilorin West | 46.9 | 750 |
|  | Ilorin East | 26.3 | 429 |
|  | Ilorin South | 26.8 | 421 |
|  | Total | 100 | 1600 |

Initial sample size - Montevideo

|  | **Departments in MMA of Uruguay** | **Population distribution**  **%** | **Estimated sample size required** |
| --- | --- | --- | --- |
| **Uruguay** is divided into 19 departments. The Montevideo Metropolitan Area (MMA) territory includes the whole department of Montevideo (the capital city) and parts of the departments of San Jose and Canelones. The sample was stratified by the 3 departments proportionally to population size. The next stage involves randomly selecting 62 neighbourhoods in Montevideo and 6 neighbourhoods in the other 2 departments; followed by randomly selecting blocks within each neighbourhood proportional to population size, followed by the final stage of selecting 5 households within each block under a ‘protocol of start and jump’. | Montevideo | 69.6 | 1113 |
|  | Canelones | 25.1 | 401 |
|  | San Jose | 5.3 | 85 |
|  | Total | 100 | 1600 |
