# Supplementary material for: Context and culture associated with alcohol use amongst youth in major urban cities: A cross-country population based survey
Source: PLoS One. 2017 Nov 20;12(11):e0187812. doi: 10.1371/journal.pone.0187812 (PMC5695777; doi:10.1371/journal.pone.0187812)
Supplement: S2 Table — (DOCX) [file pone.0187812.s009.docx]

Supporting Table 2: Response rates by City/Country

|  | **Ilorin  (Nigeria)** | | **Montevideo (Uruguay)** | | **Moscow  (Russia)** | | **Wuhan  (China)** | |
| --- | --- | --- | --- | --- | --- | --- | --- | --- |
|  | **n** | **%** | **n** | **%** | **n** | **%** | **n** | **%** |
| **Sample** | 1850 |  | 13820 |  | 9319 |  | 1675 |  |
|  |  |  |  |  |  |  |  |  |
| **Ineligible** | 386 |  | 11998 |  | 4262 |  | 29 |  |
| Not a housing unit (business, government, or other office/institution) | 304 | 16.4 | 2559 | 21.3 | 14 | 0.3 | - | - |
| Vacant housing unit | 44 | 2.4 | 480 | 4.0 | 754 | 17.7 | - |  |
| Seasonal, vacation, or temporary residence | - | - | 1 | 0.0 | - | - | - | -- |
| No eligible respondent (Respondent must be aged between 18 – 34 years old and has lived in the city/metropolitan area for at least 6 months) | 38 | 2.1 | 5662 | 47.2 | 2696 | 63.3 | 14 | 48.3 |
| Other reason |  |  | 3296 | 27.5 | 798 | 18.7 | 15 | 51.7 |
|  |  |  |  |  |  |  |  |  |
| **Eligible sample** | 1464 |  | 1828 |  | 3407 |  | 1646 |  |
| Refusal by respondent | 5 | 0.3 | 99 | 5.4 | 1538 | 45.1 |  |  |
| Non-contacted (after 3 attempts or not attempted for other reason) | 36 | 1.9 | 129 | 7.1 | 1052 | 30.1 | 4 | 0.2 |
| Incapacitated and unable to be interviewed (i.e. too ill, hearing impaired) | - | - | - | - | 62 | 1.8 |  |  |
| Not Speaking Russian |  |  | - | - | 298 | 8.7 |  |  |
| Terminated interview by respondent or interviewer | 31 | 1.7 | - | - | 21 | 0.6 |  |  |
| Respondent unavailable | 1 | - | - | - | 43 | 1.3 |  |  |
| Other reason |  |  | - | - | 393 | 11.5 |  |  |
|  |  |  |  |  |  |  |  |  |
| **Completed interviews** | 1391 |  | 1600 |  | 1650 |  | 1642 |  |
|  |  |  |  |  |  |  |  |  |
| **Participation rate** |  | 97.4 |  | 94.2 |  | 70.1 |  | 100.0 |
| **Response rate** |  | 95.0 |  | 87.5 |  | 48.4 |  | 99.8 |

The participation rate is determined by the following formula: *Participation rate = completed interviews / (initial eligible sample - non-contact after fifteen attempts*

The response rate is determined by the following formula: *Response rate = completed interviews / initial eligible sample*
