# Supplementary material for: Context and culture associated with alcohol use amongst youth in major urban cities: A cross-country population based survey
Source: PLoS One. 2017 Nov 20;12(11):e0187812. doi: 10.1371/journal.pone.0187812 (PMC5695777; doi:10.1371/journal.pone.0187812)
Supplement: S3 Table — (DOCX) [file pone.0187812.s010.docx]

Supporting Table 3: Demographic characteristics by city/country

|  | **Ilorin (Nigeria)** | | **Montevideo (Uruguay)** | | **Moscow (Russia)** | | **Wuhan (China)** | |
| --- | --- | --- | --- | --- | --- | --- | --- | --- |
|  | **n** | **% (95% CI)** | **n** | **% (95% CI)** | **n** | **% (95% CI)** | **n** | **% (95% CI)** |

| Females |  | 47.6 (44.5 - 50.7) |  | 49.2 (46.4 - 52.0) |  | 49.2 (46.6 - 51.8) |  | 51.4 (48.4 - 54.5) |
| --- | --- | --- | --- | --- | --- | --- | --- | --- |
| Males |  | 52.4 (49.3 - 55.5) |  | 50.8 (48.0 - 53.6) |  | 50.8 (48.2 - 53.4) |  | 48.6 (45.5 - 51.6) |
| **Age group** |  |  |  |  |  |  |  |  |
| 18-24 years |  | 48.0 (44.8 - 51.2) |  | 42.7 (39.9 - 45.5) |  | 38.1 (35.5 - 40.7) |  | 53.2 (50.3 - 56.0) |
| 25-29 years |  | 30.1 (27.2 - 33.1) |  | 28.5 (26.1 - 31.1) |  | 31.7 (29.3 - 34.2) |  | 25.1 (22.9 - 27.4) |
| 30-34 years |  | 21.9 (19.4 - 24.7) |  | 28.8 (26.3 - 31.5) |  | 30.2 (28.2 - 32.4) |  | 21.7 (19.8 - 23.8) |
| **Marital status** |  |  |  |  |  |  |  |  |
| Married |  | 27.7 (24.9 - 30.8) |  | 47.9 (45.1 - 50.7) |  | 38.2 (35.9 - 40.6) |  | 44.3 (41.5 - 47.1) |
| Divorced/ separated / widowed |  | 1.6 (0.9 - 2.6) |  | 2.7 (1.8 - 3.9) |  | 5.1 (4.2 - 6.2) |  | 0.5 (0.3 - 0.8) |
| Never married |  | 70.5 (67.4 - 73.4) |  | 49.4 (46.6 - 52.2) |  | 56.5 (54.0 - 58.9) |  | 54.9 (52.1 - 57.8) |
| **Do you have any children?** |  |  |  |  |  |  |  |  |
| Yes |  | 31.0 (28.1 - 34.1) |  | 43.2 (40.5 - 46.0) |  | 32.9 (30.7 - 35.2) |  | 36.1 (33.5 - 38.8) |
| No |  | 69.0 (65.9 - 71.9) |  | 56.7 (53.9 - 59.5) |  | 67.0 (64.7 - 69.2) |  | 63.6 (61.0 - 66.3) |
| **Religion** |  |  |  |  |  |  |  |  |
| Christian |  | 0.1 # |  | 47.4 (44.6 - 50.2) |  | 12.8 (11.2 - 14.5) |  | 81.6 (79.3 - 83.8) |
| Muslim |  | 33.9 (31.1 - 36.8) |  | 49.1 (46.3 - 51.9) |  | 78.2 (76.1 - 80.1) |  | 1.2 (0.7 - 1.9) |
| Agnostic/atheist |  | 65.9 (62.9 - 68.7) |  | - |  | 4.2 (3.3 - 5.4) |  | - |
| Other |  | 0.1 # |  | 3.4 (2.5 - 4.5) |  | 1.0 (0.6 - 1.7) |  | 3.6 (2.6 - 5.1) |
| **Highest education** |  |  |  |  |  |  |  |  |
| Secondary school or less |  | 57.1 (54.0 - 60.1) |  | 58.0 (55.2 - 60.7) |  | 14.5 (12.7 - 16.4) |  | 39.2 (36.4 - 42.1) |
| Vocational/professional/Non-university tertiary education |  | 28.8 (26.1 - 31.7) |  | 33.2 (30.6 - 35.9) |  | 46.3 (43.7 - 48.8) |  | 51.6 (48.6 - 54.5) |
| University degree or higher |  | 14.0 (12.1 - 16.2) |  | 8.8 (7.4 - 10.5) |  | 39.2 (36.8 - 41.7) |  | 8.8 (7.0 - 10.8) |
| **Are you currently a student?** |  |  |  |  |  |  |  |  |
| Yes |  | 39.4 (36.4 - 42.5) |  | 35.1 (32.6 - 37.6) |  | 27.3 (25.0 - 29.8) |  | 20.1 (17.6 - 22.9) |
| No |  | 60.5 (57.4 - 63.6) |  | 64.9 (62.4 - 67.4) |  | 72.7 (70.2 - 75.0) |  | 79.5 (76.7 - 82.0) |
| **Employment status** |  |  |  |  |  |  |  |  |
| Employed full-time |  | 33.8 (30.8 - 36.8) |  | 49.8 (47.0 - 52.6) |  | 61.2 (58.7 - 63.7) |  | 68.0 (65.1 - 70.8) |
| Employed part-time |  | 10.8 (8.9 - 13.0) |  | 17.9 (16.0 - 20.0) |  | 8.8 (7.4 - 10.4) |  | 3.1 (2.2 - 4.5) |
| Unemployed |  | 16.8 (14.5 - 19.3) |  | 17.1 (15.0 - 19.5) |  | 2.0 (1.4 - 2.9) |  | 3.5 (2.5 - 4.8) |
| Student |  | 33.6 (30.7 - 36.6) |  | 12.8 (11.1 - 14.7) |  | 0.3 (0.1 - 0.9) |  | 17.1 (14.8 - 19.7) |
| Other |  | 5.0 (3.8 - 6.6) |  | 2.4 (1.6 - 3.5) |  | 27.4 (25.1 - 29.7) |  | 7.6 (6.2 - 9.3) |

CI: Confidence Interval. # Insufficient numbers. Note: The weighting of data can result in rounding discrepancies or totals not adding. Don’t know/refused not included.
